# Trends in esophageal and esophagogastric junction cancer research from 2007 to 2016: A bibliometric analysis

**DOI:** 10.1097/MD.0000000000006924

**Published:** 2017-05-19

**Authors:** Yan Miao, Ran Liu, Yuepu Pu, Lihong Yin

**Affiliations:** Key Laboratory of Environmental Medicine Engineering of Ministry of Education, School of Public Health, Southeast University, Nanjing, Jiangsu Province, China.

**Keywords:** bibliometric, CiteSpace, esophageal cancer, esophagogastric junction cancer

## Abstract

**Background:**

This study aimed to analyze the scientific outputs of esophageal and esophagogastric junction (EGJ) cancer and construct a model to quantitatively and qualitatively evaluate pertinent publications from the past decade.

**Methods:**

Publications from 2007 to 2016 were retrieved from the Web of Science Core Collection database. Microsoft Excel 2016 (Redmond, WA) and the CiteSpace (Drexel University, Philadelphia, PA) software were used to analyze publication outcomes, journals, countries, institutions, authors, research areas, and research frontiers.

**Results:**

A total of 12,978 publications on esophageal and EGJ cancer were identified published until March 23, 2017. The *Journal of Clinical Oncology* had the largest number of publications, the USA was the leading country, and the University of Texas MD Anderson Cancer Center was the leading institution. Ajani JA published the most papers, and Jemal A had the highest co-citation counts. Esophageal squamous cell carcinoma ranked the first in research hotspots, and preoperative chemotherapy/chemoradiotherapy ranked the first in research frontiers.

**Conclusion:**

The annual number of publications steadily increased in the past decade. A considerable number of papers were published in journals with high impact factor. Many Chinese institutions engaged in esophageal and EGJ cancer research but significant collaborations among them were not noted. Jemal A, Van Hagen P, Cunningham D, and Enzinger PC were identified as good candidates for research collaboration. Neoadjuvant therapy and genome-wide association study in esophageal and EGJ cancer research should be closely observed.

## Abstract 2

Supplemental Digital Content is available in the text

## 1 Introduction

Esophageal cancer originates in the esophagus—a tubular structure that runs from the throat and the stomach.^[1]^ The cancer establishes in the inner of the esophagus and then spreads over the other layers of the esophagus and finally to other parts of the human body; this process is termed as “metastasis.”^[2]^ Esophageal cancer is the eighth most common cancer in the world,^[1]^ and accounted for nearly 40 million deaths in 2012. The number of deaths due to esophageal cancer in 1990 was 34.5 million.^[3]^ In Western nations, the lower third of the esophagus is the most common site of esophageal cancer, and a frequent involvement of the esophagogastric junction (EGJ) is noted.^[4]^ Because of a notable increase in the prevalence of EGJ involvement in esophageal cancer in recent decades, EGJ cancer has become a public health concern.

Until now, many journals have published articles on esophageal or EGJ cancer. Nevertheless, few attempts have been made to systematically analyze the data from these publications. Bibliometrics has been used in various fields to assess the productivity of nations, institutions, and authors, and identify international collaborations, research hotspots, and frontiers in particular areas.

This article qualitatively and quantitatively evaluates esophageal and EGJ cancer research from 2007 to 2016. Our purpose is to estimate the scientific outcomes of esophageal and EGJ cancer and explore its trends and frontiers.

## 2 Materials and methods

### 2.1 Source of the data and search strategy

Literature was explored from the Science Citation Index-Expanded (SCI-E) and the Social Science Citation Index (SSCI) of the Web of Science Core Collection (WoSCC) of Thomson Reuters on March 23, 2017. The data was downloaded from the public database, and there was no ethical question about the data. Ethical approval was not applicable here.

The following terms were used to retrieve related publications from 2007 to 2016: (“esophageal cancer^∗^”) OR (“esophageal neoplasm^∗^”) OR (“esophagus cancer^∗^”) OR (“esophagus neoplasm^∗^”) OR (“cancer of the esophagus”) OR (“cancer of esophagus”)) OR (“esophagogastric junction” AND (“neoplas^∗^” OR “cancer^∗^” OR “tumor^∗^” OR “carcinoma^∗^” OR “malign^∗^”)) OR (“gastroesophageal junction” AND (“neoplas^∗^” OR “cancer^∗^” OR “tumor^∗^” OR “carcinoma^∗^” OR “malign^∗^”). All electronic searches were performed on the same day, March 23, 2017.

### 2.2 Data collection

All data were independently collected by 2 authors (YM and LY) and downloaded in txt format. The data were imported to Microsoft Excel 2016 and CiteSpace and quantitatively and qualitatively analyzed.

### 2.3 Statistical methods

WoSCC was used to analyze the characteristics of the publications, including the countries or territories, institutions, journals, authors, research areas, document types, and languages.

CiteSpace was used to identify the collaborations between countries/institutions/authors, perform co-citation analysis in authors and references, perform a co-occurrence analysis of the keywords, and generate knowledge maps of all the items mentioned above.

## 3 Results

### 3.1 Publication outputs

From 2007 to 2016, 10 document types were found in 12,978 publications. Most publications were research articles (72.64%), followed by meeting abstracts (13.95%), and review articles (8.57%) (Supplemental Table 1). Approximately 97.66% of the publications were in English, whereas the remaining 2.34% of the publications were in other languages (Supplemental Table 2).

**Figure 1:**
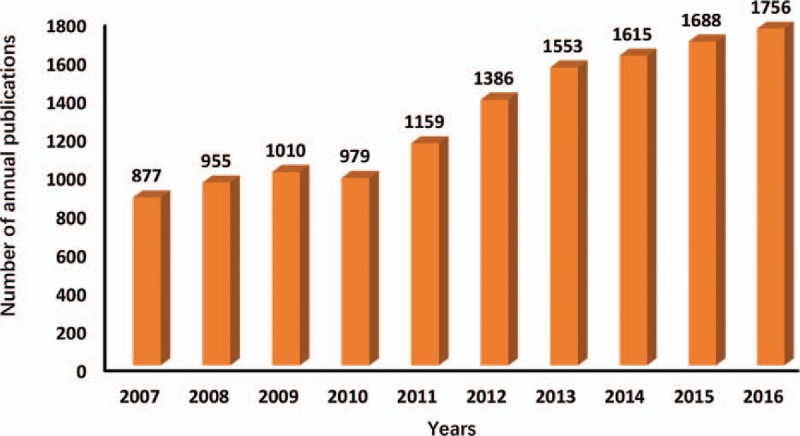
The number of annual publications on esophageal and esophagogastric junction cancer from 2007 to 2016.

The distribution of annual publications was presented in different time stages (Fig. 1). Except for a slight decline in 2010, the overall trend of publication increased from 877 publications in 2007 to 1756 publications in 2016.

### 3.2 Distribution by journals

**Figure 2:**
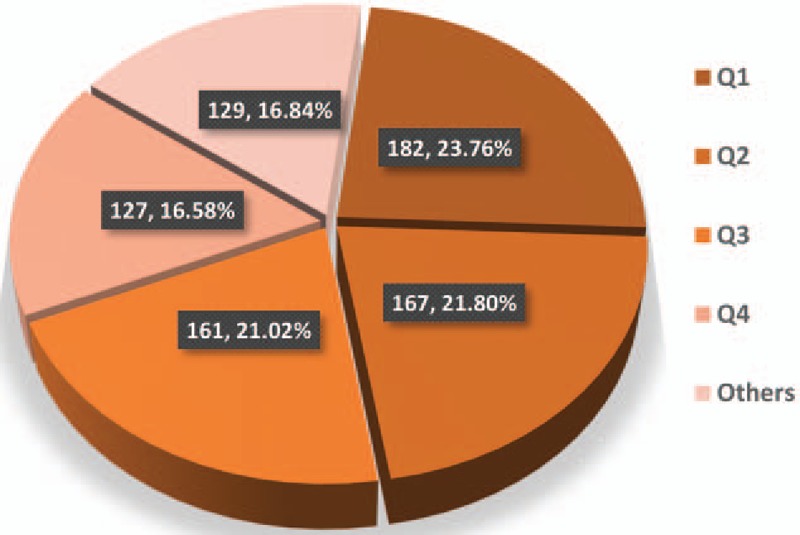
JCR standards classify journals contributed to publications on esophageal and esophagogastric junction cancer from 2007 to 2016. JCR = Journal Citation Reports.

In total, 766 academic journals have published articles on esophageal and EGJ cancer (Supplemental Table 3). According to the Journal Citation Reports (JCR) 2015 standards, 182 journals (23.76%) were classified as Q1, 167 (21.80%) were classified as Q2, 161 (21.02%) were classified as Q3, and 127 (16.58%) were classified as Q4 (Fig. 2). The remaining (129 journals, 16.84%) did not meet the JCR 2015 standards.

Among the top 15 journals (Table 1), the *Journal of Clinical Oncology* (impact factor (IF) 2015, 20.982) contributed to the largest number of publications on esophageal and EGJ cancer (465 publications, 3.58%), followed by *Diseases of the Esophagus* (IF2015, 2.146; 422 publications, 3.25%), *International Journal of Radiation Oncology Biology Physics* (IF2015, 4.495; 411 publications, 3.17%), and *Annals of Surgical Oncology* (IF2015, 3.655; 328 publications, 2.53%).

**Table 1:**
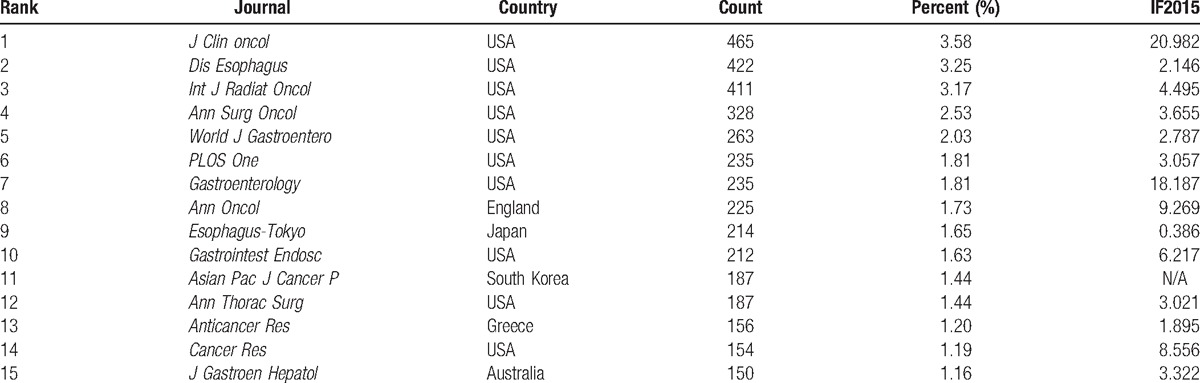
The top 15 journals contributed to publications on esophageal and esophagogastric junction cancer from 2007 to 2016.

### 3.3 Distribution by countries and institutions

#### 3.3.1 Analyses of countries

**Figure 3:**
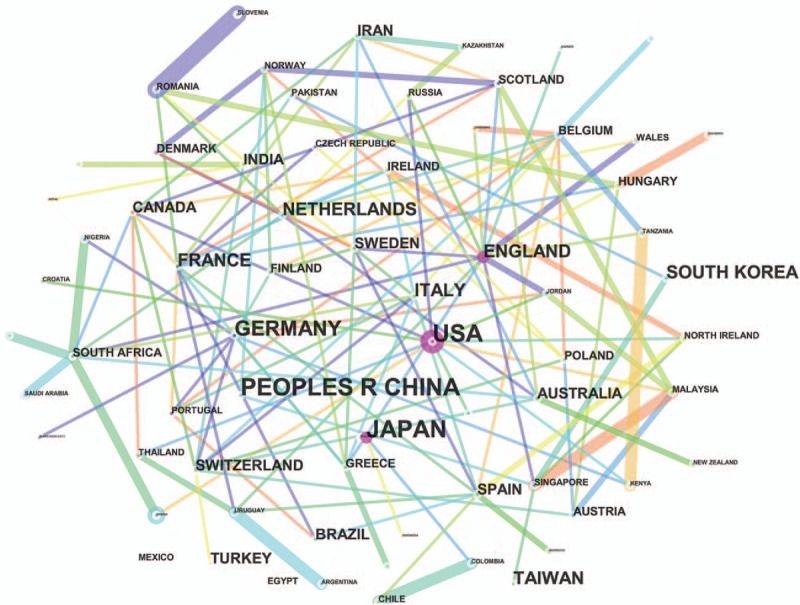
Network map of countries/regions contributed to publications on esophageal and esophagogastric junction cancer from 2007 to 2016.

The 12,978 publications on esophageal and EGJ cancer were contributed by 91 countries/regions (Supplemental Table 4). There were extensive collaborations between countries/regions (Fig. 3). In relation to the top 10 countries that contributed to esophageal cancer research (Table 2), the USA had the largest number of publications (3246), followed by China (2932), Japan (2267), and Germany (940).

**Table 2:**
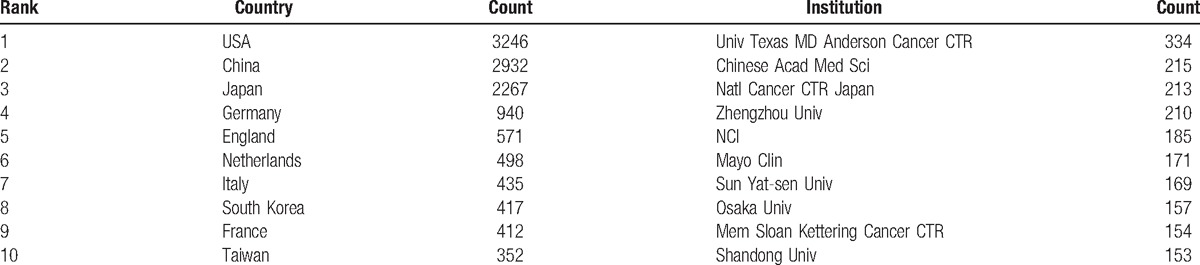
The top 10 countries and institutions contributed to publications on esophageal and esophagogastric junction cancer from 2007 to 2016.

#### 3.3.2 Institutions analysis

**Figure 4:**
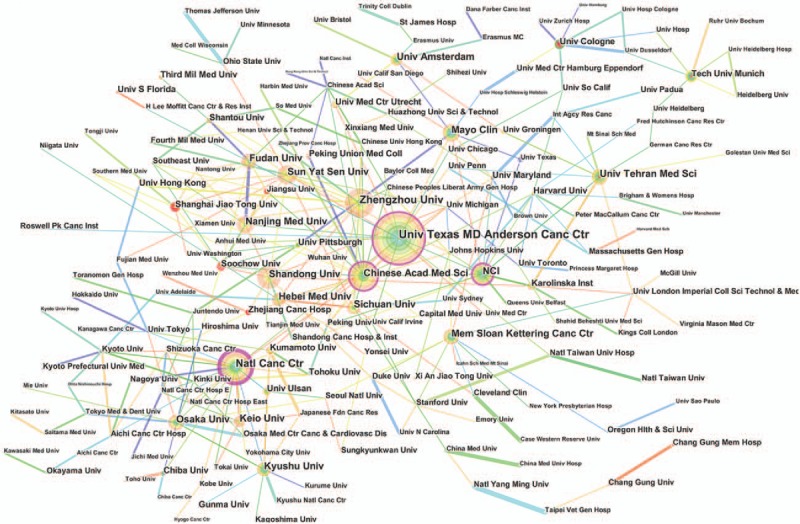
Network map of institutions contributed to publications on esophageal and esophagogastric junction cancer from 2007 to 2016.

Over 2700 institutions contributed to the publications on esophageal and EGJ cancer (Supplemental Table 5). Compared with countries, there was very little cooperation between the institutions (Fig. 4). The top 10 institutions contributed to 1961 articles, which accounted for 15.11% of the total number of publications. The University of Texas MD Anderson Cancer Center led the first research echelon, followed by the Chinese Academy of Medical Sciences, Peking Union Medical College, National Cancer Center Japan, and Zhengzhou University (Table 2).

### 3.4 Distribution by authors

**Figure 5:**
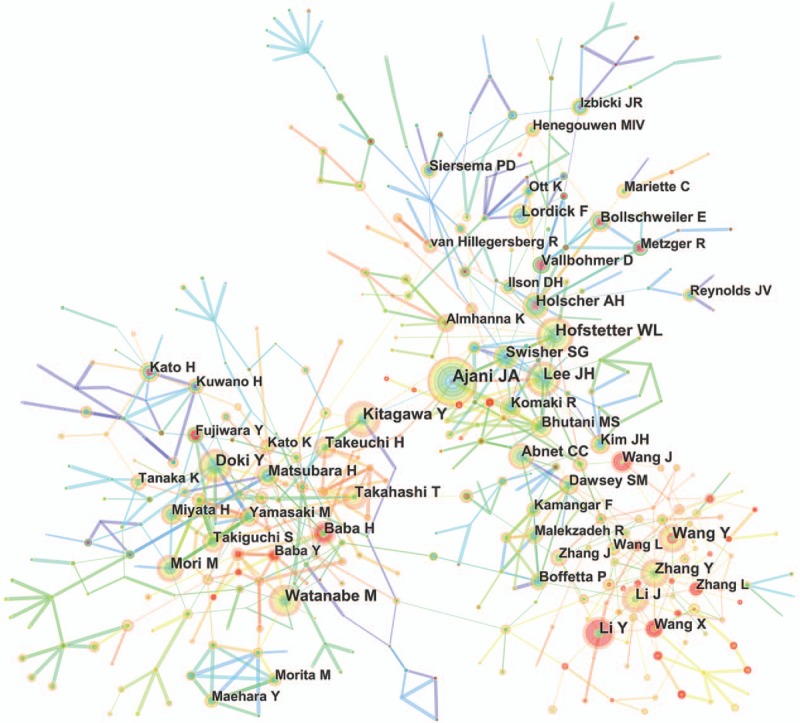
Network map of authors contributed to publications on esophageal and esophagogastric junction cancer from 2007 to 2016.

More than 13,000 authors contributed to the total number of publications (Supplemental Table 6). The cooperation between authors was presented in a network map (Fig. 5). For authors who had the most publications (Table 3), Ajani JA ranked the first (143 publications), followed by Hofstetter WL (116 publications), Kitagawa Y (105 publications), and Lee JH (104 publications).

**Table 3:**
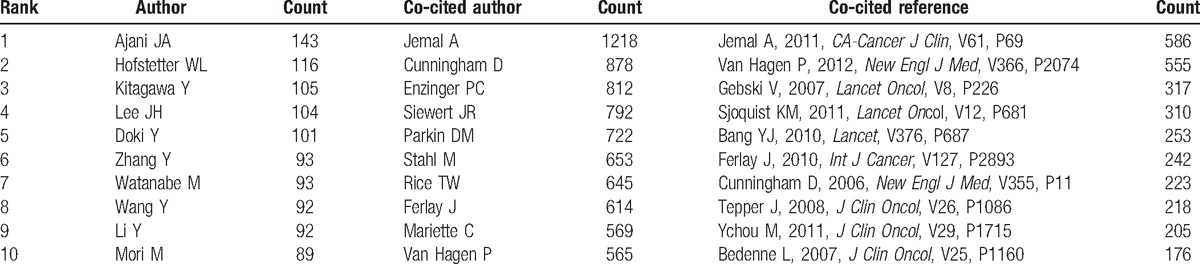
The top 10 active authors, co-cited authors, and co-cited references of publications on esophageal and esophagogastric junction cancer from 2007 to 2016.

**Figure 6:**
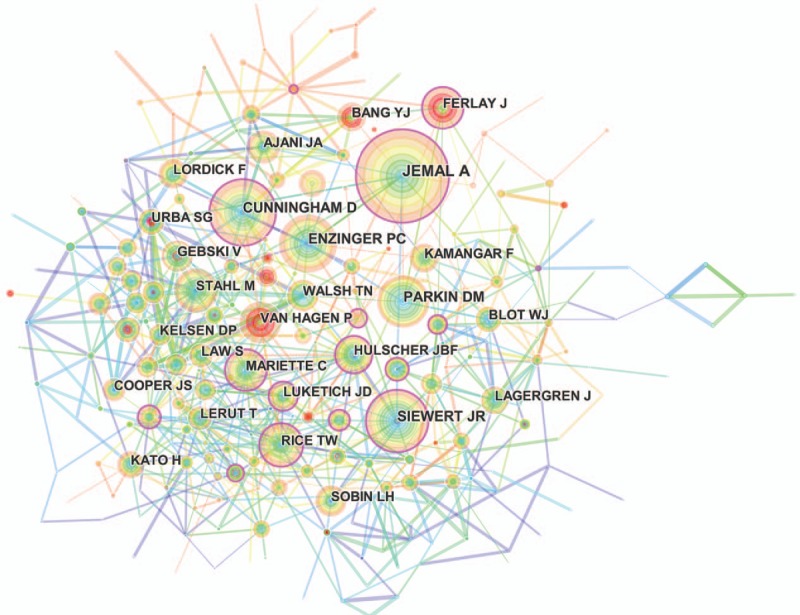
Network map of co-cited authors contributed to publications on esophageal and esophagogastric junction cancer from 2007 to 2016.

CiteSpace detected the information on author citations and presented it through a network map (Fig. 6). According to the top 10 co-cited authors (Table 3) (Supplemental Fig. 1), Jemal A (1218 citations) ranked first, followed by Cunningham D (878 citations), Enzinger PC (812 citations), and Siewert JR (792 citations).

### 3.5 Analysis of references

**Figure 7:**
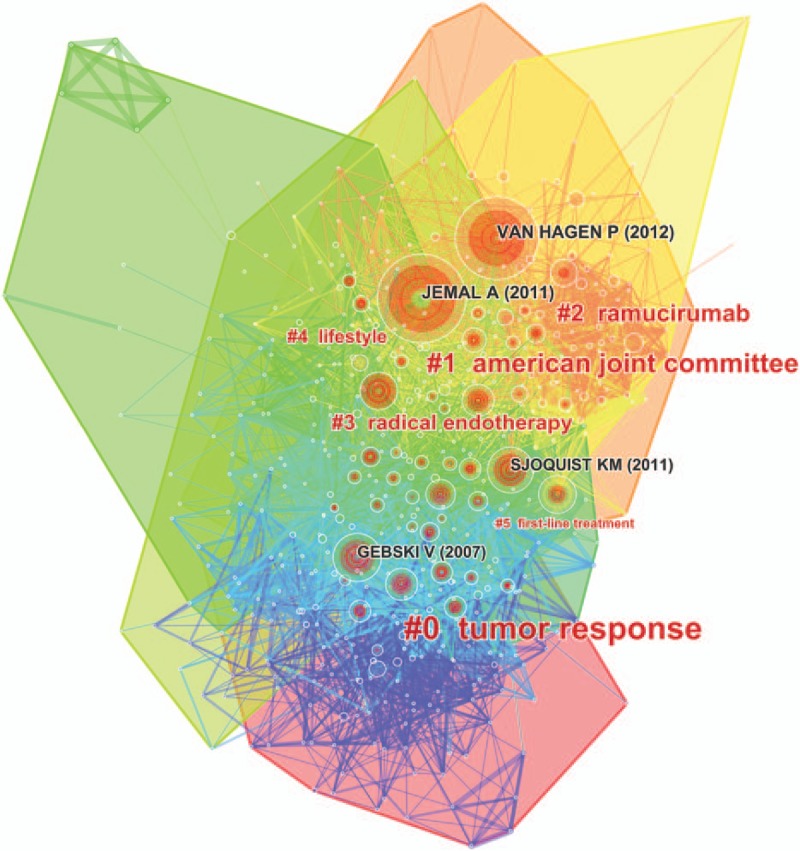
Reference co-citation map of publications on esophageal and esophagogastric junction cancer from 2007 to 2016.

**Figure 8:**
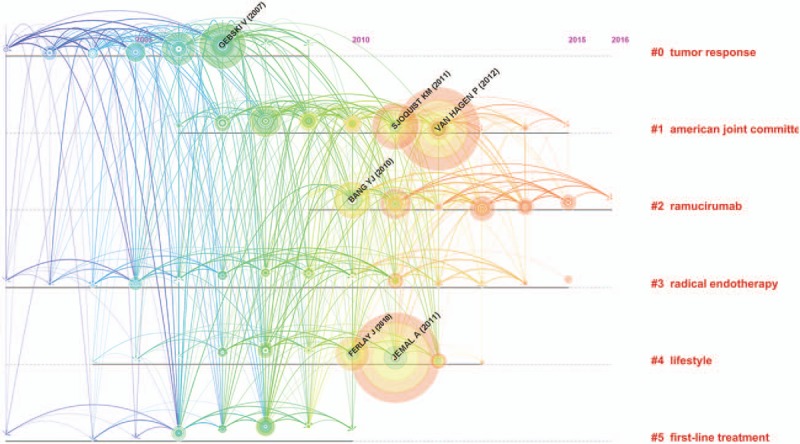
Reference co-citation (timeline view) map of publications on esophageal and esophagogastric junction cancer from 2007 to 2016.

The analysis of references is one of the most significant indicators of bibliometrics. The co-citation map of references estimated the scientific relevance of the publications (Fig. 7). In this map, the modularity Q score was greater than 0.5 (0.5136) (Supplemental Fig. 2), which means the network was reasonably divided into loosely coupled clusters. The average silhouette score was greater than 0.5 (0.5405) (Supplemental Fig. 2), suggesting that the homogeneity of these clusters on average was acceptable. All clusters were labeled by index terms extracted from the references (Supplemental Fig. 3). The largest cluster #0 was labeled as “tumor response,” followed by the second largest cluster #1, labeled as “American joint committee,” and the third largest cluster #2, labeled as “ramucirumab.” These clusters were also shown in a timeline view (Fig. 8).

### 3.6 Analysis of research areas

**Figure 9:**
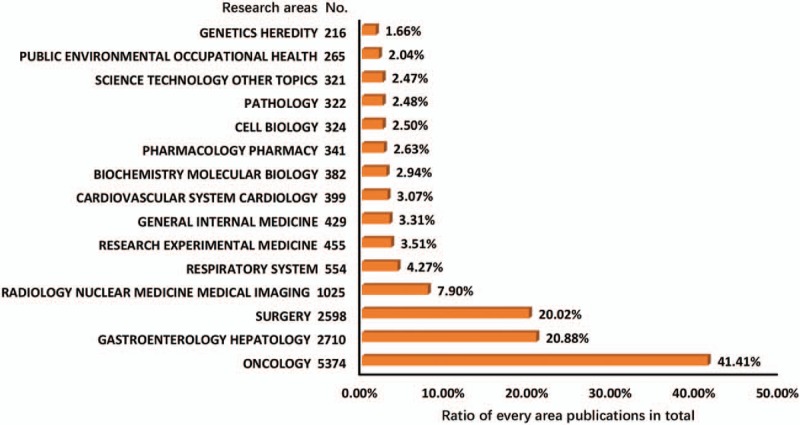
The top 15 research areas related to esophageal and esophagogastric junction cancer from 2007 to 2016.

The investigation of esophageal and EGJ cancer has occurred in 83 specific research areas (Supplemental Table 7). Here, we chose the top 15 research areas that were frequently featured in publications (Fig. 9). *Oncology* accounted for the largest proportion of the publications (41.41%), followed by *Gastroenterology Hepatology* (20.88%), *Surgery* (20.02%), and *Radiology Nuclear Medicine Medical Imaging* (7.90%).

### 3.7 Analysis of keywords

**Figure 10:**
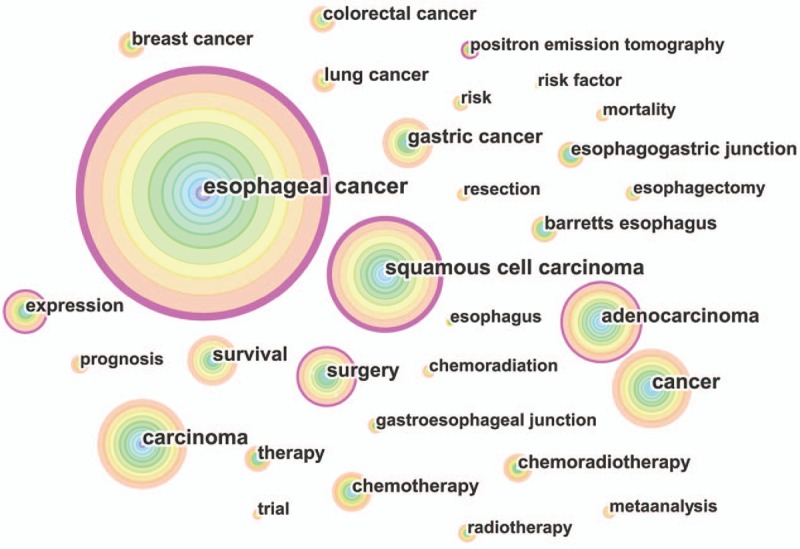
Keyword co-occurrence map of publications on esophageal and esophagogastric junction cancer from 2007 to 2016.

**Figure 11:**
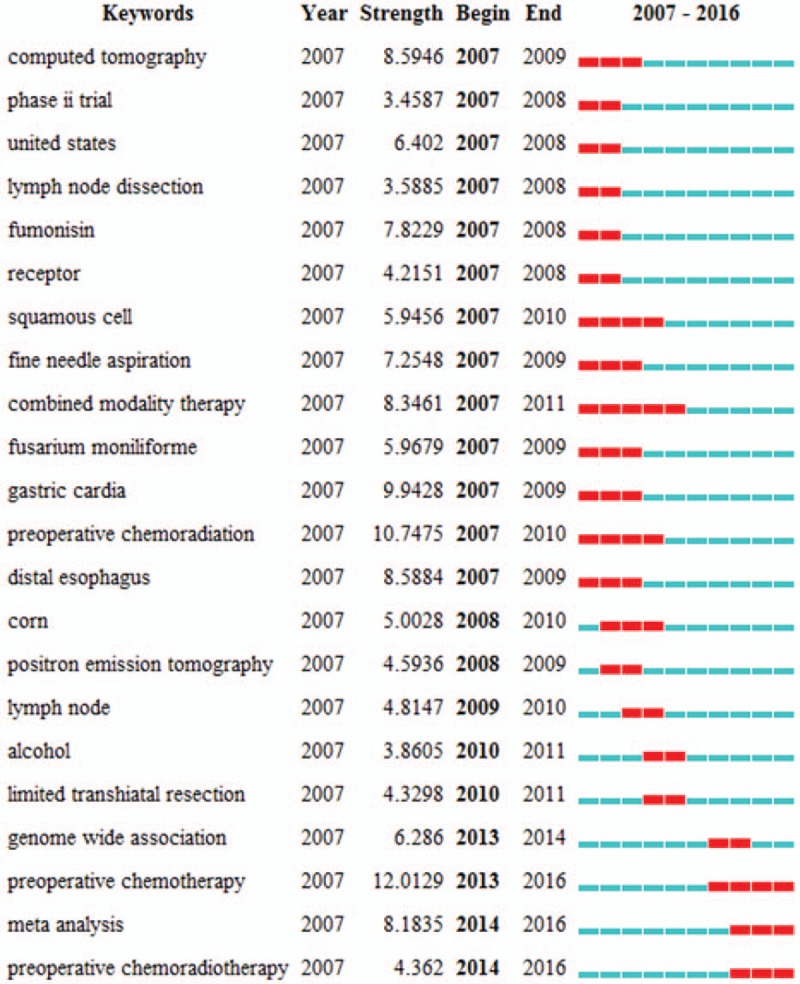
The keywords with the strongest citation bursts of publications on esophageal and esophagogastric junction cancer from 2007 to 2016.

Keywords that occurred in the 12,978 publications were extracted and analyzed with CiteSpace (Fig. 10) (Supplemental Fig. 4). The keywords with over 1000 usage count were identified as follows (Table 4): esophageal cancer (4674 counts), squamous cell carcinoma (2258 counts), carcinoma (1831 counts), cancer (1826 counts), adenocarcinoma (1714 counts), surgery (1311 counts), gastric cancer (1222 counts), survival (1219 counts), and chemotherapy (1016 counts). Keywords with the strongest citation bursts were also detected and analyzed with CiteSpace (Fig. 11). The keywords with the strongest citation bursts after 2010 are listed as follows: alcohol (2010–2011), limited transhiatal resection (2010–2011), genome-wide association (2013–2014), preoperative chemotherapy (2013–2016), meta-analysis (2014–2016), and preoperative chemoradiotherapy (2014–2016).

**Table 4:**
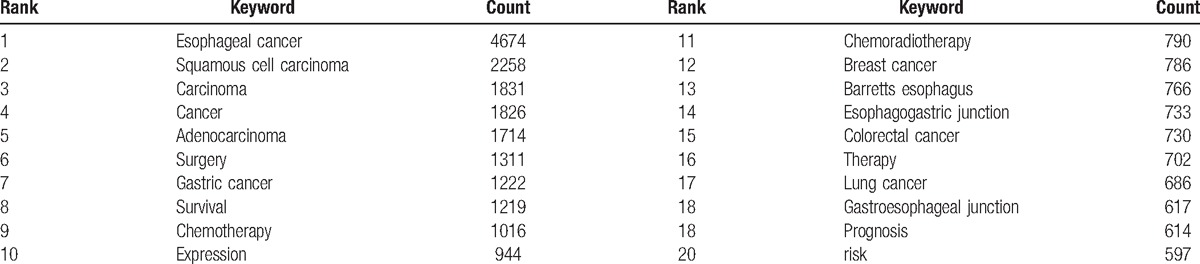
The top 20 keywords of publications on esophageal and esophagogastric junction cancer from 2007 to 2016.

## 4 Discussion

### 4.1 General data

In relation to the number of publications, in the first 4 years, the overall trend slowly increased from 877 articles in 2007 to 979 articles in 2010 (a slight drop in this year), maintaining a significant growth rate in the subsequent 3 years. The concentrated research of microRNAs expression in esophageal cancer^[5–8]^ might be the contributor to publication growth during that period (2011–2013). Besides, the increasing number of journals that indexed in the WoSCC database may have also contributed to the increase in the number of publications. With increase in the intensity of research, research period will also increase. This may account for the increase in the number of publications within those 3 years, but at a relatively slow rate.

According to the top 15 journals, 13.33% of the journals, including the *Journal of Clinical Oncology* (IF2015, 20.982) and *Gastroenterology* (IF2015, 18.187) had an IF greater than 10.000; 20.00% of the journals, including *Annals of Oncology* (IF2015, 9.269), *Gastrointestinal Endoscopy* (IF2015, 6.217), and *Cancer Research* (IF2015, 8.556), had an IF between 5.000 and 10.000; 33.33% of the journals, including *International Journal of Radiation Oncology Biology Physics* (IF2015, 4.495), *Annals of Surgical Oncology* (IF2015, 3.655), *PLoS One* (IF2015, 3.057), *Annals of Thoracic Surgery* (IF2015, 3.021), and *Journal of Gastroenterology and Hepatology* (IF2015, 3.322), had an IF between 3.000 and 5.000. Furthermore, the journals with high IF (greater than 3.000) contributed to 20.05% (IF >10.000, 5.39%; 10.000 >IF >5.000, 4.55%; 5.000 >IF >3.000, 10.11%) of the total number of publications. In summary, it was challenging to have papers related to esophageal and EGJ cancer, published in high IF journals.

The top 10 countries/regions (5 European countries, 1 American country, and 4 Asian countries/regions) who engaged in esophageal and EGJ cancer research, contributed to 12,070 publications, accounting for 93.00% of the total number of publications. China was the only developing country in the list, which indicated its significant progress in the life sciences over the past decade. The USA contributed to 3246 publications (about one-quarter of the total number of publications), reflecting its dominant position in esophageal and EGJ cancer research. The strongest collaborations by countries were identified between Romania and Slovenia, between Singapore and Malaysia, and between Tanzania and Kenya.

The top 10 institutions that were engaged in esophageal and EGJ research, contributed to 1961 publications, accounting for 15.11% of the total number of publications. In this list, the Chinese institutions accounted for nearly half of these publications. These institutions included the Chinese Academy of Medical Sciences Peking Union Medical College, Zhengzhou University, Sun Yat Sen University, and Shandong University. As China is one of the high-risk areas of esophageal cancer, especially in North-Central China (belonging to the “Esophageal Cancer Belt”),^[9–11]^ the number of Chinese institutions engaged in esophageal and EGJ research is expected.

### 4.2 Citation data

Each of the top 10 authors identified in this analysis contributed to at least 89 publications. Therefore, they were referred to as “prolific authors.” Unfortunately, none of these prolific authors was included in the list of the top 10, with regard to the annual co-citation counts, suggesting that prolific authors should consider not only the number of articles but also the quality of articles. For co-cited authors, the authors who had at least 700 co-citation counts, included Jemal A, Cunningham D, Enzinger PC, Siewert JR, and Parkin DM. Although none of these authors belonged to the prolific authors, they played a major role in esophageal and EGJ cancer research; particularly, Jemal A, who made crucial contributions to cancer statistics (including statistical data on esophageal cancer).^[12–15]^

For cited reference clusters, the timeline view map of co-citation clusters indicated that most clusters were concentrated in the period from 2010 to 2013. This result was consistent with the trend of annual publications. According to the results of the top 10 references with co-citation counts, Jemal A (2011) who published in *CA: A Cancer Journal for Clinicians* had the highest co-citation counts (586), followed by Van Hagen P (2012, 555 co-citation counts), Gebski V (2007, 317 co-citation counts), and Sjoquist KM (2011, 310 co-citation counts), who published in the *New England Journal of Medicine*, *Lancet Oncology*, and *Lancet Oncology* respectively. Additionally, *Lancet*, *International Journal of Cancer*, and *Journal of Clinical Oncology* also published some highly influential papers. These journals were fundamental for esophageal and EGJ research.

### 4.3 Further information on esophagogastric junction cancer

Of the total number of publications, there were 2459 publications on EGJ cancer. Over the past few decades, EGJ cancer has been a subject of controversy, both in diagnostic classification and management. This is mainly due to the difficulty in determining whether malignant tumors of EGJ are primary esophageal or gastric lesions. Until recently, EGJ cancer was considered as belonging to the esophagus.^[16]^

The most common histologic types of EGJ cancer are squamous cell carcinoma and adenocarcinoma. From available data collected by the SEER cancer registry program in the USA, the incidence of EGJ adenocarcinoma increased by nearly 2.5-fold, between 1973 and 1992, and maintained a stable rate in the last 2 decades.^[17]^ In addition to the 2 histologic types mentioned above, researchers have found the Epstein–Barr Virus (EBV) associated lymphoepithelioma-like carcinoma (LEC) and the non-EBV associated LEC in recent case reports.^[18,19]^ LEC is rarely diagnosed in the esophagus and the stomach, so its occurrence with EGJ is rare. Usually, EBV-induced LEC is not associated with microsatellite instability.^[20]^ However, in the case of a non-EBV-induced LEC, a total different histological pathway is presented which needs further studies to elucidate its origin.^[18]^

Regarding treatment, surgical resection with lymphadenectomy was the primary strategy for all resectable tumors of the EGJ. EGJ adenocarcinoma accounts for nearly 90% of EGJ cancer, and surgical resection is its initial management strategy.^[21]^ Generally, Siewert type I EGJ adenocarcinoma is treated by esophagectomy. However, the treatment choice for Siewert type II and III EGJ adenocarcinoma remains controversial. A recent study has found that Ivor-Lewis esophagogastrectomy, a new surgical approach, does not increase complication rates and perioperative mortality in Siewert type II EGJ adenocarcinoma, compared to the traditional left transthoracic approach.^[22]^ In addition, evidence suggests that spleen-preserving No.10 lymphadenectomy (SPL) may improve the prognosis of Siewert type III adenocarcinoma (tumor diameter ≥4 cm) of the EGJ, while for patients with Siewert type II or III adenocarcinoma (tumor diameter <4 cm), SPL may be omitted without reducing the survival rate.^[23]^ Although the above-mentioned surgical approaches have some limitations, they provide new methods for the treatment of EGJ cancer.

Except for surgical management, other therapeutic approach to EGJ cancer include chemotherapy, radiotherapy, chemoradiotherapy, and multimodal therapy.^[24]^ However, most of these therapies can only provide short-term benefits.^[25]^ With the significant increase in the understanding of the biology and molecular pathogenesis of EGJ cancer, target therapy has developed into a new therapeutic approach. According to the novel target therapies, antihuman epidermal growth factor receptor 2 trastuzumab, and antivascular endothelial growth factor receptor 2 ramucirumab have proven beneficial as first-line and second-line therapies, respectively.^[25,26]^ A subset analysis has identified that patients with epidermal growth factor receptor, fibroblast growth factor receptor 2, and met proto-oncogene genome abnormalities may benefit from matching target therapies.^[26]^ Nevertheless, it is extremely challenging to design traditional trials for such interpatient heterogeneity and infrequent aberrations. Therefore, more research is warranted to optimize the target therapies.

### 4.4 Research hotspots and frontiers

Keywords (concentrated expression of current research issues or concepts) provide a reasonable description for research hotspots, while burst words (emerging trends or abrupt changes) stand for research frontiers.^[27]^ According to the top 20 keywords of esophageal cancer, we inferred the top 3 research hotspots and listed them accordingly:

Esophageal squamous cell carcinoma (ESCC): ESCC is the most common histological type of esophageal cancer and is identified as the world's sixth leading cause of cancer death.^[28,29]^ This carcinoma has a significant geographic and ethnic distribution, especially in some Asian countries (e.g., China, Japan, and Iran).^[30,31]^ In high-risk areas, family clustering is observed in different populations, which implies that the risk factors are both genetic and environmental.^[32,33]^

Esophageal adenocarcinoma: The incidence of esophageal adenocarcinoma (EAC) is rapidly increasing in industrialized countries (e.g., Australia, USA, and Northern Europe) and is at the moment, the most prevalent histological type in these countries.^[34,35]^ EAC occurs after the normal squamous epithelium undergoes metaplasia, into a specialized columnar epithelium, which can eventually progress to subsequent malignancy.^[36]^ Furthermore, EAC has been associated with excessive alcohol intake and/or cigarette smoking.^[37–39]^

Esophageal surgery/esophagectomy: Surgery to resect some or most of the esophagus is “esophagectomy.” Usually, a small part of the stomach is also resected.^[40,41]^ It is worth mentioning that postoperative mortality after esophagectomy remains a major factor in the prognosis of esophageal cancer, which largely depends on the preoperative physiological state of patients.^[42,43]^ Moreover, esophagectomy is also the treatment choice for Siewert type I EGJ adenocarcinoma.^[44]^

The burst keywords captured by CiteSpace were identified as research frontiers over time. Here, the time interval was plotted on the blue line and the period of burst keyword was plotted on the red line, which indicated the beginning and end of the time interval of each burst.^[45]^ The top 3 research frontiers of esophageal and EGJ cancers were as follow.

Preoperative chemotherapy/chemoradiotherapy: The optimal management of esophageal and EGJ cancer remains a controversy. Despite this, there is a consensus that surgery alone is inadequate for patients with locally advanced esophageal or EGJ cancer.^[24,46]^ Many trials suggested that preoperative chemotherapy or chemoradiotherapy as a neoadjuvant therapy improves overall survival in operable esophageal or EGJ cancer.^[24,47,48]^ Furthermore, recent evidence found no difference between the 2 therapy options.^[49]^ Therefore, either approach was reasonable.

Meta-analysis: Many meta-analysis articles on esophageal and EGJ cancer were published from 2007 to 2016, including some high-quality ones.^[50–56]^

Genome-wide association study: The genome-wide association study (also called “GWAS”) is an examination, in which whole-gene variants in different individuals were examined, to assess the association of any variant with a trait.^[57]^ GWAS is concerned with the association between single-nucleotide polymorphisms and traits such as major human diseases. In esophageal and EGJ cancer research, GWAS has been mainly used to find a series of susceptible genes and loci ^[58–60]^ related to esophageal cancer and to provide researchers with new strategies for treatment, diagnosis, and prevention.

### 4.5 Strengths and limitations

As far as we are concerned, this is the first bibliometric analysis of the trend in esophageal and EGJ cancer research over the past decade. Data were retrieved and extracted from the SCI-E and SSCI journals, in WoSCC database. The data analysis was relatively objective and comprehensive. However, the majority of articles in the WoSCC database were written in English. Non-English articles involved were very few, to some degree, resulting in incomplete analysis. Therefore, future analysis can focus on non-English esophageal and EGJ cancer studies.

## 5 Conclusion

In conclusion, this study helps investigators master the trend of esophageal and EGJ cancer research. The top 3 journals that had the largest number of publications were *Journal of Clinical Oncology*, *Diseases of the Esophagus*, and *International Journal of Radiation Oncology Biology Physics*. The USA (3246 publications), China (2932 publications), and Japan (2267 publications) were the top 3 countries engaged in esophageal and EGJ cancer research. Strong research collaborations were observed between some neighboring countries. There were many Chinese institutions engaged in esophageal and EGJ cancer research, but significant collaborations among them were not noted. Jemal A, Van Hagen P, Cunningham D, and Enzinger PC may be good candidates for research collaboration in this field. ESCC and EAC are still the hotspots in this field. Neoadjuvant therapy, target therapy, and GWAS may be the frontiers of esophageal and EGJ cancer research in the next few years.

## Supplementary Material

Supplemental Digital Content
