# Supplementary material for: Trends in esophageal and esophagogastric junction cancer research from 2007 to 2016: A bibliometric analysis
Source: Medicine (Baltimore). 2017 May 19;96(20):e6924. doi: 10.1097/MD.0000000000006924 (PMC5440148; doi:10.1097/MD.0000000000006924)
Supplement: Supplemental Digital Content [file medi-96-e6924-s001.doc]

**Supplemental digital content**

**Supplemental Figure 1.** Figure that demonstrates co-cited authors that analyzed by CiteSpace.


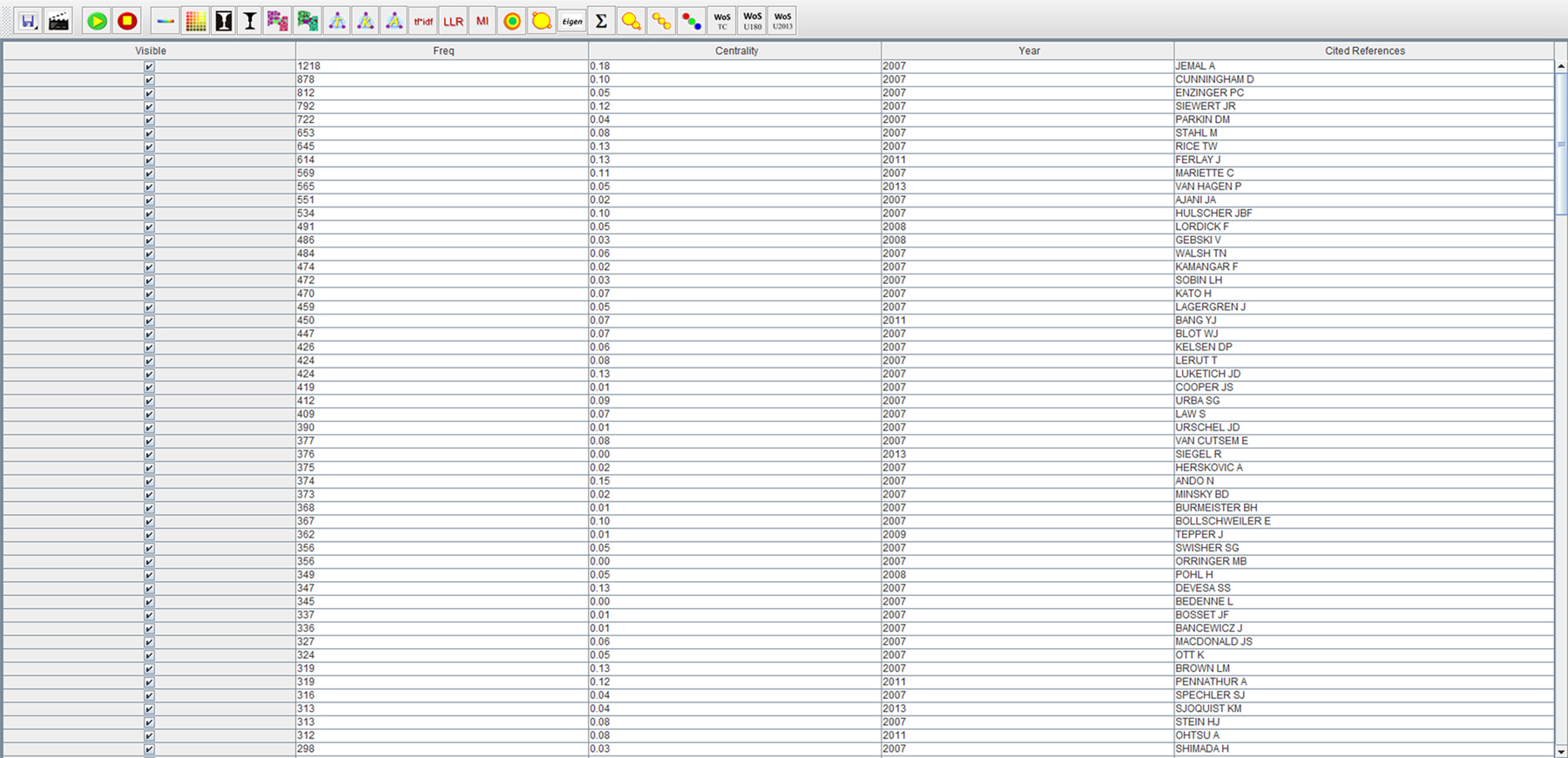


**Supplemental Figure 2.** Figure that demonstrates co-citation map of references that analyzed by CiteSpace.


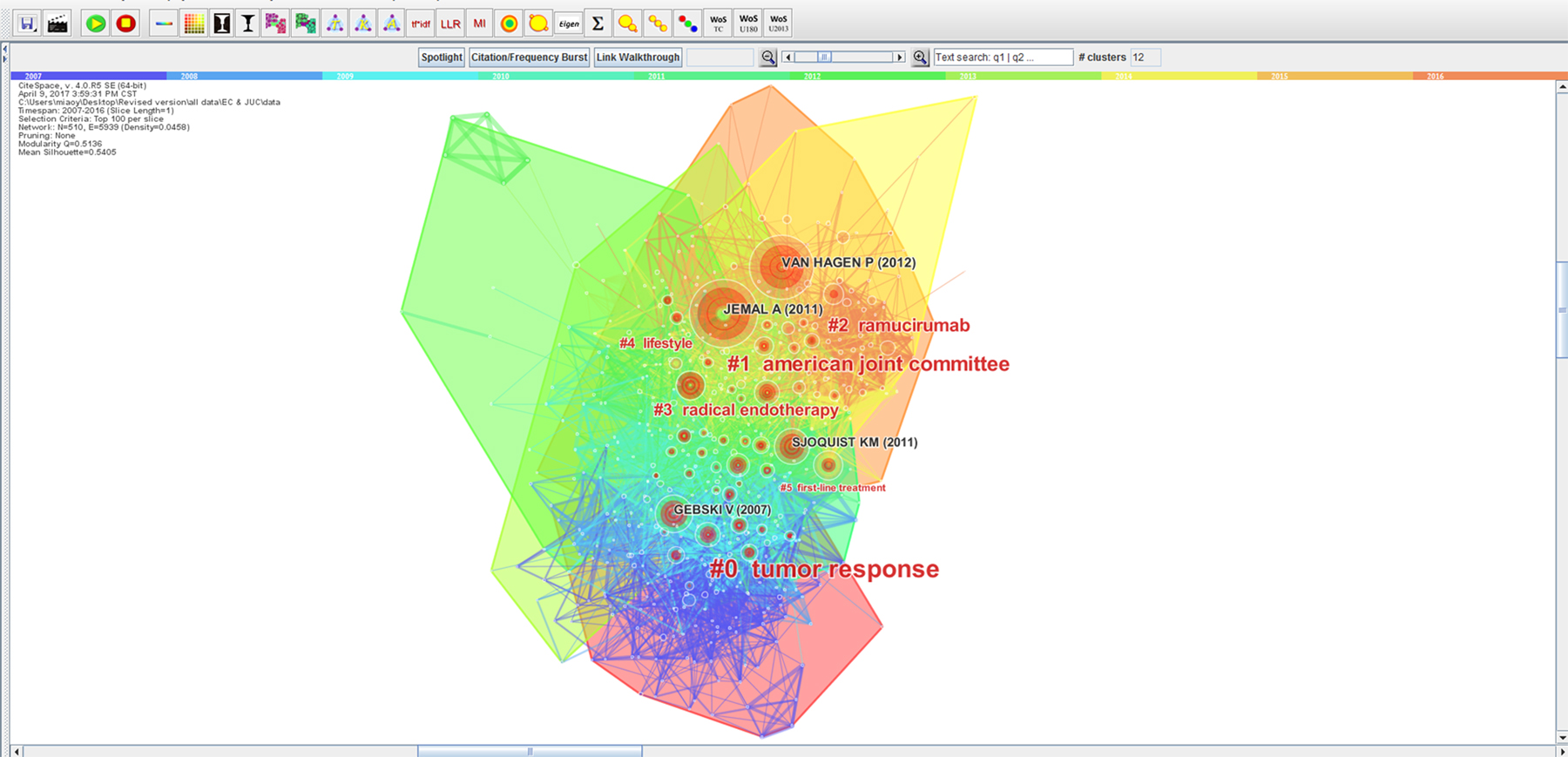


**Supplemental Figure 3.** Figure that demonstrates co-cited references that analyzed by CiteSpace.


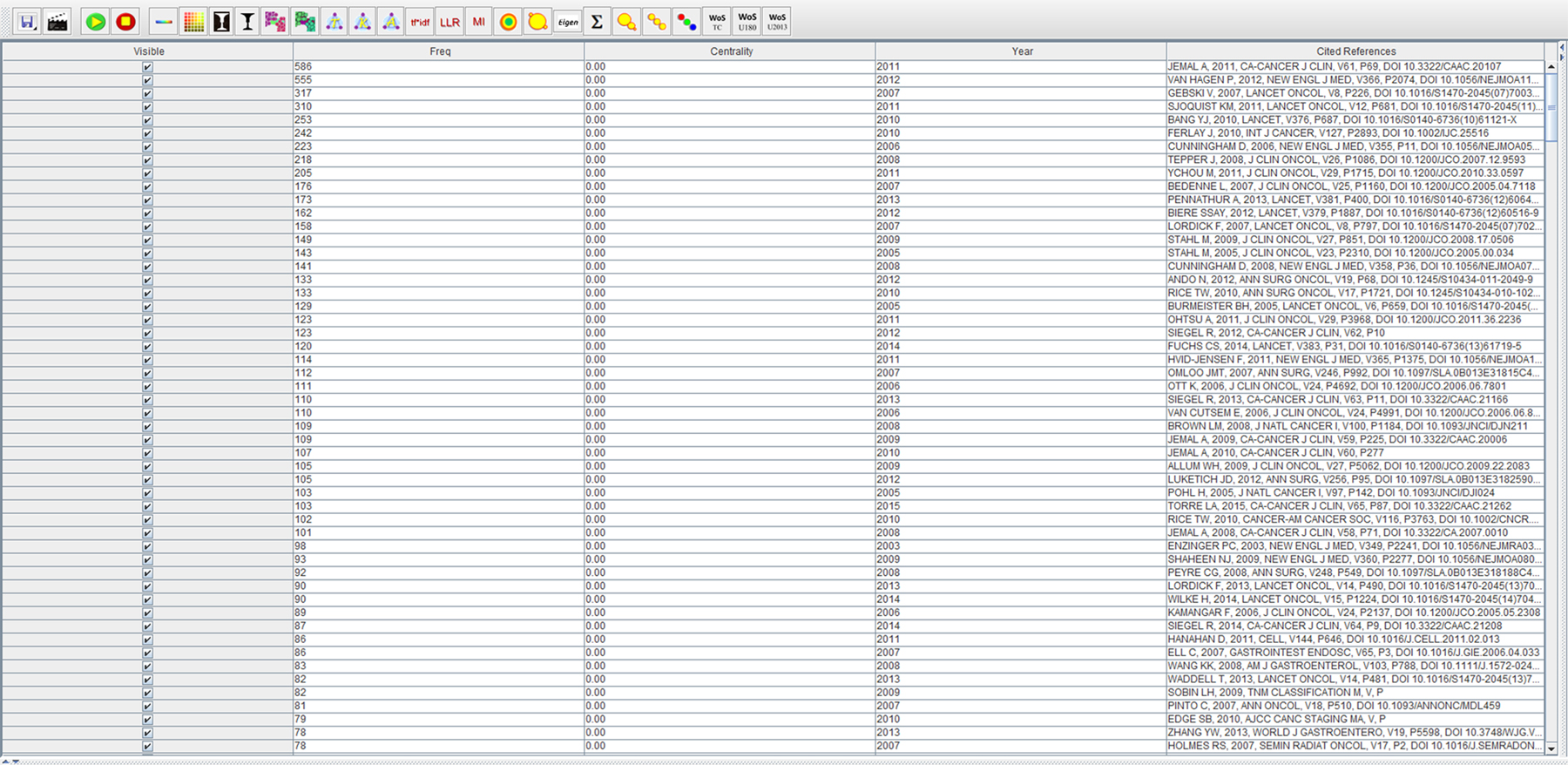


**Supplemental Figure 4.** Figure that demonstrates keywords that analyzed by CiteSpace.

**
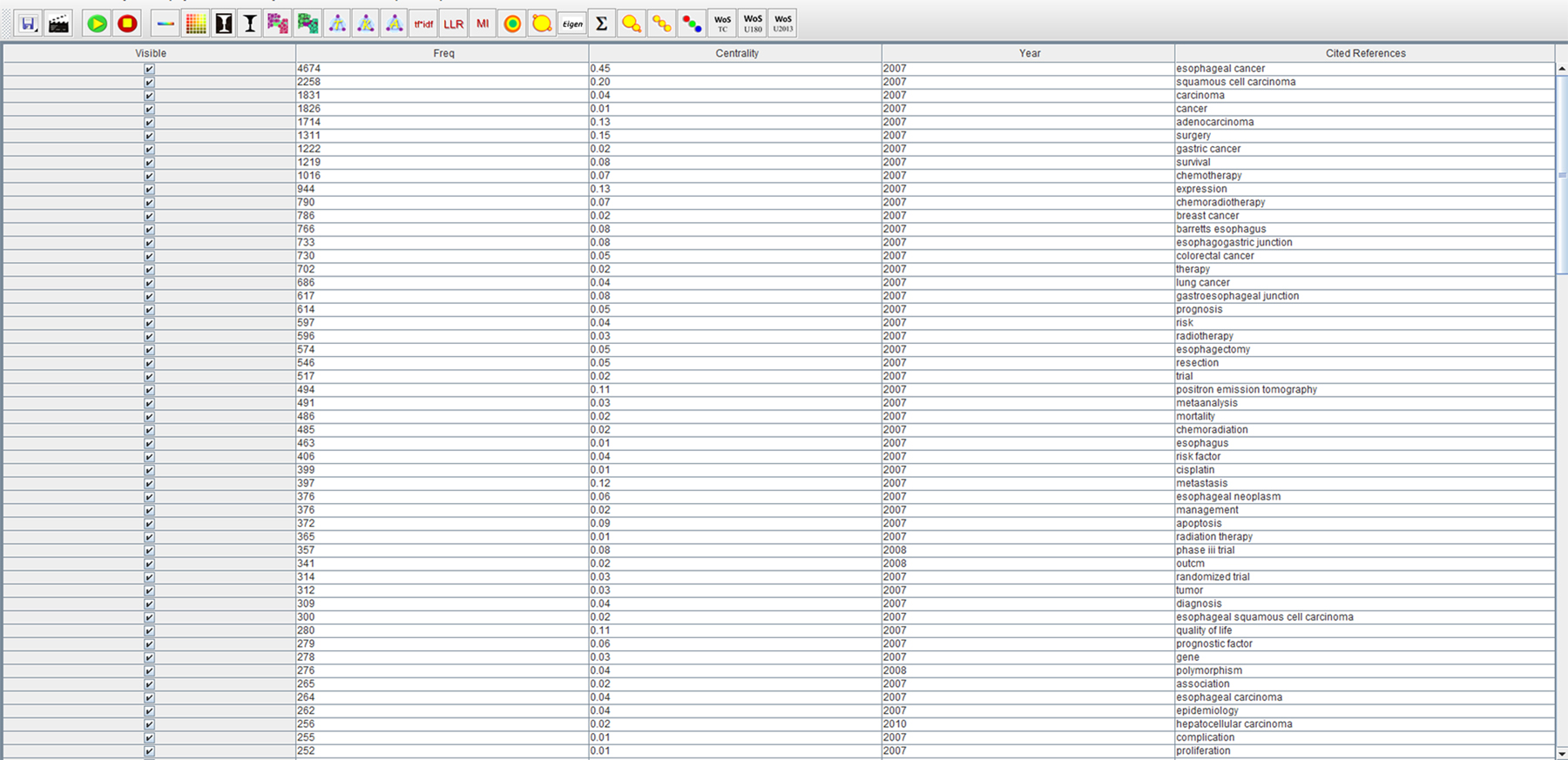
**

**Supplemental Table 1.** Table that demonstrates document types of publications extracted from the Web of Science ^TM^ Core Collection.

| Document Types | records | % of 12978 |
| --- | --- | --- |
| ARTICLE | 9427 | 72.638 |
| MEETING ABSTRACT | 1811 | 13.954 |
| REVIEW | 1112 | 8.568 |
| EDITORIAL MATERIAL | 400 | 3.082 |
| PROCEEDINGS PAPER | 351 | 2.705 |
| LETTER | 178 | 1.372 |
| CORRECTION | 39 | 0.301 |
| BOOK CHAPTER | 14 | 0.108 |
| NEWS ITEM | 11 | 0.085 |
| RETRACTED PUBLICATION | 3 | 0.023 |
| (0 Document Types {0} {1} value(s) outside display options.) | | |
| (0 records (0.000%){0} records{1} do not contain data in the field being analyzed.) | | |

**Supplemental Table 2.** Table that demonstrates language types of publications extracted from the Web of Science ^TM^ Core Collection.

| Languages | records | % of 12978 |
| --- | --- | --- |
| ENGLISH | 12674 | 97.658 |
| GERMAN | 183 | 1.41 |
| FRENCH | 56 | 0.431 |
| SPANISH | 35 | 0.27 |
| CHINESE | 6 | 0.046 |
| ROMANIAN | 5 | 0.039 |
| POLISH | 5 | 0.039 |
| PORTUGUESE | 4 | 0.031 |
| TURKISH | 2 | 0.015 |
| SERBIAN | 2 | 0.015 |
| (0 Languages {0} {1} value(s) outside display options.) | | |
| (0 records (0.000%){0} records{1} do not contain data in the field being analyzed.) | | |

**Supplemental Table 3.** Table that demonstrates journals contributed to publications on esophageal and esophagogastric junction cancer from the Web of Science ^TM^ Core Collection.

| Source Titles | records | % of 12978 |
| --- | --- | --- |
| JOURNAL OF CLINICAL ONCOLOGY | 465 | 3.583 |
| DISEASES OF THE ESOPHAGUS | 422 | 3.252 |
| INTERNATIONAL JOURNAL OF RADIATION ONCOLOGY BIOLOGY PHYSICS | 411 | 3.167 |
| ANNALS OF SURGICAL ONCOLOGY | 328 | 2.527 |
| WORLD JOURNAL OF GASTROENTEROLOGY | 263 | 2.027 |
| PLOS ONE | 235 | 1.811 |
| GASTROENTEROLOGY | 235 | 1.811 |
| ANNALS OF ONCOLOGY | 225 | 1.734 |
| ESOPHAGUS | 214 | 1.649 |
| GASTROINTESTINAL ENDOSCOPY | 212 | 1.634 |
| ASIAN PACIFIC JOURNAL OF CANCER PREVENTION | 187 | 1.441 |
| ANNALS OF THORACIC SURGERY | 187 | 1.441 |
| ANTICANCER RESEARCH | 156 | 1.202 |
| CANCER RESEARCH | 154 | 1.187 |
| JOURNAL OF GASTROENTEROLOGY AND HEPATOLOGY | 150 | 1.156 |
| INTERNATIONAL JOURNAL OF CANCER | 145 | 1.117 |
| JOURNAL OF GASTROINTESTINAL SURGERY | 141 | 1.086 |
| ANNALS OF SURGERY | 141 | 1.086 |
| SURGICAL ENDOSCOPY AND OTHER INTERVENTIONAL TECHNIQUES | 135 | 1.04 |
| AMERICAN JOURNAL OF GASTROENTEROLOGY | 131 | 1.009 |
| WORLD JOURNAL OF SURGERY | 125 | 0.963 |
| TUMOR BIOLOGY | 123 | 0.948 |
| JOURNAL OF SURGICAL ONCOLOGY | 122 | 0.94 |
| ONCOTARGET | 103 | 0.794 |
| ONCOLOGY REPORTS | 103 | 0.794 |
| SURGERY TODAY | 101 | 0.778 |
| ONCOLOGY LETTERS | 91 | 0.701 |
| BMC CANCER | 89 | 0.686 |
| HEPATO GASTROENTEROLOGY | 88 | 0.678 |
| CANCER | 88 | 0.678 |
| INTERNATIONAL JOURNAL OF CLINICAL AND EXPERIMENTAL MEDICINE | 82 | 0.632 |
| JOURNAL OF THORACIC ONCOLOGY | 77 | 0.593 |
| EUROPEAN JOURNAL OF CANCER | 77 | 0.593 |
| DIGESTIVE DISEASES AND SCIENCES | 77 | 0.593 |
| JOURNAL OF THORACIC AND CARDIOVASCULAR SURGERY | 75 | 0.578 |
| JOURNAL OF THORACIC DISEASE | 73 | 0.562 |
| ENDOSCOPY | 73 | 0.562 |
| RADIOTHERAPY AND ONCOLOGY | 65 | 0.501 |
| WORLD JOURNAL OF SURGICAL ONCOLOGY | 63 | 0.485 |
| JAPANESE JOURNAL OF CLINICAL ONCOLOGY | 58 | 0.447 |
| INTERNATIONAL JOURNAL OF ONCOLOGY | 58 | 0.447 |
| INTERNATIONAL JOURNAL OF CLINICAL AND EXPERIMENTAL PATHOLOGY | 58 | 0.447 |
| CLINICAL CANCER RESEARCH | 56 | 0.431 |
| MEDICAL ONCOLOGY | 55 | 0.424 |
| GASTRIC CANCER | 55 | 0.424 |
| JOURNAL OF THE AMERICAN COLLEGE OF SURGEONS | 54 | 0.416 |
| STRAHLENTHERAPIE UND ONKOLOGIE | 52 | 0.401 |
| EUROPEAN JOURNAL OF NUCLEAR MEDICINE AND MOLECULAR IMAGING | 51 | 0.393 |
| CHIRURG | 51 | 0.393 |
| DIGESTIVE ENDOSCOPY | 50 | 0.385 |
| CANCER EPIDEMIOLOGY BIOMARKERS PREVENTION | 49 | 0.378 |
| ONCOTARGETS AND THERAPY | 47 | 0.362 |
| MEDICINE | 47 | 0.362 |
| EJSO | 47 | 0.362 |
| CLINICAL GASTROENTEROLOGY AND HEPATOLOGY | 47 | 0.362 |
| CANCER LETTERS | 47 | 0.362 |
| CANCER SCIENCE | 46 | 0.354 |
| CANCER CHEMOTHERAPY AND PHARMACOLOGY | 42 | 0.324 |
| BRITISH JOURNAL OF CANCER | 42 | 0.324 |
| JOURNAL OF NUCLEAR MEDICINE | 40 | 0.308 |
| CANCER CAUSES CONTROL | 39 | 0.301 |
| BRITISH JOURNAL OF SURGERY | 38 | 0.293 |
| SCIENTIFIC REPORTS | 37 | 0.285 |
| ANNALS OF THE NEW YORK ACADEMY OF SCIENCES | 37 | 0.285 |
| THORACIC CANCER | 36 | 0.277 |
| MOLECULAR MEDICINE REPORTS | 35 | 0.27 |
| MOLECULAR BIOLOGY REPORTS | 35 | 0.27 |
| EUROPEAN JOURNAL OF CARDIO THORACIC SURGERY | 35 | 0.27 |
| CHINESE MEDICAL JOURNAL | 35 | 0.27 |
| CARCINOGENESIS | 35 | 0.27 |
| AMERICAN JOURNAL OF CLINICAL ONCOLOGY CANCER CLINICAL TRIALS | 35 | 0.27 |
| RADIATION ONCOLOGY | 34 | 0.262 |
| LANGENBECKS ARCHIVES OF SURGERY | 34 | 0.262 |
| JOURNAL OF SURGICAL RESEARCH | 34 | 0.262 |
| GENETICS AND MOLECULAR RESEARCH | 33 | 0.254 |
| CHINESE JOURNAL OF CANCER RESEARCH | 33 | 0.254 |
| ONKOLOGE | 32 | 0.247 |
| ONCOLOGY | 32 | 0.247 |
| INTERNATIONAL JOURNAL OF CLINICAL ONCOLOGY | 32 | 0.247 |
| EXPERIMENTAL AND THERAPEUTIC MEDICINE | 32 | 0.247 |
| ARCHIVES OF IRANIAN MEDICINE | 32 | 0.247 |
| TUMORI | 30 | 0.231 |
| DIGESTIVE SURGERY | 30 | 0.231 |
| CLINICAL NUCLEAR MEDICINE | 30 | 0.231 |
| SURGERY | 29 | 0.223 |
| JOURNAL OF CANCER RESEARCH AND CLINICAL ONCOLOGY | 29 | 0.223 |
| GASTROENTEROLOGY CLINICS OF NORTH AMERICA | 29 | 0.223 |
| MEDICAL PHYSICS | 28 | 0.216 |
| JOURNAL OF CANCER RESEARCH AND THERAPEUTICS | 28 | 0.216 |
| SURGICAL LAPAROSCOPY ENDOSCOPY PERCUTANEOUS TECHNIQUES | 27 | 0.208 |
| SCANDINAVIAN JOURNAL OF GASTROENTEROLOGY | 27 | 0.208 |
| ONKOLOGIE | 27 | 0.208 |
| NUTRITION AND CANCER AN INTERNATIONAL JOURNAL | 27 | 0.208 |
| AMERICAN JOURNAL OF SURGICAL PATHOLOGY | 27 | 0.208 |
| AMERICAN JOURNAL OF SURGERY | 27 | 0.208 |
| AMERICAN JOURNAL OF CANCER RESEARCH | 27 | 0.208 |
| MEDICAL SCIENCE MONITOR | 26 | 0.2 |
| EUROPEAN SURGERY ACTA CHIRURGICA AUSTRIACA | 26 | 0.2 |
| CANCER EPIDEMIOLOGY | 26 | 0.2 |
| ANNALS OF THORACIC AND CARDIOVASCULAR SURGERY | 26 | 0.2 |
| ONCOLOGIST | 25 | 0.193 |
| EXPERT REVIEW OF ANTICANCER THERAPY | 25 | 0.193 |
| DIGESTION | 25 | 0.193 |
| THORACIC SURGERY CLINICS | 24 | 0.185 |
| CANCER BIOLOGY THERAPY | 24 | 0.185 |
| ORAL ONCOLOGY | 23 | 0.177 |
| JOURNAL OF TRANSLATIONAL MEDICINE | 23 | 0.177 |
| JOURNAL OF LAPAROENDOSCOPIC ADVANCED SURGICAL TECHNIQUES | 23 | 0.177 |
| JOURNAL OF EXPERIMENTAL CLINICAL CANCER RESEARCH | 23 | 0.177 |
| JOURNAL OF DIGESTIVE DISEASES | 23 | 0.177 |
| EJC SUPPLEMENTS | 23 | 0.177 |
| CANCER INVESTIGATION | 23 | 0.177 |
| ACTA ONCOLOGICA | 23 | 0.177 |
| JOURNAL OF CLINICAL GASTROENTEROLOGY | 22 | 0.17 |
| INTERNATIONAL JOURNAL OF SURGERY | 22 | 0.17 |
| EXPERT REVIEW OF GASTROENTEROLOGY HEPATOLOGY | 22 | 0.17 |
| BMC GASTROENTEROLOGY | 22 | 0.17 |
| SUPPORTIVE CARE IN CANCER | 21 | 0.162 |
| JOURNAL OF THE NATIONAL COMPREHENSIVE CANCER NETWORK | 21 | 0.162 |
| HUMAN PATHOLOGY | 21 | 0.162 |
| EUROPEAN JOURNAL OF GASTROENTEROLOGY HEPATOLOGY | 21 | 0.162 |
| AMERICAN SURGEON | 21 | 0.162 |
| ZEITSCHRIFT FUR GASTROENTEROLOGIE | 20 | 0.154 |
| ONCOGENE | 20 | 0.154 |
| MOLECULAR CARCINOGENESIS | 20 | 0.154 |
| JOURNAL OF RADIATION RESEARCH | 20 | 0.154 |
| SEMINARS IN RADIATION ONCOLOGY | 19 | 0.146 |
| INTERNATIONAL JOURNAL OF MOLECULAR MEDICINE | 19 | 0.146 |
| GASTROENTEROLOGY RESEARCH AND PRACTICE | 19 | 0.146 |
| BEST PRACTICE RESEARCH IN CLINICAL GASTROENTEROLOGY | 19 | 0.146 |
| NEW ENGLAND JOURNAL OF MEDICINE | 18 | 0.139 |
| MODERN PATHOLOGY | 18 | 0.139 |
| INVESTIGATIONAL NEW DRUGS | 18 | 0.139 |
| EUROPEAN JOURNAL OF CANCER PREVENTION | 18 | 0.139 |
| CURRENT OPINION IN GASTROENTEROLOGY | 18 | 0.139 |
| CLINICAL TRANSLATIONAL ONCOLOGY | 18 | 0.139 |
| ARCHIVES OF SURGERY | 18 | 0.139 |
| FOOD ADDITIVES AND CONTAMINANTS PART A CHEMISTRY ANALYSIS CONTROL EXPOSURE RISK ASSESSMENT | 17 | 0.131 |
| DIGESTIVE AND LIVER DISEASE | 17 | 0.131 |
| COCHRANE DATABASE OF SYSTEMATIC REVIEWS | 17 | 0.131 |
| AMERICAN JOURNAL OF EPIDEMIOLOGY | 17 | 0.131 |
| 12TH OESO WORLD CONFERENCE CANCERS OF THE ESOPHAGUS | 17 | 0.131 |
| ZENTRALBLATT FUR CHIRURGIE | 16 | 0.123 |
| SURGICAL CLINICS OF NORTH AMERICA | 16 | 0.123 |
| QUALITY OF LIFE RESEARCH | 16 | 0.123 |
| JOURNAL OF CANCER | 16 | 0.123 |
| CANCER RADIOTHERAPIE | 16 | 0.123 |
| CANCER BIOMARKERS | 16 | 0.123 |
| JOURNAL OF GASTROENTEROLOGY | 15 | 0.116 |
| INTERNAL MEDICINE | 15 | 0.116 |
| GUT AND LIVER | 15 | 0.116 |
| FUTURE ONCOLOGY | 15 | 0.116 |
| EUROPEAN REVIEW FOR MEDICAL AND PHARMACOLOGICAL SCIENCES | 15 | 0.116 |
| CRITICAL REVIEWS IN ONCOLOGY HEMATOLOGY | 15 | 0.116 |
| CANCER PREVENTION RESEARCH | 15 | 0.116 |
| BIOCHEMICAL AND BIOPHYSICAL RESEARCH COMMUNICATIONS | 15 | 0.116 |
| NUCLEAR MEDICINE COMMUNICATIONS | 14 | 0.108 |
| MINERVA CHIRURGICA | 14 | 0.108 |
| INTERNATIONAL SURGERY | 14 | 0.108 |
| INTERNATIONAL JOURNAL OF MOLECULAR SCIENCES | 14 | 0.108 |
| INDIAN JOURNAL OF CANCER | 14 | 0.108 |
| HEAD AND NECK JOURNAL FOR THE SCIENCES AND SPECIALTIES OF THE HEAD AND NECK | 14 | 0.108 |
| FOOD AND CHEMICAL TOXICOLOGY | 14 | 0.108 |
| EUROPEAN SURGICAL RESEARCH | 14 | 0.108 |
| EUROPEAN RADIOLOGY | 14 | 0.108 |
| CURRENT ONCOLOGY | 14 | 0.108 |
| CELLULAR PHYSIOLOGY AND BIOCHEMISTRY | 14 | 0.108 |
| CANCER MEDICINE | 14 | 0.108 |
| ASIA PACIFIC JOURNAL OF CLINICAL ONCOLOGY | 14 | 0.108 |
| ARCHIVES OF PATHOLOGY LABORATORY MEDICINE | 14 | 0.108 |
| ABDOMINAL IMAGING | 14 | 0.108 |
| TRANSLATIONAL CANCER RESEARCH | 13 | 0.1 |
| PHARMACOEPIDEMIOLOGY AND DRUG SAFETY | 13 | 0.1 |
| PATHOLOGY INTERNATIONAL | 13 | 0.1 |
| ONCOLOGY RESEARCH AND TREATMENT | 13 | 0.1 |
| MOLECULAR CANCER THERAPEUTICS | 13 | 0.1 |
| JOURNAL OF TOXICOLOGY AND ENVIRONMENTAL HEALTH PART A CURRENT ISSUES | 13 | 0.1 |
| JOURNAL OF APPLIED CLINICAL MEDICAL PHYSICS | 13 | 0.1 |
| BIOMARKERS | 13 | 0.1 |
| AMERICAN JOURNAL OF ROENTGENOLOGY | 13 | 0.1 |
| SURGICAL ONCOLOGY CLINICS OF NORTH AMERICA | 12 | 0.092 |
| JOURNAL OF BUON | 12 | 0.092 |
| GUT | 12 | 0.092 |
| GENETIC TESTING AND MOLECULAR BIOMARKERS | 12 | 0.092 |
| GENE | 12 | 0.092 |
| BIOMEDICINE PHARMACOTHERAPY | 12 | 0.092 |
| APPLIED IMMUNOHISTOCHEMISTRY MOLECULAR MORPHOLOGY | 12 | 0.092 |
| ANNALS OF NUCLEAR MEDICINE | 12 | 0.092 |
| VIRCHOWS ARCHIV | 11 | 0.085 |
| TURK GOGUS KALP DAMAR CERRAHISI DERGISI TURKISH JOURNAL OF THORACIC AND CARDIOVASCULAR SURGERY | 11 | 0.085 |
| REVISTA ESPANOLA DE ENFERMEDADES DIGESTIVAS | 11 | 0.085 |
| RADIOLOGY | 11 | 0.085 |
| PATHOLOGY ONCOLOGY RESEARCH | 11 | 0.085 |
| MOLECULAR CANCER | 11 | 0.085 |
| LANCET ONCOLOGY | 11 | 0.085 |
| JOURNAL OF THE CHINESE MEDICAL ASSOCIATION | 11 | 0.085 |
| JOURNAL OF CARDIOTHORACIC SURGERY | 11 | 0.085 |
| INTERACTIVE CARDIOVASCULAR AND THORACIC SURGERY | 11 | 0.085 |
| HISTOPATHOLOGY | 11 | 0.085 |
| EUROPEAN JOURNAL OF RADIOLOGY | 11 | 0.085 |
| DISEASE MARKERS | 11 | 0.085 |
| CURRENT TREATMENT OPTIONS IN ONCOLOGY | 11 | 0.085 |
| CLINICAL EXPERIMENTAL METASTASIS | 11 | 0.085 |
| CHINESE JOURNAL OF CANCER | 11 | 0.085 |
| CANCER TREATMENT REVIEWS | 11 | 0.085 |
| CANCER CONTROL | 11 | 0.085 |
| BIOMED RESEARCH INTERNATIONAL | 11 | 0.085 |
| VISZERALMEDIZIN | 10 | 0.077 |
| THORACIC AND CARDIOVASCULAR SURGEON | 10 | 0.077 |
| SURGICAL ONCOLOGY OXFORD | 10 | 0.077 |
| SAUDI MEDICAL JOURNAL | 10 | 0.077 |
| PHARMACOGENOMICS | 10 | 0.077 |
| PATHOLOGY RESEARCH AND PRACTICE | 10 | 0.077 |
| ONCOLOGY NEW YORK | 10 | 0.077 |
| ONCOLOGIE | 10 | 0.077 |
| JOURNAL OF GASTROINTESTINAL AND LIVER DISEASES | 10 | 0.077 |
| JOURNAL OF AGRICULTURAL AND FOOD CHEMISTRY | 10 | 0.077 |
| EUROPEAN ARCHIVES OF OTO RHINO LARYNGOLOGY | 10 | 0.077 |
| CURRENT OPINION IN ONCOLOGY | 10 | 0.077 |
| AMERICAN JOURNAL OF TRANSLATIONAL RESEARCH | 10 | 0.077 |
| PSYCHO ONCOLOGY | 9 | 0.069 |
| JOURNAL OF THE NATIONAL CANCER INSTITUTE | 9 | 0.069 |
| JOURNAL OF KOREAN MEDICAL SCIENCE | 9 | 0.069 |
| JAPANESE JOURNAL OF RADIOLOGY | 9 | 0.069 |
| INDIAN JOURNAL OF SURGERY | 9 | 0.069 |
| FOOD CONTROL | 9 | 0.069 |
| EXPERT OPINION ON BIOLOGICAL THERAPY | 9 | 0.069 |
| CURRENT PHARMACEUTICAL DESIGN | 9 | 0.069 |
| CURRENT ONCOLOGY REPORTS | 9 | 0.069 |
| CIRUGIA ESPANOLA | 9 | 0.069 |
| BARRETT S ESOPHAGUS THE 10TH OESO WORLD CONGRESS PROCEEDINGS | 9 | 0.069 |
| ANTI CANCER DRUGS | 9 | 0.069 |
| ALCOHOLISM CLINICAL AND EXPERIMENTAL RESEARCH | 9 | 0.069 |
| THERAPEUTICS AND CLINICAL RISK MANAGEMENT | 8 | 0.062 |
| ONCOIMMUNOLOGY | 8 | 0.062 |
| NEOPLASMA | 8 | 0.062 |
| NATURE REVIEWS CLINICAL ONCOLOGY | 8 | 0.062 |
| MYCOPATHOLOGIA | 8 | 0.062 |
| MEDICAL HYPOTHESES | 8 | 0.062 |
| LABORATORY INVESTIGATION | 8 | 0.062 |
| JOURNAL OF VASCULAR AND INTERVENTIONAL RADIOLOGY | 8 | 0.062 |
| JOURNAL OF CLINICAL PATHOLOGY | 8 | 0.062 |
| JNCI JOURNAL OF THE NATIONAL CANCER INSTITUTE | 8 | 0.062 |
| INTERNATIONAL JOURNAL OF COLORECTAL DISEASE | 8 | 0.062 |
| DIGESTIVE DISEASES | 8 | 0.062 |
| CLINICAL COLORECTAL CANCER | 8 | 0.062 |
| CHIRURGIA | 8 | 0.062 |
| CANCER IMAGING | 8 | 0.062 |
| BRACHYTHERAPY | 8 | 0.062 |
| ANNALS OF NUTRITION AND METABOLISM | 8 | 0.062 |
| AMERICAN JOURNAL OF CLINICAL NUTRITION | 8 | 0.062 |
| ACTA CIRURGICA BRASILEIRA | 8 | 0.062 |
| YONSEI MEDICAL JOURNAL | 7 | 0.054 |
| VIDEOSURGERY AND OTHER MINIINVASIVE TECHNIQUES | 7 | 0.054 |
| UNITED EUROPEAN GASTROENTEROLOGY JOURNAL | 7 | 0.054 |
| TOXICOLOGY AND APPLIED PHARMACOLOGY | 7 | 0.054 |
| SCANDINAVIAN JOURNAL OF SURGERY | 7 | 0.054 |
| SAUDI JOURNAL OF GASTROENTEROLOGY | 7 | 0.054 |
| RADIATION RESEARCH | 7 | 0.054 |
| PHOTODIAGNOSIS AND PHOTODYNAMIC THERAPY | 7 | 0.054 |
| PAKISTAN JOURNAL OF MEDICAL SCIENCES | 7 | 0.054 |
| ONCOLOGY RESEARCH | 7 | 0.054 |
| OBESITY SURGERY | 7 | 0.054 |
| NUTRIENTS | 7 | 0.054 |
| NATURE REVIEWS GASTROENTEROLOGY HEPATOLOGY | 7 | 0.054 |
| NATURE CLINICAL PRACTICE GASTROENTEROLOGY HEPATOLOGY | 7 | 0.054 |
| LUNG CANCER | 7 | 0.054 |
| JOURNAL OF THE SCIENCE OF FOOD AND AGRICULTURE | 7 | 0.054 |
| JOURNAL OF CELLULAR BIOCHEMISTRY | 7 | 0.054 |
| JOURNAL OF BIOMEDICAL OPTICS | 7 | 0.054 |
| JAMA SURGERY | 7 | 0.054 |
| JAMA JOURNAL OF THE AMERICAN MEDICAL ASSOCIATION | 7 | 0.054 |
| IRANIAN JOURNAL OF PUBLIC HEALTH | 7 | 0.054 |
| INTERNATIONAL JOURNAL OF BIOLOGICAL MARKERS | 7 | 0.054 |
| HELICOBACTER | 7 | 0.054 |
| FOLIA HISTOCHEMICA ET CYTOBIOLOGICA | 7 | 0.054 |
| EXPERT OPINION ON PHARMACOTHERAPY | 7 | 0.054 |
| DEUTSCHE MEDIZINISCHE WOCHENSCHRIFT | 7 | 0.054 |
| CURRENT CANCER DRUG TARGETS | 7 | 0.054 |
| CLINICAL ONCOLOGY | 7 | 0.054 |
| CLINICAL NUTRITION | 7 | 0.054 |
| CHIRURGISCHE GASTROENTEROLOGIE | 7 | 0.054 |
| CELLULAR ONCOLOGY | 7 | 0.054 |
| BULLETIN DU CANCER | 7 | 0.054 |
| BMC SURGERY | 7 | 0.054 |
| AURIS NASUS LARYNX | 7 | 0.054 |
| ARCHIVES OF MEDICAL RESEARCH | 7 | 0.054 |
| ACTA GASTRO ENTEROLOGICA BELGICA | 7 | 0.054 |
| ACTA ENDOSCOPICA | 7 | 0.054 |
| TURKISH JOURNAL OF MEDICAL SCIENCES | 6 | 0.046 |
| TRIALS | 6 | 0.046 |
| TOXICOLOGY LETTERS | 6 | 0.046 |
| TOHOKU JOURNAL OF EXPERIMENTAL MEDICINE | 6 | 0.046 |
| RADIOLOGY AND ONCOLOGY | 6 | 0.046 |
| RADIOGRAPHICS | 6 | 0.046 |
| NUTRITION | 6 | 0.046 |
| MOLECULAR NUTRITION FOOD RESEARCH | 6 | 0.046 |
| MOLECULAR CANCER RESEARCH | 6 | 0.046 |
| MOLECULAR AND CELLULAR BIOCHEMISTRY | 6 | 0.046 |
| MEDICAL DOSIMETRY | 6 | 0.046 |
| KOREAN JOURNAL OF INTERNAL MEDICINE | 6 | 0.046 |
| JOURNAL OF THE FORMOSAN MEDICAL ASSOCIATION | 6 | 0.046 |
| JOURNAL OF NUTRITION | 6 | 0.046 |
| JOURNAL OF HUMAN GENETICS | 6 | 0.046 |
| INTERNATIONAL JOURNAL OF NANOMEDICINE | 6 | 0.046 |
| GASTROENTEROLOGIE CLINIQUE ET BIOLOGIQUE | 6 | 0.046 |
| FASEB JOURNAL | 6 | 0.046 |
| EXPERT REVIEW OF MEDICAL DEVICES | 6 | 0.046 |
| EXPERT OPINION ON INVESTIGATIONAL DRUGS | 6 | 0.046 |
| EXPERIMENTAL AND MOLECULAR PATHOLOGY | 6 | 0.046 |
| EPIGENETICS | 6 | 0.046 |
| DRUGS | 6 | 0.046 |
| DIAGNOSTIC PATHOLOGY | 6 | 0.046 |
| CLINICS AND RESEARCH IN HEPATOLOGY AND GASTROENTEROLOGY | 6 | 0.046 |
| CELL CYCLE | 6 | 0.046 |
| CELL BIOCHEMISTRY AND BIOPHYSICS | 6 | 0.046 |
| CANCER RESEARCH AND TREATMENT | 6 | 0.046 |
| CANCER NURSING | 6 | 0.046 |
| CANCER GENETICS AND CYTOGENETICS | 6 | 0.046 |
| CANCER GENE THERAPY | 6 | 0.046 |
| BMJ OPEN | 6 | 0.046 |
| ANTI CANCER AGENTS IN MEDICINAL CHEMISTRY | 6 | 0.046 |
| ANNALS OF THE ROYAL COLLEGE OF SURGEONS OF ENGLAND | 6 | 0.046 |
| ADVANCES IN CLINICAL AND EXPERIMENTAL MEDICINE | 6 | 0.046 |
| ACTA PHARMACOLOGICA SINICA | 6 | 0.046 |
| WORLD MYCOTOXIN JOURNAL | 5 | 0.039 |
| TURKIYE KLINIKLERI TIP BILIMLERI DERGISI | 5 | 0.039 |
| TECHNOLOGY IN CANCER RESEARCH TREATMENT | 5 | 0.039 |
| SURGICAL INNOVATION | 5 | 0.039 |
| STEM CELLS AND DEVELOPMENT | 5 | 0.039 |
| SPRINGERPLUS | 5 | 0.039 |
| SCIENCE OF THE TOTAL ENVIRONMENT | 5 | 0.039 |
| RADIOLOGE | 5 | 0.039 |
| PROCEEDINGS OF THE NATIONAL ACADEMY OF SCIENCES OF THE UNITED STATES OF AMERICA | 5 | 0.039 |
| PHYSICS IN MEDICINE AND BIOLOGY | 5 | 0.039 |
| PHARMACOGENOMICS JOURNAL | 5 | 0.039 |
| PAKISTAN JOURNAL OF PHARMACEUTICAL SCIENCES | 5 | 0.039 |
| OTOLARYNGOLOGY HEAD AND NECK SURGERY | 5 | 0.039 |
| NUTRITION CLINIQUE ET METABOLISME | 5 | 0.039 |
| NUTRICION HOSPITALARIA | 5 | 0.039 |
| MEDICINA CLINICA | 5 | 0.039 |
| MAYO CLINIC PROCEEDINGS | 5 | 0.039 |
| LIFE SCIENCE JOURNAL ACTA ZHENGZHOU UNIVERSITY OVERSEAS EDITION | 5 | 0.039 |
| LASERS IN MEDICAL SCIENCE | 5 | 0.039 |
| KARDIOCHIRURGIA I TORAKOCHIRURGIA POLSKA | 5 | 0.039 |
| JSLS JOURNAL OF THE SOCIETY OF LAPAROENDOSCOPIC SURGEONS | 5 | 0.039 |
| JOURNAL OF VISCERAL SURGERY | 5 | 0.039 |
| JOURNAL OF MOLECULAR MEDICINE JMM | 5 | 0.039 |
| JOURNAL OF INVESTIGATIVE MEDICINE | 5 | 0.039 |
| JOURNAL OF IMMUNOTHERAPY | 5 | 0.039 |
| JOURNAL OF FOOD PROTECTION | 5 | 0.039 |
| JOURNAL OF COMPUTER ASSISTED TOMOGRAPHY | 5 | 0.039 |
| JOURNAL OF CARDIOTHORACIC AND VASCULAR ANESTHESIA | 5 | 0.039 |
| JCPSP JOURNAL OF THE COLLEGE OF PHYSICIANS AND SURGEONS PAKISTAN | 5 | 0.039 |
| IRANIAN RED CRESCENT MEDICAL JOURNAL | 5 | 0.039 |
| HISTOLOGY AND HISTOPATHOLOGY | 5 | 0.039 |
| GENES CHROMOSOMES CANCER | 5 | 0.039 |
| GASTROENTEROLOGY NURSING | 5 | 0.039 |
| FOOD CHEMISTRY | 5 | 0.039 |
| FAMILIAL CANCER | 5 | 0.039 |
| EXPERT OPINION ON THERAPEUTIC TARGETS | 5 | 0.039 |
| EXPERT OPINION ON EMERGING DRUGS | 5 | 0.039 |
| EXPERIMENTAL CELL RESEARCH | 5 | 0.039 |
| EUROPEAN JOURNAL OF MEDICAL RESEARCH | 5 | 0.039 |
| EUROPEAN JOURNAL OF EPIDEMIOLOGY | 5 | 0.039 |
| DNA AND CELL BIOLOGY | 5 | 0.039 |
| CURRENT PROBLEMS IN SURGERY | 5 | 0.039 |
| CLINICAL BIOCHEMISTRY | 5 | 0.039 |
| CLINICA CHIMICA ACTA | 5 | 0.039 |
| CHEST | 5 | 0.039 |
| CARDIOVASCULAR AND INTERVENTIONAL RADIOLOGY | 5 | 0.039 |
| CANCER DETECTION AND PREVENTION | 5 | 0.039 |
| CANCER BIOTHERAPY AND RADIOPHARMACEUTICALS | 5 | 0.039 |
| CA A CANCER JOURNAL FOR CLINICIANS | 5 | 0.039 |
| BRITISH JOURNAL OF NUTRITION | 5 | 0.039 |
| BREAST CANCER RESEARCH AND TREATMENT | 5 | 0.039 |
| APMIS | 5 | 0.039 |
| AMERICAN JOURNAL OF CLINICAL PATHOLOGY | 5 | 0.039 |
| ALIMENTARY PHARMACOLOGY THERAPEUTICS | 5 | 0.039 |
| 11TH OESO WORLD CONFERENCE REFLUX DISEASE | 5 | 0.039 |
| TRANSLATIONAL ONCOLOGY | 4 | 0.031 |
| TOXICON | 4 | 0.031 |
| THERAPEUTIC ADVANCES IN GASTROENTEROLOGY | 4 | 0.031 |
| SOUTHERN MEDICAL JOURNAL | 4 | 0.031 |
| SEMINARS IN ONCOLOGY | 4 | 0.031 |
| SEMINARS IN CANCER BIOLOGY | 4 | 0.031 |
| RSC ADVANCES | 4 | 0.031 |
| REVISTA MEDICA DE CHILE | 4 | 0.031 |
| PROTEOMICS CLINICAL APPLICATIONS | 4 | 0.031 |
| PROGRESS IN BIOCHEMISTRY AND BIOPHYSICS | 4 | 0.031 |
| PHARMACOGENETICS AND GENOMICS | 4 | 0.031 |
| PATHOLOGE | 4 | 0.031 |
| NEOPLASIA | 4 | 0.031 |
| NATURE CLINICAL PRACTICE ONCOLOGY | 4 | 0.031 |
| MUTATION RESEARCH GENETIC TOXICOLOGY AND ENVIRONMENTAL MUTAGENESIS | 4 | 0.031 |
| MOLECULAR ONCOLOGY | 4 | 0.031 |
| MOLECULAR IMAGING AND BIOLOGY | 4 | 0.031 |
| MEDECINE NUCLEAIRE IMAGERIE FONCTIONNELLE ET METABOLIQUE | 4 | 0.031 |
| KAOHSIUNG JOURNAL OF MEDICAL SCIENCES | 4 | 0.031 |
| JOURNAL OF RESEARCH IN MEDICAL SCIENCES | 4 | 0.031 |
| JOURNAL OF RECONSTRUCTIVE MICROSURGERY | 4 | 0.031 |
| JOURNAL OF PROTEOMICS | 4 | 0.031 |
| JOURNAL OF PARENTERAL AND ENTERAL NUTRITION | 4 | 0.031 |
| JOURNAL OF ORAL PATHOLOGY MEDICINE | 4 | 0.031 |
| JOURNAL OF NIPPON MEDICAL SCHOOL | 4 | 0.031 |
| JOURNAL OF NEURO ONCOLOGY | 4 | 0.031 |
| JOURNAL OF MEDICINAL PLANTS RESEARCH | 4 | 0.031 |
| JOURNAL OF MEDICAL VIROLOGY | 4 | 0.031 |
| JOURNAL OF GASTRIC CANCER JGC | 4 | 0.031 |
| JOURNAL OF FOOD SCIENCE | 4 | 0.031 |
| JOURNAL OF CELLULAR PHYSIOLOGY | 4 | 0.031 |
| JOURNAL OF BIOLOGICAL CHEMISTRY | 4 | 0.031 |
| INTERNATIONAL JOURNAL OF PHARMACEUTICS | 4 | 0.031 |
| INTERNATIONAL JOURNAL OF FOOD MICROBIOLOGY | 4 | 0.031 |
| INTERNATIONAL JOURNAL OF EPIDEMIOLOGY | 4 | 0.031 |
| INTERNATIONAL JOURNAL OF ENVIRONMENTAL RESEARCH AND PUBLIC HEALTH | 4 | 0.031 |
| HUMAN IMMUNOLOGY | 4 | 0.031 |
| FOOD ADDITIVES AND CONTAMINANTS | 4 | 0.031 |
| EVIDENCE BASED COMPLEMENTARY AND ALTERNATIVE MEDICINE | 4 | 0.031 |
| EPIDEMIOLOGY | 4 | 0.031 |
| DISCOVERY MEDICINE | 4 | 0.031 |
| CYTOKINE | 4 | 0.031 |
| CURRENT OPINION IN CLINICAL NUTRITION AND METABOLIC CARE | 4 | 0.031 |
| CLINICAL RADIOLOGY | 4 | 0.031 |
| CLINICAL CHEMISTRY AND LABORATORY MEDICINE | 4 | 0.031 |
| CELLULAR AND MOLECULAR LIFE SCIENCES | 4 | 0.031 |
| CANCER JOURNAL | 4 | 0.031 |
| CANCER IMMUNOLOGY IMMUNOTHERAPY | 4 | 0.031 |
| CANCER GENETICS | 4 | 0.031 |
| CANADIAN JOURNAL OF GASTROENTEROLOGY | 4 | 0.031 |
| BRITISH JOURNAL OF RADIOLOGY | 4 | 0.031 |
| BIOORGANIC MEDICINAL CHEMISTRY LETTERS | 4 | 0.031 |
| BIOMATERIALS | 4 | 0.031 |
| BIOMARKERS IN MEDICINE | 4 | 0.031 |
| ARCHIVES OF INTERNAL MEDICINE | 4 | 0.031 |
| ANNALS OF DIAGNOSTIC PATHOLOGY | 4 | 0.031 |
| ANNALI ITALIANI DI CHIRURGIA | 4 | 0.031 |
| ANESTHESIA AND ANALGESIA | 4 | 0.031 |
| AMERICAN JOURNAL OF THE MEDICAL SCIENCES | 4 | 0.031 |
| AMERICAN JOURNAL OF RESPIRATORY AND CRITICAL CARE MEDICINE | 4 | 0.031 |
| AMERICAN JOURNAL OF OTOLARYNGOLOGY | 4 | 0.031 |
| ALCOHOL RESEARCH CURRENT REVIEWS | 4 | 0.031 |
| ALCOHOL AND ALCOHOLISM | 4 | 0.031 |
| AFRICAN HEALTH SCIENCES | 4 | 0.031 |
| ADVANCES IN EXPERIMENTAL MEDICINE AND BIOLOGY | 4 | 0.031 |
| ADVANCED DRUG DELIVERY REVIEWS | 4 | 0.031 |
| ACTA OTO LARYNGOLOGICA | 4 | 0.031 |
| 13TH OESO WORLD CONFERENCE THE ESOPHAGIOME II | 4 | 0.031 |
| WIENER KLINISCHE WOCHENSCHRIFT | 3 | 0.023 |
| VISZERALCHIRURGIE | 3 | 0.023 |
| VACCINE | 3 | 0.023 |
| TURKISH JOURNAL OF GASTROENTEROLOGY | 3 | 0.023 |
| TRANSPLANTATION PROCEEDINGS | 3 | 0.023 |
| TRANSLATIONAL RESEARCH | 3 | 0.023 |
| TOXICOLOGY IN VITRO | 3 | 0.023 |
| THERAPEUTIC ADVANCES IN MEDICAL ONCOLOGY | 3 | 0.023 |
| TARGETED ONCOLOGY | 3 | 0.023 |
| SURGICAL INFECTIONS | 3 | 0.023 |
| SURGERY FOR OBESITY AND RELATED DISEASES | 3 | 0.023 |
| SCANNING | 3 | 0.023 |
| ROMANIAN JOURNAL OF MORPHOLOGY AND EMBRYOLOGY | 3 | 0.023 |
| ROFO FORTSCHRITTE AUF DEM GEBIET DER RONTGENSTRAHLEN UND DER BILDGEBENDEN VERFAHREN | 3 | 0.023 |
| REVISTA PANAMERICANA DE SALUD PUBLICA PAN AMERICAN JOURNAL OF PUBLIC HEALTH | 3 | 0.023 |
| REVISTA CHILENA DE CIRUGIA | 3 | 0.023 |
| POSTGRADUATE MEDICAL JOURNAL | 3 | 0.023 |
| POSTEPY HIGIENY I MEDYCYNY DOSWIADCZALNEJ | 3 | 0.023 |
| ORAL SURGERY ORAL MEDICINE ORAL PATHOLOGY ORAL RADIOLOGY | 3 | 0.023 |
| OPEN MEDICINE | 3 | 0.023 |
| ONCOLOGY NURSING FORUM | 3 | 0.023 |
| OMICS A JOURNAL OF INTEGRATIVE BIOLOGY | 3 | 0.023 |
| OCCUPATIONAL AND ENVIRONMENTAL MEDICINE | 3 | 0.023 |
| NUTRITION REVIEWS | 3 | 0.023 |
| NUTRITION IN CLINICAL PRACTICE | 3 | 0.023 |
| MUTATION RESEARCH FUNDAMENTAL AND MOLECULAR MECHANISMS OF MUTAGENESIS | 3 | 0.023 |
| MINERVA MEDICA | 3 | 0.023 |
| METABOLISM CLINICAL AND EXPERIMENTAL | 3 | 0.023 |
| LIFE SCIENCES | 3 | 0.023 |
| LASERS IN SURGERY AND MEDICINE | 3 | 0.023 |
| LASER PHYSICS LETTERS | 3 | 0.023 |
| LARYNGOSCOPE | 3 | 0.023 |
| KOREAN JOURNAL OF PATHOLOGY | 3 | 0.023 |
| JOURNAL OF X RAY SCIENCE AND TECHNOLOGY | 3 | 0.023 |
| JOURNAL OF THE KOREAN SURGICAL SOCIETY | 3 | 0.023 |
| JOURNAL OF THE AMERICAN GERIATRICS SOCIETY | 3 | 0.023 |
| JOURNAL OF PROTEOME RESEARCH | 3 | 0.023 |
| JOURNAL OF PATHOLOGY | 3 | 0.023 |
| JOURNAL OF PAIN AND SYMPTOM MANAGEMENT | 3 | 0.023 |
| JOURNAL OF INORGANIC BIOCHEMISTRY | 3 | 0.023 |
| JOURNAL OF HUAZHONG UNIVERSITY OF SCIENCE AND TECHNOLOGY MEDICAL SCIENCES | 3 | 0.023 |
| JOURNAL OF HEMATOLOGY ONCOLOGY | 3 | 0.023 |
| JOURNAL OF ETHNOPHARMACOLOGY | 3 | 0.023 |
| JOURNAL OF EPIDEMIOLOGY | 3 | 0.023 |
| JOURNAL OF CLINICAL BIOCHEMISTRY AND NUTRITION | 3 | 0.023 |
| JOURNAL OF CHROMATOGRAPHY B ANALYTICAL TECHNOLOGIES IN THE BIOMEDICAL AND LIFE SCIENCES | 3 | 0.023 |
| JOURNAL OF AOAC INTERNATIONAL | 3 | 0.023 |
| JAMA OTOLARYNGOLOGY HEAD NECK SURGERY | 3 | 0.023 |
| IRISH JOURNAL OF MEDICAL SCIENCE | 3 | 0.023 |
| INTERNIST | 3 | 0.023 |
| INTERNATIONAL JOURNAL OF SURGICAL PATHOLOGY | 3 | 0.023 |
| INTERNATIONAL JOURNAL OF ORAL AND MAXILLOFACIAL SURGERY | 3 | 0.023 |
| INTERNATIONAL JOURNAL OF MEDICAL SCIENCES | 3 | 0.023 |
| INTERNATIONAL JOURNAL OF MEDICAL ROBOTICS AND COMPUTER ASSISTED SURGERY | 3 | 0.023 |
| INDIAN JOURNAL OF MEDICAL RESEARCH | 3 | 0.023 |
| IN VIVO | 3 | 0.023 |
| IMMUNOLOGICAL INVESTIGATIONS | 3 | 0.023 |
| HUMAN GENETICS | 3 | 0.023 |
| HUMAN GENE THERAPY | 3 | 0.023 |
| HUMAN EXPERIMENTAL TOXICOLOGY | 3 | 0.023 |
| HNO | 3 | 0.023 |
| FOOD RESEARCH INTERNATIONAL | 3 | 0.023 |
| FOOD ADDITIVES CONTAMINANTS PART B SURVEILLANCE | 3 | 0.023 |
| FEBS LETTERS | 3 | 0.023 |
| EUROPEAN JOURNAL OF ANAESTHESIOLOGY | 3 | 0.023 |
| ENVIRONMENT INTERNATIONAL | 3 | 0.023 |
| ENDOSCOPIC ULTRASOUND | 3 | 0.023 |
| DYSPHAGIA | 3 | 0.023 |
| DRUG DESIGN DEVELOPMENT AND THERAPY | 3 | 0.023 |
| DIAGNOSTIC AND INTERVENTIONAL RADIOLOGY | 3 | 0.023 |
| DEUTSCHES ARZTEBLATT INTERNATIONAL | 3 | 0.023 |
| CURRENT OPINION IN PULMONARY MEDICINE | 3 | 0.023 |
| CURRENT OPINION IN PHARMACOLOGY | 3 | 0.023 |
| CURRENT MEDICINAL CHEMISTRY | 3 | 0.023 |
| CLINICAL LUNG CANCER | 3 | 0.023 |
| CLINICAL IMMUNOLOGY | 3 | 0.023 |
| CHEMOTHERAPY | 3 | 0.023 |
| CHEMICO BIOLOGICAL INTERACTIONS | 3 | 0.023 |
| CHEMICAL RESEARCH IN TOXICOLOGY | 3 | 0.023 |
| CELL DEATH DISEASE | 3 | 0.023 |
| CELL BIOLOGY INTERNATIONAL | 3 | 0.023 |
| CELL AND TISSUE RESEARCH | 3 | 0.023 |
| CANCER CELL INTERNATIONAL | 3 | 0.023 |
| CANCER AND METASTASIS REVIEWS | 3 | 0.023 |
| CANADIAN ASSOCIATION OF RADIOLOGISTS JOURNAL JOURNAL DE L ASSOCIATION CANADIENNE DES RADIOLOGISTES | 3 | 0.023 |
| BRITISH JOURNAL OF ORAL MAXILLOFACIAL SURGERY | 3 | 0.023 |
| BRITISH JOURNAL OF BIOMEDICAL SCIENCE | 3 | 0.023 |
| BRAZILIAN JOURNAL OF MEDICAL AND BIOLOGICAL RESEARCH | 3 | 0.023 |
| BRAZILIAN ARCHIVES OF BIOLOGY AND TECHNOLOGY | 3 | 0.023 |
| BMC PUBLIC HEALTH | 3 | 0.023 |
| BMC HEALTH SERVICES RESEARCH | 3 | 0.023 |
| BMC COMPLEMENTARY AND ALTERNATIVE MEDICINE | 3 | 0.023 |
| BIOMEDICAL RESEARCH INDIA | 3 | 0.023 |
| BIOLOGICAL BASIS OF ALCOHOL INDUCED CANCER | 3 | 0.023 |
| BIOCHEMICAL GENETICS | 3 | 0.023 |
| ASIA PACIFIC JOURNAL OF CLINICAL NUTRITION | 3 | 0.023 |
| ANZ JOURNAL OF SURGERY | 3 | 0.023 |
| ANNALS OF SURGICAL TREATMENT AND RESEARCH | 3 | 0.023 |
| ADDICTION | 3 | 0.023 |
| ACTA MEDICA OKAYAMA | 3 | 0.023 |
| ACTA CHIRURGICA BELGICA | 3 | 0.023 |
| ACTA BIOCHIMICA ET BIOPHYSICA SINICA | 3 | 0.023 |
| WSPOLCZESNA ONKOLOGIA CONTEMPORARY ONCOLOGY | 2 | 0.015 |
| WIDEOCHIRURGIA I INNE TECHNIKI MALOINWAZYJNE | 2 | 0.015 |
| VOJNOSANITETSKI PREGLED | 2 | 0.015 |
| VALUE IN HEALTH | 2 | 0.015 |
| UROLOGIC ONCOLOGY SEMINARS AND ORIGINAL INVESTIGATIONS | 2 | 0.015 |
| UROLOGE | 2 | 0.015 |
| UPSALA JOURNAL OF MEDICAL SCIENCES | 2 | 0.015 |
| TOXINS | 2 | 0.015 |
| TOXICOLOGY | 2 | 0.015 |
| TOXICOLOGICAL SCIENCES | 2 | 0.015 |
| THROMBOSIS RESEARCH | 2 | 0.015 |
| THESCIENTIFICWORLDJOURNAL | 2 | 0.015 |
| TETRAHEDRON LETTERS | 2 | 0.015 |
| SRPSKI ARHIV ZA CELOKUPNO LEKARSTVO | 2 | 0.015 |
| SPECTROSCOPY AND SPECTRAL ANALYSIS | 2 | 0.015 |
| SMALL | 2 | 0.015 |
| SEMINARS IN ROENTGENOLOGY | 2 | 0.015 |
| SEMINARS IN NUCLEAR MEDICINE | 2 | 0.015 |
| SCANDINAVIAN JOURNAL OF IMMUNOLOGY | 2 | 0.015 |
| SAO PAULO MEDICAL JOURNAL | 2 | 0.015 |
| ROMANIAN BIOTECHNOLOGICAL LETTERS | 2 | 0.015 |
| RISK ANALYSIS | 2 | 0.015 |
| RHEUMATIC DISEASE CLINICS OF NORTH AMERICA | 2 | 0.015 |
| REVISTA DE SAUDE PUBLICA | 2 | 0.015 |
| RESPIRATION | 2 | 0.015 |
| REGIONAL ANESTHESIA AND PAIN MEDICINE | 2 | 0.015 |
| RADIOLOGIA MEDICA | 2 | 0.015 |
| QUARTERLY JOURNAL OF NUCLEAR MEDICINE AND MOLECULAR IMAGING | 2 | 0.015 |
| QUALITY ASSURANCE AND SAFETY OF CROPS FOODS | 2 | 0.015 |
| QUALITATIVE HEALTH RESEARCH | 2 | 0.015 |
| PRESSE MEDICALE | 2 | 0.015 |
| POLISH JOURNAL OF PATHOLOGY | 2 | 0.015 |
| PLOS MEDICINE | 2 | 0.015 |
| PHARMACOTHERAPY | 2 | 0.015 |
| PHARMACOLOGICAL RESEARCH | 2 | 0.015 |
| PHARMACOECONOMICS | 2 | 0.015 |
| PHARMACEUTICAL RESEARCH | 2 | 0.015 |
| PATHOLOGY | 2 | 0.015 |
| PANCREAS | 2 | 0.015 |
| ONCOGENESIS | 2 | 0.015 |
| NATURE GENETICS | 2 | 0.015 |
| MUTAGENESIS | 2 | 0.015 |
| MOLECULES AND CELLS | 2 | 0.015 |
| MOLECULAR THERAPY | 2 | 0.015 |
| MOLECULAR PHARMACEUTICS | 2 | 0.015 |
| MOLECULAR GENETICS AND GENOMICS | 2 | 0.015 |
| MOLECULAR ENDOCRINOLOGY | 2 | 0.015 |
| MOLECULAR CELLULAR PROTEOMICS | 2 | 0.015 |
| MOLECULAR BIOSYSTEMS | 2 | 0.015 |
| MOLECULAR BIOLOGY OF THE CELL | 2 | 0.015 |
| MOLECULAR AND CELLULAR BIOLOGY | 2 | 0.015 |
| MINIMALLY INVASIVE THERAPY ALLIED TECHNOLOGIES | 2 | 0.015 |
| MICROSURGERY | 2 | 0.015 |
| MEDICAL PRINCIPLES AND PRACTICE | 2 | 0.015 |
| MEDICAL MYCOLOGY | 2 | 0.015 |
| MEDICAL DECISION MAKING | 2 | 0.015 |
| MEDICAL CLINICS OF NORTH AMERICA | 2 | 0.015 |
| MAMMALIAN GENOME | 2 | 0.015 |
| LWT FOOD SCIENCE AND TECHNOLOGY | 2 | 0.015 |
| LASER PHYSICS | 2 | 0.015 |
| JOVE JOURNAL OF VISUALIZED EXPERIMENTS | 2 | 0.015 |
| JOURNAL OF ZHEJIANG UNIVERSITY SCIENCE B | 2 | 0.015 |
| JOURNAL OF VASCULAR ACCESS | 2 | 0.015 |
| JOURNAL OF TRADITIONAL CHINESE MEDICINE | 2 | 0.015 |
| JOURNAL OF THE AMERICAN DIETETIC ASSOCIATION | 2 | 0.015 |
| JOURNAL OF THE ACADEMY OF NUTRITION AND DIETETICS | 2 | 0.015 |
| JOURNAL OF RECEPTORS AND SIGNAL TRANSDUCTION | 2 | 0.015 |
| JOURNAL OF PHYTOPATHOLOGY | 2 | 0.015 |
| JOURNAL OF PHOTOCHEMISTRY AND PHOTOBIOLOGY B BIOLOGY | 2 | 0.015 |
| JOURNAL OF PEDIATRIC SURGERY | 2 | 0.015 |
| JOURNAL OF PEDIATRIC HEMATOLOGY ONCOLOGY | 2 | 0.015 |
| JOURNAL OF PALLIATIVE CARE | 2 | 0.015 |
| JOURNAL OF OCCUPATIONAL AND ENVIRONMENTAL MEDICINE | 2 | 0.015 |
| JOURNAL OF NEUROSURGERY | 2 | 0.015 |
| JOURNAL OF NATURAL PRODUCTS | 2 | 0.015 |
| JOURNAL OF NANOSCIENCE AND NANOTECHNOLOGY | 2 | 0.015 |
| JOURNAL OF MOLECULAR HISTOLOGY | 2 | 0.015 |
| JOURNAL OF MINIMAL ACCESS SURGERY | 2 | 0.015 |
| JOURNAL OF MEDICINAL FOOD | 2 | 0.015 |
| JOURNAL OF MEDICAL MICROBIOLOGY | 2 | 0.015 |
| JOURNAL OF MAGNETIC RESONANCE IMAGING | 2 | 0.015 |
| JOURNAL OF LARYNGOLOGY AND OTOLOGY | 2 | 0.015 |
| JOURNAL OF INVESTIGATIVE DERMATOLOGY | 2 | 0.015 |
| JOURNAL OF HISTOCHEMISTRY CYTOCHEMISTRY | 2 | 0.015 |
| JOURNAL OF GERIATRIC ONCOLOGY | 2 | 0.015 |
| JOURNAL OF FUNCTIONAL FOODS | 2 | 0.015 |
| JOURNAL OF EPIDEMIOLOGY AND COMMUNITY HEALTH | 2 | 0.015 |
| JOURNAL OF EMERGENCY MEDICINE | 2 | 0.015 |
| JOURNAL OF DERMATOLOGY | 2 | 0.015 |
| JOURNAL OF CONTEMPORARY BRACHYTHERAPY | 2 | 0.015 |
| JOURNAL OF CLINICAL LABORATORY ANALYSIS | 2 | 0.015 |
| JOURNAL OF CHEMOTHERAPY | 2 | 0.015 |
| JOURNAL OF CELLULAR AND MOLECULAR MEDICINE | 2 | 0.015 |
| JOURNAL OF CANCER EDUCATION | 2 | 0.015 |
| JOURNAL OF CACHEXIA SARCOPENIA AND MUSCLE | 2 | 0.015 |
| JOURNAL OF BIOMEDICAL SCIENCE | 2 | 0.015 |
| JOURNAL OF ANESTHESIA | 2 | 0.015 |
| JOURNAL DE CHIRURGIE | 2 | 0.015 |
| ISRAEL MEDICAL ASSOCIATION JOURNAL | 2 | 0.015 |
| IRANIAN JOURNAL OF BASIC MEDICAL SCIENCES | 2 | 0.015 |
| INTERNATIONAL JOURNAL OF UROLOGY | 2 | 0.015 |
| INTERNATIONAL JOURNAL OF OCCUPATIONAL AND ENVIRONMENTAL HEALTH | 2 | 0.015 |
| INTERNATIONAL JOURNAL OF HYGIENE AND ENVIRONMENTAL HEALTH | 2 | 0.015 |
| INTERNATIONAL JOURNAL OF CLINICAL PRACTICE | 2 | 0.015 |
| INTERNATIONAL JOURNAL OF BIOLOGICAL SCIENCES | 2 | 0.015 |
| INTENSIVE CARE MEDICINE | 2 | 0.015 |
| INFLAMMATION RESEARCH | 2 | 0.015 |
| INDIAN JOURNAL OF PATHOLOGY AND MICROBIOLOGY | 2 | 0.015 |
| HYBRIDOMA | 2 | 0.015 |
| HORMONES INTERNATIONAL JOURNAL OF ENDOCRINOLOGY AND METABOLISM | 2 | 0.015 |
| GENE THERAPY AND MOLECULAR BIOLOGY | 2 | 0.015 |
| FREE RADICAL BIOLOGY AND MEDICINE | 2 | 0.015 |
| FOOD FUNCTION | 2 | 0.015 |
| FEBS JOURNAL | 2 | 0.015 |
| EXPERT REVIEW OF PROTEOMICS | 2 | 0.015 |
| EXPERT REVIEW OF PHARMACOECONOMICS OUTCOMES RESEARCH | 2 | 0.015 |
| EXPERT REVIEW OF MOLECULAR DIAGNOSTICS | 2 | 0.015 |
| EXPERIMENTAL AND MOLECULAR MEDICINE | 2 | 0.015 |
| EUROPEAN RESPIRATORY JOURNAL | 2 | 0.015 |
| EUROPEAN JOURNAL OF PEDIATRIC SURGERY | 2 | 0.015 |
| EUROPEAN JOURNAL OF ONCOLOGY NURSING | 2 | 0.015 |
| EUROPEAN JOURNAL OF NUTRITION | 2 | 0.015 |
| EUROPEAN JOURNAL OF INTERNAL MEDICINE | 2 | 0.015 |
| EUROPEAN JOURNAL OF IMMUNOLOGY | 2 | 0.015 |
| EUROPEAN JOURNAL OF HISTOCHEMISTRY | 2 | 0.015 |
| EUROPEAN JOURNAL OF HAEMATOLOGY | 2 | 0.015 |
| EUROPEAN JOURNAL OF GYNAECOLOGICAL ONCOLOGY | 2 | 0.015 |
| EUROPEAN JOURNAL OF DERMATOLOGY | 2 | 0.015 |
| EUROPEAN JOURNAL OF CLINICAL NUTRITION | 2 | 0.015 |
| EUROPEAN JOURNAL OF CANCER CARE | 2 | 0.015 |
| EUROPEAN ANNALS OF OTORHINOLARYNGOLOGY HEAD AND NECK DISEASES | 2 | 0.015 |
| ENVIRONMENTAL TOXICOLOGY AND PHARMACOLOGY | 2 | 0.015 |
| ENVIRONMENTAL SCIENCE TECHNOLOGY | 2 | 0.015 |
| ENVIRONMENTAL RESEARCH | 2 | 0.015 |
| ENVIRONMENTAL HEALTH PERSPECTIVES | 2 | 0.015 |
| ENVIRONMENTAL HEALTH | 2 | 0.015 |
| ENVIRONMENTAL GEOCHEMISTRY AND HEALTH | 2 | 0.015 |
| ENVIRONMENTAL AND MOLECULAR MUTAGENESIS | 2 | 0.015 |
| ENDOSKOPIE HEUTE | 2 | 0.015 |
| DRUG AND CHEMICAL TOXICOLOGY | 2 | 0.015 |
| DISEASES OF THE COLON RECTUM | 2 | 0.015 |
| DIAGNOSTIC CYTOPATHOLOGY | 2 | 0.015 |
| DANISH MEDICAL JOURNAL | 2 | 0.015 |
| CYTOGENETIC AND GENOME RESEARCH | 2 | 0.015 |
| CURRENT OPINION IN RHEUMATOLOGY | 2 | 0.015 |
| CURRENT MOLECULAR MEDICINE | 2 | 0.015 |
| CURRENT MEDICAL RESEARCH AND OPINION | 2 | 0.015 |
| CURRENT MEDICAL IMAGING REVIEWS | 2 | 0.015 |
| CURRENT GENOMICS | 2 | 0.015 |
| CRITICAL REVIEWS IN FOOD SCIENCE AND NUTRITION | 2 | 0.015 |
| COMPUTER METHODS AND PROGRAMS IN BIOMEDICINE | 2 | 0.015 |
| COLORECTAL DISEASE | 2 | 0.015 |
| CLINICS | 2 | 0.015 |
| CLINICAL THERAPEUTICS | 2 | 0.015 |
| CLINICAL LABORATORY | 2 | 0.015 |
| CLINICAL JOURNAL OF ONCOLOGY NURSING | 2 | 0.015 |
| CLINICAL IMAGING | 2 | 0.015 |
| CLINICAL GENITOURINARY CANCER | 2 | 0.015 |
| CIRUGIA Y CIRUJANOS | 2 | 0.015 |
| CHEMICAL RESEARCH IN CHINESE UNIVERSITIES | 2 | 0.015 |
| CELLULAR MOLECULAR BIOLOGY LETTERS | 2 | 0.015 |
| CELLULAR AND MOLECULAR BIOLOGY | 2 | 0.015 |
| CANCER IMMUNOLOGY RESEARCH | 2 | 0.015 |
| CANCER DISCOVERY | 2 | 0.015 |
| CANCER CELL | 2 | 0.015 |
| CANADIAN MEDICAL ASSOCIATION JOURNAL | 2 | 0.015 |
| BRITISH JOURNAL OF ANAESTHESIA | 2 | 0.015 |
| BONE | 2 | 0.015 |
| BMC PALLIATIVE CARE | 2 | 0.015 |
| BMC MEDICAL GENETICS | 2 | 0.015 |
| BMC CELL BIOLOGY | 2 | 0.015 |
| BIOSCIENCE TRENDS | 2 | 0.015 |
| BIOMEDICAL RESEARCH TOKYO | 2 | 0.015 |
| BIOMEDICAL PAPERS OLOMOUC | 2 | 0.015 |
| BIOMEDICAL OPTICS EXPRESS | 2 | 0.015 |
| BIOMEDICAL CHROMATOGRAPHY | 2 | 0.015 |
| BIOMEDICAL AND ENVIRONMENTAL SCIENCES | 2 | 0.015 |
| BIOCHIMICA ET BIOPHYSICA ACTA MOLECULAR CELL RESEARCH | 2 | 0.015 |
| BIOCHIMICA ET BIOPHYSICA ACTA MOLECULAR BASIS OF DISEASE | 2 | 0.015 |
| BIOCHIMICA ET BIOPHYSICA ACTA GENE REGULATORY MECHANISMS | 2 | 0.015 |
| BIOCHEMISTRY AND CELL BIOLOGY BIOCHIMIE ET BIOLOGIE CELLULAIRE | 2 | 0.015 |
| BASIC CLINICAL PHARMACOLOGY TOXICOLOGY | 2 | 0.015 |
| BANGLADESH JOURNAL OF PHARMACOLOGY | 2 | 0.015 |
| AUTOPHAGY | 2 | 0.015 |
| ASIAN PACIFIC JOURNAL OF TROPICAL MEDICINE | 2 | 0.015 |
| ASIAN JOURNAL OF SURGERY | 2 | 0.015 |
| ASIAN BIOMEDICINE | 2 | 0.015 |
| ARCHIVES OF TOXICOLOGY | 2 | 0.015 |
| ARCHIVES OF OTOLARYNGOLOGY HEAD NECK SURGERY | 2 | 0.015 |
| ARCHIVES OF MEDICAL SCIENCE | 2 | 0.015 |
| ARCHIVES OF GYNECOLOGY AND OBSTETRICS | 2 | 0.015 |
| ARCHIVES OF DERMATOLOGICAL RESEARCH | 2 | 0.015 |
| ARCHIVES OF BIOCHEMISTRY AND BIOPHYSICS | 2 | 0.015 |
| APPLIED PHYSICS LETTERS | 2 | 0.015 |
| APOPTOSIS | 2 | 0.015 |
| ANNALS OF THORACIC MEDICINE | 2 | 0.015 |
| ANNALS OF PLASTIC SURGERY | 2 | 0.015 |
| ANNALS OF PHARMACOTHERAPY | 2 | 0.015 |
| ANNALS OF OTOLOGY RHINOLOGY AND LARYNGOLOGY | 2 | 0.015 |
| ANNALS OF EPIDEMIOLOGY | 2 | 0.015 |
| ANALYTICAL METHODS | 2 | 0.015 |
| ANALYTICAL CHEMISTRY | 2 | 0.015 |
| ANALYTICAL AND BIOANALYTICAL CHEMISTRY | 2 | 0.015 |
| ANAESTHESIST | 2 | 0.015 |
| AMERICAN JOURNAL OF PUBLIC HEALTH | 2 | 0.015 |
| AMERICAN JOURNAL OF PHYSIOLOGY GASTROINTESTINAL AND LIVER PHYSIOLOGY | 2 | 0.015 |
| AMERICAN JOURNAL OF MEDICINE | 2 | 0.015 |
| AMERICAN JOURNAL OF INFECTION CONTROL | 2 | 0.015 |
| AMERICAN JOURNAL OF INDUSTRIAL MEDICINE | 2 | 0.015 |
| ALCOHOL | 2 | 0.015 |
| AFRICAN JOURNAL OF BIOTECHNOLOGY | 2 | 0.015 |
| ADVANCES IN MEDICAL SCIENCES | 2 | 0.015 |
| ADVANCES IN ANATOMIC PATHOLOGY | 2 | 0.015 |
| ACTA RADIOLOGICA | 2 | 0.015 |
| ACTA HISTOCHEMICA | 2 | 0.015 |
| ACTA CYTOLOGICA | 2 | 0.015 |
| (756 Source Titles {0} {1} value(s) outside display options.) |  |  |
| (0 records (0.000%){0} records{1} do not contain data in the field being analyzed.) | |  |

**Supplemental Table 4.** Table that demonstrates countries/regions contributed to publications on esophageal and esophagogastric junction cancer from the Web of Science ^TM^ Core Collection.

| Countries/Territories | records | % of 12978 |
| --- | --- | --- |
| USA | 3246 | 25.012 |
| PEOPLES R CHINA | 2932 | 22.592 |
| JAPAN | 2267 | 17.468 |
| GERMANY | 940 | 7.243 |
| ENGLAND | 571 | 4.4 |
| NETHERLANDS | 498 | 3.837 |
| ITALY | 435 | 3.352 |
| SOUTH KOREA | 417 | 3.213 |
| FRANCE | 412 | 3.175 |
| TAIWAN | 352 | 2.712 |
| CANADA | 297 | 2.288 |
| AUSTRALIA | 265 | 2.042 |
| IRAN | 242 | 1.865 |
| INDIA | 239 | 1.842 |
| SWEDEN | 188 | 1.449 |
| SPAIN | 173 | 1.333 |
| SWITZERLAND | 167 | 1.287 |
| BRAZIL | 125 | 0.963 |
| AUSTRIA | 124 | 0.955 |
| BELGIUM | 122 | 0.94 |
| TURKEY | 116 | 0.894 |
| POLAND | 102 | 0.786 |
| IRELAND | 101 | 0.778 |
| DENMARK | 91 | 0.701 |
| SOUTH AFRICA | 78 | 0.601 |
| GREECE | 75 | 0.578 |
| SCOTLAND | 73 | 0.562 |
| NORWAY | 70 | 0.539 |
| FINLAND | 63 | 0.485 |
| SINGAPORE | 53 | 0.408 |
| RUSSIA | 52 | 0.401 |
| CZECH REPUBLIC | 46 | 0.354 |
| WALES | 44 | 0.339 |
| HUNGARY | 44 | 0.339 |
| NORTH IRELAND | 43 | 0.331 |
| PORTUGAL | 41 | 0.316 |
| ROMANIA | 39 | 0.301 |
| ISRAEL | 33 | 0.254 |
| THAILAND | 24 | 0.185 |
| ARGENTINA | 21 | 0.162 |
| PAKISTAN | 20 | 0.154 |
| MALAYSIA | 20 | 0.154 |
| MEXICO | 19 | 0.146 |
| CHILE | 18 | 0.139 |
| SERBIA | 17 | 0.131 |
| EGYPT | 17 | 0.131 |
| CROATIA | 15 | 0.116 |
| URUGUAY | 12 | 0.092 |
| SAUDI ARABIA | 12 | 0.092 |
| NEW ZEALAND | 12 | 0.092 |
| KENYA | 12 | 0.092 |
| COLOMBIA | 11 | 0.085 |
| KAZAKHSTAN | 10 | 0.077 |
| SLOVENIA | 9 | 0.069 |
| NIGERIA | 9 | 0.069 |
| TANZANIA | 8 | 0.062 |
| INDONESIA | 8 | 0.062 |
| JORDAN | 7 | 0.054 |
| SUDAN | 6 | 0.046 |
| UGANDA | 5 | 0.039 |
| NEPAL | 5 | 0.039 |
| CUBA | 5 | 0.039 |
| BULGARIA | 5 | 0.039 |
| ZIMBABWE | 4 | 0.031 |
| ZAMBIA | 4 | 0.031 |
| UKRAINE | 4 | 0.031 |
| U ARAB EMIRATES | 4 | 0.031 |
| TUNISIA | 4 | 0.031 |
| SRI LANKA | 4 | 0.031 |
| SLOVAKIA | 4 | 0.031 |
| PHILIPPINES | 4 | 0.031 |
| LEBANON | 4 | 0.031 |
| GHANA | 4 | 0.031 |
| ESTONIA | 4 | 0.031 |
| MALAWI | 3 | 0.023 |
| LITHUANIA | 3 | 0.023 |
| BYELARUS | 3 | 0.023 |
| BANGLADESH | 3 | 0.023 |
| ALBANIA | 3 | 0.023 |
| VIETNAM | 2 | 0.015 |
| UZBEKISTAN | 2 | 0.015 |
| REP OF GEORGIA | 2 | 0.015 |
| MOROCCO | 2 | 0.015 |
| MALTA | 2 | 0.015 |
| LUXEMBOURG | 2 | 0.015 |
| LATVIA | 2 | 0.015 |
| IRAQ | 2 | 0.015 |
| GUATEMALA | 2 | 0.015 |
| GRENADA | 2 | 0.015 |
| ETHIOPIA | 2 | 0.015 |
| CYPRUS | 2 | 0.015 |
| (81 Countries/Territories {0} {1} value(s) outside display options.) | | |
| (416 records (3.205%){0} records{1} do not contain data in the field being analyzed.) | | |

**Supplemental Table 5.** Table that demonstrates institutions contributed to publications on esophageal and esophagogastric junction cancer from the Web of Science ^TM^ Core Collection.

| Organizations | records | % of 12978 |
| --- | --- | --- |
| UNIV TEXAS MD ANDERSON CANC CTR | 334 | 2.574 |
| CHINESE ACAD MED SCI | 215 | 1.657 |
| NATL CANC CTR JAPAN | 213 | 1.641 |
| ZHENGZHOU UNIV | 210 | 1.618 |
| NCI | 185 | 1.425 |
| MAYO CLIN | 171 | 1.318 |
| SUN YAT SEN UNIV | 169 | 1.302 |
| OSAKA UNIV | 157 | 1.21 |
| MEM SLOAN KETTERING CANC CTR | 154 | 1.187 |
| SHANDONG UNIV | 153 | 1.179 |
| NANJING MED UNIV | 149 | 1.148 |
| HARVARD UNIV | 146 | 1.125 |
| FUDAN UNIV | 142 | 1.094 |
| UNIV TEHRAN MED SCI | 135 | 1.04 |
| KEIO UNIV | 133 | 1.025 |
| PEKING UNION MED COLL | 126 | 0.971 |
| KAROLINSKA INST | 125 | 0.963 |
| TECH UNIV MUNICH | 122 | 0.94 |
| SICHUAN UNIV | 117 | 0.902 |
| HEBEI MED UNIV | 116 | 0.894 |
| UNIV AMSTERDAM | 111 | 0.855 |
| UNIV PITTSBURGH | 110 | 0.848 |
| KYUSHU UNIV | 106 | 0.817 |
| SHANGHAI JIAO TONG UNIV | 104 | 0.801 |
| CHIBA UNIV | 100 | 0.771 |
| UNIV COLOGNE | 98 | 0.755 |
| UNIV MED CTR UTRECHT | 97 | 0.747 |
| UNIV HONG KONG | 89 | 0.686 |
| KYOTO UNIV | 88 | 0.678 |
| UNIV TORONTO | 87 | 0.67 |
| INT AGCY RES CANC | 87 | 0.67 |
| AICHI CANC CTR HOSP | 87 | 0.67 |
| JOHNS HOPKINS UNIV | 86 | 0.663 |
| UNIV MARYLAND | 85 | 0.655 |
| PEKING UNIV | 85 | 0.655 |
| UNIV PADUA | 82 | 0.632 |
| UNIV TOKYO | 81 | 0.624 |
| UNIV PENN | 81 | 0.624 |
| SHANTOU UNIV | 80 | 0.616 |
| MASSACHUSETTS GEN HOSP | 80 | 0.616 |
| GUNMA UNIV | 79 | 0.609 |
| NATL TAIWAN UNIV HOSP | 78 | 0.601 |
| KUMAMOTO UNIV | 78 | 0.601 |
| UNIV ULSAN | 77 | 0.593 |
| UNIV CHICAGO | 77 | 0.593 |
| NATL YANG MING UNIV | 76 | 0.586 |
| UNIV WASHINGTON | 73 | 0.562 |
| SHIZUOKA CANC CTR | 73 | 0.562 |
| UNIV MICHIGAN | 72 | 0.555 |
| SOOCHOW UNIV | 72 | 0.555 |
| OSAKA MED CTR CANC CARDIOVASC DIS | 71 | 0.547 |
| UNIV SO CALIF | 70 | 0.539 |
| TOHOKU UNIV | 70 | 0.539 |
| ZHEJIANG UNIV | 69 | 0.532 |
| THIRD MIL MED UNIV | 68 | 0.524 |
| DUKE UNIV | 68 | 0.524 |
| CLEVELAND CLIN | 68 | 0.524 |
| CHANG GUNG UNIV | 68 | 0.524 |
| SEOUL NATL UNIV | 67 | 0.516 |
| UNIV HOSP | 66 | 0.509 |
| STANFORD UNIV | 66 | 0.509 |
| UNIV S FLORIDA | 65 | 0.501 |
| NATL TAIWAN UNIV | 65 | 0.501 |
| UNIV N CAROLINA | 64 | 0.493 |
| OHIO STATE UNIV | 63 | 0.485 |
| ZHEJIANG CANC HOSP | 62 | 0.478 |
| OREGON HLTH SCI UNIV | 62 | 0.478 |
| SUNGKYUNKWAN UNIV | 61 | 0.47 |
| CHINA MED UNIV | 61 | 0.47 |
| HIROSHIMA UNIV | 60 | 0.462 |
| UNIV LONDON IMPERIAL COLL SCI TECHNOL MED | 59 | 0.455 |
| UNIV GRONINGEN | 59 | 0.455 |
| NAGOYA UNIV | 59 | 0.455 |
| JIANGSU UNIV | 59 | 0.455 |
| KYOTO PREFECTURAL UNIV MED | 58 | 0.447 |
| KINGS COLL LONDON | 58 | 0.447 |
| SOUTHEAST UNIV | 57 | 0.439 |
| UNIV MILAN | 56 | 0.431 |
| ST JAMES HOSP | 56 | 0.431 |
| OKAYAMA UNIV | 56 | 0.431 |
| ERASMUS MC | 56 | 0.431 |
| FOX CHASE CANC CTR | 55 | 0.424 |
| HUAZHONG UNIV SCI TECHNOL | 54 | 0.416 |
| YONSEI UNIV | 53 | 0.408 |
| UNIV CALIF LOS ANGELES | 53 | 0.408 |
| MT SINAI SCH MED | 53 | 0.408 |
| CHINESE ACAD SCI | 53 | 0.408 |
| UNIV OXFORD | 52 | 0.401 |
| MED UNIV VIENNA | 52 | 0.401 |
| WASHINGTON UNIV | 51 | 0.393 |
| CAPITAL MED UNIV | 51 | 0.393 |
| VANDERBILT UNIV | 50 | 0.385 |
| NATL CANC CTR HOSP EAST | 50 | 0.385 |
| KAGOSHIMA UNIV | 50 | 0.385 |
| FOURTH MIL MED UNIV | 50 | 0.385 |
| CHINESE UNIV HONG KONG | 50 | 0.385 |
| UNIV ROCHESTER | 49 | 0.378 |
| NORTHWESTERN UNIV | 49 | 0.378 |
| KAOHSIUNG MED UNIV | 49 | 0.378 |
| UNIV MED CTR HAMBURG EPPENDORF | 48 | 0.37 |
| ROSWELL PK CANC INST | 48 | 0.37 |
| GERMAN CANC RES CTR | 48 | 0.37 |
| TOKYO MED DENT UNIV | 47 | 0.362 |
| TAIPEI VET GEN HOSP | 47 | 0.362 |
| CHANG GUNG MEM HOSP | 47 | 0.362 |
| BAYLOR COLL MED | 47 | 0.362 |
| ANHUI MED UNIV | 47 | 0.362 |
| UNIV QUEENSLAND | 46 | 0.354 |
| XI AN JIAO TONG UNIV | 45 | 0.347 |
| JOHANNES GUTENBERG UNIV MAINZ | 45 | 0.347 |
| DANA FARBER CANC INST | 45 | 0.347 |
| TORANOMON GEN HOSP | 44 | 0.339 |
| SHANDONG CANC HOSP INST | 44 | 0.339 |
| EMORY UNIV | 44 | 0.339 |
| BRIGHAM WOMENS HOSP | 44 | 0.339 |
| XINXIANG MED UNIV | 43 | 0.331 |
| HOKKAIDO UNIV | 43 | 0.331 |
| UNIV CALIF SAN DIEGO | 42 | 0.324 |
| HEIDELBERG UNIV | 42 | 0.324 |
| FUJIAN MED UNIV | 42 | 0.324 |
| COLUMBIA UNIV | 42 | 0.324 |
| KINKI UNIV | 41 | 0.316 |
| JAPANESE FDN CANC RES | 41 | 0.316 |
| YALE UNIV | 40 | 0.308 |
| THOMAS JEFFERSON UNIV | 40 | 0.308 |
| CHINESE PEOPLES LIBERAT ARMY GEN HOSP | 40 | 0.308 |
| UNIV CAMBRIDGE | 39 | 0.301 |
| ROYAL MARSDEN HOSP | 39 | 0.301 |
| XINJIANG MED UNIV | 38 | 0.293 |
| UNIV MINNESOTA | 38 | 0.293 |
| UNIV DUSSELDORF | 38 | 0.293 |
| TOKAI UNIV | 38 | 0.293 |
| NATL CANC CTR HOSP E | 38 | 0.293 |
| MORGAN STATE UNIV | 38 | 0.293 |
| CASE WESTERN RESERVE UNIV | 38 | 0.293 |
| YOKOHAMA CITY UNIV | 37 | 0.285 |
| UNIV CALIF IRVINE | 37 | 0.285 |
| SECOND MIL MED UNIV | 37 | 0.285 |
| KURUME UNIV | 37 | 0.285 |
| FRED HUTCHINSON CANC RES CTR | 37 | 0.285 |
| UNIV SYDNEY | 36 | 0.277 |
| UNIV HEIDELBERG HOSP | 36 | 0.277 |
| UNIV HEIDELBERG | 36 | 0.277 |
| UNIV FREIBURG | 36 | 0.277 |
| KYUSHU NATL CANC CTR | 36 | 0.277 |
| KOBE UNIV | 36 | 0.277 |
| KANAGAWA CANC CTR | 36 | 0.277 |
| INSERM | 36 | 0.277 |
| BROWN UNIV | 36 | 0.277 |
| VIRGINIA MASON MED CTR | 35 | 0.27 |
| UNIV MELBOURNE | 35 | 0.27 |
| UNIV ATHENS | 35 | 0.27 |
| NIIGATA UNIV | 35 | 0.27 |
| CENT S UNIV | 35 | 0.27 |
| UNIV TEXAS | 34 | 0.262 |
| UNIV MED CTR | 34 | 0.262 |
| UNIV BRITISH COLUMBIA | 34 | 0.262 |
| TIANJIN MED UNIV | 34 | 0.262 |
| GOLESTAN UNIV MED SCI | 34 | 0.262 |
| CITY HOPE NATL MED CTR | 34 | 0.262 |
| WUHAN UNIV | 33 | 0.254 |
| UNIV COLORADO | 33 | 0.254 |
| OSAKA CITY UNIV | 33 | 0.254 |
| DOKKYO MED UNIV | 33 | 0.254 |
| AKITA UNIV | 33 | 0.254 |
| UNIV SAO PAULO | 32 | 0.247 |
| UCL | 32 | 0.247 |
| TOKYO WOMENS MED UNIV | 32 | 0.247 |
| SAITAMA MED UNIV | 32 | 0.247 |
| QUEENS UNIV BELFAST | 32 | 0.247 |
| MCGILL UNIV | 32 | 0.247 |
| H LEE MOFFITT CANC CTR RES INST | 32 | 0.247 |
| TRINITY COLL DUBLIN | 31 | 0.239 |
| SHANDONG ACAD MED SCI | 31 | 0.239 |
| PETER MACCALLUM CANC CTR | 31 | 0.239 |
| NAGOYA CITY UNIV | 31 | 0.239 |
| CHARITE | 31 | 0.239 |
| UNIV GLASGOW | 30 | 0.231 |
| MED COLL WISCONSIN | 30 | 0.231 |
| INT PREVENT RES INST | 30 | 0.231 |
| FLINDERS UNIV S AUSTRALIA | 30 | 0.231 |
| UNIV TEXAS SW MED CTR DALLAS | 29 | 0.223 |
| UNIV HELSINKI | 29 | 0.223 |
| QINGDAO UNIV | 29 | 0.223 |
| JUNTENDO UNIV | 29 | 0.223 |
| AICHI CANC CTR | 29 | 0.223 |
| WAYNE STATE UNIV | 28 | 0.216 |
| UNIV MIAMI | 28 | 0.216 |
| UNIV ILLINOIS | 28 | 0.216 |
| UNIV BRISTOL | 28 | 0.216 |
| UNIV ADELAIDE | 28 | 0.216 |
| QUEENSLAND INST MED RES | 28 | 0.216 |
| MIE UNIV | 28 | 0.216 |
| CHINA MED UNIV HOSP | 28 | 0.216 |
| UNIV WISCONSIN | 27 | 0.208 |
| UNIV NAPLES 2 | 27 | 0.208 |
| UNIV LEEDS | 27 | 0.208 |
| UNIV FLORIDA | 27 | 0.208 |
| TOKYO UNIV HOSP | 27 | 0.208 |
| NATL HLTH RES INST | 27 | 0.208 |
| NANJING UNIV | 27 | 0.208 |
| CLEVELAND CLIN FDN | 27 | 0.208 |
| CHIBA CANC CTR | 27 | 0.208 |
| CATHOLIC UNIV KOREA | 27 | 0.208 |
| CATHARINA HOSP | 27 | 0.208 |
| ATATURK UNIV | 27 | 0.208 |
| UNIV ZURICH HOSP | 26 | 0.2 |
| UNIV UTAH | 26 | 0.2 |
| UNIV LOUISVILLE | 26 | 0.2 |
| UNIV KANSAS | 26 | 0.2 |
| SANJAY GANDHI POSTGRAD INST MED SCI | 26 | 0.2 |
| MED UNIV S CAROLINA | 26 | 0.2 |
| KAROLINSKA UNIV HOSP | 26 | 0.2 |
| JILIN UNIV | 26 | 0.2 |
| XIAMEN UNIV | 25 | 0.193 |
| WEILL CORNELL MED COLL | 25 | 0.193 |
| UNIV CALIF SAN FRANCISCO | 25 | 0.193 |
| UNIV ARIZONA | 25 | 0.193 |
| UNIV ALBERTA | 25 | 0.193 |
| TOKYO MED UNIV | 25 | 0.193 |
| TATA MEM HOSP | 25 | 0.193 |
| TAIPEI MED UNIV | 25 | 0.193 |
| RUHR UNIV BOCHUM | 25 | 0.193 |
| OITA UNIV | 25 | 0.193 |
| KITASATO UNIV | 25 | 0.193 |
| KAOHSIUNG MED UNIV HOSP | 25 | 0.193 |
| IRCCS | 25 | 0.193 |
| ERASMUS UNIV | 25 | 0.193 |
| CORNELL UNIV | 25 | 0.193 |
| UNIV BERN | 24 | 0.185 |
| TONGJI UNIV | 24 | 0.185 |
| SOUTHERN MED UNIV | 24 | 0.185 |
| SHOWA UNIV | 24 | 0.185 |
| SHIHEZI UNIV | 24 | 0.185 |
| SHAHID BEHESHTI UNIV MED SCI | 24 | 0.185 |
| SAPPORO MED UNIV | 24 | 0.185 |
| PRINCESS MARGARET HOSP | 24 | 0.185 |
| NATL HOSP ORG | 24 | 0.185 |
| NANTONG UNIV | 24 | 0.185 |
| LUND UNIV | 24 | 0.185 |
| LEIDEN UNIV | 24 | 0.185 |
| KEIYUKAI SAPPORO HOSP | 24 | 0.185 |
| JINAN UNIV | 24 | 0.185 |
| INDIANA UNIV | 24 | 0.185 |
| I SHOU UNIV | 24 | 0.185 |
| HYOGO COLL MED | 24 | 0.185 |
| HARBIN MED UNIV | 24 | 0.185 |
| CHONGQING MED UNIV | 24 | 0.185 |
| ANTONI VAN LEEUWENHOEK HOSP | 24 | 0.185 |
| ACAD MED CTR | 24 | 0.185 |
| WENZHOU MED UNIV | 23 | 0.177 |
| VENETO INST ONCOL IOV IRCCS | 23 | 0.177 |
| UNIV TEXAS HOUSTON | 23 | 0.177 |
| KAWASAKI MED UNIV | 23 | 0.177 |
| GUANGXI MED UNIV | 23 | 0.177 |
| FAR EASTERN MEM HOSP | 23 | 0.177 |
| BOSTON UNIV | 23 | 0.177 |
| UNIV VIRGINIA | 22 | 0.17 |
| UNIV TSUKUBA | 22 | 0.17 |
| UNIV NAPLES FEDERICO II | 22 | 0.17 |
| UNIV MUNICH | 22 | 0.17 |
| UNIV MED CTR GRONINGEN | 22 | 0.17 |
| TOYAMA UNIV | 22 | 0.17 |
| TOHO UNIV | 22 | 0.17 |
| SHANDONG CANC HOSP | 22 | 0.17 |
| SEOUL NATL UNIV HOSP | 22 | 0.17 |
| RADBOUD UNIV NIJMEGEN | 22 | 0.17 |
| NYU | 22 | 0.17 |
| MAASTRICHT UNIV | 22 | 0.17 |
| IST RIC FARMACOL MARIO NEGRI | 22 | 0.17 |
| INFORMAT MANAGEMENT SERV INC | 22 | 0.17 |
| CHARLES UNIV PRAGUE | 22 | 0.17 |
| CANC INST HOSP | 22 | 0.17 |
| BRITISH COLUMBIA CANC AGCY | 22 | 0.17 |
| UNIV NEW S WALES | 21 | 0.162 |
| SUZHOU UNIV | 21 | 0.162 |
| ST THOMAS HOSP | 21 | 0.162 |
| MACKAY MEM HOSP | 21 | 0.162 |
| KLINIKEN ESSEN MITTE | 21 | 0.162 |
| YAMAGATA UNIV | 20 | 0.154 |
| VRIJE UNIV AMSTERDAM | 20 | 0.154 |
| VIRGINIA COMMONWEALTH UNIV | 20 | 0.154 |
| UNIV SASKATCHEWAN | 20 | 0.154 |
| UNIV CAPE TOWN | 20 | 0.154 |
| SUNY BUFFALO | 20 | 0.154 |
| ROYAL MARSDEN NHS FDN TRUST | 20 | 0.154 |
| OSAKA MED COLL | 20 | 0.154 |
| ODENSE UNIV HOSP | 20 | 0.154 |
| NIPPON MED SCH | 20 | 0.154 |
| NATL UNIV SINGAPORE | 20 | 0.154 |
| NATL INST PUBL HLTH | 20 | 0.154 |
| NATL CHENG KUNG UNIV | 20 | 0.154 |
| NARA MED UNIV | 20 | 0.154 |
| KOREA UNIV | 20 | 0.154 |
| HSK WIESBADEN | 20 | 0.154 |
| WAKAYAMA MED UNIV | 19 | 0.146 |
| UNIV WALES HOSP | 19 | 0.146 |
| UNIV TROMSO | 19 | 0.146 |
| UNIV RYUKYUS | 19 | 0.146 |
| UNIV HOSP COLOGNE | 19 | 0.146 |
| UNIV BIRMINGHAM | 19 | 0.146 |
| TEXAS A M UNIV | 19 | 0.146 |
| SHERIKASHMIR INST MED SCI | 19 | 0.146 |
| SAITAMA CANC CTR | 19 | 0.146 |
| RUTGERS STATE UNIV | 19 | 0.146 |
| OSAKA CITY GEN HOSP | 19 | 0.146 |
| JICHI MED UNIV | 19 | 0.146 |
| INST GUSTAVE ROUSSY | 19 | 0.146 |
| GUANGZHOU MED UNIV | 19 | 0.146 |
| FDN IRCCS IST NAZL TUMORI | 19 | 0.146 |
| AARHUS UNIV HOSP | 19 | 0.146 |
| UNIV YAMANASHI | 18 | 0.139 |
| UNIV MUNSTER | 18 | 0.139 |
| UNIV ALABAMA BIRMINGHAM | 18 | 0.139 |
| TORONTO GEN HOSP | 18 | 0.139 |
| JOHNS HOPKINS UNIV HOSP | 18 | 0.139 |
| ICAHN SCH MED MT SINAI | 18 | 0.139 |
| CREIGHTON UNIV | 18 | 0.139 |
| VET AFFAIRS MED CTR | 17 | 0.131 |
| UNIV MED CTR ROTTERDAM | 17 | 0.131 |
| UNIV MANCHESTER | 17 | 0.131 |
| UNIV HOSP LEUVEN | 17 | 0.131 |
| UNIV DUBLIN TRINITY COLL | 17 | 0.131 |
| UNIV ABERDEEN | 17 | 0.131 |
| SIDNEY KIMMEL COMPREHENS CANC CTR JOHNS HOPKINS | 17 | 0.131 |
| SHIZUOKA CANC CTR HOSP | 17 | 0.131 |
| SHIGA UNIV MED SCI | 17 | 0.131 |
| NEW YORK PRESBYTERIAN HOSP | 17 | 0.131 |
| NATL CANC INST | 17 | 0.131 |
| MRC | 17 | 0.131 |
| MED UNIV GRAZ | 17 | 0.131 |
| MCMASTER UNIV | 17 | 0.131 |
| MASHHAD UNIV MED SCI | 17 | 0.131 |
| KLINIKUM BRAUNSCHWEIG | 17 | 0.131 |
| JIKEI UNIV | 17 | 0.131 |
| HOSP UNIV PENN | 17 | 0.131 |
| HENAN UNIV SCI TECHNOL | 17 | 0.131 |
| CHONNAM NATL UNIV | 17 | 0.131 |
| BETH ISRAEL DEACONESS MED CTR | 17 | 0.131 |
| VET AFFAIRS BOSTON HEALTHCARE SYST | 16 | 0.123 |
| UNIV TUBINGEN | 16 | 0.123 |
| UNIV TOKUSHIMA | 16 | 0.123 |
| UNIV PORTO | 16 | 0.123 |
| UNIV FED RIO GRANDE DO SUL | 16 | 0.123 |
| UNIV COPENHAGEN | 16 | 0.123 |
| UMEA UNIV | 16 | 0.123 |
| SO MED UNIV | 16 | 0.123 |
| PRINCESS ALEXANDRA HOSP | 16 | 0.123 |
| NATL DEF MED COLL | 16 | 0.123 |
| MONASH UNIV | 16 | 0.123 |
| KLINIKUM BAYREUTH | 16 | 0.123 |
| KATHOLIEKE UNIV LEUVEN | 16 | 0.123 |
| KANAZAWA UNIV | 16 | 0.123 |
| KAISER PERMANENTE | 16 | 0.123 |
| ISTANBUL UNIV | 16 | 0.123 |
| DANISH CANC SOC | 16 | 0.123 |
| AMER CANC SOC | 16 | 0.123 |
| ALL INDIA INST MED SCI | 16 | 0.123 |
| ACAD SINICA | 16 | 0.123 |
| VELINDRE HOSP | 15 | 0.116 |
| VANDERBILT INGRAM CANC CTR | 15 | 0.116 |
| UNIV TURIN | 15 | 0.116 |
| UNIV SOUTHAMPTON | 15 | 0.116 |
| UNIV MISSOURI | 15 | 0.116 |
| UNIV KASHMIR | 15 | 0.116 |
| UNIV HOSP GENEVA | 15 | 0.116 |
| UNIV HOSP GASTHUISBERG | 15 | 0.116 |
| UNIV HOSP CASE MED CTR | 15 | 0.116 |
| TOKYO METROPOLITAN INST GERONTOL | 15 | 0.116 |
| TECH UNIV DRESDEN | 15 | 0.116 |
| TABRIZ UNIV MED SCI | 15 | 0.116 |
| ST MARIANNA UNIV | 15 | 0.116 |
| ROYAL ADELAIDE HOSP | 15 | 0.116 |
| PUSAN NATL UNIV | 15 | 0.116 |
| NIHON UNIV | 15 | 0.116 |
| LANZHOU UNIV | 15 | 0.116 |
| KAOHSIUNG VET GEN HOSP | 15 | 0.116 |
| HYOGO CANC CTR | 15 | 0.116 |
| HANNOVER MED SCH | 15 | 0.116 |
| CANC REGISTRY NORWAY | 15 | 0.116 |
| ADDENBROOKES HOSP | 15 | 0.116 |
| WAKE FOREST UNIV | 14 | 0.108 |
| UNIV WURZBURG | 14 | 0.108 |
| UNIV TENNESSEE | 14 | 0.108 |
| UNIV OSLO | 14 | 0.108 |
| UNIV HOSP CLAUDE HURIEZ | 14 | 0.108 |
| UNIV HLTH NETWORK | 14 | 0.108 |
| UNIV ERLANGEN NURNBERG | 14 | 0.108 |
| SHENZHEN UNIV | 14 | 0.108 |
| QUEENS UNIV | 14 | 0.108 |
| MED UNIV BIALYSTOK | 14 | 0.108 |
| KRANKENHAUS NW FRANKFURT | 14 | 0.108 |
| IWATE MED UNIV | 14 | 0.108 |
| INDIANA UNIV SCH MED | 14 | 0.108 |
| FU JEN CATHOLIC UNIV | 14 | 0.108 |
| CITY HOPE COMPREHENS CANC CTR | 14 | 0.108 |
| ALBERT EINSTEIN COLL MED | 14 | 0.108 |
| UNIV WESTERN ONTARIO | 13 | 0.1 |
| UNIV STELLENBOSCH | 13 | 0.1 |
| UNIV SIENA | 13 | 0.1 |
| UNIV OCCUPAT ENVIRONM HLTH | 13 | 0.1 |
| UNIV NAPLES FEDERICO 2 | 13 | 0.1 |
| UNIV LAUSANNE HOSP | 13 | 0.1 |
| UNIV KENTUCKY | 13 | 0.1 |
| UNIV HOSP SCHLESWIG HOLSTEIN | 13 | 0.1 |
| UNIV CALGARY | 13 | 0.1 |
| UNIV BOLOGNA | 13 | 0.1 |
| TEXAS TECH UNIV | 13 | 0.1 |
| TAICHUNG VET GEN HOSP | 13 | 0.1 |
| SUNY STONY BROOK | 13 | 0.1 |
| SUNNYBROOK HLTH SCI CTR | 13 | 0.1 |
| STATE KEY LAB ONCOL SOUTH CHINA | 13 | 0.1 |
| ST MICHAELS HOSP | 13 | 0.1 |
| SHINSHU UNIV | 13 | 0.1 |
| OSAKA NATL HOSP | 13 | 0.1 |
| NIIGATA CANC CTR HOSP | 13 | 0.1 |
| NETHERLANDS CANC INST | 13 | 0.1 |
| NANJING DRUM TOWER HOSP | 13 | 0.1 |
| NANCHANG UNIV | 13 | 0.1 |
| MED UNIV | 13 | 0.1 |
| LOYOLA UNIV | 13 | 0.1 |
| KYOTO UNIV HOSP | 13 | 0.1 |
| JOHNS HOPKINS MED INST | 13 | 0.1 |
| INST CANC RES | 13 | 0.1 |
| GUANGDONG ESOPHAGEAL CANC INST | 13 | 0.1 |
| FUJITA HLTH UNIV | 13 | 0.1 |
| DARTMOUTH HITCHCOCK MED CTR | 13 | 0.1 |
| CTR RIFERIMENTO ONCOL | 13 | 0.1 |
| CTR OSCAR LAMBRET | 13 | 0.1 |
| CHANGHUA CHRISTIAN HOSP | 13 | 0.1 |
| CATHOLIC UNIV LOUVAIN | 13 | 0.1 |
| ARDABIL UNIV MED SCI | 13 | 0.1 |
| AJOU UNIV | 13 | 0.1 |
| ZHEJIANG PROV CANC HOSP | 12 | 0.092 |
| WENZHOU MED COLL | 12 | 0.092 |
| UNIV ZURICH | 12 | 0.092 |
| UNIV OTTAWA | 12 | 0.092 |
| UNIV MAGDEBURG | 12 | 0.092 |
| UNIV LEIPZIG | 12 | 0.092 |
| UNIV KLINIKUM LEIPZIG | 12 | 0.092 |
| UNIV KLINIKUM HEIDELBERG | 12 | 0.092 |
| UNIV IOWA | 12 | 0.092 |
| UNIV HOSP BRISTOL NHS FDN TRUST | 12 | 0.092 |
| UNIV BERGEN | 12 | 0.092 |
| UNIV BASEL HOSP | 12 | 0.092 |
| TOHOKU UNIV HOSP | 12 | 0.092 |
| TEIKYO UNIV | 12 | 0.092 |
| ROUEN UNIV HOSP | 12 | 0.092 |
| OSLO UNIV HOSP | 12 | 0.092 |
| MAYO CLIN ARIZONA | 12 | 0.092 |
| KOMAGOME HOSP | 12 | 0.092 |
| HIROSHIMA UNIV HOSP | 12 | 0.092 |
| HARVARD MED SCH | 12 | 0.092 |
| GUYS ST THOMAS NHS FDN TRUST | 12 | 0.092 |
| FUKUOKA UNIV | 12 | 0.092 |
| ELI LILLY CO | 12 | 0.092 |
| CHARITE UNIV MED BERLIN | 12 | 0.092 |
| BENGBU MED COLL | 12 | 0.092 |
| ASAN MED CTR | 12 | 0.092 |
| AMGEN INC | 12 | 0.092 |
| VRIJE UNIV AMSTERDAM MED CTR | 11 | 0.085 |
| UNIV REGENSBURG | 11 | 0.085 |
| UNIV PARIS 11 | 11 | 0.085 |
| UNIV NEBRASKA | 11 | 0.085 |
| UNIV MILANO BICOCCA | 11 | 0.085 |
| UNIV JOHANNESBURG | 11 | 0.085 |
| UNIV HOSP HAMBURG EPPENDORF | 11 | 0.085 |
| UNIV HOSP ESSEN | 11 | 0.085 |
| UNIV FED SAO PAULO | 11 | 0.085 |
| UNIV FED SANTA MARIA | 11 | 0.085 |
| UNIV CATTOLICA SACRO CUORE | 11 | 0.085 |
| UNIV CALIF BERKELEY | 11 | 0.085 |
| UNIV AUTONOMA BARCELONA | 11 | 0.085 |
| UNIV ALABAMA | 11 | 0.085 |
| TEL AVIV UNIV | 11 | 0.085 |
| SHIMANE UNIV | 11 | 0.085 |
| SHANXI CANC HOSP | 11 | 0.085 |
| SAHLGRENS UNIV HOSP | 11 | 0.085 |
| RABIN MED CTR | 11 | 0.085 |
| QUEEN ELIZABETH HOSP | 11 | 0.085 |
| PUSAN NATL UNIV HOSP | 11 | 0.085 |
| NORTH FRANCE UNIV | 11 | 0.085 |
| MT SINAI MED CTR | 11 | 0.085 |
| MT SINAI HOSP | 11 | 0.085 |
| MICHAEL E DEBAKEY VA MED CTR | 11 | 0.085 |
| MED UNIV INNSBRUCK | 11 | 0.085 |
| LOMA LINDA UNIV | 11 | 0.085 |
| KITANO HOSP | 11 | 0.085 |
| KANAZAWA UNIV HOSP | 11 | 0.085 |
| JIANGSU CANC HOSP | 11 | 0.085 |
| ICHIKAWA GEN HOSP | 11 | 0.085 |
| HAUKELAND HOSP | 11 | 0.085 |
| GUANGDONG PHARMACEUT UNIV | 11 | 0.085 |
| GEN HOSP | 11 | 0.085 |
| FUKUSHIMA MED UNIV | 11 | 0.085 |
| DALIAN MED UNIV | 11 | 0.085 |
| CAVALE BLANCHE UNIV HOSP | 11 | 0.085 |
| AIX MARSEILLE UNIV | 11 | 0.085 |
| WROCLAW MED UNIV | 10 | 0.077 |
| VELINDRE CANC CTR | 10 | 0.077 |
| UPPSALA UNIV | 10 | 0.077 |
| UNIV VERONA | 10 | 0.077 |
| UNIV VALENCIA | 10 | 0.077 |
| UNIV TEHRAN | 10 | 0.077 |
| UNIV ROUEN | 10 | 0.077 |
| UNIV ROMA LA SAPIENZA | 10 | 0.077 |
| UNIV PARIS 05 | 10 | 0.077 |
| UNIV MICHIGAN HLTH SYST | 10 | 0.077 |
| UNIV MED PHARM | 10 | 0.077 |
| UNIV MED DENT NEW JERSEY | 10 | 0.077 |
| UNIV MALAYA | 10 | 0.077 |
| UNIV LILLE NORD FRANCE | 10 | 0.077 |
| UNIV KLINIKUM HAMBURG EPPENDORF | 10 | 0.077 |
| UNIV CONNECTICUT | 10 | 0.077 |
| UNIV CALIF DAVIS | 10 | 0.077 |
| UNIV BRESCIA | 10 | 0.077 |
| UNIV BELGRADE | 10 | 0.077 |
| TOCHIGI CANC CTR | 10 | 0.077 |
| ST VINCENTS HOSP | 10 | 0.077 |
| ST MARYS HOSP | 10 | 0.077 |
| ST JAMES UNIV HOSP | 10 | 0.077 |
| ST ANTONIUS HOSP | 10 | 0.077 |
| SIRIC ONCOLILLE | 10 | 0.077 |
| SICHUAN CANC HOSP | 10 | 0.077 |
| SHIRAZ UNIV MED SCI | 10 | 0.077 |
| SEMMELWEIS UNIV | 10 | 0.077 |
| SANJAY GANDHI POST GRAD INST MED SCI | 10 | 0.077 |
| SAKU GEN HOSP | 10 | 0.077 |
| SAGA UNIV | 10 | 0.077 |
| ROYAL VICTORIA INFIRM | 10 | 0.077 |
| PONTCHAILLOU UNIV HOSP | 10 | 0.077 |
| PLA | 10 | 0.077 |
| PALACKY UNIV | 10 | 0.077 |
| OKAYAMA UNIV HOSP | 10 | 0.077 |
| NORFOLK NORWICH UNIV HOSP | 10 | 0.077 |
| NAGASAKI UNIV HOSP | 10 | 0.077 |
| MONTEFIORE MED CTR | 10 | 0.077 |
| KEY LAB DIAG TREATMENT TECHNOL THORAC ONCOL | 10 | 0.077 |
| KATHOLIEKE UNIV LEUVEN HOSP | 10 | 0.077 |
| KAOHSIUNG CHANG GUNG MEM HOSP | 10 | 0.077 |
| JAGIELLONIAN UNIV | 10 | 0.077 |
| ISLAMIC AZAD UNIV | 10 | 0.077 |
| INT UNIV HLTH WELF | 10 | 0.077 |
| INST ONCOL | 10 | 0.077 |
| HOKKAIDO UNIV HOSP | 10 | 0.077 |
| GOVT MED COLL | 10 | 0.077 |
| FUJIAN NORMAL UNIV | 10 | 0.077 |
| CTR DIS CONTROL PREVENT | 10 | 0.077 |
| COPENHAGEN UNIV HOSP | 10 | 0.077 |
| CHUNG SHAN MED UNIV | 10 | 0.077 |
| CHONBUK NATL UNIV | 10 | 0.077 |
| CHINA JAPAN FRIENDSHIP HOSP | 10 | 0.077 |
| CATHOLIC UNIV | 10 | 0.077 |
| CANISIUS WILHELMINA HOSP | 10 | 0.077 |
| ANDALUSIAN SCH PUBL HLTH | 10 | 0.077 |
| YAMAGUCHI UNIV | 9 | 0.069 |
| WAGENINGEN UNIV | 9 | 0.069 |
| W VIRGINIA UNIV | 9 | 0.069 |
| US FDA | 9 | 0.069 |
| UNIV UTRECHT | 9 | 0.069 |
| UNIV SHEFFIELD | 9 | 0.069 |
| UNIV PISA | 9 | 0.069 |
| UNIV NEBRASKA MED CTR | 9 | 0.069 |
| UNIV MED MAINZ | 9 | 0.069 |
| UNIV JINAN | 9 | 0.069 |
| UNIV HOSP LILLE | 9 | 0.069 |
| UNIV HOSP BERN | 9 | 0.069 |
| UNIV GOTHENBURG | 9 | 0.069 |
| UNIV GHENT | 9 | 0.069 |
| UNIV ESTADUAL CAMPINAS | 9 | 0.069 |
| UNIV E ANGLIA | 9 | 0.069 |
| UNIV BONN | 9 | 0.069 |
| THOMAS JEFFERSON UNIV HOSP | 9 | 0.069 |
| TENWEK HOSP | 9 | 0.069 |
| SOONCHUNHYANG UNIV | 9 | 0.069 |
| SHIKOKU CANC CTR | 9 | 0.069 |
| SHANGHAI CANC INST | 9 | 0.069 |
| RUSSIAN ACAD MED SCI | 9 | 0.069 |
| RIGSHOSP | 9 | 0.069 |
| QUEEN MARY UNIV LONDON | 9 | 0.069 |
| PARACELSUS MED UNIV | 9 | 0.069 |
| ONTARIO CANC INST | 9 | 0.069 |
| NORTH SICHUAN MED COLL | 9 | 0.069 |
| NINGXIA MED UNIV | 9 | 0.069 |
| NEWCASTLE UNIV | 9 | 0.069 |
| NATL DEF MED CTR | 9 | 0.069 |
| NATL CTR GLOBAL HLTH MED | 9 | 0.069 |
| NANKAI UNIV | 9 | 0.069 |
| N CAROLINA CENT UNIV | 9 | 0.069 |
| MED COLL GEORGIA | 9 | 0.069 |
| LINZHOU CANC HOSP | 9 | 0.069 |
| KOCHI MED SCH | 9 | 0.069 |
| KLINIKUM NURNBERG | 9 | 0.069 |
| KLINIKUM AUGSBURG | 9 | 0.069 |
| KERMAN UNIV MED SCI | 9 | 0.069 |
| KANTONSSPITAL ST GALLEN | 9 | 0.069 |
| JOHNS HOPKINS SCH MED | 9 | 0.069 |
| JOHNS HOPKINS BLOOMBERG SCH PUBL HLTH | 9 | 0.069 |
| IRCCS IST RIC FARMACOL MARIO NEGRI | 9 | 0.069 |
| INDIAN COUNCIL MED RES | 9 | 0.069 |
| HOSP CLIN BARCELONA | 9 | 0.069 |
| HENAN UNIV | 9 | 0.069 |
| HELLEN HLTH FDN | 9 | 0.069 |
| HAMAMATSU UNIV SCH MED | 9 | 0.069 |
| GUNMA UNIV HOSP | 9 | 0.069 |
| GIFU UNIV | 9 | 0.069 |
| EHIME UNIV | 9 | 0.069 |
| CTR GEORGES FRANCOIS LECLERC | 9 | 0.069 |
| CTR FRANCOIS BACLESSE | 9 | 0.069 |
| CHINESE CTR DIS CONTROL PREVENT | 9 | 0.069 |
| CHANGZHI MED COLL | 9 | 0.069 |
| CARDIFF UNIV | 9 | 0.069 |
| BEIJING UNIV CHEM TECHNOL | 9 | 0.069 |
| ATRIUM MED CTR | 9 | 0.069 |
| ATATURK TRAINING RES HOSP CHEST DIS CHEST SUR | 9 | 0.069 |
| AARP | 9 | 0.069 |
| AARHUS UNIV | 9 | 0.069 |
| ZHEJIANG PROV PEOPLES HOSP | 8 | 0.062 |
| YEUNGNAM UNIV | 8 | 0.062 |
| YANGZHOU UNIV | 8 | 0.062 |
| WISTAR INST ANAT BIOL | 8 | 0.062 |
| WEILL CORNELL MED CTR | 8 | 0.062 |
| UNIV WITWATERSRAND | 8 | 0.062 |
| UNIV WESTERN SYDNEY | 8 | 0.062 |
| UNIV UDINE | 8 | 0.062 |
| UNIV SZEGED | 8 | 0.062 |
| UNIV OKLAHOMA | 8 | 0.062 |
| UNIV NEW MEXICO | 8 | 0.062 |
| UNIV MASSACHUSETTS | 8 | 0.062 |
| UNIV MANITOBA | 8 | 0.062 |
| UNIV LONDON | 8 | 0.062 |
| UNIV HOSP MOTOL | 8 | 0.062 |
| UNIV GEORGIA | 8 | 0.062 |
| UNIV FREIBURG KLINIKUM | 8 | 0.062 |
| UNIV ESTADO RIO DE JANEIRO | 8 | 0.062 |
| UNIV EDINBURGH | 8 | 0.062 |
| UNIV DUISBURG ESSEN | 8 | 0.062 |
| UNIV BUENOS AIRES | 8 | 0.062 |
| UCT UNIV CANC CTR | 8 | 0.062 |
| TSINGHUA UNIV | 8 | 0.062 |
| TIANJIN MED UNIV CANC INST HOSP | 8 | 0.062 |
| TECHNION ISRAEL INST TECHNOL | 8 | 0.062 |
| TARBIAT MODARES UNIV | 8 | 0.062 |
| ST ORSOLA MARCELLO MALPIGHI HOSP | 8 | 0.062 |
| ST GEORGE HOSP | 8 | 0.062 |
| SHANGHAI CHEST HOSP | 8 | 0.062 |
| SAKU CENT HOSP | 8 | 0.062 |
| RUSSIAN ACAD SCI | 8 | 0.062 |
| RTI INT | 8 | 0.062 |
| REGINA ELENA INST CANC RES | 8 | 0.062 |
| QIMR BERGHOFER MED RES INST | 8 | 0.062 |
| PURPAN UNIV HOSP | 8 | 0.062 |
| PRINCESS MARGARET CANC CTR | 8 | 0.062 |
| PEOPLES LIBERAT ARMY | 8 | 0.062 |
| PCL OSAKA INC | 8 | 0.062 |
| NAVARRE PUBL HLTH INST | 8 | 0.062 |
| NATL UNIV SINGAPORE HOSP | 8 | 0.062 |
| NATL INST PUBL HLTH ENVIRONM RIVM | 8 | 0.062 |
| NAGASAKI UNIV | 8 | 0.062 |
| MURCIA REG HLTH COUNCIL | 8 | 0.062 |
| MD ANDERSON CANC CTR | 8 | 0.062 |
| MAYO CLIN FLORIDA | 8 | 0.062 |
| MARMARA UNIV | 8 | 0.062 |
| M M MED BIOINFORMAT | 8 | 0.062 |
| LIAOCHENG PEOPLES HOSP | 8 | 0.062 |
| KUMAMOTO UNIV HOSP | 8 | 0.062 |
| KANSAI MED UNIV | 8 | 0.062 |
| KAISER PERMANENTE NO CALIF | 8 | 0.062 |
| JERSEY SHORE UNIV | 8 | 0.062 |
| IRAN UNIV MED SCI | 8 | 0.062 |
| INST NACL CANC | 8 | 0.062 |
| INST CURIE | 8 | 0.062 |
| HUTCHISON MRC RES CTR | 8 | 0.062 |
| HUBEI UNIV MED | 8 | 0.062 |
| HOSP GELDERSE VALLEI | 8 | 0.062 |
| HOSP CLIN PORTO ALEGRE | 8 | 0.062 |
| HOP TENON | 8 | 0.062 |
| HOP ST ANTOINE | 8 | 0.062 |
| HOP EUROPEEN GEORGES POMPIDOU | 8 | 0.062 |
| HENRY FORD HOSP | 8 | 0.062 |
| HENAN UNIV TCM | 8 | 0.062 |
| HENAN PROV PEOPLES HOSP | 8 | 0.062 |
| HAUT LEVEQUE UNIV HOSP | 8 | 0.062 |
| GUANGDONG GEN HOSP | 8 | 0.062 |
| GOETHE UNIV FRANKFURT | 8 | 0.062 |
| GERMAN INST HUMAN NUTR POTSDAM REHBRUCKE | 8 | 0.062 |
| GERMAN CANC CONSORTIUM DKTK | 8 | 0.062 |
| GEORGETOWN UNIV | 8 | 0.062 |
| ERASMUS MC UNIV MED CTR | 8 | 0.062 |
| DALHOUSIE UNIV | 8 | 0.062 |
| CROSS CANC INST | 8 | 0.062 |
| CROIX ROUSSE UNIV HOSP | 8 | 0.062 |
| COLLABORAT INNOVAT CTR CANC MED | 8 | 0.062 |
| CLAUDE HURIEZ UNIV HOSP | 8 | 0.062 |
| CIVILE MP AREZZO HOSP | 8 | 0.062 |
| CHU VAUDOIS | 8 | 0.062 |
| CHIBA UNIV HOSP | 8 | 0.062 |
| CATALAN INST ONCOL | 8 | 0.062 |
| CAROLINAS MED CTR | 8 | 0.062 |
| CAMS | 8 | 0.062 |
| ASTANA MED UNIV | 8 | 0.062 |
| AKITA UNIV HOSP | 8 | 0.062 |
| XINXIANG MED COLL | 7 | 0.054 |
| XINJIANG UNIV | 7 | 0.054 |
| UNIV VIRGINIA HLTH SYST | 7 | 0.054 |
| UNIV ULM | 7 | 0.054 |
| UNIV TEXAS HLTH SCI CTR SAN ANTONIO | 7 | 0.054 |
| UNIV SAINS MALAYSIA | 7 | 0.054 |
| UNIV S AUSTRALIA | 7 | 0.054 |
| UNIV PECS | 7 | 0.054 |
| UNIV PARIS 06 | 7 | 0.054 |
| UNIV NOTTINGHAM HOSP | 7 | 0.054 |
| UNIV NOTTINGHAM | 7 | 0.054 |
| UNIV MONTREAL | 7 | 0.054 |
| UNIV MED LEIPZIG | 7 | 0.054 |
| UNIV LIVERPOOL | 7 | 0.054 |
| UNIV LAUSANNE | 7 | 0.054 |
| UNIV KLINIKUM MUNSTER | 7 | 0.054 |
| UNIV HOSP CLEVELAND | 7 | 0.054 |
| UNIV HAMBURG | 7 | 0.054 |
| UNIV HALLE WITTENBERG | 7 | 0.054 |
| UNIV GOTTINGEN | 7 | 0.054 |
| UNIV FLORENCE | 7 | 0.054 |
| UNIV DELHI | 7 | 0.054 |
| UNIV CINCINNATI | 7 | 0.054 |
| UNIV BASEL | 7 | 0.054 |
| UNIV BARCELONA | 7 | 0.054 |
| TULANE UNIV | 7 | 0.054 |
| TUFTS UNIV | 7 | 0.054 |
| TANGSHAN PEOPLES HOSP | 7 | 0.054 |
| TAIPEI CITY HOSP | 7 | 0.054 |
| SW ONCOL GRP | 7 | 0.054 |
| SUNY DOWNSTATE MED CTR | 7 | 0.054 |
| ST LUKES INT HOSP | 7 | 0.054 |
| SHIGEI MED RES INST | 7 | 0.054 |
| SHANDONG TUMOR HOSP | 7 | 0.054 |
| SARAH CANNON RES INST | 7 | 0.054 |
| RICE UNIV | 7 | 0.054 |
| RESPONSE GENET INC | 7 | 0.054 |
| REG MED RES CTR | 7 | 0.054 |
| PENN STATE UNIV | 7 | 0.054 |
| PEKING UNIV CANC HOSP INST | 7 | 0.054 |
| OSAKA MED COLL HOSP | 7 | 0.054 |
| OHTA NISHINOUCHI HOSP | 7 | 0.054 |
| NATL INST RADIOL SCI | 7 | 0.054 |
| NATL INST PUBL HLTH ENVIRONM | 7 | 0.054 |
| NATL CHENG KUNG UNIV HOSP | 7 | 0.054 |
| NAGOYA UNIV HOSP | 7 | 0.054 |
| MINIST HLTH | 7 | 0.054 |
| MICHIGAN STATE UNIV | 7 | 0.054 |
| MERCY UNIV HOSP | 7 | 0.054 |
| MED UNIV XINJIANG | 7 | 0.054 |
| MED UNIV LUBECK | 7 | 0.054 |
| MAZANDARAN UNIV MED SCI | 7 | 0.054 |
| KYUNGPOOK NATL UNIV | 7 | 0.054 |
| KOBE UNIV HOSP | 7 | 0.054 |
| KLINIKUM NURNBERG NORD | 7 | 0.054 |
| KAWASAKI MUNICIPAL HOSP | 7 | 0.054 |
| KAOHSIUNG MUNICIPAL HSIAOKANG HOSP | 7 | 0.054 |
| KAGAWA UNIV | 7 | 0.054 |
| JOHNS HOPKINS BAYVIEW MED CTR | 7 | 0.054 |
| IST CLIN HUMANITAS | 7 | 0.054 |
| IMAM KHOMEINI HOSP | 7 | 0.054 |
| HUAIAN 2 HOSP | 7 | 0.054 |
| HOSP UNIV BELLVITGE | 7 | 0.054 |
| HOSP UNIV 12 OCTUBRE | 7 | 0.054 |
| HOP NORD MARSEILLE | 7 | 0.054 |
| HOP CAVALE BLANCHE | 7 | 0.054 |
| HONG KONG POLYTECH UNIV | 7 | 0.054 |
| HIROSAKI UNIV | 7 | 0.054 |
| HENAN CANC HOSP | 7 | 0.054 |
| HANYANG UNIV | 7 | 0.054 |
| HALLYM UNIV | 7 | 0.054 |
| GUANGDONG MED COLL | 7 | 0.054 |
| GLASGOW ROYAL INFIRM | 7 | 0.054 |
| GENENTECH INC | 7 | 0.054 |
| ERASMUS MC UNIV MED CTR ROTTERDAM | 7 | 0.054 |
| DEPT VET AFFAIRS MED CTR | 7 | 0.054 |
| DEPT SURG | 7 | 0.054 |
| DANA FARBER BRIGHAM WOMENS CANC CTR | 7 | 0.054 |
| CTR EUGENE MARQUIS | 7 | 0.054 |
| CTR CANC | 7 | 0.054 |
| CTR ALEXIS VAUTRIN | 7 | 0.054 |
| CRO AVIANO | 7 | 0.054 |
| COLUMBIA UNIV COLL PHYS SURG | 7 | 0.054 |
| CLIN CTR SERBIA | 7 | 0.054 |
| CITY HOPE CANC CTR | 7 | 0.054 |
| CHURCHILL HOSP | 7 | 0.054 |
| CANC RES PREVENT INST ISPO | 7 | 0.054 |
| BELFAST HLTH SOCIAL CARE TRUST | 7 | 0.054 |
| BEIJING UNIV TECHNOL | 7 | 0.054 |
| BARNES JEWISH HOSP | 7 | 0.054 |
| ANYANG TUMOR HOSP | 7 | 0.054 |
| YAOCUN ESOPHAGEAL CANC HOSP | 6 | 0.046 |
| WEIFANG MED COLL | 6 | 0.046 |
| WALTER REED ARMY MED CTR | 6 | 0.046 |
| UNIV WISCONSIN HOSP CLIN | 6 | 0.046 |
| UNIV VIENNA | 6 | 0.046 |
| UNIV VERMONT | 6 | 0.046 |
| UNIV TEXAS HLTH SCI CTR HOUSTON | 6 | 0.046 |
| UNIV SO DENMARK | 6 | 0.046 |
| UNIV SCI TECHNOL CHINA | 6 | 0.046 |
| UNIV S CAROLINA | 6 | 0.046 |
| UNIV PERUGIA | 6 | 0.046 |
| UNIV PARMA | 6 | 0.046 |
| UNIV PALERMO | 6 | 0.046 |
| UNIV NEWCASTLE | 6 | 0.046 |
| UNIV LIBRE BRUXELLES | 6 | 0.046 |
| UNIV KLINIKUM WURZBURG | 6 | 0.046 |
| UNIV IOANNINA | 6 | 0.046 |
| UNIV HOSP SOUTHAMPTON NHS FDN TRUST | 6 | 0.046 |
| UNIV HOSP LEICESTER NHS TRUST | 6 | 0.046 |
| UNIV HOSP LEICESTER | 6 | 0.046 |
| UNIV HAWAII | 6 | 0.046 |
| UNIV GRONINGEN UNIV MED CTR GRONINGEN | 6 | 0.046 |
| UNIV FED RIO DE JANEIRO | 6 | 0.046 |
| UNIV COLORADO DENVER | 6 | 0.046 |
| UNIV COLL LONDON HOSP | 6 | 0.046 |
| UNIV CLIN LEIPZIG | 6 | 0.046 |
| UNIV CHICAGO MED | 6 | 0.046 |
| UNIV CANC CTR LEIPZIG | 6 | 0.046 |
| UNIFORMED SERV UNIV HLTH SCI | 6 | 0.046 |
| TOTTORI UNIV | 6 | 0.046 |
| TENRI HOSP | 6 | 0.046 |
| TENNESSEE ONCOL PLLC | 6 | 0.046 |
| SWEDISH CANC INST | 6 | 0.046 |
| STATE KEY LAB ONCOL S CHINA | 6 | 0.046 |
| ST ANTOINE UNIV HOSP | 6 | 0.046 |
| SHIZUOKA PREFECTURAL GEN HOSP | 6 | 0.046 |
| SHANTOU UNIV MED COLL | 6 | 0.046 |
| SHANGHAI UNIV TRADIT CHINESE MED | 6 | 0.046 |
| SHAANXI NORMAL UNIV | 6 | 0.046 |
| SA PATHOL | 6 | 0.046 |
| ROYAL VICTORIA HOSP | 6 | 0.046 |
| ROYAL BRISBANE HOSP | 6 | 0.046 |
| RHODE ISL HOSP | 6 | 0.046 |
| RED CROSS HOSP | 6 | 0.046 |
| PEOPLES HOSP ZHENGZHOU | 6 | 0.046 |
| OULU UNIV HOSP | 6 | 0.046 |
| OSAKA GEN MED CTR | 6 | 0.046 |
| OREGON CLIN | 6 | 0.046 |
| NATL INST CANC RES | 6 | 0.046 |
| NATL CANC CTR HOSP | 6 | 0.046 |
| MED UNIV LODZ | 6 | 0.046 |
| MED SPECTRUM TWENTE | 6 | 0.046 |
| MASARYK UNIV | 6 | 0.046 |
| LIAONING MED UNIV | 6 | 0.046 |
| KYUNG HEE UNIV | 6 | 0.046 |
| KURDISTAN UNIV MED SCI | 6 | 0.046 |
| KOREA CANC CTR HOSP | 6 | 0.046 |
| KOO FDN SUN YAT SEN CANC CTR | 6 | 0.046 |
| KONKUK UNIV | 6 | 0.046 |
| KOCHI HLTH SCI CTR | 6 | 0.046 |
| KLINIKUM WOLFSBURG | 6 | 0.046 |
| KEIMYUNG UNIV | 6 | 0.046 |
| KAPLAN MED CTR | 6 | 0.046 |
| KANSAI ROSAI HOSP | 6 | 0.046 |
| JOHNS HOPKINS | 6 | 0.046 |
| JIANGSU PROV CTR DIS CONTROL PREVENT | 6 | 0.046 |
| JIANGNAN UNIV | 6 | 0.046 |
| ISHIKAWA PREFECTURAL CENT HOSP | 6 | 0.046 |
| ISFAHAN UNIV MED SCI | 6 | 0.046 |
| INST PATHOL | 6 | 0.046 |
| INST CANC MONTPELLIER | 6 | 0.046 |
| IMPERIAL COLL LONDON | 6 | 0.046 |
| IARC | 6 | 0.046 |
| HUMBOLDT UNIV | 6 | 0.046 |
| HUAIAN CTR DIS CONTROL PREVENT | 6 | 0.046 |
| HOSP BADALONA GERMANS TRIAS PUJOL | 6 | 0.046 |
| HOP EDOUARD HERRIOT | 6 | 0.046 |
| HARBIN MED COLL | 6 | 0.046 |
| HACETTEPE UNIV | 6 | 0.046 |
| GURU GOBIND SINGH INDRAPRASTHA UNIV | 6 | 0.046 |
| GUNMA PREFECTURAL CANC CTR | 6 | 0.046 |
| GUANGDONG ESOPHAGEAL CANC RES INST | 6 | 0.046 |
| GLOUCESTERSHIRE ROYAL HOSP | 6 | 0.046 |
| GHENT UNIV HOSP | 6 | 0.046 |
| GAZI UNIV | 6 | 0.046 |
| GANYU CTR DIS CONTROL PREVENT | 6 | 0.046 |
| FUNDACAO OSWALDO CRUZ | 6 | 0.046 |
| FLINDERS MED CTR | 6 | 0.046 |
| EVANGEL KRANKENHAUS | 6 | 0.046 |
| EDOUARD HERRIOT UNIV HOSP | 6 | 0.046 |
| E CAROLINA UNIV | 6 | 0.046 |
| DUKE CANC INST | 6 | 0.046 |
| DALLAS VA MED CTR | 6 | 0.046 |
| DAFENG CTR DIS CONTROL PREVENT | 6 | 0.046 |
| CTR JEAN PERRIN | 6 | 0.046 |
| CTR HENRI BECQUEREL | 6 | 0.046 |
| CIBERESP | 6 | 0.046 |
| CHULALONGKORN UNIV | 6 | 0.046 |
| CHRU | 6 | 0.046 |
| CHI MEI MED CTR | 6 | 0.046 |
| CASTLE HILL HOSP | 6 | 0.046 |
| CANC TREATMENT CTR AMER | 6 | 0.046 |
| BC CANC AGCY | 6 | 0.046 |
| AZIENDA OSPED PADOVA | 6 | 0.046 |
| AZIENDA OSPED | 6 | 0.046 |
| AUSTRALIAN NATL UNIV | 6 | 0.046 |
| ANTICANCER INC | 6 | 0.046 |
| ANKARA NUMUNE TRAINING RES HOSP | 6 | 0.046 |
| AALBORG UNIV HOSP | 6 | 0.046 |
| ZHEJIANG KEY LAB DIAG TREATMENT TECHNOL THORAC | 5 | 0.039 |
| YILDIRIM BEYAZIT UNIV | 5 | 0.039 |
| YANTING CANC RES INST | 5 | 0.039 |
| XUZHOU MED COLL | 5 | 0.039 |
| WILLIAM BEAUMONT HOSP | 5 | 0.039 |
| WEIFANG PEOPLES HOSP | 5 | 0.039 |
| WANNAN MED COLL | 5 | 0.039 |
| VENETO ONCOL INST IOV IRCCS | 5 | 0.039 |
| VAN ANDEL RES INST | 5 | 0.039 |
| VA PALO ALTO HLTH CARE SYST | 5 | 0.039 |
| VA MED CTR | 5 | 0.039 |
| UNIV TWENTE | 5 | 0.039 |
| UNIV TRIESTE | 5 | 0.039 |
| UNIV TEXAS MD ANDERSON HOSP | 5 | 0.039 |
| UNIV TAMPERE | 5 | 0.039 |
| UNIV SAARLAND | 5 | 0.039 |
| UNIV OULU | 5 | 0.039 |
| UNIV MED MANNHEIM | 5 | 0.039 |
| UNIV MED CTR MANNHEIM | 5 | 0.039 |
| UNIV MED CTR MAINZ | 5 | 0.039 |
| UNIV MED BERLIN | 5 | 0.039 |
| UNIV MAINZ | 5 | 0.039 |
| UNIV LUBECK | 5 | 0.039 |
| UNIV LILLE 2 | 5 | 0.039 |
| UNIV KWAZULU NATAL | 5 | 0.039 |
| UNIV KLINIKUM CARL GUSTAV CARUS | 5 | 0.039 |
| UNIV KLIN KOLN | 5 | 0.039 |
| UNIV KHARTOUM | 5 | 0.039 |
| UNIV IOWA HOSP CLIN | 5 | 0.039 |
| UNIV GENOA | 5 | 0.039 |
| UNIV FED MINAS GERAIS | 5 | 0.039 |
| UNIV DUNDEE | 5 | 0.039 |
| UNIV COLL HOSP GALWAY | 5 | 0.039 |
| UNIV COLL DUBLIN | 5 | 0.039 |
| UNIV BOURGOGNE | 5 | 0.039 |
| UNIV ARKANSAS MED SCI | 5 | 0.039 |
| UNIV AARHUS | 5 | 0.039 |
| UNIKLIN KOLN | 5 | 0.039 |
| TZU CHI UNIV | 5 | 0.039 |
| TRISERV GEN HOSP | 5 | 0.039 |
| TRI SERV GEN HOSP | 5 | 0.039 |
| TOKYO UNIV SCI | 5 | 0.039 |
| TOKYO METROPOLITAN KOMAGOME HOSP | 5 | 0.039 |
| TEMPLE UNIV | 5 | 0.039 |
| TAIAN CENT HOSP | 5 | 0.039 |
| SZENT ISTVAN UNIV | 5 | 0.039 |
| ST VINCENT HOSP | 5 | 0.039 |
| ST JOSEPHS HOSP | 5 | 0.039 |
| SOCIAL INSURANCE YOKOHAMA CENT HOSP | 5 | 0.039 |
| SK INST MED SCI | 5 | 0.039 |
| SHANXI MED UNIV | 5 | 0.039 |
| SHANDONG TUMOR HOSP INST | 5 | 0.039 |
| SCH MED | 5 | 0.039 |
| SAMFUNDET FOLKHALSAN | 5 | 0.039 |
| SAKAI MUNICIPAL HOSP | 5 | 0.039 |
| RUSH UNIV | 5 | 0.039 |
| ROYAL SURREY CTY HOSP | 5 | 0.039 |
| ROYAL PRINCE ALFRED HOSP | 5 | 0.039 |
| ROYAL N SHORE HOSP | 5 | 0.039 |
| ROYAL INFIRM | 5 | 0.039 |
| ROYAL GWENT HOSP | 5 | 0.039 |
| ROYAL GLAMORGAN HOSP | 5 | 0.039 |
| ROMED KLINIKUM ROSENHEIM | 5 | 0.039 |
| QUEEN ALEXANDRA HOSP | 5 | 0.039 |
| PRINCE SONGKLA UNIV | 5 | 0.039 |
| POSTGRAD INST MED EDUC RES | 5 | 0.039 |
| PONTIFICIA UNIV CATOLICA RIO GRANDE DO SUL | 5 | 0.039 |
| PEOPLES HOSP XISHUANGBANNA DAI AUTONOMOUS PREFECT | 5 | 0.039 |
| PARIS DESCARTES UNIV | 5 | 0.039 |
| PADJADJARAN STATE UNIV | 5 | 0.039 |
| OXFORD UNIV HOSP NHS TRUST | 5 | 0.039 |
| OTTAWA HOSP | 5 | 0.039 |
| OTSUKA PHARMACEUT CO LTD | 5 | 0.039 |
| OSAKA UNIV HOSP | 5 | 0.039 |
| NW MEM HOSP | 5 | 0.039 |
| NTT MED CTR TOKYO | 5 | 0.039 |
| NORWEGIAN UNIV SCI TECHNOL | 5 | 0.039 |
| NORTH WALES CANC TREATMENT CTR | 5 | 0.039 |
| NATL CTR TUMOR DIS | 5 | 0.039 |
| NATL CHUNG HSING UNIV | 5 | 0.039 |
| NATL CARDIOVASC CTR | 5 | 0.039 |
| NANTONG TUMOR HOSP | 5 | 0.039 |
| MUENSTER UNIV HOSP | 5 | 0.039 |
| MIL GEN HOSP BEIJING PLA | 5 | 0.039 |
| MAYO CLIN MAYO FDN | 5 | 0.039 |
| MALMO UNIV HOSP | 5 | 0.039 |
| MAIMONIDES HOSP | 5 | 0.039 |
| MACKAY MED COLL | 5 | 0.039 |
| M SKLODOWSKA CURIE MEM CANC CTR | 5 | 0.039 |
| LOUISIANA STATE UNIV | 5 | 0.039 |
| LILLE UNIV HOSP | 5 | 0.039 |
| KYUSHU CANC CTR | 5 | 0.039 |
| KUNMING MED UNIV | 5 | 0.039 |
| KRANKENHAUS BARMHERZIGEN SCHWESTERN LINZ | 5 | 0.039 |
| KOBE CITY MED CTR GEN HOSP | 5 | 0.039 |
| KING FAISAL SPECIALIST HOSP RES CTR | 5 | 0.039 |
| KAZAKH RES INST ONCOL RADIOL | 5 | 0.039 |
| KAWASAKI UNIV MED WELF | 5 | 0.039 |
| KANAGAWA CANC CTR HOSP | 5 | 0.039 |
| KAMINENI HOSP | 5 | 0.039 |
| JPARC | 5 | 0.039 |
| JOHANNES GUTENBERG UNIV HOSP | 5 | 0.039 |
| JIAMUSI UNIV | 5 | 0.039 |
| JEAN PIERRE AUBERT RES CTR | 5 | 0.039 |
| INT MED CTR JAPAN | 5 | 0.039 |
| INST CYTOL PREVENT ONCOL ICMR | 5 | 0.039 |
| INNER MONGOLIA UNIV | 5 | 0.039 |
| HUGEF FDN | 5 | 0.039 |
| HOSP SAO JOAO | 5 | 0.039 |
| HOSP SANTA CREU SANT PAU | 5 | 0.039 |
| HOSP AC CAMARGO FUND ANTONIO PRUDENTE | 5 | 0.039 |
| HOP LOUIS PASTEUR | 5 | 0.039 |
| HOP COCHIN | 5 | 0.039 |
| HOP AMBROISE PARE | 5 | 0.039 |
| HONG KONG UNIV SCI TECHNOL | 5 | 0.039 |
| HENAN TUMOR HOSP | 5 | 0.039 |
| HENAN KEY LAB TUMOR EPIDEMIOL | 5 | 0.039 |
| HELMHOLTZ ZENTRUM MUNCHEN | 5 | 0.039 |
| HEBREW UNIV JERUSALEM | 5 | 0.039 |
| HARVARD RADIAT ONCOL PROGRAM | 5 | 0.039 |
| HAMMERSMITH HOSP | 5 | 0.039 |
| GYEONGSANG NATL UNIV | 5 | 0.039 |
| GUANGDONG ACAD MED SCI | 5 | 0.039 |
| GRIFFITH UNIV | 5 | 0.039 |
| GIFU GRAD SCH MED | 5 | 0.039 |
| GEORGIA REGENTS UNIV | 5 | 0.039 |
| GACHON UNIV | 5 | 0.039 |
| FUKUI PREFECTURAL HOSP | 5 | 0.039 |
| FIRST PEOPLES HOSP HUAIAN | 5 | 0.039 |
| FINNISH CANC REGISTRY | 5 | 0.039 |
| EULJI UNIV | 5 | 0.039 |
| ERASMUS MC UNIV | 5 | 0.039 |
| EMORY UNIV HOSP | 5 | 0.039 |
| EGE UNIV | 5 | 0.039 |
| E DA HOSP | 5 | 0.039 |
| DR B BOROOAH CANC INST | 5 | 0.039 |
| DONGHUA UNIV | 5 | 0.039 |
| DONGGUK UNIV | 5 | 0.039 |
| DKFZ | 5 | 0.039 |
| DEPT THORAC SURG | 5 | 0.039 |
| CHU PONTCHAILLOU | 5 | 0.039 |
| CHU MORVAN | 5 | 0.039 |
| CHU BREST | 5 | 0.039 |
| CHU ANGERS | 5 | 0.039 |
| CHRU LILLE | 5 | 0.039 |
| CHINESE PEOPLES LIBERAT ARMY | 5 | 0.039 |
| CHANG GUNG INST TECHNOL | 5 | 0.039 |
| CENT ASIA CANC INST | 5 | 0.039 |
| CEDARS SINAI MED CTR | 5 | 0.039 |
| CANC REGISTRY | 5 | 0.039 |
| BEAUMONT HOSP | 5 | 0.039 |
| BASQUE GOVT | 5 | 0.039 |
| BANARAS HINDU UNIV | 5 | 0.039 |
| ARIZONA CTR DIGEST HLTH | 5 | 0.039 |
| ARISTOTLE UNIV THESSALONIKI | 5 | 0.039 |
| ANYANG CANC HOSP | 5 | 0.039 |
| ALBANY MED COLL | 5 | 0.039 |
| AINTREE UNIV HOSP NHS FDN TRUST | 5 | 0.039 |
| AICHI MED UNIV | 5 | 0.039 |
| ACAD ATHENS | 5 | 0.039 |
| ZHEJIANG CHINESE MED UNIV | 4 | 0.031 |
| YUZUNCU YIL UNIV | 4 | 0.031 |
| YONSEI UNIV HLTH SYST | 4 | 0.031 |
| YONSEI CANC CTR | 4 | 0.031 |
| YALE CANC CTR | 4 | 0.031 |
| WINSHIP CANC INST | 4 | 0.031 |
| VET ADM MED CTR | 4 | 0.031 |
| VALL DHEBRON UNIV HOSP | 4 | 0.031 |
| VA NORTH TEXAS HLTH CARE SYST | 4 | 0.031 |
| VA BOSTON HEALTHCARE SYST | 4 | 0.031 |
| UT MD ANDERSON CANC CTR | 4 | 0.031 |
| USDA ARS | 4 | 0.031 |
| USC NORRIS COMPREHENS CANC CTR | 4 | 0.031 |
| UNIV WROCLAW | 4 | 0.031 |
| UNIV VITA SALUTE SAN RAFFAELE | 4 | 0.031 |
| UNIV UPPSALA HOSP | 4 | 0.031 |
| UNIV TEXAS MED BRANCH | 4 | 0.031 |
| UNIV SURREY | 4 | 0.031 |
| UNIV ROSTOCK | 4 | 0.031 |
| UNIV PUERTO RICO | 4 | 0.031 |
| UNIV PRETORIA | 4 | 0.031 |
| UNIV PARIS 13 | 4 | 0.031 |
| UNIV NAT RESOURCES LIFE SCI | 4 | 0.031 |
| UNIV NACL AUTONOMA MEXICO | 4 | 0.031 |
| UNIV MIGUEL HERNANDEZ | 4 | 0.031 |
| UNIV MED PHARM CRAIOVA | 4 | 0.031 |
| UNIV MED CTR EPPENDORF | 4 | 0.031 |
| UNIV LILLE | 4 | 0.031 |
| UNIV KLINIKUM MANNHEIM | 4 | 0.031 |
| UNIV KLINIKUM KOLN | 4 | 0.031 |
| UNIV KLINIKUM | 4 | 0.031 |
| UNIV KIEL | 4 | 0.031 |
| UNIV HOSP ULM | 4 | 0.031 |
| UNIV HOSP MARQUES DE VALDECILLA | 4 | 0.031 |
| UNIV HOSP ERLANGEN | 4 | 0.031 |
| UNIV ESSEN GESAMTHSCH | 4 | 0.031 |
| UNIV CLIN SCHLESWIG HOLSTEIN | 4 | 0.031 |
| UNIV CHINESE ACAD SCI | 4 | 0.031 |
| UNIV CHILE | 4 | 0.031 |
| UNIV CANC CTR | 4 | 0.031 |
| UNIV BURGUNDY | 4 | 0.031 |
| UNIV BARI | 4 | 0.031 |
| UNIV ALCALA DE HENARES | 4 | 0.031 |
| UNIV AIX MARSEILLE 2 | 4 | 0.031 |
| UNITED BRISTOL HEALTHCARE TRUST | 4 | 0.031 |
| UNESP | 4 | 0.031 |
| UCT UNIV CANC CTR FRANKFURT | 4 | 0.031 |
| TOKYO METROPOLITAN GERIATR HOSP | 4 | 0.031 |
| TIANJIN MED UNIV GEN HOSP | 4 | 0.031 |
| TEXAS WOMANS UNIV | 4 | 0.031 |
| TEXAS ONCOL | 4 | 0.031 |
| TECH UNIV DENMARK | 4 | 0.031 |
| TAMA NAGAYAMA HOSP | 4 | 0.031 |
| TAIZHOU PEOPLES HOSP | 4 | 0.031 |
| TAIZHOU HOSP | 4 | 0.031 |
| TAISHAN MED UNIV | 4 | 0.031 |
| TAIPEI MED UNIV HOSP | 4 | 0.031 |
| TAIHE HOSP | 4 | 0.031 |
| STANFORD CANC INST | 4 | 0.031 |
| STADT KLINIKUM SOLINGEN | 4 | 0.031 |
| STADT KLINIKUM BRAUNSCHWEIG | 4 | 0.031 |
| ST VINCENTS CTR APPL MED RES | 4 | 0.031 |
| SOUTHAMPTON UNIV HOSP | 4 | 0.031 |
| SOOKMYUNG WOMENS UNIV | 4 | 0.031 |
| SOKOINE UNIV AGR | 4 | 0.031 |
| SKANE UNIV HOSP | 4 | 0.031 |
| SICHUAN PROV PEOPLES HOSP | 4 | 0.031 |
| SICHUAN CANC HOSP INST | 4 | 0.031 |
| SHANDONG MED COLL | 4 | 0.031 |
| SHAHEED BEHESHTI UNIV MED SCI | 4 | 0.031 |
| SE UNIV | 4 | 0.031 |
| SCOTTSDALE HEALTHCARE | 4 | 0.031 |
| S CHINA NORMAL UNIV | 4 | 0.031 |
| S AFRICAN MRC | 4 | 0.031 |
| ROYAL MELBOURNE HOSP | 4 | 0.031 |
| ROYAL MARSDEN HOSP NHS FDN TRUST | 4 | 0.031 |
| ROYAL ALEXANDRA HOSP | 4 | 0.031 |
| ROCKY MT CANC CTR | 4 | 0.031 |
| REG CANC CTR | 4 | 0.031 |
| RAMBAM HLTH CARE CAMPUS | 4 | 0.031 |
| QUEEN MARY HOSP | 4 | 0.031 |
| QINGDAO MUNICIPAL HOSP | 4 | 0.031 |
| PUBL HLTH HLTH PLANNING DIRECTORATE | 4 | 0.031 |
| PUBL HLTH DIRECTORATE | 4 | 0.031 |
| PROVIDENCE UNIV | 4 | 0.031 |
| PRINCE WALES HOSP | 4 | 0.031 |
| PORTSMOUTH HOSP TRUST | 4 | 0.031 |
| PONTIFICIA UNIV CATOLICA CHILE | 4 | 0.031 |
| PO ROYAL BRISBANE HOSP | 4 | 0.031 |
| PEOPLES LIBERAT ARMY GEN HOSP | 4 | 0.031 |
| PENN STATE COLL MED | 4 | 0.031 |
| PAYAM NOOR UNIV | 4 | 0.031 |
| PANJAB UNIV | 4 | 0.031 |
| OTTO VON GUERICKE UNIV | 4 | 0.031 |
| OSPED NIGUARDA CA GRANDA | 4 | 0.031 |
| ONCOTHERAPY SCI INC | 4 | 0.031 |
| NN BLOKHIN RUSSIAN CANC RES CTR | 4 | 0.031 |
| NAVY GEN HOSP | 4 | 0.031 |
| NATL UNIV HLTH SYST | 4 | 0.031 |
| NATL INST HLTH WELF | 4 | 0.031 |
| NATL INST HLTH SCI | 4 | 0.031 |
| NATL INST GENET ENGN BIOTECHNOL | 4 | 0.031 |
| NATL HOSP ORG KURIHAMA MED ADDICT CTR | 4 | 0.031 |
| NATL CTR TUMOR DIS NCT | 4 | 0.031 |
| NARA SOCIAL INSURANCE HOSP | 4 | 0.031 |
| NANJING NORMAL UNIV | 4 | 0.031 |
| N SICHUAN MED COLL | 4 | 0.031 |
| MINIST HLTH MED EDUC | 4 | 0.031 |
| METHODIST HOSP | 4 | 0.031 |
| MERCK KGAA | 4 | 0.031 |
| MEM UNIV NEWFOUNDLAND | 4 | 0.031 |
| MED UNIV LUBLIN | 4 | 0.031 |
| MED CTR POSTGRAD EDUC | 4 | 0.031 |
| MED COLL HOSP | 4 | 0.031 |
| MAYO CLIN SCOTTSDALE | 4 | 0.031 |
| MAYO CLIN ROCHESTER | 4 | 0.031 |
| MASARYK MEM CANC INST | 4 | 0.031 |
| MARIA SKLODOWSKA CURIE MEM CANC CTR | 4 | 0.031 |
| MAKERERE UNIV | 4 | 0.031 |
| MAASTRO CLIN | 4 | 0.031 |
| LUOHE MED COLL | 4 | 0.031 |
| LONDON HLTH SCI CTR | 4 | 0.031 |
| LIHUILI HOSP | 4 | 0.031 |
| LEICESTER ROYAL INFIRM | 4 | 0.031 |
| KYUSHU UNIV HOSP | 4 | 0.031 |
| KYOTO PHARMACEUT UNIV | 4 | 0.031 |
| KYORIN UNIV | 4 | 0.031 |
| KURUME UNIV HOSP | 4 | 0.031 |
| KUMAGAI SATELLITE CLIN | 4 | 0.031 |
| KOSIN UNIV | 4 | 0.031 |
| KOSHIGAYA MUNICIPAL HOSP | 4 | 0.031 |
| KOREA INST RADIOL MED SCI | 4 | 0.031 |
| KOO FDN | 4 | 0.031 |
| KLINIKUM RECHTS DER ISAR | 4 | 0.031 |
| KLINIKUM MAGDEBURG | 4 | 0.031 |
| KLINIKUM BREMEN OST | 4 | 0.031 |
| KLINIKUM ASCHAFFENBURG | 4 | 0.031 |
| KEELE UNIV | 4 | 0.031 |
| KARMANOS CANC INST | 4 | 0.031 |
| KANTONSSPITAL AARAU | 4 | 0.031 |
| JOHN PAUL 2 HOSP | 4 | 0.031 |
| JOHANNES GUTENBERG UNIV HOSP MAINZ | 4 | 0.031 |
| JINLING HOSP | 4 | 0.031 |
| JICHI MED UNIV HOSP | 4 | 0.031 |
| JIANGSU PROV INST CANC RES | 4 | 0.031 |
| JESSE BROWN VA MED CTR | 4 | 0.031 |
| JAWAHARLAL NEHRU UNIV | 4 | 0.031 |
| JAMES COOK UNIV | 4 | 0.031 |
| IST ONCOL VENETO | 4 | 0.031 |
| IST NAZL TUMORI | 4 | 0.031 |
| ISALA KLIN | 4 | 0.031 |
| IOV IRCCS | 4 | 0.031 |
| INT GOODWILL HOSP | 4 | 0.031 |
| INT EPIDEMIOL INST | 4 | 0.031 |
| INT AGCY RES CANC IARC WHO | 4 | 0.031 |
| INST UNIV MED SOCIALE PREVENT | 4 | 0.031 |
| INST STAT EPIDEMIOL CANC RES | 4 | 0.031 |
| INST ONCOL LJUBLJANA | 4 | 0.031 |
| INST OCCUPAT MED | 4 | 0.031 |
| INST J PAOLI I CALMETTES | 4 | 0.031 |
| INST CLIN EVALUAT SCI | 4 | 0.031 |
| INST CANCEROL GUSTAVE ROUSSY | 4 | 0.031 |
| INRA | 4 | 0.031 |
| INGHAM INST APPL MED RES | 4 | 0.031 |
| IDIBELL | 4 | 0.031 |
| HYOGO MED UNIV | 4 | 0.031 |
| HUNGKUANG UNIV | 4 | 0.031 |
| HUMAN GENET FDN HUGEF | 4 | 0.031 |
| HUBEI CANC HOSP | 4 | 0.031 |
| HUAZHONG AGR UNIV | 4 | 0.031 |
| HOSP SICK CHILDREN | 4 | 0.031 |
| HOSP RAMON CAJAL | 4 | 0.031 |
| HOSP DURAN REYNALS | 4 | 0.031 |
| HOSP CLIN MONTEVIDEO | 4 | 0.031 |
| HOSP CIVILS LYON | 4 | 0.031 |
| HOSP 12 OCTUBRE | 4 | 0.031 |
| HOP MORVAN | 4 | 0.031 |
| HOP LA PITIE SALPETRIERE | 4 | 0.031 |
| HOP CLAUDE HURIEZ | 4 | 0.031 |
| HIROSHIMA RED CROSS HOSP | 4 | 0.031 |
| HENRY FORD HLTH SYST | 4 | 0.031 |
| HELSINKI UNIV HOSP | 4 | 0.031 |
| HELEN F GRAHAM CANC CTR | 4 | 0.031 |
| HEBEI UNIV | 4 | 0.031 |
| HEBEI CHEST HOSP | 4 | 0.031 |
| HANGZHOU NORMAL UNIV | 4 | 0.031 |
| HAMACHO CTR BLDG CLIN | 4 | 0.031 |
| HAGA HOSP | 4 | 0.031 |
| GUSTAVE ROUSSY | 4 | 0.031 |
| GURU NANAK DEV UNIV | 4 | 0.031 |
| GUNMA CHUO HOSP | 4 | 0.031 |
| GUILAN UNIV MED SCI | 4 | 0.031 |
| GREENEBAUM CANC CTR | 4 | 0.031 |
| GEN HOSP PORDENONE | 4 | 0.031 |
| FUKUOKA WAJIRO HOSP | 4 | 0.031 |
| FIRST HOSP ZIBO | 4 | 0.031 |
| FERDOWSI UNIV MASHHAD | 4 | 0.031 |
| FEICHENG PEOPLES HOSP | 4 | 0.031 |
| FEDERAT FRANCOPHONE CANCEROL DIGEST | 4 | 0.031 |
| EWHA WOMANS UNIV | 4 | 0.031 |
| EUROPEAN INST ONCOL | 4 | 0.031 |
| ESMO HEAD OFF | 4 | 0.031 |
| DUKE CLIN RES INST | 4 | 0.031 |
| DREXEL UNIV | 4 | 0.031 |
| DNA CHIP RES INC | 4 | 0.031 |
| DEPT RADIOTHERAPY RADIODIAGNOST FEDERICO II NAP | 4 | 0.031 |
| DEPT GASTROENTEROL | 4 | 0.031 |
| DANDERYD HOSP | 4 | 0.031 |
| CTR MOL IMMUNOL | 4 | 0.031 |
| CTR LEON BERARD | 4 | 0.031 |
| CTR ANTOINE LACASSAGNE | 4 | 0.031 |
| CTR ADDICT MENTAL HLTH | 4 | 0.031 |
| CLEMENT J ZABLOCKI VA MED CTR | 4 | 0.031 |
| CHUNG SHAN MED UNIV HOSP | 4 | 0.031 |
| CHUGOKU CANC CTR | 4 | 0.031 |
| CHU CLERMONT FERRAND | 4 | 0.031 |
| CHINA PHARMACEUT UNIV | 4 | 0.031 |
| CHILDRENS HOSP | 4 | 0.031 |
| CHARITE CAMPUS VIRCHOW KLINIKUM | 4 | 0.031 |
| CHANGI GEN HOSP | 4 | 0.031 |
| CHA UNIV | 4 | 0.031 |
| CATALAN INST ONCOL ICO IDIBELL | 4 | 0.031 |
| CANGZHOU CENT HOSP | 4 | 0.031 |
| CANC CARE ONTARIO | 4 | 0.031 |
| CAMBRIDGE UNIV HOSP NHS FDN TRUST | 4 | 0.031 |
| BRISTOL HAEMATOL ONCOL CTR | 4 | 0.031 |
| BOWLING GREEN STATE UNIV | 4 | 0.031 |
| BOSTON MED CTR | 4 | 0.031 |
| BIODONOSTIA RES INST | 4 | 0.031 |
| BEILINSON MED CTR | 4 | 0.031 |
| BEIJING UNIV CHINESE MED | 4 | 0.031 |
| BEIJING MIL GEN HOSP | 4 | 0.031 |
| BEIJING HOSP | 4 | 0.031 |
| BEIJING DIGEST DIS CTR | 4 | 0.031 |
| AVICENNE HOSP | 4 | 0.031 |
| AUSTIN HLTH | 4 | 0.031 |
| ARIAKE HOSP | 4 | 0.031 |
| ANKARA UNIV | 4 | 0.031 |
| AMSTERDAM MED CTR | 4 | 0.031 |
| AMER COLL SURG | 4 | 0.031 |
| ALLEGHENY GEN HOSP | 4 | 0.031 |
| ALFRED HOSP | 4 | 0.031 |
| AGR UNIV HEBEI | 4 | 0.031 |
| ACAD TEACHING HOSP WOLFSBURG | 4 | 0.031 |
| ACAD MIL MED SCI | 4 | 0.031 |
| ABERDEEN ROYAL INFIRM | 4 | 0.031 |
| AALBORG HOSP | 4 | 0.031 |
| 81ST HOSP PLA | 4 | 0.031 |
| ZUOYING ARMED FORCES GEN HOSP | 3 | 0.023 |
| ZHONGSHAN UNIV | 3 | 0.023 |
| ZHONGSHAN HOSP | 3 | 0.023 |
| ZHENGZHOU HOSP TRADIT CHINESE MED | 3 | 0.023 |
| ZANJAN UNIV MED SCI | 3 | 0.023 |
| YUNNAN UNIV | 3 | 0.023 |
| YOTSUYA MED CUBE | 3 | 0.023 |
| YOKOHAMA MUNICIPAL HOSP | 3 | 0.023 |
| YOKOHAMA MUNICIPAL CITIZENS HOSP | 3 | 0.023 |
| YOKOHAMA CITY UNIV MED | 3 | 0.023 |
| YIXING PEOPLES HOSP | 3 | 0.023 |
| YEUNGNAM UNIV HOSP | 3 | 0.023 |
| YANTAI YUHUANGDING HOSP | 3 | 0.023 |
| YANGZHOU 1 PEOPLES HOSP | 3 | 0.023 |
| YANBIAN UNIV | 3 | 0.023 |
| YANAN UNIV | 3 | 0.023 |
| YAMAGATA PREFECTURAL CENT HOSP | 3 | 0.023 |
| YALE SCH MED | 3 | 0.023 |
| YAKULT CENT INST MICROBIOL RES | 3 | 0.023 |
| WRIGHT STATE UNIV | 3 | 0.023 |
| WINTHROP UNIV HOSP | 3 | 0.023 |
| WICHITA COMMUNITY CLIN ONCOL PROGRAM | 3 | 0.023 |
| WHO | 3 | 0.023 |
| WEST CHINA HOSP | 3 | 0.023 |
| WASEDA UNIV | 3 | 0.023 |
| VU MED CTR | 3 | 0.023 |
| VET AFFAIRS HOSP | 3 | 0.023 |
| VET AFFAIRS CTR CLIN MANAGEMENT RES | 3 | 0.023 |
| VENETO INST ONCOL | 3 | 0.023 |
| VASAVI MED RES CTR | 3 | 0.023 |
| VARIAN MED SYST | 3 | 0.023 |
| USC | 3 | 0.023 |
| US ONCOL RES INC | 3 | 0.023 |
| US ONCOL | 3 | 0.023 |
| UPMC PRESBYTERIAN | 3 | 0.023 |
| UNIV ZARAGOZA | 3 | 0.023 |
| UNIV ZAGREB | 3 | 0.023 |
| UNIV WUERZBURG HOSP | 3 | 0.023 |
| UNIV WESTERN CAPE | 3 | 0.023 |
| UNIV WESTERN AUSTRALIA | 3 | 0.023 |
| UNIV WARWICK | 3 | 0.023 |
| UNIV VERSAILLES ST QUENTIN | 3 | 0.023 |
| UNIV VALLE | 3 | 0.023 |
| UNIV ULM KLINIKUM | 3 | 0.023 |
| UNIV TUBINGEN HOSP | 3 | 0.023 |
| UNIV TEXAS SCH PUBL HLTH | 3 | 0.023 |
| UNIV TEXAS HLTH SCI CTR TYLER | 3 | 0.023 |
| UNIV TEXAS GRAD SCH BIOMED SCI HOUSTON | 3 | 0.023 |
| UNIV TEACHING HOSP | 3 | 0.023 |
| UNIV SOUTHERN DENMARK | 3 | 0.023 |
| UNIV SOUTHERN CALIF | 3 | 0.023 |
| UNIV SHIZUOKA | 3 | 0.023 |
| UNIV S MANCHESTER HOSP | 3 | 0.023 |
| UNIV S ALABAMA | 3 | 0.023 |
| UNIV ROMA TOR VERGATA | 3 | 0.023 |
| UNIV REPUBLICA | 3 | 0.023 |
| UNIV PORTO IPATIMUP | 3 | 0.023 |
| UNIV POLITECN MARCHE | 3 | 0.023 |
| UNIV PISANA | 3 | 0.023 |
| UNIV PERADENIYA | 3 | 0.023 |
| UNIV PAVIA | 3 | 0.023 |
| UNIV PARIS SUD | 3 | 0.023 |
| UNIV OTAGO | 3 | 0.023 |
| UNIV NOTRE DAME | 3 | 0.023 |
| UNIV NANTES | 3 | 0.023 |
| UNIV NACL CORDOBA | 3 | 0.023 |
| UNIV MONS | 3 | 0.023 |
| UNIV MARBURG | 3 | 0.023 |
| UNIV MANSOURA | 3 | 0.023 |
| UNIV MAINZ KLINIKUM | 3 | 0.023 |
| UNIV LUND HOSP | 3 | 0.023 |
| UNIV LOS ANDES | 3 | 0.023 |
| UNIV LODZ | 3 | 0.023 |
| UNIV LLEIDA | 3 | 0.023 |
| UNIV LISBON | 3 | 0.023 |
| UNIV LEUVEN | 3 | 0.023 |
| UNIV LEICESTER | 3 | 0.023 |
| UNIV KUOPIO | 3 | 0.023 |
| UNIV KLINIKUM ROSTOCK | 3 | 0.023 |
| UNIV KLINIKUM MAGDEBURG AOR | 3 | 0.023 |
| UNIV KLINIKUM LUBECK | 3 | 0.023 |
| UNIV KLINIKUM JENA | 3 | 0.023 |
| UNIV KLINIKUM ESSEN | 3 | 0.023 |
| UNIV KLINIKUM DUSSELDORF | 3 | 0.023 |
| UNIV JENA | 3 | 0.023 |
| UNIV INDONESIA | 3 | 0.023 |
| UNIV HOSP OLOMOUC | 3 | 0.023 |
| UNIV HOSP MANNHEIM | 3 | 0.023 |
| UNIV HOSP MAINZ | 3 | 0.023 |
| UNIV HOSP DUSSELDORF | 3 | 0.023 |
| UNIV HOSP CHUV | 3 | 0.023 |
| UNIV HOSP BOCAGE | 3 | 0.023 |
| UNIV HOSP AACHEN | 3 | 0.023 |
| UNIV HOSP 12 OCTUBRE | 3 | 0.023 |
| UNIV HERTFORDSHIRE | 3 | 0.023 |
| UNIV HAMBURG EPPENDORF | 3 | 0.023 |
| UNIV GOETTINGEN | 3 | 0.023 |
| UNIV GEN HOSP | 3 | 0.023 |
| UNIV FED GOIAS | 3 | 0.023 |
| UNIV CRETE | 3 | 0.023 |
| UNIV COLL HOSP NHS FDN TRUST | 3 | 0.023 |
| UNIV CHICAGO HOSP | 3 | 0.023 |
| UNIV CENT FLORIDA | 3 | 0.023 |
| UNIV CATANIA | 3 | 0.023 |
| UNIV CANC CTR HAMBURG | 3 | 0.023 |
| UNIV CALIF | 3 | 0.023 |
| UNIV CAGLIARI | 3 | 0.023 |
| UNIV BOCHUM | 3 | 0.023 |
| UNIV AUTONOMA MADRID | 3 | 0.023 |
| UNIV ANTWERP | 3 | 0.023 |
| ULUDAG UNIV | 3 | 0.023 |
| ULSTER HOSP | 3 | 0.023 |
| UICC ASIAN REG OFF CANC CONTROL | 3 | 0.023 |
| UCSF HELEN DILLER FAMILY COMPREHENS CANC CTR | 3 | 0.023 |
| UC IRVINE SCH MED | 3 | 0.023 |
| TUMOR HOSP JIANGSU PROV | 3 | 0.023 |
| TRIEMLI HOSP | 3 | 0.023 |
| TRAKIA UNIV | 3 | 0.023 |
| TORALDO HOSP | 3 | 0.023 |
| TON YEN GEN HOSP | 3 | 0.023 |
| TOKYO DENT COLL | 3 | 0.023 |
| TNO QUAL LIFE | 3 | 0.023 |
| TIANJIN CHEST HOSP | 3 | 0.023 |
| TENGZHOU CENT PEOPLES HOSP | 3 | 0.023 |
| TANZANIA FOOD DRUGS AUTHOR | 3 | 0.023 |
| TAMPERE UNIV | 3 | 0.023 |
| TAKARA BIO INC | 3 | 0.023 |
| TAIXING PEOPLES HOSP | 3 | 0.023 |
| TAIXING CTR DIS PREVENT CONTROL | 3 | 0.023 |
| SYLVESTER COMPREHENS CANC CTR | 3 | 0.023 |
| SWEDISH CANC INST MED CTR | 3 | 0.023 |
| SWANSEA UNIV | 3 | 0.023 |
| SUWA RED CROSS HOSP | 3 | 0.023 |
| SUNNYBROOK RES INST | 3 | 0.023 |
| STOCKHOLM UNIV | 3 | 0.023 |
| STADT KLINIKUM | 3 | 0.023 |
| STADT KLINIKEN | 3 | 0.023 |
| ST ORSOLA MALPIGHI UNIV HOSP | 3 | 0.023 |
| ST LUKES HOSP | 3 | 0.023 |
| ST LUCAS ANDREAS HOSP | 3 | 0.023 |
| ST JOSEPH HOSP | 3 | 0.023 |
| ST JOSEPH CANC INST | 3 | 0.023 |
| ST ELIZABETH HOSP | 3 | 0.023 |
| ST CLARA HOSP | 3 | 0.023 |
| SOUTHERN TOHOKU PROTON THERAPY CTR | 3 | 0.023 |
| SOUTHAMPTON GEN HOSP | 3 | 0.023 |
| SOUTH AFRICAN MED RES COUNCIL | 3 | 0.023 |
| SO GEN HOSP | 3 | 0.023 |
| SKIMS | 3 | 0.023 |
| SIMON FRASER UNIV | 3 | 0.023 |
| SIDNEY KIMMEL COMPREHENS CANC CTR | 3 | 0.023 |
| SICHUAN ACAD MED SCI | 3 | 0.023 |
| SHIRAZ UNIV | 3 | 0.023 |
| SHERI KASHMIR INST MED SCI | 3 | 0.023 |
| SHARIATI HOSP | 3 | 0.023 |
| SHANGHAI XIANHUI PHARMACEUT CO LTD | 3 | 0.023 |
| SHAN DONG CANC HOSP | 3 | 0.023 |
| SHAHID BEHESHTI UNIV | 3 | 0.023 |
| SENDAI CITY MED CTR | 3 | 0.023 |
| SEATTLE CANC CARE ALLIANCE | 3 | 0.023 |
| SD ASFENDIYAROV KAZAKH NATL MED UNIV | 3 | 0.023 |
| SCRIPPS CLIN | 3 | 0.023 |
| SANTA CHIARA HOSP | 3 | 0.023 |
| SANFORD BURNHAM MED RES INST | 3 | 0.023 |
| SANDWELL GEN HOSP | 3 | 0.023 |
| SAISEIKAI FUKUOKA GEN HOSP | 3 | 0.023 |
| SAFDARJANG HOSP | 3 | 0.023 |
| RYERSON UNIV | 3 | 0.023 |
| RUTGERS CANC INST NEW JERSEY | 3 | 0.023 |
| ROYAL SHREWSBURY HOSP | 3 | 0.023 |
| ROYAL LIVERPOOL UNIV HOSP | 3 | 0.023 |
| ROYAL INST TECHNOL | 3 | 0.023 |
| ROYAL DEVON EXETER NHS FDN TRUST | 3 | 0.023 |
| ROYAL COLL SURGEONS IRELAND | 3 | 0.023 |
| ROYAL BOURNEMOUTH HOSP | 3 | 0.023 |
| ROCHE PROD PTY LTD | 3 | 0.023 |
| ROCHE | 3 | 0.023 |
| RHODES UNIV | 3 | 0.023 |
| RES INST TRAUMATOL ORTHOPED | 3 | 0.023 |
| RES INST | 3 | 0.023 |
| REINIER DE GRAAF HOSP | 3 | 0.023 |
| REG UNIV HOSP CTR | 3 | 0.023 |
| RAJIV GANDHI CANC INST RES CTR | 3 | 0.023 |
| QMUL | 3 | 0.023 |
| PUBL HLTH INST NAVARRA | 3 | 0.023 |
| PORTUGUESE INST ONCOL | 3 | 0.023 |
| POMERANIAN MED UNIV | 3 | 0.023 |
| PFIZER | 3 | 0.023 |
| PERELMAN CTR ADV MED | 3 | 0.023 |
| PEOPLES HOSP TANGSHAN | 3 | 0.023 |
| PEOPLES HOSP HENAN PROV | 3 | 0.023 |
| PEOPLES HOSP GUANGXI ZHUANG AUTONOMOUS REG | 3 | 0.023 |
| PEI LING GUAN SI HOSP | 3 | 0.023 |
| PARIS S UNIV | 3 | 0.023 |
| PARACELSUS PRIVATE MED UNIV | 3 | 0.023 |
| PARACELSUS MED UNIV SALZBURG | 3 | 0.023 |
| PALACKY UNIV MED | 3 | 0.023 |
| OSPED S MARIA MISERICORDIA | 3 | 0.023 |
| ORTENAU KLINIKUM LAHR | 3 | 0.023 |
| OKAYAMA SAISEIKAI GEN HOSP | 3 | 0.023 |
| NUOVO REGINA MARGHERITA HOSP | 3 | 0.023 |
| NOTTINGHAM UNIV NHS TRUST | 3 | 0.023 |
| NORWEGIAN INST PUBL HLTH | 3 | 0.023 |
| NORTHWEST UNIV | 3 | 0.023 |
| NORTHSHORE UNIV HLTH SYST | 3 | 0.023 |
| NORDLAND HOSP TRUST | 3 | 0.023 |
| NO GEN HOSP | 3 | 0.023 |
| NINGBO UNIV | 3 | 0.023 |
| NIH | 3 | 0.023 |
| NIGEB | 3 | 0.023 |
| NIAAA | 3 | 0.023 |
| NEW CROSS HOSP | 3 | 0.023 |
| NE AGR UNIV | 3 | 0.023 |
| NATL UNIV IRELAND UNIV COLL CORK | 3 | 0.023 |
| NATL SUN YAT SEN UNIV | 3 | 0.023 |
| NATL RES CTR | 3 | 0.023 |
| NATL NAVAL MED CTR | 3 | 0.023 |
| NATL KAOHSIUNG UNIV APPL SCI | 3 | 0.023 |
| NATL INST ONCOL | 3 | 0.023 |
| NATL INST ADV IND SCI TECHNOL | 3 | 0.023 |
| NATL HOSP ORG SHIKOKU CANC CTR | 3 | 0.023 |
| NATL HOSP ORG KURIHAMA ALCOHOLISM CTR | 3 | 0.023 |
| NATL HOSP ORG HOKKAIDO CANC CTR | 3 | 0.023 |
| NATL ENGN RES CTR MINIATURIZED DETECT SYST | 3 | 0.023 |
| NATL CTR PLANT GENE RES WUHAN | 3 | 0.023 |
| NANTONG CANC HOSP | 3 | 0.023 |
| NANJING MIL COMMAND | 3 | 0.023 |
| NANJING FIRST HOSP | 3 | 0.023 |
| NANJING BAYI HOSP | 3 | 0.023 |
| NANCHONG CENT HOSP | 3 | 0.023 |
| NAGOYA PET IMAGING CTR | 3 | 0.023 |
| MURCIA REG HLTH AUTHOR | 3 | 0.023 |
| MUNSTER UNIV HOSP | 3 | 0.023 |
| MUNICIPAL SAKATA HOSP | 3 | 0.023 |
| MUNICH CTR CLIN STUDIES | 3 | 0.023 |
| MOSCOW MV LOMONOSOV STATE UNIV | 3 | 0.023 |
| MITSUKOSHI HLTH WELF FDN | 3 | 0.023 |
| MINIST EDUC | 3 | 0.023 |
| MERCK SERONO | 3 | 0.023 |
| MEM HOSP | 3 | 0.023 |
| MED RES CTR OULU | 3 | 0.023 |
| MED CTR LEEUWARDEN | 3 | 0.023 |
| MED BIOL LABS CO LTD | 3 | 0.023 |
| MAYO CLIN JACKSONVILLE | 3 | 0.023 |
| MARIO NEGRI INST PHARMACOL RES | 3 | 0.023 |
| MARIE CURIE HOSP BELFAST | 3 | 0.023 |
| MANSOURA UNIV | 3 | 0.023 |
| MAHIDOL UNIV | 3 | 0.023 |
| MACAU UNIV SCI TECHNOL | 3 | 0.023 |
| LYELL MCEWIN HOSP | 3 | 0.023 |
| LOUIS MOURIER UNIV HOSP | 3 | 0.023 |
| LMU | 3 | 0.023 |
| LIVERPOOL HOSP | 3 | 0.023 |
| LINZHOU TUMOR HOSP | 3 | 0.023 |
| LINZHOU ESOPHAGEAL CANC HOSP | 3 | 0.023 |
| LINYI PEOPLES HOSP | 3 | 0.023 |
| LILLY DEUTSCHLAND GMBH | 3 | 0.023 |
| LIAONING PROV CANC HOSP | 3 | 0.023 |
| LEEDS TEACHING HOSP NHS TRUST | 3 | 0.023 |
| LANCASTER GASTROENTEROL INC | 3 | 0.023 |
| LAC USC MED CTR | 3 | 0.023 |
| LA TIMONE UNIV HOSP | 3 | 0.023 |
| KYUNGPOOK NATL UNIV HOSP | 3 | 0.023 |
| KWONG WAH HOSP | 3 | 0.023 |
| KURE MED CTR | 3 | 0.023 |
| KUMAMOTO REG MED CTR | 3 | 0.023 |
| KLINIKUM REG HANNOVER | 3 | 0.023 |
| KLINIKUM NUERNBERG NORD | 3 | 0.023 |
| KLINIKUM LUDWIGSBURG | 3 | 0.023 |
| KLINIKUM KARLSRUHE | 3 | 0.023 |
| KLINIKEN ESSEN SUD | 3 | 0.023 |
| KITASATO UNIV HOSP | 3 | 0.023 |
| KING HUSSEIN CANC CTR | 3 | 0.023 |
| KING FAHAD MED CITY | 3 | 0.023 |
| KEY THORAC TUMOUR EXPT LAB ZHENGZHOU | 3 | 0.023 |
| KERMANSHAH UNIV MED SCI | 3 | 0.023 |
| KEIYUKAI DAINI HOSP | 3 | 0.023 |
| KANTONSSPITAL GRAUBUNDEN | 3 | 0.023 |
| KANTONSSPITAL CHUR | 3 | 0.023 |
| KANTONSSPITAL BADEN | 3 | 0.023 |
| KANTONSSPITAL | 3 | 0.023 |
| KANSAS CITY VA MED CTR | 3 | 0.023 |
| KANAZAWA MED UNIV | 3 | 0.023 |
| KAMEDA MED CTR | 3 | 0.023 |
| KAISERIN ELISABETH SPITAL | 3 | 0.023 |
| KAGOSHIMA MED CTR | 3 | 0.023 |
| JOHNS HOPKINS MED CTR | 3 | 0.023 |
| JINING MED UNIV | 3 | 0.023 |
| JIANGSU PROV HOSP | 3 | 0.023 |
| JIANGSU CANC HOSP RES INST | 3 | 0.023 |
| JEROEN BOSCH HOSP | 3 | 0.023 |
| JAWAHARLAL INST POSTGRAD MED EDUC RES | 3 | 0.023 |
| JAPAN ESOPHAGEAL SOC | 3 | 0.023 |
| IWATE PREFECTURAL CHUBU HOSP | 3 | 0.023 |
| IST TUMORI FDN PASCALE | 3 | 0.023 |
| IST ONCOL VENETO IOV IRCCS | 3 | 0.023 |
| IRCCS SAN RAFFAELE PISANA | 3 | 0.023 |
| INT CTR GENET ENGN BIOTECHNOL | 3 | 0.023 |
| INST TRANSLAT EPIDEMIOL | 3 | 0.023 |
| INST QUAL EFFICIENCY HLTH CARE IQWIG | 3 | 0.023 |
| INST NACL CANCEROL | 3 | 0.023 |
| INST NACL CANC INCA | 3 | 0.023 |
| INST HYG PUBL HLTH HLTH SERV MANAGEMENT | 3 | 0.023 |
| INST CANC ESTADO SAO PAULO | 3 | 0.023 |
| INSELSPITAL BERN | 3 | 0.023 |
| INNSBRUCK MED UNIV | 3 | 0.023 |
| INNER MONGOLIA MED UNIV | 3 | 0.023 |
| INJE UNIV | 3 | 0.023 |
| INHA UNIV | 3 | 0.023 |
| IMPERIAL COLL HEALTHCARE NHS TRUST | 3 | 0.023 |
| ILAM UNIV MED SCI | 3 | 0.023 |
| IGR | 3 | 0.023 |
| ICGEB | 3 | 0.023 |
| IAEA | 3 | 0.023 |
| HUNGARIAN ACAD SCI | 3 | 0.023 |
| HOWARD UNIV | 3 | 0.023 |
| HOWARD HUGHES MED INST | 3 | 0.023 |
| HOUJU MEM HOSP | 3 | 0.023 |
| HOSP VALLE DE HEBRON | 3 | 0.023 |
| HOSP UNIV MIGUEL SERVET | 3 | 0.023 |
| HOSP UNIV MARQUES VALDECILLA | 3 | 0.023 |
| HOSP UNIV MAR | 3 | 0.023 |
| HOSP UNIV CANARIAS | 3 | 0.023 |
| HOSP PEREIRA ROSSELL | 3 | 0.023 |
| HOSP CLIN UNIV | 3 | 0.023 |
| HOSP CLIN SAN CARLOS | 3 | 0.023 |
| HOP ST LOUIS | 3 | 0.023 |
| HOP PRIVE JEAN MERMOZ | 3 | 0.023 |
| HOP HAUTEPIERRE | 3 | 0.023 |
| HOP AVICENNE | 3 | 0.023 |
| HOKKAIDO CANC CTR | 3 | 0.023 |
| HLTH SCI UNIV HOKKAIDO | 3 | 0.023 |
| HIROSHIMA PREFECTURAL HOSP | 3 | 0.023 |
| HERLEV UNIV HOSP | 3 | 0.023 |
| HENAN UNIV TRADIT CHINESE MED | 3 | 0.023 |
| HELIOS KLINIKUM BAD SAAROW | 3 | 0.023 |
| HEBEI UNITED UNIV | 3 | 0.023 |
| HEBEI GEN HOSP | 3 | 0.023 |
| HAUTEPIERRE UNIV HOSP | 3 | 0.023 |
| HANGZHOU CANC HOSP | 3 | 0.023 |
| GUYS ST THOMAS HOSP | 3 | 0.023 |
| GUIZHOU MED UNIV | 3 | 0.023 |
| GUANGXI CANC INST | 3 | 0.023 |
| GORGAN UNIV MED SCI | 3 | 0.023 |
| GERMAN RES CTR ENVIRONM HLTH | 3 | 0.023 |
| GERMAN INST HUMAN NUTR | 3 | 0.023 |
| GERMAN CONSORTIUM TRANSLAT CANC RES DKTK | 3 | 0.023 |
| GEORGIA INST TECHNOL | 3 | 0.023 |
| GEORGE MASON UNIV | 3 | 0.023 |
| GEN UNIV HOSP PRAGUE | 3 | 0.023 |
| GEN UNIV HOSP | 3 | 0.023 |
| GEN HOSP PLA | 3 | 0.023 |
| GEISINGER MED CTR | 3 | 0.023 |
| GAZIANTEP UNIV | 3 | 0.023 |
| GASTROINTESTINAL ASSOCIATES | 3 | 0.023 |
| GARVAN INST MED RES | 3 | 0.023 |
| FUSCC | 3 | 0.023 |
| FUKUI RED CROSS HOSP | 3 | 0.023 |
| FUJIAN PROV CANC HOSP | 3 | 0.023 |
| FUDAN TAIZHOU INST HLTH SCI | 3 | 0.023 |
| FREE UNIV BRUSSELS | 3 | 0.023 |
| FREE UNIV AMSTERDAM | 3 | 0.023 |
| FORDE CENT HOSP | 3 | 0.023 |
| FLORIDA HOSP | 3 | 0.023 |
| FIRST PEOPLES HOSP CHANGZHOU | 3 | 0.023 |
| FDN ARTURO LOPEZ PEREZ | 3 | 0.023 |
| FAC MED | 3 | 0.023 |
| F HOFFMANN LA ROCHE LTD | 3 | 0.023 |
| ERSTA HOSP | 3 | 0.023 |
| EPE | 3 | 0.023 |
| EORTC HEADQUARTERS | 3 | 0.023 |
| EMD SERONO | 3 | 0.023 |
| EAST HOSP | 3 | 0.023 |
| DURHAM VET AFFAIRS MED CTR | 3 | 0.023 |
| DONG A UNIV | 3 | 0.023 |
| DOKUZ EYLUL UNIV | 3 | 0.023 |
| DEUTSCH KREBSFORSCHUNGSZENTRUM | 3 | 0.023 |
| DEUTSCH KLIN DIAGNOST | 3 | 0.023 |
| DEPT ONCOL | 3 | 0.023 |
| DARTMOUTH COLL | 3 | 0.023 |
| DANKOOK UNIV | 3 | 0.023 |
| DANISH CANC SOC RES CTR | 3 | 0.023 |
| DALLAS VET AFFAIRS MED CTR | 3 | 0.023 |
| CTR STAT | 3 | 0.023 |
| CTR RECH CORDELIERS | 3 | 0.023 |
| CTR HOSP UNIV | 3 | 0.023 |
| CTR HOSP REG UNIV LILLE | 3 | 0.023 |
| CTR HOSP REG UNIV | 3 | 0.023 |
| CTR COMPREHENS CANC | 3 | 0.023 |
| CSIR | 3 | 0.023 |
| CRO | 3 | 0.023 |
| CRLC VAL AURELLE | 3 | 0.023 |
| CORK UNIV HOSP | 3 | 0.023 |
| COMPREHENS CANC CTR S | 3 | 0.023 |
| COMPLEJO HOSP NAVARRA | 3 | 0.023 |
| COLLABORAT INNOVAT CTR CANC CHEMOPREVENT | 3 | 0.023 |
| COLL MED | 3 | 0.023 |
| COCHIN HOSP | 3 | 0.023 |
| CNRS | 3 | 0.023 |
| CNR | 3 | 0.023 |
| CLIN VICTOR HUGO | 3 | 0.023 |
| CLEVELAND CLIN FLORIDA | 3 | 0.023 |
| CLCC OSCAR LAMBRET COMPREHENS CANC CTR | 3 | 0.023 |
| CIXIAN CANC INST | 3 | 0.023 |
| CIXIAN CANC HOSP | 3 | 0.023 |
| CIVIL HOSP | 3 | 0.023 |
| CITY HOSP NOTTINGHAM | 3 | 0.023 |
| CHUTUNG VET HOSP | 3 | 0.023 |
| CHUNGBUK NATL UNIV | 3 | 0.023 |
| CHU TIMONE | 3 | 0.023 |
| CHU ROBERT DEBRE | 3 | 0.023 |
| CHU POITIERS | 3 | 0.023 |
| CHU MONTPELLIER | 3 | 0.023 |
| CHU | 3 | 0.023 |
| CHRISTIE NHS FDN TRUST | 3 | 0.023 |
| CHONNAM NATL UNIV HOSP | 3 | 0.023 |
| CHONGQING CANC INST | 3 | 0.023 |
| CHINA US HENAN HORMEL CANC INST | 3 | 0.023 |
| CHINA AGR UNIV | 3 | 0.023 |
| CHIA YI CHRISTIAN HOSP | 3 | 0.023 |
| CHELTENHAM GEN HOSP | 3 | 0.023 |
| CHARLES STURT UNIV | 3 | 0.023 |
| CHANGZHI CANC HOSP | 3 | 0.023 |
| CHAIM SHEBA MED CTR | 3 | 0.023 |
| CENT HOSP | 3 | 0.023 |
| CATHOLIC UNIV DAEGU | 3 | 0.023 |
| CAROL DAVILA UNIV MED PHARM | 3 | 0.023 |
| CARITASKLIN ST THERESIA | 3 | 0.023 |
| CARDIFF VALE NHS TRUST UNIV HOSP WALES | 3 | 0.023 |
| CAPE PENINSULA UNIV TECHNOL | 3 | 0.023 |
| CANC SCI INST SINGAPORE | 3 | 0.023 |
| CANC INST HOSP JFCR | 3 | 0.023 |
| CANC HOSP | 3 | 0.023 |
| CANC CTR SOUTHEASTERN ONTARIO | 3 | 0.023 |
| BP KOIRALA MEM CANC HOSP | 3 | 0.023 |
| BIRMINGHAM HEARTLANDS HOSP | 3 | 0.023 |
| BENQ MED CTR | 3 | 0.023 |
| BELFAST CITY HOSP | 3 | 0.023 |
| BEIJING UNIV | 3 | 0.023 |
| BEIJING UNION MED COLL HOSP | 3 | 0.023 |
| BEIJING CANC HOSP | 3 | 0.023 |
| BEATSON WEST SCOTLAND CANC CTR | 3 | 0.023 |
| BAYSTATE MED CTR | 3 | 0.023 |
| BAYREUTH HOSP | 3 | 0.023 |
| BARTS LONDON QUEEN MARYS SCH MED DENT | 3 | 0.023 |
| BARRETOS CANC HOSP | 3 | 0.023 |
| BALTIMORE VET AFFAIRS MED CTR | 3 | 0.023 |
| BABOL UNIV MED SCI | 3 | 0.023 |
| AZIENDA OSPED UNIV CAREGGI | 3 | 0.023 |
| AVIANO CANC CTR | 3 | 0.023 |
| ATOM BOMB SURVIVORS HOSP | 3 | 0.023 |
| ASSIUT UNIV | 3 | 0.023 |
| ASSISTANCE PUBL HOP MARSEILLE | 3 | 0.023 |
| ASIA UNIV | 3 | 0.023 |
| ANHUI PROV HOSP | 3 | 0.023 |
| ALLEGHENY HLTH NETWORK | 3 | 0.023 |
| ALLAN BLAIR CANC CTR | 3 | 0.023 |
| ALBANY MED CTR | 3 | 0.023 |
| AKDENIZ UNIV | 3 | 0.023 |
| AIN SHAMS UNIV | 3 | 0.023 |
| AGR RES COUNCIL | 3 | 0.023 |
| AGA KHAN UNIV HOSP | 3 | 0.023 |
| AFFILIATED HOSP | 3 | 0.023 |
| ACAD SCI CZECH REPUBLIC | 3 | 0.023 |
| ACAD MED CTR AMSTERDAM | 3 | 0.023 |
| ZUNYI MED COLL | 2 | 0.015 |
| ZHENGZHOU PEOPLES HOSP | 2 | 0.015 |
| ZHEJIANG MED COLL | 2 | 0.015 |
| ZHEJIANG HOSP | 2 | 0.015 |
| ZHEJIANG CANC INST | 2 | 0.015 |
| ZABOL UNIV MED SCI | 2 | 0.015 |
| YUANS GEN HOSP | 2 | 0.015 |
| YORK HOSP | 2 | 0.015 |
| YIXING CANC HOSP | 2 | 0.015 |
| YIWU CITY CENT HOSP | 2 | 0.015 |
| YASHODA HOSP | 2 | 0.015 |
| YANTING CANC HOSP | 2 | 0.015 |
| YANGZHONG PEOPLES HOSP | 2 | 0.015 |
| YANCHENG INST HLTH SCI | 2 | 0.015 |
| YANCHENG CITY 1 PEOPLES HOSP | 2 | 0.015 |
| XUZHOU CENT HOSP | 2 | 0.015 |
| XIJING HOSP | 2 | 0.015 |
| XIANGFAN CENT HOSP | 2 | 0.015 |
| XIAN JIAOTONG UNIV | 2 | 0.015 |
| WYTHENSHAWE HOSP | 2 | 0.015 |
| WOLVERHAMPTON UNIV | 2 | 0.015 |
| WILLIS KNIGHTON CANC CTR | 2 | 0.015 |
| WILFORD HALL USAF MED CTR | 2 | 0.015 |
| WESTAT CORP | 2 | 0.015 |
| WESLEY HOSP | 2 | 0.015 |
| WELLESLEY COLL | 2 | 0.015 |
| WELLCOME TRUST SANGER INST | 2 | 0.015 |
| WEIZMANN INST SCI | 2 | 0.015 |
| WEILL CORNELL UNIV | 2 | 0.015 |
| WEIFANG MED UNIV | 2 | 0.015 |
| WATSON CLIN | 2 | 0.015 |
| WALTER SISULU UNIV | 2 | 0.015 |
| WAKE FOREST UNIV HLTH SCI | 2 | 0.015 |
| VIVANTES KLINIKUM FRIEDRICHSHAIN | 2 | 0.015 |
| VIRGINIA ONCOL ASSOCIATES | 2 | 0.015 |
| VIOLLIER AG | 2 | 0.015 |
| VET AFFAIRS SAN DIEGO HEALTHCARE SYST | 2 | 0.015 |
| VET AFFAIRS GREATER LOS ANGELES HEALTHCARE SYST | 2 | 0.015 |
| VERBEETEN INST | 2 | 0.015 |
| VELLORE INST TECHNOL | 2 | 0.015 |
| VAUDOIS UNIV HOSP | 2 | 0.015 |
| VANDERBILT EPIDEMIOL CTR | 2 | 0.015 |
| VANCOUVER GEN HOSP | 2 | 0.015 |
| VALL HEBRON UNIV HOSP | 2 | 0.015 |
| VA PUGET SOUND HLTH CARE SYST | 2 | 0.015 |
| VA NORTH TEXAS HEALTHCARE SYST | 2 | 0.015 |
| VA N TEXAS HEALTHCARE SYST | 2 | 0.015 |
| VA LONG BEACH HEALTHCARE SYST | 2 | 0.015 |
| VA GREATER LOS ANGELES HEALTHCARE SYST | 2 | 0.015 |
| USDA | 2 | 0.015 |
| US ONCOL RES | 2 | 0.015 |
| US DEPT VET AFFAIRS | 2 | 0.015 |
| URMIA UNIV MED SCI | 2 | 0.015 |
| UNIWERSYTET MED BIALYMSTOKU | 2 | 0.015 |
| UNIV ZIMBABWE | 2 | 0.015 |
| UNIV ZIEKENHUIZEN LEUVEN | 2 | 0.015 |
| UNIV ZAMBIA | 2 | 0.015 |
| UNIV YORK | 2 | 0.015 |
| UNIV WOLLONGONG | 2 | 0.015 |
| UNIV WITTEN HERDECKE | 2 | 0.015 |
| UNIV WARSAW | 2 | 0.015 |
| UNIV VIGO | 2 | 0.015 |
| UNIV ULSTER | 2 | 0.015 |
| UNIV TURKU | 2 | 0.015 |
| UNIV TRONDHEIM HOSP | 2 | 0.015 |
| UNIV TOULOUSE | 2 | 0.015 |
| UNIV TOLEDO | 2 | 0.015 |
| UNIV THESSALY | 2 | 0.015 |
| UNIV TEXAS SAN ANTONIO | 2 | 0.015 |
| UNIV TEXAS HLTH SCI CTR | 2 | 0.015 |
| UNIV TEACHING HOSP LUNEBURG | 2 | 0.015 |
| UNIV TAUBATE | 2 | 0.015 |
| UNIV TARTU | 2 | 0.015 |
| UNIV STRATHCLYDE | 2 | 0.015 |
| UNIV SPITAL BASEL | 2 | 0.015 |
| UNIV SOCIAL WELF REHABIL SCI | 2 | 0.015 |
| UNIV SFAX | 2 | 0.015 |
| UNIV SCHLESWIG HOLSTEIN | 2 | 0.015 |
| UNIV SASSARI | 2 | 0.015 |
| UNIV SAO PAULO RIBEIRAO PRETO | 2 | 0.015 |
| UNIV SAO FRANCISCO | 2 | 0.015 |
| UNIV SANTO TOMAS | 2 | 0.015 |
| UNIV PORTO ISPUP | 2 | 0.015 |
| UNIV POITIERS HOSP | 2 | 0.015 |
| UNIV PIEMONTE ORIENTALE | 2 | 0.015 |
| UNIV PESHAWAR | 2 | 0.015 |
| UNIV PARIS SACLAY | 2 | 0.015 |
| UNIV PARIS 07 | 2 | 0.015 |
| UNIV OVIEDO | 2 | 0.015 |
| UNIV OREBRO | 2 | 0.015 |
| UNIV ORANGE FREE STATE | 2 | 0.015 |
| UNIV ORADEA | 2 | 0.015 |
| UNIV NORD NORGE | 2 | 0.015 |
| UNIV NAVARRA CLIN | 2 | 0.015 |
| UNIV N TEXAS | 2 | 0.015 |
| UNIV MURCIA | 2 | 0.015 |
| UNIV MODENA REGGIO EMILIA | 2 | 0.015 |
| UNIV MICHIGAN HOSP | 2 | 0.015 |
| UNIV MED PHARM IULIU HATIEGANU | 2 | 0.015 |
| UNIV MED PHARM CAROL DAVILA | 2 | 0.015 |
| UNIV MED CTR REGENSBURG | 2 | 0.015 |
| UNIV MED CTR LEIPZIG | 2 | 0.015 |
| UNIV MED CTR HEIDELBERG | 2 | 0.015 |
| UNIV MED CTR FREIBURG | 2 | 0.015 |
| UNIV MED | 2 | 0.015 |
| UNIV MARYLAND MED SYST | 2 | 0.015 |
| UNIV MARCHE | 2 | 0.015 |
| UNIV MALDONADO | 2 | 0.015 |
| UNIV MALAYSIA SABAH | 2 | 0.015 |
| UNIV MALAWI | 2 | 0.015 |
| UNIV LYON 1 | 2 | 0.015 |
| UNIV LYON | 2 | 0.015 |
| UNIV LONDON LONDON SCH HYG TROP MED | 2 | 0.015 |
| UNIV LJUBLJANA | 2 | 0.015 |
| UNIV LAVAL | 2 | 0.015 |
| UNIV LAGOS | 2 | 0.015 |
| UNIV KLINIKUM SCHLESWIG HOLSTEIN | 2 | 0.015 |
| UNIV KLINIKUM BONN | 2 | 0.015 |
| UNIV KLINIKUM AACHEN | 2 | 0.015 |
| UNIV KLIN CHIRURG | 2 | 0.015 |
| UNIV INNSBRUCK | 2 | 0.015 |
| UNIV HULL | 2 | 0.015 |
| UNIV HUELVA | 2 | 0.015 |
| UNIV HOUSTON | 2 | 0.015 |
| UNIV HOSP WUERZBURG | 2 | 0.015 |
| UNIV HOSP VERONA | 2 | 0.015 |
| UNIV HOSP ROBERT DEBRE | 2 | 0.015 |
| UNIV HOSP PADOVA | 2 | 0.015 |
| UNIV HOSP OULU | 2 | 0.015 |
| UNIV HOSP NHS TRUST | 2 | 0.015 |
| UNIV HOSP KU LEUVEN | 2 | 0.015 |
| UNIV HOSP HOTEL DIEU | 2 | 0.015 |
| UNIV HOSP GOTTINGEN | 2 | 0.015 |
| UNIV HOSP FREIBURG | 2 | 0.015 |
| UNIV HOSP EPPENDORF | 2 | 0.015 |
| UNIV HOSP DIJON | 2 | 0.015 |
| UNIV HOSP CTR ZAGREB | 2 | 0.015 |
| UNIV HOSP CTR MOTHER THERESA | 2 | 0.015 |
| UNIV HOSP COVENTRY | 2 | 0.015 |
| UNIV HOSP COLOGNE CIO | 2 | 0.015 |
| UNIV HOSP CARL GUSTAV CARUS | 2 | 0.015 |
| UNIV HOSP C HURIEZ | 2 | 0.015 |
| UNIV HOSP BESANCON | 2 | 0.015 |
| UNIV HOSP BASEL STADT | 2 | 0.015 |
| UNIV HOSP 2K12 IC | 2 | 0.015 |
| UNIV HIGHLANDS ISL | 2 | 0.015 |
| UNIV HAIFA | 2 | 0.015 |
| UNIV GRENOBLE 1 | 2 | 0.015 |
| UNIV GREENWICH | 2 | 0.015 |
| UNIV GENEVA | 2 | 0.015 |
| UNIV G DANNUNZIO | 2 | 0.015 |
| UNIV FUKUI | 2 | 0.015 |
| UNIV FERRARA | 2 | 0.015 |
| UNIV FED SANTA CATARINA | 2 | 0.015 |
| UNIV FED PELOTAS | 2 | 0.015 |
| UNIV FAVALORO | 2 | 0.015 |
| UNIV EASTERN FINLAND | 2 | 0.015 |
| UNIV DURHAM | 2 | 0.015 |
| UNIV DUISBURG GESAMTHSCH | 2 | 0.015 |
| UNIV DUESSELDORF | 2 | 0.015 |
| UNIV COPENHAGEN HOSP | 2 | 0.015 |
| UNIV CLIN | 2 | 0.015 |
| UNIV CANTABRIA | 2 | 0.015 |
| UNIV CAMPUS BIOMED | 2 | 0.015 |
| UNIV BREMEN | 2 | 0.015 |
| UNIV BORDEAUX | 2 | 0.015 |
| UNIV BATH | 2 | 0.015 |
| UNIV AUCKLAND | 2 | 0.015 |
| UNIV ADDIS ABABA | 2 | 0.015 |
| UNION MEM HOSP | 2 | 0.015 |
| UMDNJ RWJMS | 2 | 0.015 |
| UMCU | 2 | 0.015 |
| UMC UTRECHT | 2 | 0.015 |
| ULSAN UNIV HOSP | 2 | 0.015 |
| ULM UNIV CLIN | 2 | 0.015 |
| ULB ERASME BORDET UNIV HOSP | 2 | 0.015 |
| UCT FAC HLTH SCI | 2 | 0.015 |
| UC SAN DIEGO MOORES CANC CTR | 2 | 0.015 |
| UBE IND LTD | 2 | 0.015 |
| UAB | 2 | 0.015 |
| TYLER CANC CTR | 2 | 0.015 |
| TURKU UNIV HOSP | 2 | 0.015 |
| TURKU UNIV | 2 | 0.015 |
| TUNGS TAICHUNG METROHARBOR HOSP | 2 | 0.015 |
| TUMOR HOSP JILIN PROV | 2 | 0.015 |
| TUMOR HOSP HENAN | 2 | 0.015 |
| TUFTS MED CTR | 2 | 0.015 |
| TUEN MUN HOSP | 2 | 0.015 |
| TU MUENCHEN | 2 | 0.015 |
| TSUYAMA CHUO HOSP | 2 | 0.015 |
| TRIEMLISPITAL ZURICH | 2 | 0.015 |
| TRANSLAT GENOM RES INST | 2 | 0.015 |
| TOYONAKA CITY HOSP | 2 | 0.015 |
| TOYAMA UNIV HOSP | 2 | 0.015 |
| TOYAMA MED UNIV | 2 | 0.015 |
| TONGJI CANC CTR HOSP | 2 | 0.015 |
| TOLEDO COMMUNITY HOSP ONCOL PROGRAM CCOP | 2 | 0.015 |
| TOKYO UNIV AGR TECHNOL | 2 | 0.015 |
| TOKYO METROPOLITAN CANC INFECT DIS CTR KOMAGOME | 2 | 0.015 |
| TOKUSHIMA MUNICIPAL HOSP | 2 | 0.015 |
| TOKAI CENT HOSP | 2 | 0.015 |
| TOHOKU UNIV SCH MED | 2 | 0.015 |
| TOHO SAKURA MED CTR | 2 | 0.015 |
| TIANJIN UNIV TRADIT CHINESE MED | 2 | 0.015 |
| TIANJIN UNIV COMMERCE | 2 | 0.015 |
| TIANJIN UNIV | 2 | 0.015 |
| TIANJIN UNION MED CTR | 2 | 0.015 |
| TIANJIN HOSP | 2 | 0.015 |
| THEODOR BILLROTH ACAD | 2 | 0.015 |
| TEXAS SO UNIV | 2 | 0.015 |
| TENNESSEE VALLEY HEALTHCARE SYST | 2 | 0.015 |
| TENNESSEE ONCOL | 2 | 0.015 |
| TEILGEMEINSCHAFTSPRAXIS MOL PATHOL SUDBAYERN | 2 | 0.015 |
| TECH UNIV MUENCHEN | 2 | 0.015 |
| TECH UNIV | 2 | 0.015 |
| TEACHING HOSP | 2 | 0.015 |
| TAZUKE KOFUKAI MED RES INST | 2 | 0.015 |
| TAUSSIG CANC INST | 2 | 0.015 |
| TATA MEM CTR ANNEXE | 2 | 0.015 |
| TARGOS MOL PATHOL | 2 | 0.015 |
| TAPADIA DIAGNOST SERV | 2 | 0.015 |
| TANGSHAN WORKERS HOSP | 2 | 0.015 |
| TANGSHAN GONGREN HOSP | 2 | 0.015 |
| TAMPERE UNIV HOSP | 2 | 0.015 |
| TAIXING CITY CTR DIS CONTROL PREVENT CDC | 2 | 0.015 |
| TAISHAN MED COLL | 2 | 0.015 |
| TAIAN CITY CENT HOSP | 2 | 0.015 |
| SZPITAL SPECJALISTYCZNY SZCZECIN ZDUNOWO | 2 | 0.015 |
| SZENT LASZLO HOSP | 2 | 0.015 |
| SZENT ISTVAN UNIV BUDAPEST | 2 | 0.015 |
| SWOG STAT CTR | 2 | 0.015 |
| SWISS GRP CLIN CANC RES SAKK | 2 | 0.015 |
| SWEDISH UNIV AGR SCI | 2 | 0.015 |
| SWEDISH MED CTR | 2 | 0.015 |
| SUZHOU MUNICIPAL HOSP | 2 | 0.015 |
| SURG COLLABORAT NETWORK LIMBURG | 2 | 0.015 |
| SUREXAM BIOTECH CO LTD | 2 | 0.015 |
| SUNY UPSTATE MED UNIV | 2 | 0.015 |
| SUNTORY LTD | 2 | 0.015 |
| SUNGKYUNKWAN UNIV SCH MED | 2 | 0.015 |
| SUMITOMO HOSP | 2 | 0.015 |
| SUINING CENT HOSP | 2 | 0.015 |
| STRAHLENKLIN ERLANGEN | 2 | 0.015 |
| STAVANGER UNIV HOSP | 2 | 0.015 |
| STATENS SERUM INST | 2 | 0.015 |
| STATE OCEAN ADM | 2 | 0.015 |
| STATE KEY LAB INFECT DIS PREVENT CONTROL | 2 | 0.015 |
| STATE HOSP | 2 | 0.015 |
| STANFORD CANC CTR | 2 | 0.015 |
| STADTSPITAL TRIEMLI | 2 | 0.015 |
| ST VINCENTIUS KLINIKEN | 2 | 0.015 |
| ST VINCENT REG CANC CTR CCOP | 2 | 0.015 |
| ST PIERRE UNIV HOSP | 2 | 0.015 |
| ST PETERSBURG STATE PEDIAT MED ACAD | 2 | 0.015 |
| ST PAULS HOSP | 2 | 0.015 |
| ST MARYS HOSP NHS TRUST | 2 | 0.015 |
| ST MARKS HOSP | 2 | 0.015 |
| ST LOUIS UNIV | 2 | 0.015 |
| ST LOUIS HOSP | 2 | 0.015 |
| ST JUDE CHILDRENS HOSP | 2 | 0.015 |
| ST JOSEPH MERCY HOSP | 2 | 0.015 |
| ST JOSEPH MED CTR | 2 | 0.015 |
| ST JOSEFS HOSP | 2 | 0.015 |
| ST GEORGES UNIV LONDON | 2 | 0.015 |
| ST GEORGES UNIV | 2 | 0.015 |
| ST FRANCIS HOSP MED CTR | 2 | 0.015 |
| ST BARNABAS HOSP | 2 | 0.015 |
| SRINAKHARINWIROT UNIV | 2 | 0.015 |
| SPOLVERINI HOSP | 2 | 0.015 |
| SPITALUL UNIV BUCURESTI | 2 | 0.015 |
| SPITALUL CLIN URGENTA SIBIU | 2 | 0.015 |
| SPEDALI CIVIL BRESCIA | 2 | 0.015 |
| SPECIALIZED STATE HLTH INST | 2 | 0.015 |
| SOUTHEAST RADIAT ONCOL | 2 | 0.015 |
| SOUTHEAST CANC CONTROL CONSORTIUM INC | 2 | 0.015 |
| SOUTHAMPTON UNIV HOSP NHS TRUST | 2 | 0.015 |
| SOONCHUNHYANG UNIV HOSP | 2 | 0.015 |
| SOLOVE RES INST | 2 | 0.015 |
| SOCIAL INSURANCE KYOTO HOSP | 2 | 0.015 |
| SOC THORAC SURG | 2 | 0.015 |
| SO ILLINOIS UNIV | 2 | 0.015 |
| SO ARIZONA VET AFFAIRS HLTH CARE SYST | 2 | 0.015 |
| SNIADECKI REG HOSP | 2 | 0.015 |
| SMHS HOSP | 2 | 0.015 |
| SINT LUCAS ANDREAS HOSP | 2 | 0.015 |
| SINGLETON HOSP | 2 | 0.015 |
| SICHUAN MED UNIV | 2 | 0.015 |
| SHOW CHWAN MEM HOSP | 2 | 0.015 |
| SHEXIAN CANC INST | 2 | 0.015 |
| SHER E KASHMIR UNIV AGR SCI TECHNOL KASHMIR | 2 | 0.015 |
| SHAUKAT KHANUM MEM CANC HOSP RES CTR | 2 | 0.015 |
| SHANXI UNIV | 2 | 0.015 |
| SHANGHAI UNIV | 2 | 0.015 |
| SHANGHAI PROTON HEAVY ION CTR | 2 | 0.015 |
| SHANGHAI FIRST PEOPLES HOSP | 2 | 0.015 |
| SHANGHAI COLL TCM NEW DRUG DISCOVERY | 2 | 0.015 |
| SHANDONG UNIV TRADIT CHINESE MED | 2 | 0.015 |
| SHANDONG TUMOUR HOSP | 2 | 0.015 |
| SHANDONG PROV KEY LAB OTOL | 2 | 0.015 |
| SHANDONG PROV HOSP | 2 | 0.015 |
| SHANDONG NORMAL UNIV | 2 | 0.015 |
| SHANDONG CTR DIS CONTROL PREVENT | 2 | 0.015 |
| SHAN DONG TUMOR HOSP | 2 | 0.015 |
| SHAHROUD UNIV MED SCI | 2 | 0.015 |
| SHAHID SADOUGHI UNIV MED SCI | 2 | 0.015 |
| SHAHID BEHESHTI UNIV MC | 2 | 0.015 |
| SHAHEED BEHESHTI UNIV MED SCI HLTH SERV | 2 | 0.015 |
| SHADYSIDE MED CTR | 2 | 0.015 |
| SHAANXI UNIV CHINESE MED | 2 | 0.015 |
| SEOUL ST MARYS HOSP | 2 | 0.015 |
| SEMNAN UNIV MED SCI | 2 | 0.015 |
| SECOND PEOPLES HOSP NEIJIANG CITY | 2 | 0.015 |
| SECOND PEOPLES HOSP LIANYUNGANG | 2 | 0.015 |
| SECOND PEOPLES HOSP GUANGDONG PROV | 2 | 0.015 |
| SECOND MIL MED UNIV PLA | 2 | 0.015 |
| SCRIPPS GREEN HOSP | 2 | 0.015 |
| SCOTT WHITE MEM HOSP CLIN | 2 | 0.015 |
| SCHIFFLER CANC CTR | 2 | 0.015 |
| SCH PUBL HLTH | 2 | 0.015 |
| SARDAR PATEL MED COLL | 2 | 0.015 |
| SAPIENZA UNIV | 2 | 0.015 |
| SAO PAULO STATE UNIV UNESP | 2 | 0.015 |
| SANTA MARIA MISERICORDIA HOSP | 2 | 0.015 |
| SANTA MARIA DELLA MISERICORDIA HOSP | 2 | 0.015 |
| SANREMO HOSP | 2 | 0.015 |
| SANOFI KK | 2 | 0.015 |
| SANA KLINIKUM OFFENBACH | 2 | 0.015 |
| SAN MATTEO DEGLI INFERMI HOSP | 2 | 0.015 |
| SAN GIUSEPPE HOSP | 2 | 0.015 |
| SAN GIOVANNI BATTISTA HOSP | 2 | 0.015 |
| SAN GERARDO HOSP | 2 | 0.015 |
| SAKARYA UNIV | 2 | 0.015 |
| SAITAMA UNIV | 2 | 0.015 |
| SAIC FREDERICK INC | 2 | 0.015 |
| SABZEVAR UNIV MED SCI | 2 | 0.015 |
| SAARLAND CANC REGISTRY | 2 | 0.015 |
| S ORSOLA MALPIGHI UNIV HOSP | 2 | 0.015 |
| S CROCE CARLE HOSP | 2 | 0.015 |
| S CHINA UNIV TECHNOL | 2 | 0.015 |
| RUSSIAN CANC RES CTR | 2 | 0.015 |
| RTOG STAT CTR | 2 | 0.015 |
| ROYAL UNITED HOSP | 2 | 0.015 |
| ROYAL SUSSEX CTY HOSP | 2 | 0.015 |
| ROYAL LANCASTER INFIRM | 2 | 0.015 |
| ROYAL CORNWALL HOSP | 2 | 0.015 |
| ROYAL COLL SURGEONS ENGLAND | 2 | 0.015 |
| ROYAL BRISBANE WOMENS HOSP | 2 | 0.015 |
| ROUEN UNIV HOSP CHARLES NICOLLE | 2 | 0.015 |
| ROCHE SAS | 2 | 0.015 |
| ROCHE PROD DEV ASIA PACIFIC | 2 | 0.015 |
| ROCHE PHARMACEUT | 2 | 0.015 |
| RIKEN | 2 | 0.015 |
| RICHARD J SOLOVE RES INST | 2 | 0.015 |
| RHEIN WESTFAL TH AACHEN | 2 | 0.015 |
| RES CTR HOSP CHARGED PARTICLE THERAPY | 2 | 0.015 |
| RES CTR CHILDRENS HLTH | 2 | 0.015 |
| RENMIN HOSP | 2 | 0.015 |
| REINIER GRAAF HOSP | 2 | 0.015 |
| RAMS | 2 | 0.015 |
| RADIAT THERAPY ONCOL GRP | 2 | 0.015 |
| RADBOUD UNIV NIJMEGEN MED CTR | 2 | 0.015 |
| QUEENSLAND UNIV TECHNOL | 2 | 0.015 |
| QINGLONG HIGH TECH CO LTD | 2 | 0.015 |
| QEII HLTH SCI CTR | 2 | 0.015 |
| QAZVIN UNIV MED SCI | 2 | 0.015 |
| PURDUE UNIV | 2 | 0.015 |
| PUNJABI UNIV | 2 | 0.015 |
| PUBL HLTH AGCY CANADA | 2 | 0.015 |
| PROVIDENCE CANC CTR | 2 | 0.015 |
| PROSPER HOSP | 2 | 0.015 |
| PRINCETON UNIV | 2 | 0.015 |
| PRESBYTERIAN HOSP | 2 | 0.015 |
| PORTUGUESE ONCOL INST | 2 | 0.015 |
| PORTSMOUTH HOSP NHS TRUST | 2 | 0.015 |
| POLISH ACAD SCI | 2 | 0.015 |
| POLICLIN MONZA | 2 | 0.015 |
| PLA NAVY GEN HOSP | 2 | 0.015 |
| PITIE SALPETRIERE UNIV HOSP | 2 | 0.015 |
| PIERRE BENITE HOSP | 2 | 0.015 |
| PFIZER INC | 2 | 0.015 |
| PETER MACCALLUM CANC INST | 2 | 0.015 |
| PEOPLES HOSP TAIZHOU | 2 | 0.015 |
| PEOPLE HOSP XINJIANG UYGUR AUTONOMOUS REG | 2 | 0.015 |
| PENN STATE HERSHEY MED CTR | 2 | 0.015 |
| PENN STATE HERSHEY CANC INST | 2 | 0.015 |
| PATHOL NORDHESSEN | 2 | 0.015 |
| PAOLI CALMETTES INST | 2 | 0.015 |
| PANTAI HOSP KUALA LUMPUR | 2 | 0.015 |
| PAMELA YOUDE NETHERSOLE EASTERN HOSP | 2 | 0.015 |
| PALO ALTO MED FDN | 2 | 0.015 |
| OXFORD UNIV HOSP NHS FDN TRUST | 2 | 0.015 |
| OXFORD CANC HAEMATOL CTR | 2 | 0.015 |
| OTTO VONGUERICKE UNIV MAGDEGURG | 2 | 0.015 |
| OTTO VON GUERICKE UNIV HOSP | 2 | 0.015 |
| OSPED SACRO CUORE DON CALABRIA | 2 | 0.015 |
| OSPED BORGO TRENTO | 2 | 0.015 |
| OSMANIA UNIV | 2 | 0.015 |
| OSAKI CITIZEN HOSP | 2 | 0.015 |
| OSAKA SAISEIKAI NOE HOSP | 2 | 0.015 |
| OSAKA ROSAI HOSP | 2 | 0.015 |
| OSAKA RED CROSS HOSP | 2 | 0.015 |
| OSAKA POLICE HOSP | 2 | 0.015 |
| ORTENAU KLINIKUM | 2 | 0.015 |
| OREGON OSTEOPOROSIS CTR | 2 | 0.015 |
| ONKOL SCHWERPUNKTPRAXIS | 2 | 0.015 |
| ONKOL PRAXIS HAMBURG LERCHENFELD | 2 | 0.015 |
| ONCOL INST SO SWITZERLAND | 2 | 0.015 |
| ONCOL HEMATOL CARE INC | 2 | 0.015 |
| ON Q ITY INC | 2 | 0.015 |
| OMORI RED CROSS HOSP | 2 | 0.015 |
| OMID HOSP | 2 | 0.015 |
| OHSU KNIGHT CANC INST | 2 | 0.015 |
| OCHSNER HLTH SYST | 2 | 0.015 |
| NW UNIV XIAN | 2 | 0.015 |
| NUMAZU CITY HOSP | 2 | 0.015 |
| NUCL MED UNIT | 2 | 0.015 |
| NTT WEST OSAKA HOSP | 2 | 0.015 |
| NSW CANC COUNCIL | 2 | 0.015 |
| NRG ONCOL STAT DATA MANAGEMENT CTR | 2 | 0.015 |
| NOVARTIS PHARMACEUT | 2 | 0.015 |
| NOVARTIS PHARMA KK | 2 | 0.015 |
| NOVARTIS INST BIOMED RES | 2 | 0.015 |
| NOTTINGHAM UNIV HOSP NHS TRUST | 2 | 0.015 |
| NORWEST PRIVATE HOSP | 2 | 0.015 |
| NORWEGIAN UNIV LIFE SCI | 2 | 0.015 |
| NORWEGIAN RADIUM HOSP | 2 | 0.015 |
| NORWEGIAN INST AGR ENVIRONM RES | 2 | 0.015 |
| NORTHEASTERN UNIV | 2 | 0.015 |
| NORTH WEST UNIV | 2 | 0.015 |
| NORDLAND HOSP BODO | 2 | 0.015 |
| NIZAMS INST MED SCI | 2 | 0.015 |
| NIPPON MED COLL | 2 | 0.015 |
| NIOSH | 2 | 0.015 |
| NINEWELLS HOSP | 2 | 0.015 |
| NIIGATA CITY GEN HOSP | 2 | 0.015 |
| NHS NSS ISD | 2 | 0.015 |
| NHS FDN TRUST | 2 | 0.015 |
| NHLBI | 2 | 0.015 |
| NEWYORK PRESBYTERIAN HOSP | 2 | 0.015 |
| NEW YORK PRESBYTERIAN VET ADM | 2 | 0.015 |
| NEW YORK HARBOR HEALTHCARE SYST | 2 | 0.015 |
| NEW MEXICO CLIN RES OSTEOPOROSIS CTR | 2 | 0.015 |
| NEW JERSEY DEPT HLTH SENIOR SERV | 2 | 0.015 |
| NEW CTR EXCELLENCE | 2 | 0.015 |
| NETHERLANDS ORG APPL SCI RES | 2 | 0.015 |
| NETHERLANDS COMPREHENS CANC ORG IKNL | 2 | 0.015 |
| NEBRASKA MED CTR | 2 | 0.015 |
| NE REG ICMR | 2 | 0.015 |
| NE HILL UNIV | 2 | 0.015 |
| NAZARETH HOSP | 2 | 0.015 |
| NATL TAIPEI UNIV TECHNOL | 2 | 0.015 |
| NATL KAOHSIUNG FIRST UNIV SCI TECHNOL | 2 | 0.015 |
| NATL JEWISH HLTH | 2 | 0.015 |
| NATL INST STAND TECHNOL | 2 | 0.015 |
| NATL INST HLTH RES | 2 | 0.015 |
| NATL INST ENVIRONM HLTH | 2 | 0.015 |
| NATL INST BIOMED INNOVAT | 2 | 0.015 |
| NATL HOSP ORG TOKYO MED CTR | 2 | 0.015 |
| NATL HOSP ORG OSAKA NATL HOSP | 2 | 0.015 |
| NATL HOSP ORG KURE MED CTR | 2 | 0.015 |
| NATL HLTH LAB SERV | 2 | 0.015 |
| NATL ENGN CTR BIOCHIP | 2 | 0.015 |
| NATL COLL NURSING | 2 | 0.015 |
| NATL CLIN RES CTR DIGEST DIS | 2 | 0.015 |
| NATL CANC REGISTRY IRELAND | 2 | 0.015 |
| NATL CANC INST CANADA | 2 | 0.015 |
| NATL CANC CTR SINGAPORE | 2 | 0.015 |
| NATL CANC CTR E | 2 | 0.015 |
| NATIONWIDE CHILDRENS HOSP | 2 | 0.015 |
| NANYANG TECHNOL UNIV | 2 | 0.015 |
| NANYANG CTR HOSP | 2 | 0.015 |
| NANSHAN HOSP | 2 | 0.015 |
| NANPUH HOSP | 2 | 0.015 |
| NANJING UNIV TRADIT CHINESE MED | 2 | 0.015 |
| NAN JING CHEST HOSP | 2 | 0.015 |
| NAGOYA MED CTR | 2 | 0.015 |
| NAGOYA DAINI RED CROSS HOSP | 2 | 0.015 |
| N SHORE UNIV HOSP | 2 | 0.015 |
| MUSASHIKOSUGI HOSP | 2 | 0.015 |
| MURORAN CITY GEN HOSP | 2 | 0.015 |
| MT VERNON HOSP | 2 | 0.015 |
| MT VERNON CANC CTR | 2 | 0.015 |
| MRC BIOSTAT UNIT | 2 | 0.015 |
| MORRISTOWN MEM HOSP | 2 | 0.015 |
| MORINOMIYA CLIN | 2 | 0.015 |
| MONTREAL GEN HOSP | 2 | 0.015 |
| MONASH HLTH | 2 | 0.015 |
| MIYAZAKI UNIV | 2 | 0.015 |
| MIYAGI CANC CTR | 2 | 0.015 |
| MITSUI MEM HOSP | 2 | 0.015 |
| MISSOURI VALLEY CANC CONSORTIUM | 2 | 0.015 |
| MIRIAM HOSP | 2 | 0.015 |
| MIRACA LIFE SCI RES INST | 2 | 0.015 |
| MIRACA LIFE SCI INC | 2 | 0.015 |
| MINIST AGR | 2 | 0.015 |
| MIE UNIV HOSP | 2 | 0.015 |
| MICHIGAN CANC RES CONSORTIUM | 2 | 0.015 |
| METHODIST DALLAS MED CTR | 2 | 0.015 |
| METAIRIE ONCOLOGISTS | 2 | 0.015 |
| MERIDIAN CANC CARE | 2 | 0.015 |
| MERCY HLTH OSTEOPOROSIS BONE HLTH SERV | 2 | 0.015 |
| MENOUFIA UNIV | 2 | 0.015 |
| MENDEL UNIV BRNO | 2 | 0.015 |
| MELBOURNE CTR CLIN SCI | 2 | 0.015 |
| MEIHO UNIV | 2 | 0.015 |
| MEHR HOSP | 2 | 0.015 |
| MEHARRY MED COLL | 2 | 0.015 |
| MED UNIV WIEN | 2 | 0.015 |
| MED UNIV SOUTH CAROLINA | 2 | 0.015 |
| MED UNIV GDANSK | 2 | 0.015 |
| MED TOPIA SOKA | 2 | 0.015 |
| MED SURG SPECIALISTS LLC | 2 | 0.015 |
| MED CTR | 2 | 0.015 |
| MCKESSON SPECIALTY HLTH | 2 | 0.015 |
| MAXIMA MED CTR | 2 | 0.015 |
| MAULANA AZAD MED COLL | 2 | 0.015 |
| MATSUSAKA CHUO HOSP | 2 | 0.015 |
| MASSEY UNIV | 2 | 0.015 |
| MARQUES VALDECILLA UNIV HOSP | 2 | 0.015 |
| MARKUSOVSZKY CTY HOSP | 2 | 0.015 |
| MARIINSKY HOSP | 2 | 0.015 |
| MANIPAL UNIV | 2 | 0.015 |
| MAINE MED CTR | 2 | 0.015 |
| MAIMONIDES CANC CTR | 2 | 0.015 |
| MAHAVIR HOSP RES CTR | 2 | 0.015 |
| MAGGIORE HOSP | 2 | 0.015 |
| MADRAS MED COLL GOVT GEN HOSP | 2 | 0.015 |
| MACKAY MED NURSING MANAGEMENT COLL | 2 | 0.015 |
| M SKLODOWSKA CURIE INST ONCOL | 2 | 0.015 |
| LUZHOU MED COLL | 2 | 0.015 |
| LUDWIG INST CANC RES | 2 | 0.015 |
| LOUISIANA STATE UNIV HLTH SCI | 2 | 0.015 |
| LONDON SCH HYG TROP MED | 2 | 0.015 |
| LLEIDA UNIV | 2 | 0.015 |
| LINZHOU CANC INST | 2 | 0.015 |
| LIAONING UNIV | 2 | 0.015 |
| LEVINE CANC INST | 2 | 0.015 |
| LAUNCESTON GEN HOSP | 2 | 0.015 |
| KYUSHU UNIV HOSP BEPPU | 2 | 0.015 |
| KYOTO MED CTR | 2 | 0.015 |
| KYORITSU UNIV PHARM | 2 | 0.015 |
| KYORIN UNIV HOSP | 2 | 0.015 |
| KWANDONG UNIV | 2 | 0.015 |
| KUNMING INST ZOOL | 2 | 0.015 |
| KRANKENHAUS MARKISCH ODERLAND GMBH | 2 | 0.015 |
| KRANKENHAUS BARMHERZIGE BRUDER REGENSBURG | 2 | 0.015 |
| KRANKENHAUS BARMHERZIGE BRUDER | 2 | 0.015 |
| KRANKENHAUS BAD CANNSTATT | 2 | 0.015 |
| KOTHARI MED CTR | 2 | 0.015 |
| KORLE BU TEACHING HOSP | 2 | 0.015 |
| KOREA RES INST BIOSCI BIOTECHNOL | 2 | 0.015 |
| KOREA FOOD DRUG ADM | 2 | 0.015 |
| KOREA ADV INST SCI TECHNOL | 2 | 0.015 |
| KONKUK UNIV HOSP | 2 | 0.015 |
| KOCHI UNIV | 2 | 0.015 |
| KOCAELI UNIV | 2 | 0.015 |
| KOBE ROSAI HOSP | 2 | 0.015 |
| KOBE INST HLTH | 2 | 0.015 |
| KLINIKUM ST GEORG GGMBH | 2 | 0.015 |
| KLINIKUM OLDENBURG | 2 | 0.015 |
| KLINIKUM NUREMBERG | 2 | 0.015 |
| KLINIKUM NEUMARKT | 2 | 0.015 |
| KLINIKUM BIELEFELD | 2 | 0.015 |
| KLIN HIRSLANDEN | 2 | 0.015 |
| KIZ CUHK JOINT LAB BIORESOURCES MOL RES COMMON | 2 | 0.015 |
| KITAKYUSHU MUNICIPAL MED CTR | 2 | 0.015 |
| KITA HARIMA MED CTR | 2 | 0.015 |
| KINGSTON REG CANC CTR | 2 | 0.015 |
| KING FAHAD SPECIALIST HOSP | 2 | 0.015 |
| KING ABDULLAH UNIV SCI TECHNOL | 2 | 0.015 |
| KING ABDUL AZIZ MED CITY | 2 | 0.015 |
| KINDAI UNIV | 2 | 0.015 |
| KIDWAI MEM INST ONCOL | 2 | 0.015 |
| KEY LAB DIAG TREATMENT TECHNOL THORAC ONCOL ZHE | 2 | 0.015 |
| KEY LAB CANC PREVENT THERAPY | 2 | 0.015 |
| KEMPTEN CLIN | 2 | 0.015 |
| KEIYUKAI SAPPORO | 2 | 0.015 |
| KEIYUKAI INST CLIN SURG PATHOL | 2 | 0.015 |
| KEIO UNIV SCH MED | 2 | 0.015 |
| KECK SCH MED | 2 | 0.015 |
| KAZAKH NATL MED UNIV NAMED SD ASFENDIYAROV | 2 | 0.015 |
| KASTURBA MED COLL HOSP | 2 | 0.015 |
| KASHIMA HOSP | 2 | 0.015 |
| KAROLINSKA UNIV HOSP HUDDINGE | 2 | 0.015 |
| KAROLINSKA UNIV | 2 | 0.015 |
| KARADENIZ TECH UNIV | 2 | 0.015 |
| KANZAKI HOSP | 2 | 0.015 |
| KANTA HAME CENT HOSP | 2 | 0.015 |
| KANSAS STATE UNIV | 2 | 0.015 |
| KANSAI UNIV | 2 | 0.015 |
| KANGWON NATL UNIV | 2 | 0.015 |
| KAMUZU CENT HOSP | 2 | 0.015 |
| KAIFENG CANC HOSP | 2 | 0.015 |
| KAGOSHIMA MUNICIPAL HOSP | 2 | 0.015 |
| JORDAN UNIV SCI TECHNOL | 2 | 0.015 |
| JOHNS HOPKINS SCH PUBL HLTH | 2 | 0.015 |
| JOHNS HOPKINS CANC CTR | 2 | 0.015 |
| JOHN WAYNE CANC INST | 2 | 0.015 |
| JOHN RADCLIFFE HOSP | 2 | 0.015 |
| JINING MED COLL | 2 | 0.015 |
| JINAN MIL GEN HOSP | 2 | 0.015 |
| JILIN AGR UNIV | 2 | 0.015 |
| JICHI MED SCH | 2 | 0.015 |
| JIAO TONG UNIV | 2 | 0.015 |
| JIANGXI PROV PEOPLES HOSP | 2 | 0.015 |
| JIANGSU INST CANC RES | 2 | 0.015 |
| JIANGHAN UNIV | 2 | 0.015 |
| JAPAN SCI TECHNOL AGCY | 2 | 0.015 |
| JAPAN CLIN ONCOL GRP | 2 | 0.015 |
| JAMIA MILLIA ISLAMIA | 2 | 0.015 |
| JAMES COOK UNIV HOSP | 2 | 0.015 |
| JAMES CONNOLLY MEM HOSP | 2 | 0.015 |
| IWATE PREFECTURAL ISAWA HOSP | 2 | 0.015 |
| ISTANBUL BILIM UNIV | 2 | 0.015 |
| IST TOSCANO TUMORI | 2 | 0.015 |
| IST SCI ROMAGNOLO STUDIO CURA TUMORI IRST | 2 | 0.015 |
| IST OSPITALIERI CREMONA | 2 | 0.015 |
| IST ONCOL VENETO IRCCS | 2 | 0.015 |
| IST NAZL STUDIO CURA TUMORI | 2 | 0.015 |
| IRCCS IST CLIN HUMANITAS | 2 | 0.015 |
| IPATIMUP | 2 | 0.015 |
| IOWA ONCOL RES ASSOC CCOP | 2 | 0.015 |
| IOSI | 2 | 0.015 |
| INST ZOOTECNIA | 2 | 0.015 |
| INST TROP MED PRINCE LEOPOLD | 2 | 0.015 |
| INST TROP MED | 2 | 0.015 |
| INST ST CATHERINE | 2 | 0.015 |
| INST PUBL HLTH | 2 | 0.015 |
| INST POST GRAD MED EDUC RES | 2 | 0.015 |
| INST PORTUGUES ONCOL FRANCISCO GENTIL | 2 | 0.015 |
| INST ONCOL RADIOL SERBIA | 2 | 0.015 |
| INST NUCL ENERGY RES | 2 | 0.015 |
| INST NACL CIENCIAS MED NUTR SALVADOR ZUBIRAN | 2 | 0.015 |
| INST MUTUALISTE MONTSOURIS | 2 | 0.015 |
| INST MED RES OCCUPAT HLTH | 2 | 0.015 |
| INST JEAN GODINOT | 2 | 0.015 |
| INST HYG EPIDEMIOL | 2 | 0.015 |
| INST CLAUDIUS REGAUD | 2 | 0.015 |
| INST CATALA ONCOL | 2 | 0.015 |
| INST CANCEROL OUEST | 2 | 0.015 |
| INST CANCEROL LORRAINE | 2 | 0.015 |
| INST BIOMED RES INNOVAT | 2 | 0.015 |
| INST BERGONIE | 2 | 0.015 |
| INGHAM REG MED CTR | 2 | 0.015 |
| INDIAN INST PUBL HLTH | 2 | 0.015 |
| IND TOXICOL RES CTR | 2 | 0.015 |
| IMSS | 2 | 0.015 |
| ILLINOIS ONCOL RES ASSOC CCOP | 2 | 0.015 |
| IKEM | 2 | 0.015 |
| IKEDA MUNICIPAL HOSP | 2 | 0.015 |
| IILM ACAD | 2 | 0.015 |
| IDIBELL HOSP LLOBREGAT | 2 | 0.015 |
| ICO IDIBELL | 2 | 0.015 |
| ICMR | 2 | 0.015 |
| HYOGO ION BEAM MED CTR | 2 | 0.015 |
| HUNAN UNIV TECHNOL | 2 | 0.015 |
| HUMANITAS CLIN RES CTR | 2 | 0.015 |
| HUG | 2 | 0.015 |
| HUBEI UNIV ARTS SCI | 2 | 0.015 |
| HUBEI MED UNIV | 2 | 0.015 |
| HUAIAN SECOND PEOPLES HOSP | 2 | 0.015 |
| HSK DR HORST SCHMIDT KLINIKEN GMBH | 2 | 0.015 |
| HOWARD UNIV HOSP | 2 | 0.015 |
| HOUSTON METHODIST HOSP | 2 | 0.015 |
| HOSP UNIV VIRGEN DEL ROCIO | 2 | 0.015 |
| HOSP UNIV REINA SOFIA | 2 | 0.015 |
| HOSP UNIV RAMON CAJAL | 2 | 0.015 |
| HOSP UNIV PAZ | 2 | 0.015 |
| HOSP UNIV GIRONA DR JOSEP TRUETA | 2 | 0.015 |
| HOSP UNIV DEL MAR | 2 | 0.015 |
| HOSP ST RAPHAEL | 2 | 0.015 |
| HOSP NA HOMOLCE | 2 | 0.015 |
| HOSP MIGUEL SERVET | 2 | 0.015 |
| HOSP MATER DEI | 2 | 0.015 |
| HOSP LLOBREGAT | 2 | 0.015 |
| HOSP GEN UNIV GREGORIO MARANON | 2 | 0.015 |
| HOSP GEN ELCHE | 2 | 0.015 |
| HOSP ERASTO GAERTNER | 2 | 0.015 |
| HOSP DEL MAR | 2 | 0.015 |
| HOSP CLIN UNIV LOZANO BLESA | 2 | 0.015 |
| HOSP BRAUNSCHWEIG | 2 | 0.015 |
| HOSP BRAGA | 2 | 0.015 |
| HOSP BASURTO | 2 | 0.015 |
| HOP UNIV STRASBOURG | 2 | 0.015 |
| HOP ROBERT BOULIN | 2 | 0.015 |
| HOP HENRI MONDOR | 2 | 0.015 |
| HOP CANTONAL UNIV GENEVA | 2 | 0.015 |
| HOP BICHAT CLAUDE BERNARD | 2 | 0.015 |
| HLTH SCI CTR | 2 | 0.015 |
| HLTH CANADA | 2 | 0.015 |
| HLTH BUR ZHENGZHOU | 2 | 0.015 |
| HIRSLANDEN | 2 | 0.015 |
| HIROSHIMA CITY HOSP | 2 | 0.015 |
| HIRO | 2 | 0.015 |
| HINES VET AFFAIRS HOSP | 2 | 0.015 |
| HILLEROD HOSP | 2 | 0.015 |
| HENRY DUNANT HOSP | 2 | 0.015 |
| HENRI BECQUEREL CANC CTR | 2 | 0.015 |
| HENAN MED COLL STAFF WORKERS | 2 | 0.015 |
| HENAN KEY LAB ESOPHAGEAL CANC RES | 2 | 0.015 |
| HELSINKI UNIV CENT HOSP | 2 | 0.015 |
| HELMHOLZ ZENTRUM MUNCHEN | 2 | 0.015 |
| HELMHOLTZ CTR ENVIRONM HLTH | 2 | 0.015 |
| HELIOS KLINIKUM BERLIN BUCH | 2 | 0.015 |
| HEBEI UNIV ENGN | 2 | 0.015 |
| HEBEI NORTH UNIV | 2 | 0.015 |
| HEBEI MED UNIV FOURTH HOSP | 2 | 0.015 |
| HAYS PHARMA CONSULTING | 2 | 0.015 |
| HARBOR UCLA MED CTR | 2 | 0.015 |
| HANGZHOU FIRST PEOPLES HOSP | 2 | 0.015 |
| HAMAMATSU UNIV | 2 | 0.015 |
| HAMADAN UNIV MED SCI | 2 | 0.015 |
| HAKODATE GORYOUKAKU HOSP | 2 | 0.015 |
| HAINAN MED UNIV | 2 | 0.015 |
| HACKENSACK UNIV | 2 | 0.015 |
| GYEONGSANG NATL UNIV HOSP | 2 | 0.015 |
| GYEONGNAM REG CANC CTR | 2 | 0.015 |
| GWANGJU INST SCI TECHNOL | 2 | 0.015 |
| GUSTAVE ROUSSY CANC CAMPUS | 2 | 0.015 |
| GUNMA CANC CTR | 2 | 0.015 |
| GUJARAT CANC RES INST | 2 | 0.015 |
| GUILIN MED UNIV | 2 | 0.015 |
| GUANGZHOU UNIV TRADIT CHINESE MED | 2 | 0.015 |
| GUANGZHOU UNIV CHINESE MED | 2 | 0.015 |
| GUANGZHOU MED COLL | 2 | 0.015 |
| GRP HLTH RES INST | 2 | 0.015 |
| GRIFFITH MED SCH | 2 | 0.015 |
| GREYS HOSP | 2 | 0.015 |
| GREATER BALTIMORE MED CTR | 2 | 0.015 |
| GOVT ROYAPETTAH HOSP | 2 | 0.015 |
| GONGLI HOSP SHANGHAI PUDONG NEW DIST | 2 | 0.015 |
| GONGLI HOSP | 2 | 0.015 |
| GLOUCESTERSHIRE HOSP NHS TRUST | 2 | 0.015 |
| GLOUCESTERSHIRE HOSP NHS FDN TRUST | 2 | 0.015 |
| GLOUCESTER ROYAL HOSP | 2 | 0.015 |
| GLAXOSMITHKLINE INC | 2 | 0.015 |
| GIFU UNIV HOSP | 2 | 0.015 |
| GERMAN SOC SURG | 2 | 0.015 |
| GERMAN INST HUMAN NUTR DIFE POTSDAM REHBRUECKE | 2 | 0.015 |
| GERCOR | 2 | 0.015 |
| GEORGETOWN UNIV HOSP | 2 | 0.015 |
| GEORGES FRANCOIS LECLERC CANC CTR | 2 | 0.015 |
| GEOL SURVEY IRAN | 2 | 0.015 |
| GENTOFTE UNIV HOSP | 2 | 0.015 |
| GEN SAGAMI KOSEI HOSP | 2 | 0.015 |
| GEN INFIRM | 2 | 0.015 |
| GEN HOSP SHENYANG MIL REG | 2 | 0.015 |
| GEMEINSCHAFTSPRAXIS PATHOL | 2 | 0.015 |
| GEISEL SCH MED DARTMOUTH | 2 | 0.015 |
| GEBZE INST TECHNOL | 2 | 0.015 |
| GAUHATI UNIV | 2 | 0.015 |
| GANSU CANC HOSP | 2 | 0.015 |
| GANGNAM SEVERANCE HOSP | 2 | 0.015 |
| GALAXY CARE LAPAROSCOPY INST | 2 | 0.015 |
| FUZHOU GEN HOSP | 2 | 0.015 |
| FUJIAN UNIV TRADIT CHINESE MED | 2 | 0.015 |
| FUJIAN PROV TUMOR HOSP | 2 | 0.015 |
| FUJIAN PROV KEY LAB TRANSLAT CANC MED | 2 | 0.015 |
| FOURTH HOSP HEBEI MED UNIV | 2 | 0.015 |
| FORSYTH INST | 2 | 0.015 |
| FOOYIN UNIV | 2 | 0.015 |
| FLORIDA STATE UNIV | 2 | 0.015 |
| FLEVOZIEKENHUIS | 2 | 0.015 |
| FIRST PEOPLES HOSP | 2 | 0.015 |
| FIORINI HOSP | 2 | 0.015 |
| FIOH | 2 | 0.015 |
| FIOCRUZ MS | 2 | 0.015 |
| FEDERAT FRANCAISE CANCEROL DIGEST | 2 | 0.015 |
| FED UNIV TECHNOL | 2 | 0.015 |
| FDN MED INC | 2 | 0.015 |
| FDN JEAN DAUSSET CEPH | 2 | 0.015 |
| FDN IRCSS IST NAZL TUMORI MILANO | 2 | 0.015 |
| FDN DETECT EARLY GASTR CARCINOMA | 2 | 0.015 |
| FALK CARDIOVASC RES CTR | 2 | 0.015 |
| EXPRIMO NV | 2 | 0.015 |
| EUROPEAN UNIV BRITTANY | 2 | 0.015 |
| ESTONIAN ONCOL CTR | 2 | 0.015 |
| ESTAING UNIV HOSP | 2 | 0.015 |
| ERNST MORITZ ARNDT UNIV GREIFSWALD | 2 | 0.015 |
| ERASMUS MED CTR UNIV | 2 | 0.015 |
| EPWORTH HEALTHCARE | 2 | 0.015 |
| EOTVOS LORAND UNIV | 2 | 0.015 |
| ENGN KEY LAB CELL THERAPY HENAN PROV | 2 | 0.015 |
| EMORY UNIV CLIN | 2 | 0.015 |
| EMORY CLIN | 2 | 0.015 |
| EMERGENCY MURES CTY HOSP | 2 | 0.015 |
| EMERGENCY CTY HOSP | 2 | 0.015 |
| EMD SERONO INC | 2 | 0.015 |
| ELI LILLY UK | 2 | 0.015 |
| EL CAMINO GI MED ASSOCIATES | 2 | 0.015 |
| EINDHOVEN CANC REGISTRY | 2 | 0.015 |
| EDWARD HINES JR VA HOSP | 2 | 0.015 |
| DURHAM VA MED CTR | 2 | 0.015 |
| DUKE NUS GRAD MED SCH | 2 | 0.015 |
| DR LUTFI KIRDAR KARTAL EDUC RES HOSP | 2 | 0.015 |
| DR HORST SCHMIDT KLIN | 2 | 0.015 |
| DONGGUK UNIV SEOUL | 2 | 0.015 |
| DOKKYO UNIV | 2 | 0.015 |
| DIV GASTROENTEROL HEPATOL | 2 | 0.015 |
| DIV GASTROENTEROL | 2 | 0.015 |
| DIST HOSP CHANGZHI CITY | 2 | 0.015 |
| DIGEST HLTH PHYS | 2 | 0.015 |
| DIAKONIEKRANKENHAUS HENRIETTENSTIFTUNG | 2 | 0.015 |
| DHARAMSHILA CANC HOSP RES CTR | 2 | 0.015 |
| DEVENTER HOSP | 2 | 0.015 |
| DEUTSCH GESELL CHIRURG | 2 | 0.015 |
| DERRIFORD HOSP | 2 | 0.015 |
| DEPT VET AFFAIRS | 2 | 0.015 |
| DEPT SURG 1 | 2 | 0.015 |
| DEPT RADIOTHERAPY | 2 | 0.015 |
| DEPT RADIAT ONCOL | 2 | 0.015 |
| DEPT MED | 2 | 0.015 |
| DEPT INTERNAL MED | 2 | 0.015 |
| DEPT GEN VISCERAL THORAC SURG | 2 | 0.015 |
| DEPT GEN SURG | 2 | 0.015 |
| DEMOCRITUS UNIV THRACE | 2 | 0.015 |
| DEAKIN UNIV | 2 | 0.015 |
| DALIAN UNIV | 2 | 0.015 |
| DAIICHI SANKYO CO LTD | 2 | 0.015 |
| CTR VAL DAURELLE | 2 | 0.015 |
| CTR REG LUTTE CANC VAL DAURELLE | 2 | 0.015 |
| CTR PAUL STRAUSS | 2 | 0.015 |
| CTR NACL INVEST CARDIOVASC | 2 | 0.015 |
| CTR MAGELLAN HOP HAUT LEVEQUE | 2 | 0.015 |
| CTR HOSP SAO JOAO | 2 | 0.015 |
| CTR HOSP | 2 | 0.015 |
| CTR GF LECLERC | 2 | 0.015 |
| CTR EXPT MOL MED | 2 | 0.015 |
| CTR CANC RES | 2 | 0.015 |
| CSIR INDIAN INST TOXICOL RES | 2 | 0.015 |
| CROATIAN NATL INST PUBL HLTH | 2 | 0.015 |
| CROATIAN NATL CANC REGISTRY | 2 | 0.015 |
| CRO NATL CANC INST | 2 | 0.015 |
| CRLC VAL DAURELLE | 2 | 0.015 |
| CREDIT VALLEY HOSP | 2 | 0.015 |
| CRANFIELD UNIV | 2 | 0.015 |
| CONNOLLY HOSP | 2 | 0.015 |
| CONNECTICUT DEPT PUBL HLTH | 2 | 0.015 |
| COMPREHENS CANC CTR W | 2 | 0.015 |
| COMPLEJO HOSP OURENSE | 2 | 0.015 |
| COMMUNITY HOSP | 2 | 0.015 |
| COLUMBIA PRESBYTERIAN MED CTR | 2 | 0.015 |
| COLORADO SCH PUBL HLTH | 2 | 0.015 |
| COLORADO CTR BONE RES | 2 | 0.015 |
| COLLABORAT INNOVAT CTR HENAN PROV CANC CHEMOPREVE | 2 | 0.015 |
| COLLABORAT INNOVAT CTR CANC CHEMOPREVENT HENAN | 2 | 0.015 |
| COBURG CANC CTR | 2 | 0.015 |
| CLIN MINIST ECON TRADE IND | 2 | 0.015 |
| CLIN HOSP PORTO ALEGRE | 2 | 0.015 |
| CLIN AUGSBURG | 2 | 0.015 |
| CLIN ARMORICAINE RADIOL | 2 | 0.015 |
| CLATTERBRIDGE CANC CTR | 2 | 0.015 |
| CIXIAN HOSP | 2 | 0.015 |
| CIV MP AREZZO HOSP | 2 | 0.015 |
| CITY HOSP ESSLINGEN | 2 | 0.015 |
| CITY HOSP | 2 | 0.015 |
| CIBER EPIDEMIOL SALUD PUBL CIBERESP | 2 | 0.015 |
| CIBER EPIDEMIOL SALUD PUBL | 2 | 0.015 |
| CHUNG ANG UNIV | 2 | 0.015 |
| CHU TOULOUSE | 2 | 0.015 |
| CHU STRASBOURG | 2 | 0.015 |
| CHU ST MARGUERITE | 2 | 0.015 |
| CHU NANTES | 2 | 0.015 |
| CHU LILLE | 2 | 0.015 |
| CHU LA MILETRIE | 2 | 0.015 |
| CHU GRENOBLE | 2 | 0.015 |
| CHU DIJON | 2 | 0.015 |
| CHU CAEN | 2 | 0.015 |
| CHRU TOURS | 2 | 0.015 |
| CHRU MORVAN | 2 | 0.015 |
| CHRISTIE HOSP NHS TRUST | 2 | 0.015 |
| CHRISTIE HOSP NHS FDN TRUST | 2 | 0.015 |
| CHRISTCHURCH HOSP | 2 | 0.015 |
| CHINESE PLA MED SCH | 2 | 0.015 |
| CHINESE PLA | 2 | 0.015 |
| CHINESE CTR DIS CONTROL | 2 | 0.015 |
| CHINA UNIV GEOSCI | 2 | 0.015 |
| CHINA NATL CTR FOOD SAFETY RISK ASSESSMENT | 2 | 0.015 |
| CHILDRENS MED CTR | 2 | 0.015 |
| CHIBA HOKUSOH HOSP | 2 | 0.015 |
| CHIBA CANC CTR HOSP | 2 | 0.015 |
| CHIA NAN UNIV PHARM SCI | 2 | 0.015 |
| CHENGDU UNIV TRADIT CHINESE MED | 2 | 0.015 |
| CHENGDU MIL COMMAND | 2 | 0.015 |
| CHENGDU MIL AREA | 2 | 0.015 |
| CHENGDE MED COLL | 2 | 0.015 |
| CHEM VET UNTERSUCHUNGSAMT CVUA KARLSRUHE | 2 | 0.015 |
| CHARITE CAMPUS BENJAMIN FRANKLIN | 2 | 0.015 |
| CHARING CROSS HOSP | 2 | 0.015 |
| CHANGZHOU 3 PEOPLES HOSP | 2 | 0.015 |
| CHANGHUA SHOW CHWAN MEM HOSP | 2 | 0.015 |
| CHANGHAI HOSP | 2 | 0.015 |
| CERMS | 2 | 0.015 |
| CENT TAIWAN UNIV SCI TECHNOL | 2 | 0.015 |
| CENT HOSP TAIAN | 2 | 0.015 |
| CENT HOSP LINZHOU CITY | 2 | 0.015 |
| CATHOLIC UNIV PUSAN | 2 | 0.015 |
| CATHOLIC KWANDONG UNIV | 2 | 0.015 |
| CATHAY GEN HOSP | 2 | 0.015 |
| CARLE CANC CTR CCOP | 2 | 0.015 |
| CARL THIEM KLINIKUM COTTBUS | 2 | 0.015 |
| CAPITAL UNIV MED SCI | 2 | 0.015 |
| CANC RES UK | 2 | 0.015 |
| CANC RES INST JIANGSU PROV | 2 | 0.015 |
| CANC RES CTR | 2 | 0.015 |
| CANC PREVENT RES INST ISPO | 2 | 0.015 |
| CANC PREVENT INST CALIF | 2 | 0.015 |
| CANC INST WIA | 2 | 0.015 |
| CANC INST NEW JERSEY | 2 | 0.015 |
| CANC INST HOSP ARIAKE | 2 | 0.015 |
| CANC INST GIOVANNI PAOLO II | 2 | 0.015 |
| CANC HOSP SICHUAN PROV | 2 | 0.015 |
| CANC HOSP HENAN PROV | 2 | 0.015 |
| CANC COUNCIL NEW SOUTH WALES | 2 | 0.015 |
| CANBERRA HOSP | 2 | 0.015 |
| CAMPUS VIRCHOW KLINIKUM | 2 | 0.015 |
| CAMH | 2 | 0.015 |
| CAIRNS BASE HOSP | 2 | 0.015 |
| CAEN UNIV HOSP | 2 | 0.015 |
| CABRINI HOSP | 2 | 0.015 |
| BROAD INST | 2 | 0.015 |
| BRNO UNIV TECHNOL | 2 | 0.015 |
| BREMEN INST PREVENT RES SOCIAL MED | 2 | 0.015 |
| BRAZILIAN NATL CANC INST | 2 | 0.015 |
| BRADFORD TEACHING HOSP NHS FDN TRUST | 2 | 0.015 |
| BOTNAR RES CTR | 2 | 0.015 |
| BON SECOURS CANC INST | 2 | 0.015 |
| BLACKROCK CLIN | 2 | 0.015 |
| BISPEBJERG HOSP | 2 | 0.015 |
| BIOTECH PHARMACEUT CO LTD | 2 | 0.015 |
| BIODIVERS SPA | 2 | 0.015 |
| BINZHOU MED UNIV | 2 | 0.015 |
| BHAGWAN MAHAVIR MED RES CTR | 2 | 0.015 |
| BGI SHENZHEN | 2 | 0.015 |
| BETHUNE MIL MED NCO ACAD PLA | 2 | 0.015 |
| BETHUNE MIL MED COLL | 2 | 0.015 |
| BETHUNE INT PEACE HOSP | 2 | 0.015 |
| BELLVITGE HOSP | 2 | 0.015 |
| BELLVITGE BIOMED RES INST IDIBELL | 2 | 0.015 |
| BELGIAN CANC REGISTRY | 2 | 0.015 |
| BEIJING KEY LAB PRECANCEROUS LES DIGEST DIS | 2 | 0.015 |
| BEIJING INST RADIAT MED | 2 | 0.015 |
| BEIJING FRIENDSHIP HOSP | 2 | 0.015 |
| BEHESHTI UNIV MED SCI | 2 | 0.015 |
| BEATSON INST CANC RES | 2 | 0.015 |
| BAYLOR UNIV | 2 | 0.015 |
| BASHKORTOSTAN CLIN ONCOL CTR | 2 | 0.015 |
| BARTS HLTH NHS TRUST | 2 | 0.015 |
| BAQIYATALLAH UNIV MED SCI | 2 | 0.015 |
| AZIENDA OSPED UNIV PISANA | 2 | 0.015 |
| AZIENDA OSPED BOLOGNA | 2 | 0.015 |
| AUGUSTA KRANKEN ANSTALT | 2 | 0.015 |
| ATHENS MED SCH | 2 | 0.015 |
| ASTRAZENECA GLOBAL R D | 2 | 0.015 |
| ASTRAZENECA | 2 | 0.015 |
| ASST CREMONA | 2 | 0.015 |
| ASKLEPIOS KLIN HARBURG | 2 | 0.015 |
| ASKLEPIOS KLIN BARMBEK | 2 | 0.015 |
| ASAHIKAWA MED UNIV | 2 | 0.015 |
| ASAHI UNIV | 2 | 0.015 |
| ASAHI GEN HOSP | 2 | 0.015 |
| ARS | 2 | 0.015 |
| ARIZONA STATE UNIV | 2 | 0.015 |
| APSS | 2 | 0.015 |
| APOLLO GLENEAGLES CANC HOSP | 2 | 0.015 |
| APJCP EDITORIAL OFF | 2 | 0.015 |
| APHM LA TIMONE | 2 | 0.015 |
| AO AZIENDA IST OSPITALIERI CREMONA | 2 | 0.015 |
| ANTICANC CTR GEORGES FRANCOIS LECLERC | 2 | 0.015 |
| ANTICANC CTR ALEXIS VAUTRIN | 2 | 0.015 |
| ANTICANC BIOTECH BEIJING CO LTD | 2 | 0.015 |
| ANN ARBOR VET AFFAIRS MED CTR | 2 | 0.015 |
| ANKARA ONCOL HOSP | 2 | 0.015 |
| ANGELES CLIN RES INST | 2 | 0.015 |
| ANDIJAN STATE MED INST | 2 | 0.015 |
| AMRITA INST MED SCI | 2 | 0.015 |
| AMIR KABIR UNIV TECHNOL | 2 | 0.015 |
| AMER UNIV BEIRUT | 2 | 0.015 |
| ALMATY STATE INST ADV MED EDUC | 2 | 0.015 |
| ALLGEMEINES KRANKENHAUS | 2 | 0.015 |
| AL AZHAR UNIV | 2 | 0.015 |
| AICHI CANC CTR RES INST | 2 | 0.015 |
| AICHI CANC CTR CENT HOSP | 2 | 0.015 |
| AHVAZ JUNDISHAPUR UNIV MED SCI | 2 | 0.015 |
| AGRO BIOSCI INC | 2 | 0.015 |
| ADELAIDE MICROARRAY CTR | 2 | 0.015 |
| ADELAIDE MEATH HOSP | 2 | 0.015 |
| ABBOTT NW HOSP | 2 | 0.015 |
| 150 CENT HOSP PLA | 2 | 0.015 |
| 12 OCTUBRE UNIV HOSP | 2 | 0.015 |
| (2755 Organizations {0} {1} value(s) outside display options.) | |  |
| (420 records (3.236%){0} records{1} do not contain data in the field being analyzed.) | | |

**Supplemental Table 6. Table that demonstrates authors contributed to publications on esophageal and esophagogastric junction cancer from the Web of Science ^TM^ Core Collection.**

| Authors | records | % of 12978 |
| --- | --- | --- |
| AJANI JA | 143 | 1.102 |
| HOFSTETTER WL | 116 | 0.894 |
| KITAGAWA Y | 105 | 0.809 |
| LEE JH | 104 | 0.801 |
| DOKI Y | 101 | 0.778 |
| ZHANG Y | 93 | 0.717 |
| WATANABE M | 93 | 0.717 |
| WANG Y | 92 | 0.709 |
| LI Y | 92 | 0.709 |
| MORI M | 89 | 0.686 |
| HOLSCHER AH | 85 | 0.655 |
| LI J | 84 | 0.647 |
| ABNET CC | 84 | 0.647 |
| BABA H | 81 | 0.624 |
| LORDICK F | 80 | 0.616 |
| SWISHER SG | 78 | 0.601 |
| TAKEUCHI H | 77 | 0.593 |
| MATSUBARA H | 77 | 0.593 |
| TAKAHASHI T | 76 | 0.586 |
| KOMAKI R | 74 | 0.57 |
| KIM JH | 71 | 0.547 |
| BOLLSCHWEILER E | 70 | 0.539 |
| WANG J | 69 | 0.532 |
| DAWSEY SM | 69 | 0.532 |
| YAMASAKI M | 68 | 0.524 |
| MIYATA H | 68 | 0.524 |
| TAKIGUCHI S | 67 | 0.516 |
| KATO H | 67 | 0.516 |
| BHUTANI MS | 66 | 0.509 |
| BOFFETTA P | 63 | 0.485 |
| WANG X | 62 | 0.478 |
| VALLBOHMER D | 61 | 0.47 |
| SIERSEMA PD | 61 | 0.47 |
| MALEKZADEH R | 60 | 0.462 |
| ZHANG J | 59 | 0.455 |
| KUWANO H | 59 | 0.455 |
| IZBICKI JR | 59 | 0.455 |
| FUJIWARA Y | 59 | 0.455 |
| TANAKA K | 57 | 0.439 |
| KATO K | 57 | 0.439 |
| ALMHANNA K | 56 | 0.431 |
| ZHANG L | 55 | 0.424 |
| KAMANGAR F | 55 | 0.424 |
| WANG L | 54 | 0.416 |
| VAN LANSCHOT JJB | 54 | 0.416 |
| VAN HILLEGERSBERG R | 54 | 0.416 |
| MORITA M | 54 | 0.416 |
| MARIETTE C | 54 | 0.416 |
| MAEHARA Y | 54 | 0.416 |
| ILSON DH | 54 | 0.416 |
| METZGER R | 53 | 0.408 |
| BABA Y | 53 | 0.408 |
| REYNOLDS JV | 52 | 0.401 |
| OTT K | 52 | 0.401 |
| HENEGOUWEN MIV | 52 | 0.401 |
| OKAMOTO K | 50 | 0.385 |
| ZHANG H | 49 | 0.378 |
| NAKAMURA K | 49 | 0.378 |
| NAKAJIMA K | 49 | 0.378 |
| MIYAZAKI T | 49 | 0.378 |
| LEE YC | 49 | 0.378 |
| CORREA AM | 49 | 0.378 |
| TANAKA T | 48 | 0.37 |
| LIN SH | 47 | 0.362 |
| KUROKAWA Y | 47 | 0.362 |
| CASTORO C | 47 | 0.362 |
| YOSHIDA N | 46 | 0.354 |
| RICE TW | 46 | 0.354 |
| NAKAJIMA M | 46 | 0.354 |
| LI H | 45 | 0.347 |
| LEE JM | 45 | 0.347 |
| LAGERGREN J | 45 | 0.347 |
| KAKEJI Y | 45 | 0.347 |
| ISLAMI F | 45 | 0.347 |
| AKUTSU Y | 45 | 0.347 |
| YANG J | 44 | 0.339 |
| RUOL A | 44 | 0.339 |
| LIU Y | 44 | 0.339 |
| LAW S | 44 | 0.339 |
| ZHANG W | 43 | 0.331 |
| OKI E | 43 | 0.331 |
| MURO K | 43 | 0.331 |
| WIJNHOVEN BPL | 42 | 0.324 |
| UDAGAWA H | 42 | 0.324 |
| SHIMADA H | 42 | 0.324 |
| SAEKI H | 42 | 0.324 |
| OZAWA S | 42 | 0.324 |
| LIU J | 42 | 0.324 |
| GUO W | 42 | 0.324 |
| FUJIWARA H | 42 | 0.324 |
| YANO M | 41 | 0.316 |
| JUNG HY | 41 | 0.316 |
| CHEN Y | 41 | 0.316 |
| YANG Y | 40 | 0.308 |
| SHRIDHAR R | 40 | 0.308 |
| NATSUGOE S | 39 | 0.301 |
| ZHANG B | 38 | 0.293 |
| YAMADA S | 38 | 0.293 |
| WANG H | 38 | 0.293 |
| TILANUS HW | 38 | 0.293 |
| TANAKA N | 38 | 0.293 |
| OTSUJI E | 38 | 0.293 |
| OHTSU A | 38 | 0.293 |
| MUTO M | 38 | 0.293 |
| LIU L | 38 | 0.293 |
| LIU G | 38 | 0.293 |
| LAGERGREN P | 38 | 0.293 |
| ICHIKAWA D | 38 | 0.293 |
| CAGOL M | 38 | 0.293 |
| YANG H | 37 | 0.285 |
| WHITEMAN DC | 37 | 0.285 |
| WADA N | 37 | 0.285 |
| SHIMADA Y | 37 | 0.285 |
| RICE DC | 37 | 0.285 |
| LUKETICH JD | 37 | 0.285 |
| LIU C | 37 | 0.285 |
| LANGER R | 37 | 0.285 |
| GOCKEL I | 37 | 0.285 |
| CHEN L | 37 | 0.285 |
| ALFIERI R | 37 | 0.285 |
| LA VECCHIA C | 36 | 0.277 |
| KAWAKUBO H | 36 | 0.277 |
| HOFFE SE | 36 | 0.277 |
| ANCONA E | 36 | 0.277 |
| ZHANG X | 35 | 0.27 |
| WANG W | 35 | 0.27 |
| SIEWERT JR | 35 | 0.27 |
| SHIOZAKI A | 35 | 0.27 |
| OCHIAI T | 35 | 0.27 |
| MARU DM | 35 | 0.27 |
| LIU Q | 35 | 0.27 |
| IWATSUKI M | 35 | 0.27 |
| GU HY | 35 | 0.27 |
| WANG Q | 34 | 0.262 |
| WANG F | 34 | 0.262 |
| NAKAMURA R | 34 | 0.262 |
| LI B | 34 | 0.262 |
| LEE GH | 34 | 0.262 |
| KOMATSU S | 34 | 0.262 |
| KIM YH | 34 | 0.262 |
| HUNTER JG | 34 | 0.262 |
| BRENNAN P | 34 | 0.262 |
| BRABENDER J | 34 | 0.262 |
| XU Y | 33 | 0.254 |
| TANAKA H | 33 | 0.254 |
| SOHDA M | 33 | 0.254 |
| SHINODA M | 33 | 0.254 |
| SHARMA P | 33 | 0.254 |
| SCHOPPMANN SF | 33 | 0.254 |
| SAIKAWA Y | 33 | 0.254 |
| RUURDA JP | 33 | 0.254 |
| QIAO YL | 33 | 0.254 |
| NAKAMURA Y | 33 | 0.254 |
| NAKAMURA T | 33 | 0.254 |
| LIAO ZX | 33 | 0.254 |
| LI L | 33 | 0.254 |
| FUKUCHI M | 33 | 0.254 |
| FU JH | 33 | 0.254 |
| BLUM MA | 33 | 0.254 |
| BECKER K | 33 | 0.254 |
| ZHAO L | 32 | 0.247 |
| YOSHIDA K | 32 | 0.247 |
| YANG L | 32 | 0.247 |
| YAMAMOTO M | 32 | 0.247 |
| TSUBOSA Y | 32 | 0.247 |
| TOH Y | 32 | 0.247 |
| TAYLOR PR | 32 | 0.247 |
| SAKAMOTO Y | 32 | 0.247 |
| POURSHAMS A | 32 | 0.247 |
| OYAMA T | 32 | 0.247 |
| OKUMURA H | 32 | 0.247 |
| MOTOYAMA S | 32 | 0.247 |
| MIYAMOTO Y | 32 | 0.247 |
| LI X | 32 | 0.247 |
| CUNNINGHAM D | 32 | 0.247 |
| CHEN SC | 32 | 0.247 |
| YU JM | 31 | 0.239 |
| WANG Z | 31 | 0.239 |
| SCHNEIDER PM | 31 | 0.239 |
| KUMAR S | 31 | 0.239 |
| KAWANO T | 31 | 0.239 |
| HE J | 31 | 0.239 |
| FREEDMAN ND | 31 | 0.239 |
| AL-BATRAN SE | 31 | 0.239 |
| ZHANG P | 30 | 0.231 |
| YU J | 30 | 0.231 |
| XU W | 30 | 0.231 |
| WESTON B | 30 | 0.231 |
| WANG K | 30 | 0.231 |
| TANG WF | 30 | 0.231 |
| TAKAHASHI H | 30 | 0.231 |
| SHAH MA | 30 | 0.231 |
| SASAKI T | 30 | 0.231 |
| OMORI T | 30 | 0.231 |
| MITTAL B | 30 | 0.231 |
| LIN J | 30 | 0.231 |
| LI M | 30 | 0.231 |
| ISHIMOTO T | 30 | 0.231 |
| HU Y | 30 | 0.231 |
| HIHARA J | 30 | 0.231 |
| ELL C | 30 | 0.231 |
| DONG ZM | 30 | 0.231 |
| CAVALLIN F | 30 | 0.231 |
| ZHOU Y | 29 | 0.223 |
| ZHAO J | 29 | 0.223 |
| YIN J | 29 | 0.223 |
| YAMASHITA H | 29 | 0.223 |
| WU TT | 29 | 0.223 |
| VAUGHAN TL | 29 | 0.223 |
| TANABE S | 29 | 0.223 |
| SUZUKI A | 29 | 0.223 |
| KELSEN DP | 29 | 0.223 |
| HUANG J | 29 | 0.223 |
| GOODMAN KA | 29 | 0.223 |
| CHEN YJ | 29 | 0.223 |
| ZHANG LJ | 28 | 0.216 |
| YAMADA T | 28 | 0.216 |
| UCHIKADO Y | 28 | 0.216 |
| SUNTHARALINGAM M | 28 | 0.216 |
| STEIN HJ | 28 | 0.216 |
| SATO Y | 28 | 0.216 |
| SAITO H | 28 | 0.216 |
| PLUKKER JTM | 28 | 0.216 |
| PLUKKER JT | 28 | 0.216 |
| NAKAMURA M | 28 | 0.216 |
| MARUYAMA K | 28 | 0.216 |
| LOW DE | 28 | 0.216 |
| LI S | 28 | 0.216 |
| LI N | 28 | 0.216 |
| FUJITA H | 28 | 0.216 |
| DREBBER U | 28 | 0.216 |
| CHEN LQ | 28 | 0.216 |
| CHEN J | 28 | 0.216 |
| YASUDA T | 27 | 0.208 |
| YANG M | 27 | 0.208 |
| YAMASHITA Y | 27 | 0.208 |
| WANG LM | 27 | 0.208 |
| WADHWA R | 27 | 0.208 |
| TAKEDA K | 27 | 0.208 |
| TAKAHASHI M | 27 | 0.208 |
| PETERS JH | 27 | 0.208 |
| OKADA M | 27 | 0.208 |
| MURAKAMI K | 27 | 0.208 |
| MAO WM | 27 | 0.208 |
| LAGARDE SM | 27 | 0.208 |
| JINGU K | 27 | 0.208 |
| ISHIGAMI S | 27 | 0.208 |
| IMAMURA Y | 27 | 0.208 |
| FUKUDA K | 27 | 0.208 |
| FEITH M | 27 | 0.208 |
| ZHANG Q | 26 | 0.2 |
| ZHAN QM | 26 | 0.2 |
| YANG X | 26 | 0.2 |
| YAMAMOTO S | 26 | 0.2 |
| SUZUKI T | 26 | 0.2 |
| SUZUKI S | 26 | 0.2 |
| PIESSEN G | 26 | 0.2 |
| MOEHLER M | 26 | 0.2 |
| LI XY | 26 | 0.2 |
| KIMURA M | 26 | 0.2 |
| KIM SB | 26 | 0.2 |
| KIM J | 26 | 0.2 |
| ISHIHARA R | 26 | 0.2 |
| HUANG Q | 26 | 0.2 |
| D'AMICO TA | 26 | 0.2 |
| CHOI KD | 26 | 0.2 |
| WANG JY | 25 | 0.193 |
| TACHIMORI Y | 25 | 0.193 |
| SUDO K | 25 | 0.193 |
| SONG HJ | 25 | 0.193 |
| SHUTO K | 25 | 0.193 |
| SHIOZAKI H | 25 | 0.193 |
| SEMNANI S | 25 | 0.193 |
| SCARPA M | 25 | 0.193 |
| SAKAI M | 25 | 0.193 |
| RUGGE M | 25 | 0.193 |
| PECH O | 25 | 0.193 |
| OHTA T | 25 | 0.193 |
| NAKAGAWA K | 25 | 0.193 |
| MINAMIYA Y | 25 | 0.193 |
| MATSUMOTO M | 25 | 0.193 |
| LI F | 25 | 0.193 |
| KUIPERS EJ | 25 | 0.193 |
| KOJIMA T | 25 | 0.193 |
| IWAGAMI S | 25 | 0.193 |
| HUANG Y | 25 | 0.193 |
| HOSHINO I | 25 | 0.193 |
| HARADA K | 25 | 0.193 |
| HAMAI Y | 25 | 0.193 |
| GUAN XY | 25 | 0.193 |
| ALTORKI NK | 25 | 0.193 |
| ZHAO Y | 24 | 0.185 |
| ZHANG Z | 24 | 0.185 |
| YOKOYAMA A | 24 | 0.185 |
| YANO T | 24 | 0.185 |
| XU L | 24 | 0.185 |
| WONG R | 24 | 0.185 |
| WATSON DI | 24 | 0.185 |
| WANG LD | 24 | 0.185 |
| WANG KK | 24 | 0.185 |
| URA T | 24 | 0.185 |
| STEYERBERG EW | 24 | 0.185 |
| SHAHEEN NJ | 24 | 0.185 |
| PARK SI | 24 | 0.185 |
| PANTEL K | 24 | 0.185 |
| NAKAJIMA Y | 24 | 0.185 |
| MOTOORI M | 24 | 0.185 |
| MIYAZAKI Y | 24 | 0.185 |
| MEREDITH KL | 24 | 0.185 |
| MARKAR SR | 24 | 0.185 |
| LIN P | 24 | 0.185 |
| LI W | 24 | 0.185 |
| LI Q | 24 | 0.185 |
| KAIFI JT | 24 | 0.185 |
| ISHIKAWA H | 24 | 0.185 |
| IGAKI H | 24 | 0.185 |
| HOSOKAWA M | 24 | 0.185 |
| HANNA GB | 24 | 0.185 |
| DE VITA F | 24 | 0.185 |
| COX JD | 24 | 0.185 |
| ABE T | 24 | 0.185 |
| ZHANG F | 23 | 0.177 |
| ZHANG C | 23 | 0.177 |
| YEKEBAS EF | 23 | 0.177 |
| WEI WQ | 23 | 0.177 |
| WANG XL | 23 | 0.177 |
| WANG S | 23 | 0.177 |
| WANG P | 23 | 0.177 |
| VAPORCIYAN AA | 23 | 0.177 |
| TSAO SW | 23 | 0.177 |
| TEN KATE FJW | 23 | 0.177 |
| TANAKA Y | 23 | 0.177 |
| TAKAHASHI S | 23 | 0.177 |
| RUSCH VW | 23 | 0.177 |
| ROMERO Y | 23 | 0.177 |
| NAKAGAWA H | 23 | 0.177 |
| MORI Y | 23 | 0.177 |
| ITO Y | 23 | 0.177 |
| INOSE T | 23 | 0.177 |
| FUJII H | 23 | 0.177 |
| FRIESS H | 23 | 0.177 |
| ETEMADI A | 23 | 0.177 |
| DEMEESTER SR | 23 | 0.177 |
| CHOW WH | 23 | 0.177 |
| CHAO YK | 23 | 0.177 |
| BOKU N | 23 | 0.177 |
| BLAZEBY JM | 23 | 0.177 |
| ZHAO H | 22 | 0.17 |
| ZHANG YJ | 22 | 0.17 |
| ZACHERL J | 22 | 0.17 |
| YANG G | 22 | 0.17 |
| XIAO LC | 22 | 0.17 |
| XIANG JQ | 22 | 0.17 |
| WU YC | 22 | 0.17 |
| WARNECKE-EBERZ U | 22 | 0.17 |
| WANG JS | 22 | 0.17 |
| WANG D | 22 | 0.17 |
| VIETH M | 22 | 0.17 |
| UEDO N | 22 | 0.17 |
| SONG HY | 22 | 0.17 |
| SKINNER HD | 22 | 0.17 |
| SHIRATORI T | 22 | 0.17 |
| OWAKI T | 22 | 0.17 |
| ORDITURA M | 22 | 0.17 |
| NAKANISHI M | 22 | 0.17 |
| MURAKAMI Y | 22 | 0.17 |
| MIYASHITA T | 22 | 0.17 |
| MICHEL P | 22 | 0.17 |
| MEREDITH K | 22 | 0.17 |
| MEHRAN RJ | 22 | 0.17 |
| LIU B | 22 | 0.17 |
| LI YH | 22 | 0.17 |
| LI P | 22 | 0.17 |
| LEE JS | 22 | 0.17 |
| KU GY | 22 | 0.17 |
| KITA Y | 22 | 0.17 |
| KARL RC | 22 | 0.17 |
| HULSHOF MCCM | 22 | 0.17 |
| HIGUCHI K | 22 | 0.17 |
| HERRMANN K | 22 | 0.17 |
| FUKUDA H | 22 | 0.17 |
| FUJISHIRO M | 22 | 0.17 |
| DAIKO H | 22 | 0.17 |
| CORLEY DA | 22 | 0.17 |
| CIARDIELLO F | 22 | 0.17 |
| CHAK A | 22 | 0.17 |
| AJANI J | 22 | 0.17 |
| ZHENG L | 21 | 0.162 |
| ZHANG M | 21 | 0.162 |
| YU L | 21 | 0.162 |
| YOSHIDA T | 21 | 0.162 |
| YOKOBORI T | 21 | 0.162 |
| YAMAMOTO Y | 21 | 0.162 |
| WU DC | 21 | 0.162 |
| WATSON TJ | 21 | 0.162 |
| WANG CY | 21 | 0.162 |
| VLEGGAAR FP | 21 | 0.162 |
| VASHIST YK | 21 | 0.162 |
| TANAKA M | 21 | 0.162 |
| TAKEUCHI Y | 21 | 0.162 |
| SHI YJ | 21 | 0.162 |
| SCHUSTER T | 21 | 0.162 |
| SCHRODER W | 21 | 0.162 |
| SASAKI K | 21 | 0.162 |
| RUSTGI AK | 21 | 0.162 |
| PIMIENTO JM | 21 | 0.162 |
| OKUMURA T | 21 | 0.162 |
| NIWA Y | 21 | 0.162 |
| NISHIMURA Y | 21 | 0.162 |
| NAOMOTO Y | 21 | 0.162 |
| MURAYAMA Y | 21 | 0.162 |
| MIZUSAWA J | 21 | 0.162 |
| MATSUDA S | 21 | 0.162 |
| MAKINO T | 21 | 0.162 |
| MAKINO H | 21 | 0.162 |
| LIU YH | 21 | 0.162 |
| LI K | 21 | 0.162 |
| LEE S | 21 | 0.162 |
| KURASHIGE J | 21 | 0.162 |
| KUMAGAI Y | 21 | 0.162 |
| KISHI K | 21 | 0.162 |
| KATO T | 21 | 0.162 |
| JOBE BA | 21 | 0.162 |
| ITO H | 21 | 0.162 |
| ISHIGURO H | 21 | 0.162 |
| INOUE H | 21 | 0.162 |
| IKOMA H | 21 | 0.162 |
| IISHI H | 21 | 0.162 |
| HONG TS | 21 | 0.162 |
| HOFSTETTER W | 21 | 0.162 |
| HOFFE S | 21 | 0.162 |
| HIRONAKA S | 21 | 0.162 |
| HASHIMOTO T | 21 | 0.162 |
| GUO Y | 21 | 0.162 |
| FUJIWARA T | 21 | 0.162 |
| FENG JF | 21 | 0.162 |
| EROGLU A | 21 | 0.162 |
| EGASHIRA A | 21 | 0.162 |
| DING GW | 21 | 0.162 |
| COOK MB | 21 | 0.162 |
| BALDUS SE | 21 | 0.162 |
| ZHANG YW | 20 | 0.154 |
| YOSHIDA M | 20 | 0.154 |
| YOKOYAMA T | 20 | 0.154 |
| YANG CY | 20 | 0.154 |
| YAMASHITA K | 20 | 0.154 |
| YAMAGUCHI T | 20 | 0.154 |
| WU J | 20 | 0.154 |
| WEN J | 20 | 0.154 |
| WANG YY | 20 | 0.154 |
| STAHL M | 20 | 0.154 |
| SETO Y | 20 | 0.154 |
| RAVI N | 20 | 0.154 |
| ORRINGER MB | 20 | 0.154 |
| OGAWA K | 20 | 0.154 |
| NEGRI E | 20 | 0.154 |
| MILLER RC | 20 | 0.154 |
| MATSUO K | 20 | 0.154 |
| LIU XY | 20 | 0.154 |
| LIU T | 20 | 0.154 |
| LIU H | 20 | 0.154 |
| LI C | 20 | 0.154 |
| LEE J | 20 | 0.154 |
| LANG H | 20 | 0.154 |
| KURIU Y | 20 | 0.154 |
| KUBO N | 20 | 0.154 |
| KRASNA MJ | 20 | 0.154 |
| KOBAYASHI M | 20 | 0.154 |
| KIM SH | 20 | 0.154 |
| KIM JY | 20 | 0.154 |
| KAWADA K | 20 | 0.154 |
| KATO M | 20 | 0.154 |
| JANJIGIAN YY | 20 | 0.154 |
| ISHIKAWA O | 20 | 0.154 |
| HOCHWALD SN | 20 | 0.154 |
| HAYASHI Y | 20 | 0.154 |
| GUO MZ | 20 | 0.154 |
| GRONNIER C | 20 | 0.154 |
| DUBECZ A | 20 | 0.154 |
| CHENG YF | 20 | 0.154 |
| CHEN HQ | 20 | 0.154 |
| CHEN C | 20 | 0.154 |
| CHANG HK | 20 | 0.154 |
| YASUI H | 19 | 0.146 |
| YAMADA Y | 19 | 0.146 |
| WEBER J | 19 | 0.146 |
| WANG SJ | 19 | 0.146 |
| WANG N | 19 | 0.146 |
| UNO T | 19 | 0.146 |
| UENO M | 19 | 0.146 |
| THOMAS CR | 19 | 0.146 |
| TAKETA T | 19 | 0.146 |
| SHIRAKAWA Y | 19 | 0.146 |
| SCOTT WJ | 19 | 0.146 |
| SATO T | 19 | 0.146 |
| SATO H | 19 | 0.146 |
| RIZK NP | 19 | 0.146 |
| PARK JH | 19 | 0.146 |
| OKAZUMI S | 19 | 0.146 |
| OGAWA J | 19 | 0.146 |
| NUMASAKI H | 19 | 0.146 |
| MATSUMOTO S | 19 | 0.146 |
| LU J | 19 | 0.146 |
| LIU ZH | 19 | 0.146 |
| LIU X | 19 | 0.146 |
| LIU R | 19 | 0.146 |
| LIU M | 19 | 0.146 |
| LIU F | 19 | 0.146 |
| LIN JT | 19 | 0.146 |
| LIAO Z | 19 | 0.146 |
| LEWIS WG | 19 | 0.146 |
| KUBOTA T | 19 | 0.146 |
| KOZLOWSKI M | 19 | 0.146 |
| KONO K | 19 | 0.146 |
| KONISHI H | 19 | 0.146 |
| KODAIRA T | 19 | 0.146 |
| KNOEFEL WT | 19 | 0.146 |
| KIM DH | 19 | 0.146 |
| KATADA C | 19 | 0.146 |
| KANEKO K | 19 | 0.146 |
| EMI M | 19 | 0.146 |
| CHO KJ | 19 | 0.146 |
| CHANG DT | 19 | 0.146 |
| BLACKSTONE EH | 19 | 0.146 |
| BERGMAN JJGHM | 19 | 0.146 |
| AYDIN Y | 19 | 0.146 |
| AKIYAMA H | 19 | 0.146 |
| ZHAO S | 18 | 0.139 |
| YANG YS | 18 | 0.139 |
| WU XF | 18 | 0.139 |
| WANG M | 18 | 0.139 |
| WANG GQ | 18 | 0.139 |
| VAN LAARHOVEN HWM | 18 | 0.139 |
| VAN DER GAAST A | 18 | 0.139 |
| UMEZAWA R | 18 | 0.139 |
| UESATO M | 18 | 0.139 |
| TSUSHIMA T | 18 | 0.139 |
| SWISHER S | 18 | 0.139 |
| SUZUKI H | 18 | 0.139 |
| SUN XC | 18 | 0.139 |
| SUN L | 18 | 0.139 |
| SU M | 18 | 0.139 |
| SMIT JK | 18 | 0.139 |
| SHEYHIDIN I | 18 | 0.139 |
| SETOYAMA T | 18 | 0.139 |
| SANO A | 18 | 0.139 |
| SAKURAI H | 18 | 0.139 |
| SAKAI Y | 18 | 0.139 |
| SAITO Y | 18 | 0.139 |
| RYBICKI LA | 18 | 0.139 |
| OHARA T | 18 | 0.139 |
| ODA I | 18 | 0.139 |
| NISHIMURA T | 18 | 0.139 |
| NEMOTO K | 18 | 0.139 |
| NAGATA Y | 18 | 0.139 |
| MURATA K | 18 | 0.139 |
| MELTZER SJ | 18 | 0.139 |
| MA L | 18 | 0.139 |
| LEE PC | 18 | 0.139 |
| KODERA Y | 18 | 0.139 |
| KIM HR | 18 | 0.139 |
| KAWAGUCHI Y | 18 | 0.139 |
| JIANG Y | 18 | 0.139 |
| JAMIESON GG | 18 | 0.139 |
| IDA S | 18 | 0.139 |
| HONDA H | 18 | 0.139 |
| HIYOSHI Y | 18 | 0.139 |
| HIRAKAWA K | 18 | 0.139 |
| HIGASHINO K | 18 | 0.139 |
| HAN Y | 18 | 0.139 |
| FASSAN M | 18 | 0.139 |
| FAN DM | 18 | 0.139 |
| DOLAN JP | 18 | 0.139 |
| DEMEESTER TR | 18 | 0.139 |
| CHEN X | 18 | 0.139 |
| CHEN WQ | 18 | 0.139 |
| CHEN QX | 18 | 0.139 |
| CHEN G | 18 | 0.139 |
| BOSETTI C | 18 | 0.139 |
| BIRNER P | 18 | 0.139 |
| ANDO K | 18 | 0.139 |
| ADELSTEIN DJ | 18 | 0.139 |
| ZHANG ST | 17 | 0.131 |
| ZANINOTTO G | 17 | 0.131 |
| YUAN Y | 17 | 0.131 |
| YOSHIKAWA T | 17 | 0.131 |
| YAMAMOTO K | 17 | 0.131 |
| YAMAGUCHI K | 17 | 0.131 |
| XU J | 17 | 0.131 |
| WU IC | 17 | 0.131 |
| WANG YF | 17 | 0.131 |
| WANG C | 17 | 0.131 |
| VAN ROSSUM PSN | 17 | 0.131 |
| TANIGUCHI H | 17 | 0.131 |
| TAKAYAMA T | 17 | 0.131 |
| TAKAHASHI Y | 17 | 0.131 |
| SMITHERS BM | 17 | 0.131 |
| SHITARA K | 17 | 0.131 |
| SHIROUZU K | 17 | 0.131 |
| SHEN L | 17 | 0.131 |
| QIN YR | 17 | 0.131 |
| PINTO C | 17 | 0.131 |
| PAULIGK C | 17 | 0.131 |
| OMOTO I | 17 | 0.131 |
| NOMURA M | 17 | 0.131 |
| NAGAI K | 17 | 0.131 |
| LUNDELL L | 17 | 0.131 |
| LU N | 17 | 0.131 |
| LERUT T | 17 | 0.131 |
| LEE HJ | 17 | 0.131 |
| KUNISAKI C | 17 | 0.131 |
| KITAJIMA M | 17 | 0.131 |
| KIMURA Y | 17 | 0.131 |
| KIM JS | 17 | 0.131 |
| JATOI A | 17 | 0.131 |
| IWASA S | 17 | 0.131 |
| ITO T | 17 | 0.131 |
| HOPT UT | 17 | 0.131 |
| HONG L | 17 | 0.131 |
| HOELSCHER AH | 17 | 0.131 |
| HEJNA M | 17 | 0.131 |
| GIBSON MK | 17 | 0.131 |
| GAO Y | 17 | 0.131 |
| FUSHIDA S | 17 | 0.131 |
| FITZGERALD RC | 17 | 0.131 |
| ESLICK GD | 17 | 0.131 |
| ELIMOVA E | 17 | 0.131 |
| DARLING GE | 17 | 0.131 |
| CASSON AG | 17 | 0.131 |
| BEUKEMA JC | 17 | 0.131 |
| ANONYMOUS | 17 | 0.131 |
| ZHAO GQ | 16 | 0.123 |
| ZHANG XY | 16 | 0.123 |
| ZHANG SW | 16 | 0.123 |
| ZHANG S | 16 | 0.123 |
| ZHANG N | 16 | 0.123 |
| YOKOTA T | 16 | 0.123 |
| YANG K | 16 | 0.123 |
| YAMAUE H | 16 | 0.123 |
| XU B | 16 | 0.123 |
| WU MT | 16 | 0.123 |
| WAXMAN I | 16 | 0.123 |
| WANG YC | 16 | 0.123 |
| VECCHIONE L | 16 | 0.123 |
| TURKYILMAZ A | 16 | 0.123 |
| TSUKADA K | 16 | 0.123 |
| THOMPSON SK | 16 | 0.123 |
| SZMITKOWSKI M | 16 | 0.123 |
| SILENI VC | 16 | 0.123 |
| SHAN BE | 16 | 0.123 |
| SCHWAIGER M | 16 | 0.123 |
| SAITO K | 16 | 0.123 |
| RONG TH | 16 | 0.123 |
| POUSTCHI H | 16 | 0.123 |
| PORT JL | 16 | 0.123 |
| PENNATHUR A | 16 | 0.123 |
| NINOMIYA I | 16 | 0.123 |
| NIKLINSKI J | 16 | 0.123 |
| MURTHY SC | 16 | 0.123 |
| MURRAY LJ | 16 | 0.123 |
| MORI K | 16 | 0.123 |
| MONIG SP | 16 | 0.123 |
| MINASHI K | 16 | 0.123 |
| LUO KJ | 16 | 0.123 |
| LORENZEN S | 16 | 0.123 |
| LIU D | 16 | 0.123 |
| LIN CC | 16 | 0.123 |
| LIANG J | 16 | 0.123 |
| LI YM | 16 | 0.123 |
| LEE CH | 16 | 0.123 |
| KRISHNADATH KK | 16 | 0.123 |
| KOIZUMI W | 16 | 0.123 |
| KIM SJ | 16 | 0.123 |
| KHADEMI H | 16 | 0.123 |
| KARASHIMA R | 16 | 0.123 |
| ICHIKAWA H | 16 | 0.123 |
| HSU PK | 16 | 0.123 |
| HOMANN N | 16 | 0.123 |
| HOFHEINZ RD | 16 | 0.123 |
| HAYASHI T | 16 | 0.123 |
| HANAOKA N | 16 | 0.123 |
| GROEN H | 16 | 0.123 |
| GAO SG | 16 | 0.123 |
| FUJII Y | 16 | 0.123 |
| FUJII T | 16 | 0.123 |
| FERRI LE | 16 | 0.123 |
| FERGUSON MK | 16 | 0.123 |
| DARLING G | 16 | 0.123 |
| CROSBY T | 16 | 0.123 |
| COIT DG | 16 | 0.123 |
| COHEN SJ | 16 | 0.123 |
| CHEN T | 16 | 0.123 |
| CHEN Q | 16 | 0.123 |
| BROCK MV | 16 | 0.123 |
| BLANK S | 16 | 0.123 |
| BATTAGLIA G | 16 | 0.123 |
| ANDO N | 16 | 0.123 |
| ABRAMS JA | 16 | 0.123 |
| ZHOU W | 15 | 0.116 |
| ZHOU J | 15 | 0.116 |
| ZHANG YL | 15 | 0.116 |
| ZHANG XJ | 15 | 0.116 |
| ZHANG HW | 15 | 0.116 |
| YAMATSUJI T | 15 | 0.116 |
| YAMADA K | 15 | 0.116 |
| WU Y | 15 | 0.116 |
| WILLETT CG | 15 | 0.116 |
| WELSH J | 15 | 0.116 |
| WANG MR | 15 | 0.116 |
| WANG LH | 15 | 0.116 |
| WANG HP | 15 | 0.116 |
| WANG HJ | 15 | 0.116 |
| VAN DER PEET DL | 15 | 0.116 |
| UPADHYAY R | 15 | 0.116 |
| TSENG CK | 15 | 0.116 |
| TANG LH | 15 | 0.116 |
| TALAMINI R | 15 | 0.116 |
| TAKUBO K | 15 | 0.116 |
| TAKAOKA M | 15 | 0.116 |
| SISIC L | 15 | 0.116 |
| SHIN JH | 15 | 0.116 |
| SHIGEMATSU N | 15 | 0.116 |
| SHIGAKI H | 15 | 0.116 |
| SHI J | 15 | 0.116 |
| SHARMA R | 15 | 0.116 |
| SHAKERI R | 15 | 0.116 |
| SCHUHMACHER C | 15 | 0.116 |
| SAFRAN H | 15 | 0.116 |
| RADES D | 15 | 0.116 |
| PATTI MG | 15 | 0.116 |
| OZE I | 15 | 0.116 |
| ONO S | 15 | 0.116 |
| OKUMA K | 15 | 0.116 |
| OHIRA M | 15 | 0.116 |
| OHIGASHI H | 15 | 0.116 |
| ODZE RD | 15 | 0.116 |
| NOMOTO S | 15 | 0.116 |
| NOGUCHI T | 15 | 0.116 |
| NISHIMURA K | 15 | 0.116 |
| NIEUWENHUIJZEN GAP | 15 | 0.116 |
| MIYASHIRO I | 15 | 0.116 |
| MINE S | 15 | 0.116 |
| MIMORI K | 15 | 0.116 |
| MEIJER SL | 15 | 0.116 |
| MATSUSHITA H | 15 | 0.116 |
| MARTINELLI E | 15 | 0.116 |
| MALFERTHEINER P | 15 | 0.116 |
| LI T | 15 | 0.116 |
| LENZ HJ | 15 | 0.116 |
| KUTUP A | 15 | 0.116 |
| KOSAKA T | 15 | 0.116 |
| KOONG AC | 15 | 0.116 |
| KONSKI AA | 15 | 0.116 |
| KOMAKI RU | 15 | 0.116 |
| KOIKE T | 15 | 0.116 |
| KENJO M | 15 | 0.116 |
| JAGER D | 15 | 0.116 |
| HSU CH | 15 | 0.116 |
| HERMAN JG | 15 | 0.116 |
| HATOOKA S | 15 | 0.116 |
| HAGEN JA | 15 | 0.116 |
| GUO YL | 15 | 0.116 |
| GU J | 15 | 0.116 |
| GREENWALD BD | 15 | 0.116 |
| DOI T | 15 | 0.116 |
| DIGGS BS | 15 | 0.116 |
| DE MANZONI G | 15 | 0.116 |
| CHUONG MD | 15 | 0.116 |
| CHEN W | 15 | 0.116 |
| CHEN JS | 15 | 0.116 |
| CHAU I | 15 | 0.116 |
| CHAN KW | 15 | 0.116 |
| BERRY MF | 15 | 0.116 |
| BEDENNE L | 15 | 0.116 |
| BAI Y | 15 | 0.116 |
| AOKI Y | 15 | 0.116 |
| ANDO T | 15 | 0.116 |
| ZHENG RS | 14 | 0.108 |
| ZHAO YP | 14 | 0.108 |
| ZHANG ZF | 14 | 0.108 |
| ZHANG XM | 14 | 0.108 |
| ZHANG JY | 14 | 0.108 |
| YOSHIMURA K | 14 | 0.108 |
| YIN LH | 14 | 0.108 |
| YE WM | 14 | 0.108 |
| YANG PW | 14 | 0.108 |
| YAMAMOTO T | 14 | 0.108 |
| XUE LY | 14 | 0.108 |
| XU XC | 14 | 0.108 |
| XU LY | 14 | 0.108 |
| WEICHERT W | 14 | 0.108 |
| WANG WL | 14 | 0.108 |
| WANG JB | 14 | 0.108 |
| WANG HM | 14 | 0.108 |
| WANG B | 14 | 0.108 |
| WALSH GL | 14 | 0.108 |
| VAN CUTSEM E | 14 | 0.108 |
| UCHIDA E | 14 | 0.108 |
| TANG L | 14 | 0.108 |
| TAKIZAWA K | 14 | 0.108 |
| SHIOYAMA Y | 14 | 0.108 |
| SHIM YM | 14 | 0.108 |
| SHIBATA T | 14 | 0.108 |
| SHEPHARD GS | 14 | 0.108 |
| SHAO Q | 14 | 0.108 |
| SHAO AZ | 14 | 0.108 |
| SENNINGER N | 14 | 0.108 |
| SCHWEIGERT M | 14 | 0.108 |
| SANO T | 14 | 0.108 |
| SAKAMOTO N | 14 | 0.108 |
| ROSS WA | 14 | 0.108 |
| ROSHANDEL G | 14 | 0.108 |
| REICHELT U | 14 | 0.108 |
| PLASTARAS JP | 14 | 0.108 |
| PHILLIPS WA | 14 | 0.108 |
| PAUL S | 14 | 0.108 |
| PANDEYA N | 14 | 0.108 |
| OYAMA K | 14 | 0.108 |
| NGUYEN NT | 14 | 0.108 |
| NASROLLAHZADEH D | 14 | 0.108 |
| NAKANO T | 14 | 0.108 |
| NAGAI Y | 14 | 0.108 |
| MONDEN M | 14 | 0.108 |
| MAY A | 14 | 0.108 |
| MATSUSHITA K | 14 | 0.108 |
| MATSUDA T | 14 | 0.108 |
| MATONO S | 14 | 0.108 |
| MARU D | 14 | 0.108 |
| MARTIN RCG | 14 | 0.108 |
| MANNER H | 14 | 0.108 |
| LIU CY | 14 | 0.108 |
| LI R | 14 | 0.108 |
| LI JJ | 14 | 0.108 |
| LI HC | 14 | 0.108 |
| LEE KH | 14 | 0.108 |
| KUWABARA Y | 14 | 0.108 |
| KUBO A | 14 | 0.108 |
| KRAUSE BJ | 14 | 0.108 |
| KOZOWER BD | 14 | 0.108 |
| KOSUGI S | 14 | 0.108 |
| KOKUBA Y | 14 | 0.108 |
| KATAYAMA H | 14 | 0.108 |
| KANG YK | 14 | 0.108 |
| JIN Z | 14 | 0.108 |
| JIANG JT | 14 | 0.108 |
| ISOMOTO H | 14 | 0.108 |
| INOUE M | 14 | 0.108 |
| IKEDA H | 14 | 0.108 |
| HSU WH | 14 | 0.108 |
| HOFLER H | 14 | 0.108 |
| HIKI N | 14 | 0.108 |
| HE YT | 14 | 0.108 |
| HAYASHI H | 14 | 0.108 |
| HASEGAWA Y | 14 | 0.108 |
| HARMON JW | 14 | 0.108 |
| HAN J | 14 | 0.108 |
| HAMAGUCHI T | 14 | 0.108 |
| GUO H | 14 | 0.108 |
| GRIFFIN SM | 14 | 0.108 |
| GOTO H | 14 | 0.108 |
| GISBERTZ SS | 14 | 0.108 |
| GIFFEN C | 14 | 0.108 |
| GHOSHAL UC | 14 | 0.108 |
| FU L | 14 | 0.108 |
| DESCHAMPS C | 14 | 0.108 |
| DAS P | 14 | 0.108 |
| DAS A | 14 | 0.108 |
| CRANE CH | 14 | 0.108 |
| CHENG Y | 14 | 0.108 |
| CHEN YZ | 14 | 0.108 |
| CHEN JY | 14 | 0.108 |
| CHEN HY | 14 | 0.108 |
| CHEN H | 14 | 0.108 |
| CHANG JY | 14 | 0.108 |
| CHANG J | 14 | 0.108 |
| CAPANU M | 14 | 0.108 |
| BUSCH ORC | 14 | 0.108 |
| BOCKHORN M | 14 | 0.108 |
| ALLUM WH | 14 | 0.108 |
| ALLEN MS | 14 | 0.108 |
| AHN JY | 14 | 0.108 |
| ZHU HC | 13 | 0.1 |
| ZHU H | 13 | 0.1 |
| ZHENG Y | 13 | 0.1 |
| ZHANG YF | 13 | 0.1 |
| ZHANG JX | 13 | 0.1 |
| ZHANG GH | 13 | 0.1 |
| YU H | 13 | 0.1 |
| YOSHINO K | 13 | 0.1 |
| YANG HX | 13 | 0.1 |
| YAMAMOTO H | 13 | 0.1 |
| XU YP | 13 | 0.1 |
| XU M | 13 | 0.1 |
| WRIGHT CD | 13 | 0.1 |
| WRBA F | 13 | 0.1 |
| WONG J | 13 | 0.1 |
| WEI W | 13 | 0.1 |
| WEBER JM | 13 | 0.1 |
| WEBB PM | 13 | 0.1 |
| WANG YQ | 13 | 0.1 |
| WANG YJ | 13 | 0.1 |
| WANG XM | 13 | 0.1 |
| WANG WP | 13 | 0.1 |
| WANG TY | 13 | 0.1 |
| WANG RW | 13 | 0.1 |
| WANG LS | 13 | 0.1 |
| WANG G | 13 | 0.1 |
| WADA H | 13 | 0.1 |
| VAN SANDICK JW | 13 | 0.1 |
| UEDA S | 13 | 0.1 |
| TRIBOULET JP | 13 | 0.1 |
| TIAN DP | 13 | 0.1 |
| TAKAHARI D | 13 | 0.1 |
| TAKAGAWA R | 13 | 0.1 |
| SUN Q | 13 | 0.1 |
| STRONG VE | 13 | 0.1 |
| SPRANGERS MAG | 13 | 0.1 |
| SOTOUDEH M | 13 | 0.1 |
| SMYTH EC | 13 | 0.1 |
| SHIMODA T | 13 | 0.1 |
| SEITZ JF | 13 | 0.1 |
| SCHURR PG | 13 | 0.1 |
| SCHIPPER PH | 13 | 0.1 |
| SAXENA S | 13 | 0.1 |
| SAKAGUCHI Y | 13 | 0.1 |
| SAITO S | 13 | 0.1 |
| ROVIELLO F | 13 | 0.1 |
| PU YP | 13 | 0.1 |
| PRATSCHKE J | 13 | 0.1 |
| PARK CH | 13 | 0.1 |
| ONO H | 13 | 0.1 |
| OJIMA T | 13 | 0.1 |
| OHUE M | 13 | 0.1 |
| OHI M | 13 | 0.1 |
| OGAWA R | 13 | 0.1 |
| NOMURA S | 13 | 0.1 |
| NOMA K | 13 | 0.1 |
| NISHIMAKI T | 13 | 0.1 |
| NAKAMORI M | 13 | 0.1 |
| MUIJS CT | 13 | 0.1 |
| MROCZKO B | 13 | 0.1 |
| MOLENA D | 13 | 0.1 |
| MASON DP | 13 | 0.1 |
| MARUYAMA T | 13 | 0.1 |
| MALHOTRA U | 13 | 0.1 |
| MACAPINLAC HA | 13 | 0.1 |
| LU SH | 13 | 0.1 |
| LIU Z | 13 | 0.1 |
| LITLE VR | 13 | 0.1 |
| LIPHAM JC | 13 | 0.1 |
| LING ZQ | 13 | 0.1 |
| LI EM | 13 | 0.1 |
| LEE KW | 13 | 0.1 |
| LEE HS | 13 | 0.1 |
| KONO T | 13 | 0.1 |
| KOIKE M | 13 | 0.1 |
| KIM S | 13 | 0.1 |
| KIM K | 13 | 0.1 |
| KIM JJ | 13 | 0.1 |
| KE Y | 13 | 0.1 |
| KATADA T | 13 | 0.1 |
| KAPUR S | 13 | 0.1 |
| KALININ V | 13 | 0.1 |
| KAKUSHIMA N | 13 | 0.1 |
| JUNGINGER T | 13 | 0.1 |
| JIANG W | 13 | 0.1 |
| ISHIKAWA T | 13 | 0.1 |
| ILSON D | 13 | 0.1 |
| HU N | 13 | 0.1 |
| HU JM | 13 | 0.1 |
| HU J | 13 | 0.1 |
| HU H | 13 | 0.1 |
| HOLSCHER A | 13 | 0.1 |
| HAYASHI K | 13 | 0.1 |
| HASHIBE M | 13 | 0.1 |
| GUO XF | 13 | 0.1 |
| GUO L | 13 | 0.1 |
| GAMMON MD | 13 | 0.1 |
| GALLE PR | 13 | 0.1 |
| FUKADA J | 13 | 0.1 |
| FUJIWARA M | 13 | 0.1 |
| FARIED A | 13 | 0.1 |
| FAN JH | 13 | 0.1 |
| FALK GW | 13 | 0.1 |
| DUMOT JA | 13 | 0.1 |
| DI FIORE F | 13 | 0.1 |
| DAIGO Y | 13 | 0.1 |
| DAHAN L | 13 | 0.1 |
| CUESTA MA | 13 | 0.1 |
| CREHANGE G | 13 | 0.1 |
| CONROY T | 13 | 0.1 |
| CHOU SH | 13 | 0.1 |
| CHOI KS | 13 | 0.1 |
| CHEUNG ALM | 13 | 0.1 |
| CHENG JCH | 13 | 0.1 |
| CHEN XX | 13 | 0.1 |
| CHEN S | 13 | 0.1 |
| CHEN K | 13 | 0.1 |
| CHEN JP | 13 | 0.1 |
| CHEN CH | 13 | 0.1 |
| CHEN CC | 13 | 0.1 |
| CHANG AC | 13 | 0.1 |
| CASSIVI SD | 13 | 0.1 |
| CAI J | 13 | 0.1 |
| BOGOEVSKI D | 13 | 0.1 |
| BOEING H | 13 | 0.1 |
| BENTREM DJ | 13 | 0.1 |
| BAIRD A | 13 | 0.1 |
| BAINS MS | 13 | 0.1 |
| ADENIS A | 13 | 0.1 |
| ZHU HX | 12 | 0.092 |
| ZHAO YF | 12 | 0.092 |
| ZHAO XH | 12 | 0.092 |
| ZHANG XH | 12 | 0.092 |
| ZHANG SY | 12 | 0.092 |
| ZHANG R | 12 | 0.092 |
| ZHANG LW | 12 | 0.092 |
| ZHANG HX | 12 | 0.092 |
| ZANG WQ | 12 | 0.092 |
| YOSHIDA S | 12 | 0.092 |
| YOSHIDA R | 12 | 0.092 |
| YOON HH | 12 | 0.092 |
| YAMAMOTO N | 12 | 0.092 |
| XU H | 12 | 0.092 |
| WU MS | 12 | 0.092 |
| WILKE H | 12 | 0.092 |
| WATANABE T | 12 | 0.092 |
| WANG YL | 12 | 0.092 |
| WANG XH | 12 | 0.092 |
| WANG XF | 12 | 0.092 |
| WANG T | 12 | 0.092 |
| WANG JH | 12 | 0.092 |
| WANG HB | 12 | 0.092 |
| WANG BY | 12 | 0.092 |
| VISVIKIS D | 12 | 0.092 |
| VAN HEIJL M | 12 | 0.092 |
| USAMI S | 12 | 0.092 |
| UENOSONO Y | 12 | 0.092 |
| TUMINO R | 12 | 0.092 |
| TRICHOPOULOU A | 12 | 0.092 |
| TJONNELAND A | 12 | 0.092 |
| TANAKA F | 12 | 0.092 |
| TANAKA E | 12 | 0.092 |
| TAN W | 12 | 0.092 |
| TAJIMA K | 12 | 0.092 |
| SUZUKI Y | 12 | 0.092 |
| SUN X | 12 | 0.092 |
| SUGIMURA K | 12 | 0.092 |
| SU L | 12 | 0.092 |
| STOECKLEIN NH | 12 | 0.092 |
| SINGH S | 12 | 0.092 |
| SHIN SK | 12 | 0.092 |
| SHIMOSEGAWA T | 12 | 0.092 |
| SHIMOJI H | 12 | 0.092 |
| SAUTER G | 12 | 0.092 |
| SATO N | 12 | 0.092 |
| SALGIA R | 12 | 0.092 |
| SAKURAMA K | 12 | 0.092 |
| SAKURAI K | 12 | 0.092 |
| SADANAGA N | 12 | 0.092 |
| ROTH JA | 12 | 0.092 |
| ROBERTS SA | 12 | 0.092 |
| RISCH HA | 12 | 0.092 |
| RINKES IHMB | 12 | 0.092 |
| REDDY RM | 12 | 0.092 |
| PULI SR | 12 | 0.092 |
| OSHIMA T | 12 | 0.092 |
| ONODERA K | 12 | 0.092 |
| ONO HA | 12 | 0.092 |
| OJIMA H | 12 | 0.092 |
| OHTOMO K | 12 | 0.092 |
| OHGA S | 12 | 0.092 |
| NOMURA T | 12 | 0.092 |
| NAKAYAMA G | 12 | 0.092 |
| NAKANISHI Y | 12 | 0.092 |
| MUGURUMA K | 12 | 0.092 |
| MIZUMOTO M | 12 | 0.092 |
| MIYATA G | 12 | 0.092 |
| MIYAMOTO S | 12 | 0.092 |
| MITSUI A | 12 | 0.092 |
| MEYER HJ | 12 | 0.092 |
| MEUNIER B | 12 | 0.092 |
| MASUDA N | 12 | 0.092 |
| MAMON HJ | 12 | 0.092 |
| MA Z | 12 | 0.092 |
| LU Y | 12 | 0.092 |
| LIU W | 12 | 0.092 |
| LIU SG | 12 | 0.092 |
| LIU RP | 12 | 0.092 |
| LIGHTDALE CJ | 12 | 0.092 |
| LI Z | 12 | 0.092 |
| LI XH | 12 | 0.092 |
| LEE SK | 12 | 0.092 |
| LEE H | 12 | 0.092 |
| LAUWERS GY | 12 | 0.092 |
| LAUDANSKI J | 12 | 0.092 |
| KUSUNOKI M | 12 | 0.092 |
| KOSUGA T | 12 | 0.092 |
| KONDO T | 12 | 0.092 |
| KONDO S | 12 | 0.092 |
| KNOX JJ | 12 | 0.092 |
| KIMURA T | 12 | 0.092 |
| JIN L | 12 | 0.092 |
| JIN J | 12 | 0.092 |
| JIANG H | 12 | 0.092 |
| JAIN M | 12 | 0.092 |
| ITO M | 12 | 0.092 |
| IKEDA K | 12 | 0.092 |
| IIJIMA K | 12 | 0.092 |
| HUSSEY DJ | 12 | 0.092 |
| HUNG MC | 12 | 0.092 |
| HUANG W | 12 | 0.092 |
| HUANG PM | 12 | 0.092 |
| HSIEH CC | 12 | 0.092 |
| HAN C | 12 | 0.092 |
| HAGIWARA N | 12 | 0.092 |
| GALIZIA G | 12 | 0.092 |
| FUJITA M | 12 | 0.092 |
| FUJIMURA T | 12 | 0.092 |
| FUCHS CS | 12 | 0.092 |
| FU XL | 12 | 0.092 |
| FRANCESCHI D | 12 | 0.092 |
| FANG WT | 12 | 0.092 |
| FAKHRIAN K | 12 | 0.092 |
| D'JOURNO XB | 12 | 0.092 |
| CUI XB | 12 | 0.092 |
| COIMBRA R | 12 | 0.092 |
| CHIN K | 12 | 0.092 |
| CHENG J | 12 | 0.092 |
| CHEN Z | 12 | 0.092 |
| CHEN LJ | 12 | 0.092 |
| CATS A | 12 | 0.092 |
| BUENO-DE-MESQUITA HB | 12 | 0.092 |
| BOOT H | 12 | 0.092 |
| BOONE J | 12 | 0.092 |
| BERNSTEIN L | 12 | 0.092 |
| BECHTOLD ML | 12 | 0.092 |
| BANSAL A | 12 | 0.092 |
| BANG YJ | 12 | 0.092 |
| AZUMA M | 12 | 0.092 |
| ARIGAMI T | 12 | 0.092 |
| ARIGA H | 12 | 0.092 |
| ANTILLON MR | 12 | 0.092 |
| AMADORI A | 12 | 0.092 |
| ADLER DG | 12 | 0.092 |
| ZHU Y | 11 | 0.085 |
| ZHOU T | 11 | 0.085 |
| ZHOU Q | 11 | 0.085 |
| ZHENG W | 11 | 0.085 |
| ZHAO X | 11 | 0.085 |
| ZHAO P | 11 | 0.085 |
| ZHANG XL | 11 | 0.085 |
| ZHANG HF | 11 | 0.085 |
| YAMASHITA S | 11 | 0.085 |
| YAMAGUCHI N | 11 | 0.085 |
| XU NZ | 11 | 0.085 |
| WU M | 11 | 0.085 |
| WU H | 11 | 0.085 |
| WERNER M | 11 | 0.085 |
| WEN DG | 11 | 0.085 |
| WELSH JW | 11 | 0.085 |
| WATANABE S | 11 | 0.085 |
| WATANABE G | 11 | 0.085 |
| WANG ZQ | 11 | 0.085 |
| WANG YH | 11 | 0.085 |
| WANG CP | 11 | 0.085 |
| VAN DEKKEN H | 11 | 0.085 |
| UEDA M | 11 | 0.085 |
| TSAI JA | 11 | 0.085 |
| TOMITA N | 11 | 0.085 |
| THUSS-PATIENCE P | 11 | 0.085 |
| TESHIMA T | 11 | 0.085 |
| TATSUTA M | 11 | 0.085 |
| TAN LJ | 11 | 0.085 |
| TAKASE K | 11 | 0.085 |
| TAKAMURA H | 11 | 0.085 |
| TAKAHASHI K | 11 | 0.085 |
| SUN Y | 11 | 0.085 |
| SUGIYAMA T | 11 | 0.085 |
| SUGIMOTO H | 11 | 0.085 |
| SUGAWARA T | 11 | 0.085 |
| STONER GD | 11 | 0.085 |
| STILES BM | 11 | 0.085 |
| SPECHLER SJ | 11 | 0.085 |
| SPAANDER MCW | 11 | 0.085 |
| SINGH A | 11 | 0.085 |
| SHIKU H | 11 | 0.085 |
| SAXTON JP | 11 | 0.085 |
| SASAKI Y | 11 | 0.085 |
| SASAKI S | 11 | 0.085 |
| SAGGIORO D | 11 | 0.085 |
| RUMIATO E | 11 | 0.085 |
| RUHSTALLER T | 11 | 0.085 |
| ROSATO EL | 11 | 0.085 |
| ROBB WB | 11 | 0.085 |
| RIEGLER M | 11 | 0.085 |
| RIBOLI E | 11 | 0.085 |
| PRAMESH CS | 11 | 0.085 |
| POSNER MC | 11 | 0.085 |
| PINTO LFR | 11 | 0.085 |
| PENG J | 11 | 0.085 |
| PARK YS | 11 | 0.085 |
| PARK JC | 11 | 0.085 |
| OVERVAD K | 11 | 0.085 |
| OTA M | 11 | 0.085 |
| OKAMURA T | 11 | 0.085 |
| OHASHI T | 11 | 0.085 |
| NUNOBE S | 11 | 0.085 |
| NILSSON M | 11 | 0.085 |
| NASON KS | 11 | 0.085 |
| NAKASHIMA T | 11 | 0.085 |
| NAKAMATSU K | 11 | 0.085 |
| NAKAGAWA S | 11 | 0.085 |
| NAFTEUX P | 11 | 0.085 |
| NABEYA Y | 11 | 0.085 |
| MOONS J | 11 | 0.085 |
| MOHAN R | 11 | 0.085 |
| MIYASHITA M | 11 | 0.085 |
| MINSKY BD | 11 | 0.085 |
| MIMURA K | 11 | 0.085 |
| METZ JM | 11 | 0.085 |
| MESSMANN H | 11 | 0.085 |
| MCCOLL KEL | 11 | 0.085 |
| MATTSSON F | 11 | 0.085 |
| MATSUTANI T | 11 | 0.085 |
| MAKOWIEC F | 11 | 0.085 |
| MAKINO I | 11 | 0.085 |
| MAHANTA J | 11 | 0.085 |
| MA GW | 11 | 0.085 |
| LU XM | 11 | 0.085 |
| LU W | 11 | 0.085 |
| LONG H | 11 | 0.085 |
| LOCKHART AC | 11 | 0.085 |
| LIU JS | 11 | 0.085 |
| LIU HB | 11 | 0.085 |
| LIU CX | 11 | 0.085 |
| LI XM | 11 | 0.085 |
| LI SH | 11 | 0.085 |
| LI JP | 11 | 0.085 |
| LI JL | 11 | 0.085 |
| LI BS | 11 | 0.085 |
| LEVI F | 11 | 0.085 |
| LEE JY | 11 | 0.085 |
| KOSUMI K | 11 | 0.085 |
| KOBAYASHI S | 11 | 0.085 |
| KO JY | 11 | 0.085 |
| KLEINBERG LR | 11 | 0.085 |
| KLEIN-SZANTO AJ | 11 | 0.085 |
| KITAGAWA H | 11 | 0.085 |
| KIMURA H | 11 | 0.085 |
| KIM YT | 11 | 0.085 |
| KIM HS | 11 | 0.085 |
| KIM H | 11 | 0.085 |
| KIKUCHI S | 11 | 0.085 |
| KATSUDA M | 11 | 0.085 |
| KATADA N | 11 | 0.085 |
| KAGAWA S | 11 | 0.085 |
| JONES DR | 11 | 0.085 |
| JIANG YG | 11 | 0.085 |
| JACOBS M | 11 | 0.085 |
| IWAHASHI M | 11 | 0.085 |
| ITO S | 11 | 0.085 |
| ISHII H | 11 | 0.085 |
| HUMMEL R | 11 | 0.085 |
| HUANG CY | 11 | 0.085 |
| HU X | 11 | 0.085 |
| HU JK | 11 | 0.085 |
| HSIEH MJ | 11 | 0.085 |
| HORI K | 11 | 0.085 |
| HOPPO T | 11 | 0.085 |
| HOLLENBECK AR | 11 | 0.085 |
| HAYMAN JA | 11 | 0.085 |
| HAYASHI N | 11 | 0.085 |
| HASEGAWA S | 11 | 0.085 |
| GRIMMINGER PP | 11 | 0.085 |
| GRENACHER L | 11 | 0.085 |
| GOLDSTEIN AM | 11 | 0.085 |
| GOAN YG | 11 | 0.085 |
| GEINITZ H | 11 | 0.085 |
| GAO YT | 11 | 0.085 |
| FUKAI Y | 11 | 0.085 |
| FUJITA T | 11 | 0.085 |
| FRANCESCHI S | 11 | 0.085 |
| FRAKES JM | 11 | 0.085 |
| FONTAINE J | 11 | 0.085 |
| FENG MX | 11 | 0.085 |
| DUHAMEL A | 11 | 0.085 |
| CHEN ZX | 11 | 0.085 |
| CHEN ZF | 11 | 0.085 |
| CHEN WH | 11 | 0.085 |
| CHEN M | 11 | 0.085 |
| CHEN B | 11 | 0.085 |
| CHANG CY | 11 | 0.085 |
| BURROWS W | 11 | 0.085 |
| BUCHLER MW | 11 | 0.085 |
| ATSUMI K | 11 | 0.085 |
| ASARI R | 11 | 0.085 |
| ARNOLD D | 11 | 0.085 |
| ALAKUS H | 11 | 0.085 |
| ZHU J | 10 | 0.077 |
| ZHENG ST | 10 | 0.077 |
| ZHAO W | 10 | 0.077 |
| ZHAO Q | 10 | 0.077 |
| ZHANG XF | 10 | 0.077 |
| ZHANG XD | 10 | 0.077 |
| ZHANG WH | 10 | 0.077 |
| ZHANG K | 10 | 0.077 |
| ZHANG JH | 10 | 0.077 |
| ZHANG G | 10 | 0.077 |
| ZHANG CY | 10 | 0.077 |
| YOSHINAGA K | 10 | 0.077 |
| YOON DH | 10 | 0.077 |
| YCHOU M | 10 | 0.077 |
| YASHIRO M | 10 | 0.077 |
| YANG YH | 10 | 0.077 |
| YANG S | 10 | 0.077 |
| YANG HK | 10 | 0.077 |
| YANG GY | 10 | 0.077 |
| YANG F | 10 | 0.077 |
| YAMAZAKI K | 10 | 0.077 |
| XU XQ | 10 | 0.077 |
| XIE D | 10 | 0.077 |
| WU X | 10 | 0.077 |
| WU L | 10 | 0.077 |
| WU HY | 10 | 0.077 |
| WU CY | 10 | 0.077 |
| WU CP | 10 | 0.077 |
| WU AJ | 10 | 0.077 |
| WU AH | 10 | 0.077 |
| WONG RKS | 10 | 0.077 |
| WEI QY | 10 | 0.077 |
| WATANABE Y | 10 | 0.077 |
| WANI S | 10 | 0.077 |
| WANG YD | 10 | 0.077 |
| WANG XY | 10 | 0.077 |
| WANG R | 10 | 0.077 |
| WANG KJ | 10 | 0.077 |
| WANG JZ | 10 | 0.077 |
| WANG JW | 10 | 0.077 |
| WANG JL | 10 | 0.077 |
| WANG HY | 10 | 0.077 |
| VIDETIC GMM | 10 | 0.077 |
| VERHAGE RJJ | 10 | 0.077 |
| VAN HAGEN P | 10 | 0.077 |
| VAN DULLEMEN HM | 10 | 0.077 |
| VAN DER WESTHUIZEN L | 10 | 0.077 |
| VAN DER SLUIS PC | 10 | 0.077 |
| TONG DKH | 10 | 0.077 |
| TOMITA Y | 10 | 0.077 |
| TIAN H | 10 | 0.077 |
| THEISEN J | 10 | 0.077 |
| TAKEYAMA H | 10 | 0.077 |
| TAKANAMI K | 10 | 0.077 |
| TAJIMA H | 10 | 0.077 |
| TABERNERO J | 10 | 0.077 |
| SUN YH | 10 | 0.077 |
| SUN XB | 10 | 0.077 |
| STANGE A | 10 | 0.077 |
| SONG KY | 10 | 0.077 |
| SOLYMOSI N | 10 | 0.077 |
| SHIMIZU Y | 10 | 0.077 |
| SHIBATA S | 10 | 0.077 |
| SHEN Y | 10 | 0.077 |
| SHARMA A | 10 | 0.077 |
| SCHMIDT M | 10 | 0.077 |
| SCHILD SE | 10 | 0.077 |
| SATOH T | 10 | 0.077 |
| SAKURAI T | 10 | 0.077 |
| SAITO T | 10 | 0.077 |
| RUBENSTEIN JH | 10 | 0.077 |
| ROSCH T | 10 | 0.077 |
| RODRIGUEZ CP | 10 | 0.077 |
| RIVERA F | 10 | 0.077 |
| REED CE | 10 | 0.077 |
| QIN Q | 10 | 0.077 |
| PESCHEL C | 10 | 0.077 |
| PARKER MI | 10 | 0.077 |
| PALMES D | 10 | 0.077 |
| OKADA T | 10 | 0.077 |
| OKADA H | 10 | 0.077 |
| OGURA M | 10 | 0.077 |
| OEZCELIK A | 10 | 0.077 |
| OCHIAI A | 10 | 0.077 |
| NOVOTNY A | 10 | 0.077 |
| NOURA S | 10 | 0.077 |
| NONAKA S | 10 | 0.077 |
| NOMURA F | 10 | 0.077 |
| NISHIDA T | 10 | 0.077 |
| NICHOLS FC | 10 | 0.077 |
| NAKAO K | 10 | 0.077 |
| NAGATA M | 10 | 0.077 |
| NAGANO T | 10 | 0.077 |
| MULDOON C | 10 | 0.077 |
| MUL VE | 10 | 0.077 |
| MORGILLO F | 10 | 0.077 |
| MOORE MA | 10 | 0.077 |
| MOHRI Y | 10 | 0.077 |
| MIURA M | 10 | 0.077 |
| MITTAL SK | 10 | 0.077 |
| MEYERS BF | 10 | 0.077 |
| MEYER JE | 10 | 0.077 |
| METGES JP | 10 | 0.077 |
| MESSAGER M | 10 | 0.077 |
| MASHIMO H | 10 | 0.077 |
| MARRELLI D | 10 | 0.077 |
| MALIK MA | 10 | 0.077 |
| MAKUUCHI H | 10 | 0.077 |
| LU YX | 10 | 0.077 |
| LIU YC | 10 | 0.077 |
| LIU S | 10 | 0.077 |
| LIU P | 10 | 0.077 |
| LIU MZ | 10 | 0.077 |
| LIU K | 10 | 0.077 |
| LIU HM | 10 | 0.077 |
| LIU CC | 10 | 0.077 |
| LIN Q | 10 | 0.077 |
| LIN L | 10 | 0.077 |
| LIAO J | 10 | 0.077 |
| LI ZG | 10 | 0.077 |
| LI XQ | 10 | 0.077 |
| LI XL | 10 | 0.077 |
| LI SJ | 10 | 0.077 |
| LI SG | 10 | 0.077 |
| LI D | 10 | 0.077 |
| LEE SH | 10 | 0.077 |
| LEE KD | 10 | 0.077 |
| LEE CT | 10 | 0.077 |
| LANGENDIJK JA | 10 | 0.077 |
| LAGIOU P | 10 | 0.077 |
| KWAK EL | 10 | 0.077 |
| KUKAR M | 10 | 0.077 |
| KUBOZONO M | 10 | 0.077 |
| KUANG G | 10 | 0.077 |
| KOMATSU Y | 10 | 0.077 |
| KIM MS | 10 | 0.077 |
| KIM HJ | 10 | 0.077 |
| KIM GH | 10 | 0.077 |
| KHAW KT | 10 | 0.077 |
| KESWANI RN | 10 | 0.077 |
| KAWAHARA Y | 10 | 0.077 |
| KATSUBE T | 10 | 0.077 |
| KARIMATA H | 10 | 0.077 |
| KAO CH | 10 | 0.077 |
| KANZAKI H | 10 | 0.077 |
| KANDA M | 10 | 0.077 |
| JIANG GL | 10 | 0.077 |
| JANKOWSKI J | 10 | 0.077 |
| IYER PG | 10 | 0.077 |
| IVES DI | 10 | 0.077 |
| ISHIKURA S | 10 | 0.077 |
| ISHIGURO S | 10 | 0.077 |
| ISHIDO K | 10 | 0.077 |
| IQBAL S | 10 | 0.077 |
| IIDA M | 10 | 0.077 |
| IBDAH JA | 10 | 0.077 |
| HUANG CS | 10 | 0.077 |
| HUANG B | 10 | 0.077 |
| HU B | 10 | 0.077 |
| HIRAKAWA M | 10 | 0.077 |
| HENSELMANS I | 10 | 0.077 |
| HAYATA K | 10 | 0.077 |
| HARUTA S | 10 | 0.077 |
| HARA K | 10 | 0.077 |
| GUO Q | 10 | 0.077 |
| GROS SJ | 10 | 0.077 |
| GRABOWSKI K | 10 | 0.077 |
| GONZALEZ CA | 10 | 0.077 |
| GOLDBLUM JR | 10 | 0.077 |
| GERTLER R | 10 | 0.077 |
| GAO L | 10 | 0.077 |
| FUJITA Y | 10 | 0.077 |
| FUJII S | 10 | 0.077 |
| FU Z | 10 | 0.077 |
| FORASTIERE AA | 10 | 0.077 |
| FOCKENS P | 10 | 0.077 |
| FENG XS | 10 | 0.077 |
| FANG Y | 10 | 0.077 |
| FAN XS | 10 | 0.077 |
| FAGUNDES RB | 10 | 0.077 |
| ESCOFET X | 10 | 0.077 |
| EL-SERAG HB | 10 | 0.077 |
| DONAHUE JM | 10 | 0.077 |
| DING F | 10 | 0.077 |
| DAVISON JM | 10 | 0.077 |
| CHRISTOS P | 10 | 0.077 |
| CHRISTIANI DC | 10 | 0.077 |
| CHEN XZ | 10 | 0.077 |
| CHEN WC | 10 | 0.077 |
| CHEN KN | 10 | 0.077 |
| CHEN D | 10 | 0.077 |
| BUCHLER M | 10 | 0.077 |
| BOUCHE O | 10 | 0.077 |
| BLANKE CD | 10 | 0.077 |
| BERGER AC | 10 | 0.077 |
| BEN-DAVID K | 10 | 0.077 |
| BARR H | 10 | 0.077 |
| BARBOUR AP | 10 | 0.077 |
| BA-SSALAMAH A | 10 | 0.077 |
| ASOMANING K | 10 | 0.077 |
| ALLEN PK | 10 | 0.077 |
| ZHU ST | 9 | 0.069 |
| ZHU SC | 9 | 0.069 |
| ZHOU Z | 9 | 0.069 |
| ZHOU YF | 9 | 0.069 |
| ZHOU XF | 9 | 0.069 |
| ZHAO BS | 9 | 0.069 |
| ZHANG YQ | 9 | 0.069 |
| ZHANG LH | 9 | 0.069 |
| ZENG HM | 9 | 0.069 |
| ZEHETNER J | 9 | 0.069 |
| ZARGAR SA | 9 | 0.069 |
| YUAN L | 9 | 0.069 |
| YOSHINO T | 9 | 0.069 |
| YOSHII T | 9 | 0.069 |
| YODA Y | 9 | 0.069 |
| YANG Q | 9 | 0.069 |
| YAN X | 9 | 0.069 |
| YAMAGUCHI S | 9 | 0.069 |
| XU YJ | 9 | 0.069 |
| XU XL | 9 | 0.069 |
| XU JM | 9 | 0.069 |
| XU F | 9 | 0.069 |
| XU C | 9 | 0.069 |
| XIE X | 9 | 0.069 |
| WU KC | 9 | 0.069 |
| WU K | 9 | 0.069 |
| WU JZ | 9 | 0.069 |
| WU B | 9 | 0.069 |
| WHITE RE | 9 | 0.069 |
| WEI X | 9 | 0.069 |
| WEI L | 9 | 0.069 |
| WANG ZM | 9 | 0.069 |
| WANG ZG | 9 | 0.069 |
| WANG XW | 9 | 0.069 |
| WANG XS | 9 | 0.069 |
| WANG SK | 9 | 0.069 |
| WANG JF | 9 | 0.069 |
| WAKATSUKI K | 9 | 0.069 |
| VAN OIJEN MGH | 9 | 0.069 |
| VAN DER HORST S | 9 | 0.069 |
| UZUNOGLU FG | 9 | 0.069 |
| TSUJIMOTO H | 9 | 0.069 |
| TRIADAFILOPOULOS G | 9 | 0.069 |
| TOYOKAWA T | 9 | 0.069 |
| TOIYAMA Y | 9 | 0.069 |
| THRIFT AP | 9 | 0.069 |
| THOMAS PA | 9 | 0.069 |
| TANAKA S | 9 | 0.069 |
| TAKENO S | 9 | 0.069 |
| TAKENAKA R | 9 | 0.069 |
| TAJIKA M | 9 | 0.069 |
| SUZUKI O | 9 | 0.069 |
| SUN XJ | 9 | 0.069 |
| SUN XD | 9 | 0.069 |
| SUN GG | 9 | 0.069 |
| SUGIYAMA M | 9 | 0.069 |
| STUSCHKE M | 9 | 0.069 |
| STRATE T | 9 | 0.069 |
| SRIVASTAVA G | 9 | 0.069 |
| SONG X | 9 | 0.069 |
| SONG SM | 9 | 0.069 |
| SONG H | 9 | 0.069 |
| SOBAJIMA J | 9 | 0.069 |
| SHIRAISHI O | 9 | 0.069 |
| SHIRAI K | 9 | 0.069 |
| SHIMIZU S | 9 | 0.069 |
| SHIMAKAWA T | 9 | 0.069 |
| SHAPIRO J | 9 | 0.069 |
| SHAMI VM | 9 | 0.069 |
| SHAIKH T | 9 | 0.069 |
| SGOURAKIS G | 9 | 0.069 |
| SERRAINO D | 9 | 0.069 |
| SCHUMACHER G | 9 | 0.069 |
| SCHMIDT T | 9 | 0.069 |
| SCHATZKIN A | 9 | 0.069 |
| SATO F | 9 | 0.069 |
| SASAKO M | 9 | 0.069 |
| SASAKI H | 9 | 0.069 |
| SAITO M | 9 | 0.069 |
| ROUVELAS I | 9 | 0.069 |
| ROSSI M | 9 | 0.069 |
| REPICI A | 9 | 0.069 |
| REEH M | 9 | 0.069 |
| RASTOGI A | 9 | 0.069 |
| QUIROS JR | 9 | 0.069 |
| QI B | 9 | 0.069 |
| PULTRUM BB | 9 | 0.069 |
| PORTALE G | 9 | 0.069 |
| PERRY KA | 9 | 0.069 |
| PARK SY | 9 | 0.069 |
| OSAKA Y | 9 | 0.069 |
| OKABE H | 9 | 0.069 |
| OKA M | 9 | 0.069 |
| NYREN O | 9 | 0.069 |
| NOZAKI I | 9 | 0.069 |
| NISHIO M | 9 | 0.069 |
| NISHIKAWA K | 9 | 0.069 |
| NISHI T | 9 | 0.069 |
| NAVA HR | 9 | 0.069 |
| NARITAKA Y | 9 | 0.069 |
| NAKAMURA S | 9 | 0.069 |
| NAKAGAWARA H | 9 | 0.069 |
| NAKAGAWA M | 9 | 0.069 |
| NAGATA T | 9 | 0.069 |
| MORTENSEN MB | 9 | 0.069 |
| MORITA S | 9 | 0.069 |
| MORIMURA R | 9 | 0.069 |
| MOHAMMAD NH | 9 | 0.069 |
| MITSUMORI M | 9 | 0.069 |
| MIRABEL X | 9 | 0.069 |
| MIDDLETON MR | 9 | 0.069 |
| MEIJER GJ | 9 | 0.069 |
| MATSUMOTO Y | 9 | 0.069 |
| MANN O | 9 | 0.069 |
| MAINGON P | 9 | 0.069 |
| MAEDA K | 9 | 0.069 |
| MABRUT JY | 9 | 0.069 |
| MA YY | 9 | 0.069 |
| LUO AP | 9 | 0.069 |
| LIU YT | 9 | 0.069 |
| LIU YP | 9 | 0.069 |
| LIU SH | 9 | 0.069 |
| LIU JY | 9 | 0.069 |
| LIU JF | 9 | 0.069 |
| LING Y | 9 | 0.069 |
| LIN CH | 9 | 0.069 |
| LIN C | 9 | 0.069 |
| LIANG WH | 9 | 0.069 |
| LI YY | 9 | 0.069 |
| LI XN | 9 | 0.069 |
| LI XJ | 9 | 0.069 |
| LI XF | 9 | 0.069 |
| LI SY | 9 | 0.069 |
| LI SP | 9 | 0.069 |
| LI HY | 9 | 0.069 |
| LEE JE | 9 | 0.069 |
| LASSMANN S | 9 | 0.069 |
| LANDRENEAU RJ | 9 | 0.069 |
| KUSHIMA R | 9 | 0.069 |
| KUMAR A | 9 | 0.069 |
| KRUEL CDP | 9 | 0.069 |
| KOMANDURI S | 9 | 0.069 |
| KOIKE R | 9 | 0.069 |
| KOBAYASHI N | 9 | 0.069 |
| KINOSHITA Y | 9 | 0.069 |
| KINOSHITA J | 9 | 0.069 |
| KIM HK | 9 | 0.069 |
| KIM HH | 9 | 0.069 |
| KIM BS | 9 | 0.069 |
| KII T | 9 | 0.069 |
| KATO Y | 9 | 0.069 |
| KASHTAN H | 9 | 0.069 |
| KARL R | 9 | 0.069 |
| KARAOGLANOGLU N | 9 | 0.069 |
| KANO M | 9 | 0.069 |
| KANG WK | 9 | 0.069 |
| KANG CH | 9 | 0.069 |
| KAMEI T | 9 | 0.069 |
| KAJIYAMA Y | 9 | 0.069 |
| KAISE M | 9 | 0.069 |
| KAGEYAMA S | 9 | 0.069 |
| KADOYA N | 9 | 0.069 |
| JUNG KW | 9 | 0.069 |
| IZZO JG | 9 | 0.069 |
| ISHIGURO T | 9 | 0.069 |
| ISHIDA H | 9 | 0.069 |
| ISHIBASHI K | 9 | 0.069 |
| INOUE Y | 9 | 0.069 |
| INOUE T | 9 | 0.069 |
| INNOCENTE R | 9 | 0.069 |
| IIZUKA T | 9 | 0.069 |
| IIDA T | 9 | 0.069 |
| HYUNG WJ | 9 | 0.069 |
| HUANG L | 9 | 0.069 |
| HU ZB | 9 | 0.069 |
| HU W | 9 | 0.069 |
| HSU FM | 9 | 0.069 |
| HOSPERS GAP | 9 | 0.069 |
| HOSOYA Y | 9 | 0.069 |
| HONMA Y | 9 | 0.069 |
| HOEPPNER J | 9 | 0.069 |
| HECHT SS | 9 | 0.069 |
| HE QY | 9 | 0.069 |
| HASIM A | 9 | 0.069 |
| HARUSTIAK T | 9 | 0.069 |
| HANSEN T | 9 | 0.069 |
| GUPTA N | 9 | 0.069 |
| GUINDI M | 9 | 0.069 |
| GROTENHUIS BA | 9 | 0.069 |
| GREEN AC | 9 | 0.069 |
| GODA K | 9 | 0.069 |
| GIULI R | 9 | 0.069 |
| GAO J | 9 | 0.069 |
| GALAN M | 9 | 0.069 |
| GAISSERT HA | 9 | 0.069 |
| FARBER JL | 9 | 0.069 |
| FAN QX | 9 | 0.069 |
| ENDO I | 9 | 0.069 |
| EFFENBERGER KE | 9 | 0.069 |
| DONOHOE CL | 9 | 0.069 |
| DERAKHSHAN MH | 9 | 0.069 |
| DEBUS J | 9 | 0.069 |
| DANG XT | 9 | 0.069 |
| DANG CX | 9 | 0.069 |
| CROCE CM | 9 | 0.069 |
| COOSEMANS W | 9 | 0.069 |
| COLLET D | 9 | 0.069 |
| CHUNG IJ | 9 | 0.069 |
| CHOI NC | 9 | 0.069 |
| CHIBA T | 9 | 0.069 |
| CHIARION-SILENI V | 9 | 0.069 |
| CHENG HY | 9 | 0.069 |
| CHEN ZJ | 9 | 0.069 |
| CHEN YC | 9 | 0.069 |
| CHEN XD | 9 | 0.069 |
| CHEN P | 9 | 0.069 |
| CHEN JH | 9 | 0.069 |
| CERFOLIO RJ | 9 | 0.069 |
| BRUNO MJ | 9 | 0.069 |
| BRUCKNER T | 9 | 0.069 |
| BRENNAN MF | 9 | 0.069 |
| BLUDAU M | 9 | 0.069 |
| BLAKE MA | 9 | 0.069 |
| BLACKSTOCK AW | 9 | 0.069 |
| BIERMANN K | 9 | 0.069 |
| BATTAFARANO RJ | 9 | 0.069 |
| BARTHEL JS | 9 | 0.069 |
| AYAZI S | 9 | 0.069 |
| ASAKA S | 9 | 0.069 |
| ANDREOU A | 9 | 0.069 |
| ALCINDOR T | 9 | 0.069 |
| ZO JI | 8 | 0.062 |
| ZHU M | 8 | 0.062 |
| ZHOU ZG | 8 | 0.062 |
| ZHOU X | 8 | 0.062 |
| ZHOU PH | 8 | 0.062 |
| ZHENG YZ | 8 | 0.062 |
| ZHENG F | 8 | 0.062 |
| ZHAO YQ | 8 | 0.062 |
| ZHAO LJ | 8 | 0.062 |
| ZHAO KL | 8 | 0.062 |
| ZHAO JM | 8 | 0.062 |
| ZHAO JK | 8 | 0.062 |
| ZHANG ZY | 8 | 0.062 |
| ZHANG YP | 8 | 0.062 |
| ZHANG YH | 8 | 0.062 |
| ZHANG XZ | 8 | 0.062 |
| ZHANG XQ | 8 | 0.062 |
| ZHANG XB | 8 | 0.062 |
| ZHANG T | 8 | 0.062 |
| ZHANG SS | 8 | 0.062 |
| ZHANG LY | 8 | 0.062 |
| ZHANG HL | 8 | 0.062 |
| ZHAI RH | 8 | 0.062 |
| ZENDA S | 8 | 0.062 |
| ZEMANOVA M | 8 | 0.062 |
| YU W | 8 | 0.062 |
| YOSHINAGA S | 8 | 0.062 |
| YOSHIDA Y | 8 | 0.062 |
| YEH CJ | 8 | 0.062 |
| YAZDANBOD A | 8 | 0.062 |
| YAO WJ | 8 | 0.062 |
| YANG ZH | 8 | 0.062 |
| YANG Z | 8 | 0.062 |
| YANG SC | 8 | 0.062 |
| YANG CS | 8 | 0.062 |
| YANG B | 8 | 0.062 |
| YAN F | 8 | 0.062 |
| YAMAMOTO J | 8 | 0.062 |
| YAGI K | 8 | 0.062 |
| XU MD | 8 | 0.062 |
| XIONG G | 8 | 0.062 |
| XIE J | 8 | 0.062 |
| WU C | 8 | 0.062 |
| WOUTERS MWJM | 8 | 0.062 |
| WO JY | 8 | 0.062 |
| WILLETT C | 8 | 0.062 |
| WERNER J | 8 | 0.062 |
| WERNER D | 8 | 0.062 |
| WEIDERPASS E | 8 | 0.062 |
| WEBER WA | 8 | 0.062 |
| WATANABE N | 8 | 0.062 |
| WASHINGTON MK | 8 | 0.062 |
| WANG YZ | 8 | 0.062 |
| WANG XR | 8 | 0.062 |
| WANG TC | 8 | 0.062 |
| WANG LX | 8 | 0.062 |
| WAIN JC | 8 | 0.062 |
| VOKES EE | 8 | 0.062 |
| VINEIS P | 8 | 0.062 |
| VILLAFLOR VM | 8 | 0.062 |
| VERA P | 8 | 0.062 |
| VAN VULPEN M | 8 | 0.062 |
| UMAR M | 8 | 0.062 |
| TSUZUKI T | 8 | 0.062 |
| TSUNODA T | 8 | 0.062 |
| TSAI SS | 8 | 0.062 |
| TOKUNAGA R | 8 | 0.062 |
| TIAN Y | 8 | 0.062 |
| THOMAS J | 8 | 0.062 |
| TAZAWA H | 8 | 0.062 |
| TAOMOTO J | 8 | 0.062 |
| TANNAPFEL A | 8 | 0.062 |
| TAN ZH | 8 | 0.062 |
| TAN BX | 8 | 0.062 |
| TAKEUCHI M | 8 | 0.062 |
| TAKASHIMA A | 8 | 0.062 |
| TAKAHATA R | 8 | 0.062 |
| TAKAHASHI N | 8 | 0.062 |
| TAHARA M | 8 | 0.062 |
| TACHIBANA H | 8 | 0.062 |
| SVENDSEN LB | 8 | 0.062 |
| SUZUKI M | 8 | 0.062 |
| SUN ZQ | 8 | 0.062 |
| SUN J | 8 | 0.062 |
| SUN B | 8 | 0.062 |
| SUDO T | 8 | 0.062 |
| STADLHUBER RJ | 8 | 0.062 |
| SOSEF MN | 8 | 0.062 |
| SONG Y | 8 | 0.062 |
| SONG S | 8 | 0.062 |
| SONG GH | 8 | 0.062 |
| SOMI MH | 8 | 0.062 |
| SMITH BR | 8 | 0.062 |
| SLOAN JA | 8 | 0.062 |
| SKEIE G | 8 | 0.062 |
| SHINOHARA M | 8 | 0.062 |
| SHINOHARA H | 8 | 0.062 |
| SHIMADA M | 8 | 0.062 |
| SHI XJ | 8 | 0.062 |
| SHI Q | 8 | 0.062 |
| SHI H | 8 | 0.062 |
| SHEPPARD BC | 8 | 0.062 |
| SHEN YX | 8 | 0.062 |
| SHEN HB | 8 | 0.062 |
| SHAH FA | 8 | 0.062 |
| SEKINE S | 8 | 0.062 |
| SCHIMANSKI CC | 8 | 0.062 |
| SCHAUER M | 8 | 0.062 |
| SATOMI S | 8 | 0.062 |
| SAMALIN E | 8 | 0.062 |
| SAKAYAUCHI T | 8 | 0.062 |
| SAKATA H | 8 | 0.062 |
| SAKAMOTO K | 8 | 0.062 |
| SADJADI A | 8 | 0.062 |
| RYAN DP | 8 | 0.062 |
| ROWLEY S | 8 | 0.062 |
| ROVIELLO G | 8 | 0.062 |
| RONCO AL | 8 | 0.062 |
| ROELOFS HMJ | 8 | 0.062 |
| RIDWELSKI K | 8 | 0.062 |
| RHEE PL | 8 | 0.062 |
| REERINK O | 8 | 0.062 |
| REDDY JBK | 8 | 0.062 |
| RASHID A | 8 | 0.062 |
| RAO S | 8 | 0.062 |
| QIN X | 8 | 0.062 |
| PUKKALA E | 8 | 0.062 |
| POWER DG | 8 | 0.062 |
| PHAROAH PD | 8 | 0.062 |
| PFEIFFER P | 8 | 0.062 |
| PETERS WHM | 8 | 0.062 |
| PELUCCHI C | 8 | 0.062 |
| PEETERS PHM | 8 | 0.062 |
| PARK Y | 8 | 0.062 |
| PARK DY | 8 | 0.062 |
| PARK DJ | 8 | 0.062 |
| OSUGI H | 8 | 0.062 |
| OSHIKIRI T | 8 | 0.062 |
| ONOZAWA M | 8 | 0.062 |
| OKADA K | 8 | 0.062 |
| OHNITA K | 8 | 0.062 |
| OHNISHI K | 8 | 0.062 |
| OHGAKI K | 8 | 0.062 |
| OHGA T | 8 | 0.062 |
| OHASHI S | 8 | 0.062 |
| NISHIYAMA K | 8 | 0.062 |
| NISHIKAWA T | 8 | 0.062 |
| NISHIKAGE T | 8 | 0.062 |
| NISHIDA K | 8 | 0.062 |
| NIIHARA M | 8 | 0.062 |
| NIHEI K | 8 | 0.062 |
| NAKAJIMA T | 8 | 0.062 |
| NAKAHARA T | 8 | 0.062 |
| NAGAHARA H | 8 | 0.062 |
| MYOJIN M | 8 | 0.062 |
| MYLES B | 8 | 0.062 |
| MURAYAMA S | 8 | 0.062 |
| MULCAHY MF | 8 | 0.062 |
| MUKHERJEE S | 8 | 0.062 |
| MUKAI M | 8 | 0.062 |
| MORI S | 8 | 0.062 |
| MORI R | 8 | 0.062 |
| MORI N | 8 | 0.062 |
| MONTGOMERY EA | 8 | 0.062 |
| MOCHIKI E | 8 | 0.062 |
| MIYATA Y | 8 | 0.062 |
| MIYAKE K | 8 | 0.062 |
| MIYAGAWA S | 8 | 0.062 |
| MINAMI K | 8 | 0.062 |
| MILEWSKI R | 8 | 0.062 |
| MERKOW RP | 8 | 0.062 |
| MENG X | 8 | 0.062 |
| MENDILAHARSU M | 8 | 0.062 |
| MATSUMOTO T | 8 | 0.062 |
| MATSUBARA T | 8 | 0.062 |
| MARTIN JT | 8 | 0.062 |
| MARJANOVIC G | 8 | 0.062 |
| MAO T | 8 | 0.062 |
| MA YF | 8 | 0.062 |
| MA YC | 8 | 0.062 |
| MA H | 8 | 0.062 |
| LUNG ML | 8 | 0.062 |
| LU XJ | 8 | 0.062 |
| LU Q | 8 | 0.062 |
| LU C | 8 | 0.062 |
| LOPEZ R | 8 | 0.062 |
| LONE MM | 8 | 0.062 |
| LIU ZC | 8 | 0.062 |
| LIU SZ | 8 | 0.062 |
| LIU QW | 8 | 0.062 |
| LIU QS | 8 | 0.062 |
| LIU JJ | 8 | 0.062 |
| LIU HP | 8 | 0.062 |
| LIU CJ | 8 | 0.062 |
| LINDNER K | 8 | 0.062 |
| LINDBLAD M | 8 | 0.062 |
| LIN Y | 8 | 0.062 |
| LIN K | 8 | 0.062 |
| LI YJ | 8 | 0.062 |
| LI TT | 8 | 0.062 |
| LEONG T | 8 | 0.062 |
| LEMMENS VEPP | 8 | 0.062 |
| LEFEVRE JH | 8 | 0.062 |
| LEE DH | 8 | 0.062 |
| LE REST CC | 8 | 0.062 |
| LAMBERT R | 8 | 0.062 |
| KUP PG | 8 | 0.062 |
| KUMAGAI K | 8 | 0.062 |
| KUBOTA K | 8 | 0.062 |
| KRISHNAN S | 8 | 0.062 |
| KONG L | 8 | 0.062 |
| KONDOH C | 8 | 0.062 |
| KOBAYASHI K | 8 | 0.062 |
| KIMURA K | 8 | 0.062 |
| KIMURA J | 8 | 0.062 |
| KIM KM | 8 | 0.062 |
| KIM HI | 8 | 0.062 |
| KIM DJ | 8 | 0.062 |
| KIM CB | 8 | 0.062 |
| KIKUCHI D | 8 | 0.062 |
| KHUSHALANI NI | 8 | 0.062 |
| KHAN M | 8 | 0.062 |
| KAZUNO A | 8 | 0.062 |
| KAWAHARA K | 8 | 0.062 |
| KATOH M | 8 | 0.062 |
| KANETA T | 8 | 0.062 |
| KANDA T | 8 | 0.062 |
| JOHAR A | 8 | 0.062 |
| JIANG YZ | 8 | 0.062 |
| JAGER E | 8 | 0.062 |
| IWATA N | 8 | 0.062 |
| ITOH K | 8 | 0.062 |
| ISOHATA N | 8 | 0.062 |
| ISHWARAN H | 8 | 0.062 |
| IMAI K | 8 | 0.062 |
| IKEDA T | 8 | 0.062 |
| IETA K | 8 | 0.062 |
| HYODO I | 8 | 0.062 |
| HURT C | 8 | 0.062 |
| HUANG SH | 8 | 0.062 |
| HUANG C | 8 | 0.062 |
| HOTEYA S | 8 | 0.062 |
| HOSONO S | 8 | 0.062 |
| HORIBA N | 8 | 0.062 |
| HOFHEINZ R | 8 | 0.062 |
| HO L | 8 | 0.062 |
| HIRASHIMA K | 8 | 0.062 |
| HIRANO S | 8 | 0.062 |
| HIRAHARA N | 8 | 0.062 |
| HINAI Y | 8 | 0.062 |
| HEMMINKI K | 8 | 0.062 |
| HEIST RS | 8 | 0.062 |
| HE ZY | 8 | 0.062 |
| HAVERKAMP L | 8 | 0.062 |
| HAUSTERMANS K | 8 | 0.062 |
| HASEGAWA H | 8 | 0.062 |
| HASE K | 8 | 0.062 |
| HARADA H | 8 | 0.062 |
| HAMAMOTO Y | 8 | 0.062 |
| HAIER J | 8 | 0.062 |
| GUTSCHOW C | 8 | 0.062 |
| GUO X | 8 | 0.062 |
| GUO J | 8 | 0.062 |
| GUAN BX | 8 | 0.062 |
| GRIMMINGER P | 8 | 0.062 |
| GOTOH M | 8 | 0.062 |
| GONG EJ | 8 | 0.062 |
| GILLIES RS | 8 | 0.062 |
| GIACOPUZZI S | 8 | 0.062 |
| GIACOMELLI L | 8 | 0.062 |
| GERDES H | 8 | 0.062 |
| GARAVELLO W | 8 | 0.062 |
| GAO F | 8 | 0.062 |
| FUKAZAWA T | 8 | 0.062 |
| FUJII M | 8 | 0.062 |
| FORD JM | 8 | 0.062 |
| FLEISCHER DE | 8 | 0.062 |
| FISHER GA | 8 | 0.062 |
| FENG AN | 8 | 0.062 |
| FARELLA A | 8 | 0.062 |
| ENZINGER PC | 8 | 0.062 |
| ELICEIRI BP | 8 | 0.062 |
| EL-RIFAI W | 8 | 0.062 |
| DURA P | 8 | 0.062 |
| DOHRMANN T | 8 | 0.062 |
| DINJENS WNM | 8 | 0.062 |
| DIEHL JA | 8 | 0.062 |
| DENEO-PELLEGRINI H | 8 | 0.062 |
| DE STEFANI E | 8 | 0.062 |
| DE HAES HCJM | 8 | 0.062 |
| DAVIES AR | 8 | 0.062 |
| DAI LP | 8 | 0.062 |
| CUI Y | 8 | 0.062 |
| COMBS SE | 8 | 0.062 |
| CLEMONS NJ | 8 | 0.062 |
| CHUNG CS | 8 | 0.062 |
| CHO SH | 8 | 0.062 |
| CHO S | 8 | 0.062 |
| CHIU PWY | 8 | 0.062 |
| CHIU HF | 8 | 0.062 |
| CHENG YL | 8 | 0.062 |
| CHEN ZL | 8 | 0.062 |
| CHEN YS | 8 | 0.062 |
| CHEN YP | 8 | 0.062 |
| CHEN YK | 8 | 0.062 |
| CHEN XL | 8 | 0.062 |
| CHEN XC | 8 | 0.062 |
| CHEN SY | 8 | 0.062 |
| CHEN R | 8 | 0.062 |
| CHEN PT | 8 | 0.062 |
| CHEN MF | 8 | 0.062 |
| CHEN JX | 8 | 0.062 |
| CHATTOPADHYAY I | 8 | 0.062 |
| CAO J | 8 | 0.062 |
| BUTTAR NS | 8 | 0.062 |
| BURGERHOF JGM | 8 | 0.062 |
| BROWN LM | 8 | 0.062 |
| BONAVINA L | 8 | 0.062 |
| BOLDRIN E | 8 | 0.062 |
| BHANDARI P | 8 | 0.062 |
| BERGMAN JJ | 8 | 0.062 |
| BERGMAN J | 8 | 0.062 |
| BENSON AB | 8 | 0.062 |
| BENDELL JC | 8 | 0.062 |
| BEER DG | 8 | 0.062 |
| BARONE C | 8 | 0.062 |
| BADGWELL B | 8 | 0.062 |
| ATTWOOD K | 8 | 0.062 |
| AOKI T | 8 | 0.062 |
| ANANDASABAPATHY S | 8 | 0.062 |
| AMIANO P | 8 | 0.062 |
| ALBERTS SR | 8 | 0.062 |
| AIKOU S | 8 | 0.062 |
| AGARWAL R | 8 | 0.062 |
| ACOSTA G | 8 | 0.062 |
| ZOU XP | 7 | 0.054 |
| ZHOU LQ | 7 | 0.054 |
| ZHOU L | 7 | 0.054 |
| ZHOU FY | 7 | 0.054 |
| ZHOU CC | 7 | 0.054 |
| ZHENG X | 7 | 0.054 |
| ZHENG SY | 7 | 0.054 |
| ZHAO QC | 7 | 0.054 |
| ZHAO JJ | 7 | 0.054 |
| ZHAO HY | 7 | 0.054 |
| ZHAO G | 7 | 0.054 |
| ZHAO DL | 7 | 0.054 |
| ZHANG YY | 7 | 0.054 |
| ZHANG TT | 7 | 0.054 |
| ZHANG SM | 7 | 0.054 |
| ZHANG MY | 7 | 0.054 |
| ZHANG MX | 7 | 0.054 |
| ZHANG JQ | 7 | 0.054 |
| ZHANG JB | 7 | 0.054 |
| ZHANG GY | 7 | 0.054 |
| ZENG Y | 7 | 0.054 |
| ZENG J | 7 | 0.054 |
| ZANONI A | 7 | 0.054 |
| YUE J | 7 | 0.054 |
| YUAN JM | 7 | 0.054 |
| YU XY | 7 | 0.054 |
| YU CH | 7 | 0.054 |
| YOSHITAKE T | 7 | 0.054 |
| YOOK JH | 7 | 0.054 |
| YING JM | 7 | 0.054 |
| YASUI W | 7 | 0.054 |
| YASUDA Y | 7 | 0.054 |
| YASUDA H | 7 | 0.054 |
| YANG YL | 7 | 0.054 |
| YANG W | 7 | 0.054 |
| YANG HY | 7 | 0.054 |
| YANG HS | 7 | 0.054 |
| YAN M | 7 | 0.054 |
| YAMANA I | 7 | 0.054 |
| YAMANA H | 7 | 0.054 |
| YAMAGUCHI Y | 7 | 0.054 |
| YAMAGATA Y | 7 | 0.054 |
| YAMADA A | 7 | 0.054 |
| XUE Q | 7 | 0.054 |
| XU X | 7 | 0.054 |
| XU RH | 7 | 0.054 |
| XU N | 7 | 0.054 |
| XIE Y | 7 | 0.054 |
| XIAO L | 7 | 0.054 |
| WU ZY | 7 | 0.054 |
| WU YY | 7 | 0.054 |
| WU WKK | 7 | 0.054 |
| WU SG | 7 | 0.054 |
| WU Q | 7 | 0.054 |
| WONG KH | 7 | 0.054 |
| WOLFSEN HC | 7 | 0.054 |
| WOBBES T | 7 | 0.054 |
| WITTEMAN BJM | 7 | 0.054 |
| WITTEKIND C | 7 | 0.054 |
| WIKMAN A | 7 | 0.054 |
| WEI J | 7 | 0.054 |
| WATSON A | 7 | 0.054 |
| WATANABE K | 7 | 0.054 |
| WAREHAM N | 7 | 0.054 |
| WANG ZH | 7 | 0.054 |
| WANG XJ | 7 | 0.054 |
| WANG SX | 7 | 0.054 |
| WANG QM | 7 | 0.054 |
| WANG QF | 7 | 0.054 |
| WANG MY | 7 | 0.054 |
| WANG HH | 7 | 0.054 |
| WANG CC | 7 | 0.054 |
| WAKAI T | 7 | 0.054 |
| WADA T | 7 | 0.054 |
| VARGO JJ | 7 | 0.054 |
| VAN HOOFT JE | 7 | 0.054 |
| VAN DER JAGT EJ | 7 | 0.054 |
| VAN DEN BRANDT PA | 7 | 0.054 |
| VACCARO GM | 7 | 0.054 |
| UYAMA I | 7 | 0.054 |
| USUI A | 7 | 0.054 |
| UENO S | 7 | 0.054 |
| UEMURA M | 7 | 0.054 |
| TWINE CP | 7 | 0.054 |
| TUCKER SL | 7 | 0.054 |
| TSURUMARU M | 7 | 0.054 |
| TSUKADA T | 7 | 0.054 |
| TSUCHIYA E | 7 | 0.054 |
| TSUCHIDA A | 7 | 0.054 |
| TSAI YC | 7 | 0.054 |
| TROIANI T | 7 | 0.054 |
| TRICHOPOULOS D | 7 | 0.054 |
| TOMONO Y | 7 | 0.054 |
| TOMINAGA K | 7 | 0.054 |
| TOKUNAGA M | 7 | 0.054 |
| THUSS-PATIENCE PC | 7 | 0.054 |
| THALL PF | 7 | 0.054 |
| TERASHIMA M | 7 | 0.054 |
| TAO L | 7 | 0.054 |
| TAO GZ | 7 | 0.054 |
| TANIGAWA N | 7 | 0.054 |
| TANGOKU A | 7 | 0.054 |
| TANG LL | 7 | 0.054 |
| TANAKA C | 7 | 0.054 |
| TAN YF | 7 | 0.054 |
| TAMURA T | 7 | 0.054 |
| TAMAKI Y | 7 | 0.054 |
| TAKESHITA H | 7 | 0.054 |
| TAKESHIMA F | 7 | 0.054 |
| TAKAMI H | 7 | 0.054 |
| TAKAHASHI A | 7 | 0.054 |
| TAJIMA Y | 7 | 0.054 |
| SUNG SW | 7 | 0.054 |
| SUNG FC | 7 | 0.054 |
| SUN SP | 7 | 0.054 |
| SUN QQ | 7 | 0.054 |
| SUN JY | 7 | 0.054 |
| SU H | 7 | 0.054 |
| STUKENBORG GJ | 7 | 0.054 |
| STARLING N | 7 | 0.054 |
| SOUZA RF | 7 | 0.054 |
| SONG YQ | 7 | 0.054 |
| SONG YP | 7 | 0.054 |
| SONG SY | 7 | 0.054 |
| SONG QX | 7 | 0.054 |
| SMITH DD | 7 | 0.054 |
| SMITH D | 7 | 0.054 |
| SLOOF GW | 7 | 0.054 |
| SINICROPE FA | 7 | 0.054 |
| SIDDIQUI AA | 7 | 0.054 |
| SHOUP M | 7 | 0.054 |
| SHIRAISHI T | 7 | 0.054 |
| SHIMIZU K | 7 | 0.054 |
| SHIMIZU H | 7 | 0.054 |
| SHEN KR | 7 | 0.054 |
| SEUFFERLEIN T | 7 | 0.054 |
| SEPESI B | 7 | 0.054 |
| SENTANI K | 7 | 0.054 |
| SEMRAU R | 7 | 0.054 |
| SCOGGINS CR | 7 | 0.054 |
| SCHULTHEISS TE | 7 | 0.054 |
| SCHULLER JC | 7 | 0.054 |
| SCHMIDT SC | 7 | 0.054 |
| SCHIPPER MEI | 7 | 0.054 |
| SATO A | 7 | 0.054 |
| SASAMOTO R | 7 | 0.054 |
| SALOMON J | 7 | 0.054 |
| SALO J | 7 | 0.054 |
| SAKURAMOTO S | 7 | 0.054 |
| SAIGUSA S | 7 | 0.054 |
| SAADEH LM | 7 | 0.054 |
| RYU JS | 7 | 0.054 |
| ROUGIER P | 7 | 0.054 |
| ROTH MJ | 7 | 0.054 |
| RHEEDER JP | 7 | 0.054 |
| RENOUF DJ | 7 | 0.054 |
| REITSMA JB | 7 | 0.054 |
| REIM D | 7 | 0.054 |
| QUEVEDO JF | 7 | 0.054 |
| QUAN LP | 7 | 0.054 |
| QIN XG | 7 | 0.054 |
| QI H | 7 | 0.054 |
| PURKAYASTHA J | 7 | 0.054 |
| PRUIM J | 7 | 0.054 |
| PREUSSER M | 7 | 0.054 |
| PRENZEL KL | 7 | 0.054 |
| PRAGER G | 7 | 0.054 |
| PRADIER O | 7 | 0.054 |
| PONCHON T | 7 | 0.054 |
| PEZET D | 7 | 0.054 |
| PERA M | 7 | 0.054 |
| PENG L | 7 | 0.054 |
| PAWLITA M | 7 | 0.054 |
| PASINI F | 7 | 0.054 |
| PARSONS SL | 7 | 0.054 |
| PARK SH | 7 | 0.054 |
| PARK JK | 7 | 0.054 |
| PARK J | 7 | 0.054 |
| PARK IK | 7 | 0.054 |
| PAN J | 7 | 0.054 |
| PAN EC | 7 | 0.054 |
| PACELLI R | 7 | 0.054 |
| OSHIMA K | 7 | 0.054 |
| ONOZAWA Y | 7 | 0.054 |
| ONAITIS MW | 7 | 0.054 |
| OMLOO JMT | 7 | 0.054 |
| OLINER KS | 7 | 0.054 |
| OHTA K | 7 | 0.054 |
| OHKI T | 7 | 0.054 |
| O'FARRELL NJ | 7 | 0.054 |
| NWOGU CE | 7 | 0.054 |
| NONAKA T | 7 | 0.054 |
| NOH SH | 7 | 0.054 |
| NIYAZ M | 7 | 0.054 |
| NISHIMORI T | 7 | 0.054 |
| NEUHAUS P | 7 | 0.054 |
| NAVA H | 7 | 0.054 |
| NAPOLEON B | 7 | 0.054 |
| NAMIKAWA T | 7 | 0.054 |
| NAKAYAMA H | 7 | 0.054 |
| NAKAYAMA E | 7 | 0.054 |
| NAKATSU T | 7 | 0.054 |
| NAGINO M | 7 | 0.054 |
| MUROI H | 7 | 0.054 |
| MURAYAMA M | 7 | 0.054 |
| MUNGO B | 7 | 0.054 |
| MORGAN MA | 7 | 0.054 |
| MONTGOMERY E | 7 | 0.054 |
| MOHLER M | 7 | 0.054 |
| MIZUKAMI T | 7 | 0.054 |
| MIYAZAWA Y | 7 | 0.054 |
| MIYAZAKI S | 7 | 0.054 |
| MIYAWAKI Y | 7 | 0.054 |
| MIURA A | 7 | 0.054 |
| MISHRA G | 7 | 0.054 |
| MINAMI H | 7 | 0.054 |
| MIGITA K | 7 | 0.054 |
| MERAT S | 7 | 0.054 |
| MELIS M | 7 | 0.054 |
| MCALEER MF | 7 | 0.054 |
| MAYNARD ND | 7 | 0.054 |
| MATSUMOTO H | 7 | 0.054 |
| MATSUDA A | 7 | 0.054 |
| MASUDA T | 7 | 0.054 |
| MASON RC | 7 | 0.054 |
| MASAKI T | 7 | 0.054 |
| MAO YS | 7 | 0.054 |
| MANSFIELD P | 7 | 0.054 |
| MANDA R | 7 | 0.054 |
| MALIK V | 7 | 0.054 |
| MAHMOODI M | 7 | 0.054 |
| MACHIDA N | 7 | 0.054 |
| MA X | 7 | 0.054 |
| MA Q | 7 | 0.054 |
| MA JX | 7 | 0.054 |
| MA G | 7 | 0.054 |
| LUYER MDP | 7 | 0.054 |
| LUO JD | 7 | 0.054 |
| LUO H | 7 | 0.054 |
| LU M | 7 | 0.054 |
| LU L | 7 | 0.054 |
| LOU PJ | 7 | 0.054 |
| LORENZ D | 7 | 0.054 |
| LOH E | 7 | 0.054 |
| LIU YZ | 7 | 0.054 |
| LIU XX | 7 | 0.054 |
| LIU XH | 7 | 0.054 |
| LIU SW | 7 | 0.054 |
| LIU N | 7 | 0.054 |
| LIU FJ | 7 | 0.054 |
| LIU DL | 7 | 0.054 |
| LINGHU EQ | 7 | 0.054 |
| LIN YC | 7 | 0.054 |
| LIN S | 7 | 0.054 |
| LIN CY | 7 | 0.054 |
| LIETO E | 7 | 0.054 |
| LIANG Y | 7 | 0.054 |
| LIANG JA | 7 | 0.054 |
| LI YL | 7 | 0.054 |
| LI XX | 7 | 0.054 |
| LI QQ | 7 | 0.054 |
| LI MH | 7 | 0.054 |
| LI JW | 7 | 0.054 |
| LI JS | 7 | 0.054 |
| LI JC | 7 | 0.054 |
| LI JB | 7 | 0.054 |
| LI HP | 7 | 0.054 |
| LI HF | 7 | 0.054 |
| LI CY | 7 | 0.054 |
| LEVINE EA | 7 | 0.054 |
| LEE YY | 7 | 0.054 |
| LEE TH | 7 | 0.054 |
| LEE IS | 7 | 0.054 |
| LEE HW | 7 | 0.054 |
| KUMANO I | 7 | 0.054 |
| KUDO S | 7 | 0.054 |
| KOYAMA Y | 7 | 0.054 |
| KONDA VJA | 7 | 0.054 |
| KODASHIMA S | 7 | 0.054 |
| KOCHER M | 7 | 0.054 |
| KOBAYASHI Y | 7 | 0.054 |
| KOBAYASHI D | 7 | 0.054 |
| KITAMOTO Y | 7 | 0.054 |
| KINOSHITA K | 7 | 0.054 |
| KIM YJ | 7 | 0.054 |
| KIM SM | 7 | 0.054 |
| KIM SK | 7 | 0.054 |
| KIM MY | 7 | 0.054 |
| KIM MK | 7 | 0.054 |
| KIM JW | 7 | 0.054 |
| KHOSHNIA M | 7 | 0.054 |
| KESLER KA | 7 | 0.054 |
| KAWANO H | 7 | 0.054 |
| KAWAMURA T | 7 | 0.054 |
| KAWAMURA M | 7 | 0.054 |
| KAWAGUCHI T | 7 | 0.054 |
| KAWACHI H | 7 | 0.054 |
| KATZKA DA | 7 | 0.054 |
| KATAI H | 7 | 0.054 |
| KARTHIKESALINGAM A | 7 | 0.054 |
| KANEYASU Y | 7 | 0.054 |
| KAN T | 7 | 0.054 |
| KACHNIC LA | 7 | 0.054 |
| JUNG HC | 7 | 0.054 |
| JONES CE | 7 | 0.054 |
| JIN H | 7 | 0.054 |
| JIANG T | 7 | 0.054 |
| JIANG JF | 7 | 0.054 |
| JIANG F | 7 | 0.054 |
| JIANG D | 7 | 0.054 |
| JIA YB | 7 | 0.054 |
| JIA WH | 7 | 0.054 |
| JI CY | 7 | 0.054 |
| IZUMI Y | 7 | 0.054 |
| ITO K | 7 | 0.054 |
| ISOZAKI Y | 7 | 0.054 |
| ISHIYAMA A | 7 | 0.054 |
| IMATANI A | 7 | 0.054 |
| IITAKA D | 7 | 0.054 |
| IGARASHI M | 7 | 0.054 |
| HUR C | 7 | 0.054 |
| HULSHOF M | 7 | 0.054 |
| HUANG QY | 7 | 0.054 |
| HUANG M | 7 | 0.054 |
| HUANG JZ | 7 | 0.054 |
| HUANG HL | 7 | 0.054 |
| HUANG HH | 7 | 0.054 |
| HSIEH CY | 7 | 0.054 |
| HOTTA K | 7 | 0.054 |
| HOSHINO S | 7 | 0.054 |
| HORIMATSU T | 7 | 0.054 |
| HONING J | 7 | 0.054 |
| HOFFMANN AC | 7 | 0.054 |
| HOFFMAN RM | 7 | 0.054 |
| HIRATA H | 7 | 0.054 |
| HIRASAWA T | 7 | 0.054 |
| HIRAI T | 7 | 0.054 |
| HINO H | 7 | 0.054 |
| HICKS RJ | 7 | 0.054 |
| HERLYN M | 7 | 0.054 |
| HENEGOUWEN MV | 7 | 0.054 |
| HATT M | 7 | 0.054 |
| HATAZAWA J | 7 | 0.054 |
| HASINA R | 7 | 0.054 |
| HARDWICK RH | 7 | 0.054 |
| HAO HF | 7 | 0.054 |
| HANNA N | 7 | 0.054 |
| HANAZAKI K | 7 | 0.054 |
| HANAFUSA M | 7 | 0.054 |
| HAAG GM | 7 | 0.054 |
| GUO LP | 7 | 0.054 |
| GUHA S | 7 | 0.054 |
| GRABSCH HI | 7 | 0.054 |
| GOTOH K | 7 | 0.054 |
| GOTODA T | 7 | 0.054 |
| GOTLEY DC | 7 | 0.054 |
| GOSSAGE JA | 7 | 0.054 |
| GINSBERG GG | 7 | 0.054 |
| GELDERBLOM WCA | 7 | 0.054 |
| GAO YX | 7 | 0.054 |
| GAO CM | 7 | 0.054 |
| FUJISAKI J | 7 | 0.054 |
| FUJINO Y | 7 | 0.054 |
| FOSTER NR | 7 | 0.054 |
| FENG XL | 7 | 0.054 |
| FENG W | 7 | 0.054 |
| FANG XH | 7 | 0.054 |
| FANG J | 7 | 0.054 |
| EZOE Y | 7 | 0.054 |
| EVANGELISTA L | 7 | 0.054 |
| ERASMUS JJ | 7 | 0.054 |
| ENOMOTO K | 7 | 0.054 |
| ENDO S | 7 | 0.054 |
| ELOUBEIDI MA | 7 | 0.054 |
| EGUCHI H | 7 | 0.054 |
| EBERT M | 7 | 0.054 |
| DUTTON MF | 7 | 0.054 |
| DUNST CM | 7 | 0.054 |
| DU YW | 7 | 0.054 |
| DU JJ | 7 | 0.054 |
| DJARV T | 7 | 0.054 |
| DING Y | 7 | 0.054 |
| DING T | 7 | 0.054 |
| DI MARTINO N | 7 | 0.054 |
| DI FABIO F | 7 | 0.054 |
| DAVID S | 7 | 0.054 |
| DAR NA | 7 | 0.054 |
| CZITO BG | 7 | 0.054 |
| CROSBY TDL | 7 | 0.054 |
| CORTI L | 7 | 0.054 |
| CONIO M | 7 | 0.054 |
| CLAVEL-CHAPELON F | 7 | 0.054 |
| CHUNG HC | 7 | 0.054 |
| CHUNG H | 7 | 0.054 |
| CHOI YH | 7 | 0.054 |
| CHOI MG | 7 | 0.054 |
| CHOI JY | 7 | 0.054 |
| CHO CH | 7 | 0.054 |
| CHINO O | 7 | 0.054 |
| CHEN YT | 7 | 0.054 |
| CHEN YL | 7 | 0.054 |
| CHEN XN | 7 | 0.054 |
| CHEN XM | 7 | 0.054 |
| CHEN XH | 7 | 0.054 |
| CHEN XF | 7 | 0.054 |
| CHEN QR | 7 | 0.054 |
| CHEN LL | 7 | 0.054 |
| CHEN JZ | 7 | 0.054 |
| CHEN DF | 7 | 0.054 |
| CHANG YL | 7 | 0.054 |
| CHANG WK | 7 | 0.054 |
| CHANG KJ | 7 | 0.054 |
| CHANG H | 7 | 0.054 |
| CHANG CC | 7 | 0.054 |
| CATENACCI DVT | 7 | 0.054 |
| CAO L | 7 | 0.054 |
| CAI Y | 7 | 0.054 |
| CAI L | 7 | 0.054 |
| BURIAN M | 7 | 0.054 |
| BURGER HM | 7 | 0.054 |
| BRENNER B | 7 | 0.054 |
| BOUTRON-RUAULT MC | 7 | 0.054 |
| BOKEMEYER C | 7 | 0.054 |
| BLUM M | 7 | 0.054 |
| BLACKMON SH | 7 | 0.054 |
| BIEBL M | 7 | 0.054 |
| BELLA AE | 7 | 0.054 |
| BELKA C | 7 | 0.054 |
| BEL A | 7 | 0.054 |
| BASU D | 7 | 0.054 |
| BAO XH | 7 | 0.054 |
| BAEKSGAARD L | 7 | 0.054 |
| ATMACA A | 7 | 0.054 |
| ATHERTON PJ | 7 | 0.054 |
| ARAKAWA T | 7 | 0.054 |
| APPERSON-HANSEN C | 7 | 0.054 |
| APISARNTHANARAX S | 7 | 0.054 |
| ANDRICI J | 7 | 0.054 |
| ANDREOLLO NA | 7 | 0.054 |
| ANDL CD | 7 | 0.054 |
| AMINI A | 7 | 0.054 |
| AMANO R | 7 | 0.054 |
| AKLILU M | 7 | 0.054 |
| AKAZAWA Y | 7 | 0.054 |
| AKANUMA N | 7 | 0.054 |
| ADAMIETZ IA | 7 | 0.054 |
| ABE Y | 7 | 0.054 |
| ABBRUZZESE JL | 7 | 0.054 |
| ZUM BUSCHENFELDE CM | 6 | 0.046 |
| ZUCCARO G | 6 | 0.046 |
| ZINSMEISTER AR | 6 | 0.046 |
| ZINGG U | 6 | 0.046 |
| ZHU YH | 6 | 0.046 |
| ZHU W | 6 | 0.046 |
| ZHU JF | 6 | 0.046 |
| ZHU B | 6 | 0.046 |
| ZHOU XY | 6 | 0.046 |
| ZHOU SL | 6 | 0.046 |
| ZHOU JH | 6 | 0.046 |
| ZHOU H | 6 | 0.046 |
| ZHOU F | 6 | 0.046 |
| ZHONG J | 6 | 0.046 |
| ZHAO K | 6 | 0.046 |
| ZHAO D | 6 | 0.046 |
| ZHANG YM | 6 | 0.046 |
| ZHANG WJ | 6 | 0.046 |
| ZHANG SK | 6 | 0.046 |
| ZHANG QY | 6 | 0.046 |
| ZHANG LF | 6 | 0.046 |
| ZHANG JL | 6 | 0.046 |
| ZHANG JD | 6 | 0.046 |
| ZENG MS | 6 | 0.046 |
| ZENDEHDEL K | 6 | 0.046 |
| ZANCHETTIN G | 6 | 0.046 |
| YUAN ZY | 6 | 0.046 |
| YU JP | 6 | 0.046 |
| YU HP | 6 | 0.046 |
| YU CG | 6 | 0.046 |
| YIN Y | 6 | 0.046 |
| YI JL | 6 | 0.046 |
| YENDAMURI S | 6 | 0.046 |
| YEN CJ | 6 | 0.046 |
| YATABE Y | 6 | 0.046 |
| YAO LQ | 6 | 0.046 |
| YAO L | 6 | 0.046 |
| YANG YJ | 6 | 0.046 |
| YANG XY | 6 | 0.046 |
| YANG WC | 6 | 0.046 |
| YANG TL | 6 | 0.046 |
| YANG SJ | 6 | 0.046 |
| YANG SB | 6 | 0.046 |
| YANG HH | 6 | 0.046 |
| YAN H | 6 | 0.046 |
| YAMASHITA T | 6 | 0.046 |
| YAMASHINA T | 6 | 0.046 |
| YAMADA M | 6 | 0.046 |
| YAKOUB D | 6 | 0.046 |
| XU GL | 6 | 0.046 |
| XIE CY | 6 | 0.046 |
| XIAO Z | 6 | 0.046 |
| XIA Y | 6 | 0.046 |
| XI M | 6 | 0.046 |
| WU XY | 6 | 0.046 |
| WU W | 6 | 0.046 |
| WU TX | 6 | 0.046 |
| WU TN | 6 | 0.046 |
| WU SY | 6 | 0.046 |
| WU N | 6 | 0.046 |
| WORRELL SG | 6 | 0.046 |
| WONG JYC | 6 | 0.046 |
| WISNIVESKY JP | 6 | 0.046 |
| WIGLE DA | 6 | 0.046 |
| WHELAN KA | 6 | 0.046 |
| WESTON BR | 6 | 0.046 |
| WEKSLER B | 6 | 0.046 |
| WARREN GW | 6 | 0.046 |
| WARD MH | 6 | 0.046 |
| WANG ZX | 6 | 0.046 |
| WANG ZT | 6 | 0.046 |
| WANG ZL | 6 | 0.046 |
| WANG XZ | 6 | 0.046 |
| WANG XX | 6 | 0.046 |
| WANG TT | 6 | 0.046 |
| WANG NN | 6 | 0.046 |
| WANG LP | 6 | 0.046 |
| WANG LF | 6 | 0.046 |
| WANG JC | 6 | 0.046 |
| WANG GH | 6 | 0.046 |
| WANG FH | 6 | 0.046 |
| WANG CH | 6 | 0.046 |
| WALLACE MB | 6 | 0.046 |
| WALCH A | 6 | 0.046 |
| WAKITA A | 6 | 0.046 |
| VON RAHDEN BHA | 6 | 0.046 |
| VON MOOS R | 6 | 0.046 |
| VIDETIC GM | 6 | 0.046 |
| VAN VLIET EPM | 6 | 0.046 |
| VAN MARION R | 6 | 0.046 |
| VAN LANSCHOT JJ | 6 | 0.046 |
| URBA S | 6 | 0.046 |
| UEMURA N | 6 | 0.046 |
| UEDA Y | 6 | 0.046 |
| TURATI F | 6 | 0.046 |
| TSUSHIMA Y | 6 | 0.046 |
| TSUJIKAWA T | 6 | 0.046 |
| TSUGANE S | 6 | 0.046 |
| TSUCHIDA T | 6 | 0.046 |
| TRAN TCK | 6 | 0.046 |
| TOMODA K | 6 | 0.046 |
| TOMINAGA M | 6 | 0.046 |
| TOKUNAGA E | 6 | 0.046 |
| TJULANDIN S | 6 | 0.046 |
| TIAN JM | 6 | 0.046 |
| TAVANI A | 6 | 0.046 |
| TAO Q | 6 | 0.046 |
| TANAKA O | 6 | 0.046 |
| TAN S | 6 | 0.046 |
| TAN ACITL | 6 | 0.046 |
| TALLEY NJ | 6 | 0.046 |
| TAKIUCHI H | 6 | 0.046 |
| TAKESHITA N | 6 | 0.046 |
| TAKENO A | 6 | 0.046 |
| TAKANO A | 6 | 0.046 |
| TAJIRI H | 6 | 0.046 |
| TACCIOLI C | 6 | 0.046 |
| SUZUKI G | 6 | 0.046 |
| SUNDQUIST J | 6 | 0.046 |
| SUNDARAM A | 6 | 0.046 |
| SUN T | 6 | 0.046 |
| SUN M | 6 | 0.046 |
| SUN LX | 6 | 0.046 |
| SUN HL | 6 | 0.046 |
| SUN HF | 6 | 0.046 |
| SUN GP | 6 | 0.046 |
| SUGIMOTO T | 6 | 0.046 |
| SUGIHARA H | 6 | 0.046 |
| SUDA K | 6 | 0.046 |
| STRUECKER B | 6 | 0.046 |
| STOLTE M | 6 | 0.046 |
| STERZING F | 6 | 0.046 |
| STEM M | 6 | 0.046 |
| STAVELEY-O'CARROLL KF | 6 | 0.046 |
| STAFFURTH J | 6 | 0.046 |
| STAAL EFWC | 6 | 0.046 |
| SRIVASTAVA A | 6 | 0.046 |
| SPICER J | 6 | 0.046 |
| SPEICHER PJ | 6 | 0.046 |
| SONOYAMA T | 6 | 0.046 |
| SONG T | 6 | 0.046 |
| SONG JY | 6 | 0.046 |
| SONG CH | 6 | 0.046 |
| SMIT J | 6 | 0.046 |
| SMETS EMA | 6 | 0.046 |
| SMETS EM | 6 | 0.046 |
| SMALLEY KJ | 6 | 0.046 |
| SINGHAL S | 6 | 0.046 |
| SINGH V | 6 | 0.046 |
| SINGH R | 6 | 0.046 |
| SHU XO | 6 | 0.046 |
| SHIRAISHI Y | 6 | 0.046 |
| SHIRAISHI K | 6 | 0.046 |
| SHIOZAKI M | 6 | 0.046 |
| SHINOMURA Y | 6 | 0.046 |
| SHIMIZU T | 6 | 0.046 |
| SHIM HJ | 6 | 0.046 |
| SHICHINOHE T | 6 | 0.046 |
| SHIBUYA K | 6 | 0.046 |
| SHI M | 6 | 0.046 |
| SHEN YY | 6 | 0.046 |
| SHEN SP | 6 | 0.046 |
| SHEN Q | 6 | 0.046 |
| SHEN J | 6 | 0.046 |
| SHARMA VK | 6 | 0.046 |
| SHARMA S | 6 | 0.046 |
| SHARMA J | 6 | 0.046 |
| SHAN HB | 6 | 0.046 |
| SHAH IA | 6 | 0.046 |
| SENDLER A | 6 | 0.046 |
| SCHWARZ RE | 6 | 0.046 |
| SCHUCHERT MJ | 6 | 0.046 |
| SCHRUMP DS | 6 | 0.046 |
| SCHOMAS DA | 6 | 0.046 |
| SCHMIDBERGER H | 6 | 0.046 |
| SCHMALENBERG H | 6 | 0.046 |
| SCHEEPERS JJG | 6 | 0.046 |
| SAWAYAMA H | 6 | 0.046 |
| SAWADA T | 6 | 0.046 |
| SAWADA S | 6 | 0.046 |
| SATO K | 6 | 0.046 |
| SATO E | 6 | 0.046 |
| SASAKI A | 6 | 0.046 |
| SARKARIA IS | 6 | 0.046 |
| SANG MX | 6 | 0.046 |
| SALO JA | 6 | 0.046 |
| SAKAKURA C | 6 | 0.046 |
| SAHA S | 6 | 0.046 |
| RUTEGARD M | 6 | 0.046 |
| ROSS HJ | 6 | 0.046 |
| ROSENBERG R | 6 | 0.046 |
| RONELLENFITSCH U | 6 | 0.046 |
| ROEDL JB | 6 | 0.046 |
| ROBERTS A | 6 | 0.046 |
| RIZK N | 6 | 0.046 |
| RINGASH J | 6 | 0.046 |
| RIETHDORF S | 6 | 0.046 |
| RICHEL DJ | 6 | 0.046 |
| REYNOLDS J | 6 | 0.046 |
| REN JS | 6 | 0.046 |
| REN J | 6 | 0.046 |
| REHM J | 6 | 0.046 |
| RAZ DJ | 6 | 0.046 |
| RAGUNATH K | 6 | 0.046 |
| RABENSTEIN T | 6 | 0.046 |
| QUAN PL | 6 | 0.046 |
| QIN XB | 6 | 0.046 |
| QIN SK | 6 | 0.046 |
| QIAO XY | 6 | 0.046 |
| PURI V | 6 | 0.046 |
| PRASAD GA | 6 | 0.046 |
| POULTSIDES GA | 6 | 0.046 |
| POMERRI F | 6 | 0.046 |
| PODVIN S | 6 | 0.046 |
| PLUSCHNIG U | 6 | 0.046 |
| PLUKKER J | 6 | 0.046 |
| PINTO E | 6 | 0.046 |
| PHUKAN R | 6 | 0.046 |
| PENG YF | 6 | 0.046 |
| PENG H | 6 | 0.046 |
| PEDRAZZANI C | 6 | 0.046 |
| PAZDRO A | 6 | 0.046 |
| PATEL K | 6 | 0.046 |
| PASQUER A | 6 | 0.046 |
| PARK SR | 6 | 0.046 |
| PARENTE P | 6 | 0.046 |
| PANICO S | 6 | 0.046 |
| PALLI D | 6 | 0.046 |
| OYA H | 6 | 0.046 |
| OTSU H | 6 | 0.046 |
| OTA Y | 6 | 0.046 |
| OSHIMA Y | 6 | 0.046 |
| OLARU A | 6 | 0.046 |
| OKITA N | 6 | 0.046 |
| OKINES AFC | 6 | 0.046 |
| OKABE T | 6 | 0.046 |
| OHTA S | 6 | 0.046 |
| OHNO T | 6 | 0.046 |
| OHARA S | 6 | 0.046 |
| OH DY | 6 | 0.046 |
| OGAWA Y | 6 | 0.046 |
| OGAWA JI | 6 | 0.046 |
| OFNER D | 6 | 0.046 |
| OBERTOP H | 6 | 0.046 |
| O'TOOLE D | 6 | 0.046 |
| NURKIN SJ | 6 | 0.046 |
| NONOSHITA T | 6 | 0.046 |
| NOBLE F | 6 | 0.046 |
| NISHIYAMA M | 6 | 0.046 |
| NISHINA T | 6 | 0.046 |
| NISHIDA M | 6 | 0.046 |
| NIEDER C | 6 | 0.046 |
| NG T | 6 | 0.046 |
| NEORAL C | 6 | 0.046 |
| NEGORO Y | 6 | 0.046 |
| NATSUIZAKA M | 6 | 0.046 |
| NAM TK | 6 | 0.046 |
| NAKASHIMA S | 6 | 0.046 |
| NAKAJIMA TE | 6 | 0.046 |
| NAKAGAWA T | 6 | 0.046 |
| NAGATA H | 6 | 0.046 |
| MUTRI V | 6 | 0.046 |
| MURAKAMI H | 6 | 0.046 |
| MULLER RP | 6 | 0.046 |
| MULLER K | 6 | 0.046 |
| MUL VEM | 6 | 0.046 |
| MUELLER PR | 6 | 0.046 |
| MOUGHAN J | 6 | 0.046 |
| MORITA Y | 6 | 0.046 |
| MORII E | 6 | 0.046 |
| MOMMA K | 6 | 0.046 |
| MOHAGHEGHI MA | 6 | 0.046 |
| MIZOTA A | 6 | 0.046 |
| MIYAHARA Y | 6 | 0.046 |
| MIN JX | 6 | 0.046 |
| MILANO F | 6 | 0.046 |
| MIAO XP | 6 | 0.046 |
| MEYERHARDT JA | 6 | 0.046 |
| MEURER L | 6 | 0.046 |
| MEROPOL NJ | 6 | 0.046 |
| MENG L | 6 | 0.046 |
| MCNAMARA MJ | 6 | 0.046 |
| MCMILLAN DC | 6 | 0.046 |
| MCMASTERS KM | 6 | 0.046 |
| MAUGHAN T | 6 | 0.046 |
| MATSUURA N | 6 | 0.046 |
| MATSUOKA J | 6 | 0.046 |
| MATSUI F | 6 | 0.046 |
| MATSUBAYASHI H | 6 | 0.046 |
| MATHISEN DJ | 6 | 0.046 |
| MASUDA M | 6 | 0.046 |
| MASON R | 6 | 0.046 |
| MANSFIELD PF | 6 | 0.046 |
| MALTHANER R | 6 | 0.046 |
| MAKI K | 6 | 0.046 |
| MAITHEL SK | 6 | 0.046 |
| MAIER A | 6 | 0.046 |
| MAHER SG | 6 | 0.046 |
| MACEFIELD RC | 6 | 0.046 |
| MA K | 6 | 0.046 |
| MA JJ | 6 | 0.046 |
| MA JB | 6 | 0.046 |
| MA J | 6 | 0.046 |
| MA C | 6 | 0.046 |
| LYROS O | 6 | 0.046 |
| LUTZKE LS | 6 | 0.046 |
| LURJE G | 6 | 0.046 |
| LUO Y | 6 | 0.046 |
| LUO XY | 6 | 0.046 |
| LUO RZ | 6 | 0.046 |
| LUNA RA | 6 | 0.046 |
| LUKASZEWICZ-ZAJAC M | 6 | 0.046 |
| LUJAN-BARROSO L | 6 | 0.046 |
| LUC G | 6 | 0.046 |
| LU ZS | 6 | 0.046 |
| LU YY | 6 | 0.046 |
| LU XX | 6 | 0.046 |
| LU MS | 6 | 0.046 |
| LU H | 6 | 0.046 |
| LU CH | 6 | 0.046 |
| LIVINGSTONE AS | 6 | 0.046 |
| LIU ZM | 6 | 0.046 |
| LIU YM | 6 | 0.046 |
| LIU YJ | 6 | 0.046 |
| LIU XL | 6 | 0.046 |
| LIU SY | 6 | 0.046 |
| LIU SL | 6 | 0.046 |
| LIU JG | 6 | 0.046 |
| LIU A | 6 | 0.046 |
| LIPPERT H | 6 | 0.046 |
| LINDENMANN J | 6 | 0.046 |
| LIN YL | 6 | 0.046 |
| LIN PY | 6 | 0.046 |
| LIN M | 6 | 0.046 |
| LIN JC | 6 | 0.046 |
| LIN HX | 6 | 0.046 |
| LIN DX | 6 | 0.046 |
| LIM HJ | 6 | 0.046 |
| LIM CH | 6 | 0.046 |
| LIDOR AO | 6 | 0.046 |
| LIAO ZXX | 6 | 0.046 |
| LIANG S | 6 | 0.046 |
| LIANG N | 6 | 0.046 |
| LI ZY | 6 | 0.046 |
| LI ZS | 6 | 0.046 |
| LI YQ | 6 | 0.046 |
| LI YP | 6 | 0.046 |
| LI WJ | 6 | 0.046 |
| LI WH | 6 | 0.046 |
| LI SB | 6 | 0.046 |
| LI QL | 6 | 0.046 |
| LI LL | 6 | 0.046 |
| LI JY | 6 | 0.046 |
| LI JX | 6 | 0.046 |
| LI JH | 6 | 0.046 |
| LI HX | 6 | 0.046 |
| LI DJ | 6 | 0.046 |
| LEVINE MS | 6 | 0.046 |
| LEE MY | 6 | 0.046 |
| LEE MP | 6 | 0.046 |
| LECLEIRE S | 6 | 0.046 |
| LAUDANSKI W | 6 | 0.046 |
| LASKAR SG | 6 | 0.046 |
| LAL P | 6 | 0.046 |
| KUSUMOTO T | 6 | 0.046 |
| KURODA J | 6 | 0.046 |
| KULKE MH | 6 | 0.046 |
| KUBOTA H | 6 | 0.046 |
| KRISTINSSON JO | 6 | 0.046 |
| KOYOTA S | 6 | 0.046 |
| KOSHY M | 6 | 0.046 |
| KOIKE K | 6 | 0.046 |
| KOIDE N | 6 | 0.046 |
| KOEFFLER HP | 6 | 0.046 |
| KOBAYASHI T | 6 | 0.046 |
| KIM N | 6 | 0.046 |
| KIM M | 6 | 0.046 |
| KIM G | 6 | 0.046 |
| KIJIMA Y | 6 | 0.046 |
| KHURANA R | 6 | 0.046 |
| KHAN A | 6 | 0.046 |
| KERNSTINE KH | 6 | 0.046 |
| KELLY RJ | 6 | 0.046 |
| KELLER G | 6 | 0.046 |
| KELLEHER D | 6 | 0.046 |
| KAWASE T | 6 | 0.046 |
| KAWANO S | 6 | 0.046 |
| KAWAI M | 6 | 0.046 |
| KAWAGUCHI G | 6 | 0.046 |
| KAWADA I | 6 | 0.046 |
| KAWABATA R | 6 | 0.046 |
| KATO S | 6 | 0.046 |
| KATAOKA K | 6 | 0.046 |
| KANGAWA K | 6 | 0.046 |
| KANAMORI S | 6 | 0.046 |
| KAMPHUIS M | 6 | 0.046 |
| KALININA T | 6 | 0.046 |
| KAIDA S | 6 | 0.046 |
| KAHALEH M | 6 | 0.046 |
| KAAKS R | 6 | 0.046 |
| JU HX | 6 | 0.046 |
| JOHNSTON C | 6 | 0.046 |
| JOHNSTON BT | 6 | 0.046 |
| JOHNSON RL | 6 | 0.046 |
| JIN W | 6 | 0.046 |
| JIN TT | 6 | 0.046 |
| JIANG L | 6 | 0.046 |
| JIANG J | 6 | 0.046 |
| JIA Y | 6 | 0.046 |
| JESCH B | 6 | 0.046 |
| JENSEN LS | 6 | 0.046 |
| JAFARI E | 6 | 0.046 |
| IZUMI D | 6 | 0.046 |
| IZBICKI J | 6 | 0.046 |
| IZAWA S | 6 | 0.046 |
| IVESON T | 6 | 0.046 |
| ITOH Y | 6 | 0.046 |
| ITO E | 6 | 0.046 |
| ITAMI J | 6 | 0.046 |
| ISHIOKA C | 6 | 0.046 |
| ISHIKAWA Y | 6 | 0.046 |
| ISHIKAWA K | 6 | 0.046 |
| ISHIHARA M | 6 | 0.046 |
| ISHIDA T | 6 | 0.046 |
| INABA K | 6 | 0.046 |
| IMANISHI T | 6 | 0.046 |
| IMAI N | 6 | 0.046 |
| IKEMATSU H | 6 | 0.046 |
| IGISSINOV S | 6 | 0.046 |
| IGISSINOV N | 6 | 0.046 |
| HWANG JH | 6 | 0.046 |
| HWANG JE | 6 | 0.046 |
| HULSHOF MC | 6 | 0.046 |
| HUANG Z | 6 | 0.046 |
| HUANG YC | 6 | 0.046 |
| HUANG P | 6 | 0.046 |
| HUANG CT | 6 | 0.046 |
| HUANG CG | 6 | 0.046 |
| HU Q | 6 | 0.046 |
| HSIEH TY | 6 | 0.046 |
| HOSPERS GA | 6 | 0.046 |
| HOSOKAWA A | 6 | 0.046 |
| HOSHINO M | 6 | 0.046 |
| HORWHAT JD | 6 | 0.046 |
| HORIBA MN | 6 | 0.046 |
| HONJO S | 6 | 0.046 |
| HOLLEMA H | 6 | 0.046 |
| HOKAMURA N | 6 | 0.046 |
| HO KY | 6 | 0.046 |
| HINOJOSA MW | 6 | 0.046 |
| HILL J | 6 | 0.046 |
| HIGASHI M | 6 | 0.046 |
| HENNE-BRUNS D | 6 | 0.046 |
| HENDRICKS DT | 6 | 0.046 |
| HE Y | 6 | 0.046 |
| HE S | 6 | 0.046 |
| HAYASHI A | 6 | 0.046 |
| HATTORI T | 6 | 0.046 |
| HATA K | 6 | 0.046 |
| HARTMANN JT | 6 | 0.046 |
| HART J | 6 | 0.046 |
| HARADA S | 6 | 0.046 |
| HAMILTON JP | 6 | 0.046 |
| HADDOCK MG | 6 | 0.046 |
| GWYNNE S | 6 | 0.046 |
| GUERRERO T | 6 | 0.046 |
| GRIFFITHS G | 6 | 0.046 |
| GREEN S | 6 | 0.046 |
| GOSSNER L | 6 | 0.046 |
| GONEN M | 6 | 0.046 |
| GOLD JS | 6 | 0.046 |
| GOING JJ | 6 | 0.046 |
| GOH V | 6 | 0.046 |
| GOENSE L | 6 | 0.046 |
| GODFREY TE | 6 | 0.046 |
| GLEESON FV | 6 | 0.046 |
| GIAQUINTA S | 6 | 0.046 |
| GENO DM | 6 | 0.046 |
| GEISSLER M | 6 | 0.046 |
| GEBAUER F | 6 | 0.046 |
| GAUR P | 6 | 0.046 |
| GATENBY PAC | 6 | 0.046 |
| GARMAN KS | 6 | 0.046 |
| GAO XS | 6 | 0.046 |
| FUSE N | 6 | 0.046 |
| FUKUTOMI A | 6 | 0.046 |
| FUKUSHIMA J | 6 | 0.046 |
| FUKUDA T | 6 | 0.046 |
| FU J | 6 | 0.046 |
| FRIESLAND S | 6 | 0.046 |
| FRAUMENI JF | 6 | 0.046 |
| FORSHAW MJ | 6 | 0.046 |
| FORMAN D | 6 | 0.046 |
| FONTAINE JP | 6 | 0.046 |
| FONG LYY | 6 | 0.046 |
| FONG LY | 6 | 0.046 |
| FINK U | 6 | 0.046 |
| FINDLAY JM | 6 | 0.046 |
| FIETKAU R | 6 | 0.046 |
| FERRARI P | 6 | 0.046 |
| FENG SY | 6 | 0.046 |
| FENG L | 6 | 0.046 |
| FARRAN L | 6 | 0.046 |
| FANG P | 6 | 0.046 |
| FAN TY | 6 | 0.046 |
| FAIZ Z | 6 | 0.046 |
| ERASMUS J | 6 | 0.046 |
| ENGLUM BR | 6 | 0.046 |
| ENDO K | 6 | 0.046 |
| EFFENBERGER K | 6 | 0.046 |
| DUNCAN M | 6 | 0.046 |
| DRAHOS J | 6 | 0.046 |
| DOYAMA H | 6 | 0.046 |
| DONG WG | 6 | 0.046 |
| DONG W | 6 | 0.046 |
| DOBRITZ M | 6 | 0.046 |
| DEVITT PG | 6 | 0.046 |
| DEROGAR M | 6 | 0.046 |
| DENG XF | 6 | 0.046 |
| DEMARTINES N | 6 | 0.046 |
| DECKER G | 6 | 0.046 |
| DE JONGE J | 6 | 0.046 |
| DAS M | 6 | 0.046 |
| DAS KM | 6 | 0.046 |
| DAI JG | 6 | 0.046 |
| CUI J | 6 | 0.046 |
| CROSBY TD | 6 | 0.046 |
| CREEMERS GJ | 6 | 0.046 |
| CORREA P | 6 | 0.046 |
| COOPER GS | 6 | 0.046 |
| COLEN RR | 6 | 0.046 |
| COLEMAN HG | 6 | 0.046 |
| CHUANG WY | 6 | 0.046 |
| CHO JY | 6 | 0.046 |
| CHENG YJ | 6 | 0.046 |
| CHENG L | 6 | 0.046 |
| CHEN ZM | 6 | 0.046 |
| CHEN ZH | 6 | 0.046 |
| CHEN XY | 6 | 0.046 |
| CHEN PP | 6 | 0.046 |
| CHEN N | 6 | 0.046 |
| CHEN MH | 6 | 0.046 |
| CHEN MC | 6 | 0.046 |
| CHEN JF | 6 | 0.046 |
| CHEN GW | 6 | 0.046 |
| CHATTOPADHYAY TK | 6 | 0.046 |
| CHARALAMPAKIS N | 6 | 0.046 |
| CHAO J | 6 | 0.046 |
| CHANG SC | 6 | 0.046 |
| CHANG S | 6 | 0.046 |
| CHANG D | 6 | 0.046 |
| CHANG CH | 6 | 0.046 |
| CHANDRASOMA P | 6 | 0.046 |
| CHAN SC | 6 | 0.046 |
| CECCONELLO I | 6 | 0.046 |
| CATALANO G | 6 | 0.046 |
| CASCINU S | 6 | 0.046 |
| CARRATO A | 6 | 0.046 |
| CAO Y | 6 | 0.046 |
| CAO XF | 6 | 0.046 |
| CAO HX | 6 | 0.046 |
| CAO FL | 6 | 0.046 |
| CAO BP | 6 | 0.046 |
| CANTWELL MM | 6 | 0.046 |
| CAI H | 6 | 0.046 |
| BUDACH W | 6 | 0.046 |
| BUCK AK | 6 | 0.046 |
| BRONSON NW | 6 | 0.046 |
| BRIGAND C | 6 | 0.046 |
| BRIEZ N | 6 | 0.046 |
| BRADLEY KM | 6 | 0.046 |
| BOTH S | 6 | 0.046 |
| BOSSET JF | 6 | 0.046 |
| BOSCH DJ | 6 | 0.046 |
| BOOKA E | 6 | 0.046 |
| BONNETAIN F | 6 | 0.046 |
| BONDE P | 6 | 0.046 |
| BOIGE V | 6 | 0.046 |
| BLOM RLGM | 6 | 0.046 |
| BLENCOWE NS | 6 | 0.046 |
| BILIMORIA KY | 6 | 0.046 |
| BIERE SSAY | 6 | 0.046 |
| BHAT GA | 6 | 0.046 |
| BESHARAT S | 6 | 0.046 |
| BASS AJ | 6 | 0.046 |
| BARRICARTE A | 6 | 0.046 |
| BANKI F | 6 | 0.046 |
| BANERJEE S | 6 | 0.046 |
| BANDO E | 6 | 0.046 |
| BAIL JP | 6 | 0.046 |
| BABAEI M | 6 | 0.046 |
| AVERY KNL | 6 | 0.046 |
| ATHANASIOU T | 6 | 0.046 |
| ASAKA M | 6 | 0.046 |
| AOYAMA N | 6 | 0.046 |
| ANDEREGG MCJ | 6 | 0.046 |
| AMINIAN A | 6 | 0.046 |
| ALIBAKHSHI A | 6 | 0.046 |
| ALEMAN BMP | 6 | 0.046 |
| AKIYAMA J | 6 | 0.046 |
| AIKOU T | 6 | 0.046 |
| AGHCHELI K | 6 | 0.046 |
| AEBERSOLD DM | 6 | 0.046 |
| ABRAHAMSEN B | 6 | 0.046 |
| ABRAHAM JM | 6 | 0.046 |
| ABE K | 6 | 0.046 |
| ZOU XN | 5 | 0.039 |
| ZHU XF | 5 | 0.039 |
| ZHU X | 5 | 0.039 |
| ZHU SJ | 5 | 0.039 |
| ZHU Q | 5 | 0.039 |
| ZHU K | 5 | 0.039 |
| ZHOU YB | 5 | 0.039 |
| ZHOU XQ | 5 | 0.039 |
| ZHOU XL | 5 | 0.039 |
| ZHOU QH | 5 | 0.039 |
| ZHOU M | 5 | 0.039 |
| ZHOU LP | 5 | 0.039 |
| ZHOU JY | 5 | 0.039 |
| ZHOU JD | 5 | 0.039 |
| ZHENG ZG | 5 | 0.039 |
| ZHENG YL | 5 | 0.039 |
| ZHENG YD | 5 | 0.039 |
| ZHENG WH | 5 | 0.039 |
| ZHENG J | 5 | 0.039 |
| ZHAO ZF | 5 | 0.039 |
| ZHAO XK | 5 | 0.039 |
| ZHAO RH | 5 | 0.039 |
| ZHAO N | 5 | 0.039 |
| ZHAO JH | 5 | 0.039 |
| ZHANG ZQ | 5 | 0.039 |
| ZHANG ZD | 5 | 0.039 |
| ZHANG ZC | 5 | 0.039 |
| ZHANG YX | 5 | 0.039 |
| ZHANG XW | 5 | 0.039 |
| ZHANG WG | 5 | 0.039 |
| ZHANG RX | 5 | 0.039 |
| ZHANG JJ | 5 | 0.039 |
| ZHANG HZ | 5 | 0.039 |
| ZHANG HY | 5 | 0.039 |
| ZHANG HP | 5 | 0.039 |
| ZHANG CS | 5 | 0.039 |
| ZHANG BJ | 5 | 0.039 |
| ZFASS A | 5 | 0.039 |
| ZARIDZE D | 5 | 0.039 |
| ZAIDI AH | 5 | 0.039 |
| YUKAWA N | 5 | 0.039 |
| YUASA Y | 5 | 0.039 |
| YUAN QP | 5 | 0.039 |
| YUAN H | 5 | 0.039 |
| YU ZT | 5 | 0.039 |
| YU XS | 5 | 0.039 |
| YU XM | 5 | 0.039 |
| YU X | 5 | 0.039 |
| YU K | 5 | 0.039 |
| YU E | 5 | 0.039 |
| YOSHINO S | 5 | 0.039 |
| YOSHIMURA N | 5 | 0.039 |
| YOSHIMI N | 5 | 0.039 |
| YOSHIMATSU K | 5 | 0.039 |
| YOSHIKAWA Y | 5 | 0.039 |
| YOSHIKAWA R | 5 | 0.039 |
| YOSHIDA H | 5 | 0.039 |
| YORKE ED | 5 | 0.039 |
| YOON C | 5 | 0.039 |
| YONG WP | 5 | 0.039 |
| YONEYAMA Y | 5 | 0.039 |
| YIP C | 5 | 0.039 |
| YEKEBAS E | 5 | 0.039 |
| YEH CM | 5 | 0.039 |
| YE YQ | 5 | 0.039 |
| YE P | 5 | 0.039 |
| YAZAWA S | 5 | 0.039 |
| YANG WJ | 5 | 0.039 |
| YANG SY | 5 | 0.039 |
| YANG SF | 5 | 0.039 |
| YANG LY | 5 | 0.039 |
| YANG JJ | 5 | 0.039 |
| YANG JH | 5 | 0.039 |
| YANG HL | 5 | 0.039 |
| YANG DY | 5 | 0.039 |
| YANG CR | 5 | 0.039 |
| YANG C | 5 | 0.039 |
| YANG BX | 5 | 0.039 |
| YANAGITA S | 5 | 0.039 |
| YAN YJ | 5 | 0.039 |
| YAN Y | 5 | 0.039 |
| YAN S | 5 | 0.039 |
| YAMAZAKI S | 5 | 0.039 |
| YAMAZAKI M | 5 | 0.039 |
| YAMASHITA N | 5 | 0.039 |
| YAMAI H | 5 | 0.039 |
| YAMAGUCHI A | 5 | 0.039 |
| YAMAGISHI H | 5 | 0.039 |
| YAMAGAMI H | 5 | 0.039 |
| YAJIMA S | 5 | 0.039 |
| XUE L | 5 | 0.039 |
| XU Z | 5 | 0.039 |
| XU YZ | 5 | 0.039 |
| XU YQ | 5 | 0.039 |
| XU WW | 5 | 0.039 |
| XU Q | 5 | 0.039 |
| XU LP | 5 | 0.039 |
| XU JJ | 5 | 0.039 |
| XU HX | 5 | 0.039 |
| XING LG | 5 | 0.039 |
| XING J | 5 | 0.039 |
| XIE P | 5 | 0.039 |
| XIE H | 5 | 0.039 |
| XIE C | 5 | 0.039 |
| XIAO ZF | 5 | 0.039 |
| XIAO Y | 5 | 0.039 |
| WU Z | 5 | 0.039 |
| WU YL | 5 | 0.039 |
| WU XJ | 5 | 0.039 |
| WU SC | 5 | 0.039 |
| WU QC | 5 | 0.039 |
| WU KS | 5 | 0.039 |
| WU JC | 5 | 0.039 |
| WU F | 5 | 0.039 |
| WOUTERSEN D | 5 | 0.039 |
| WONG SL | 5 | 0.039 |
| WOLL E | 5 | 0.039 |
| WOJCIESZYNSKI AP | 5 | 0.039 |
| WILDING G | 5 | 0.039 |
| WILD CP | 5 | 0.039 |
| WEUSTEN BLAM | 5 | 0.039 |
| WESTER HJ | 5 | 0.039 |
| WENZ F | 5 | 0.039 |
| WEIMANN A | 5 | 0.039 |
| WEIJS TJ | 5 | 0.039 |
| WEI Y | 5 | 0.039 |
| WEI JC | 5 | 0.039 |
| WEI CM | 5 | 0.039 |
| WATERBOER T | 5 | 0.039 |
| WANG ZY | 5 | 0.039 |
| WANG ZW | 5 | 0.039 |
| WANG YX | 5 | 0.039 |
| WANG XB | 5 | 0.039 |
| WANG WY | 5 | 0.039 |
| WANG SF | 5 | 0.039 |
| WANG SC | 5 | 0.039 |
| WANG ML | 5 | 0.039 |
| WANG LW | 5 | 0.039 |
| WANG LL | 5 | 0.039 |
| WANG JM | 5 | 0.039 |
| WANG JJ | 5 | 0.039 |
| WANG HZ | 5 | 0.039 |
| WANG DL | 5 | 0.039 |
| WANG DD | 5 | 0.039 |
| WANG CL | 5 | 0.039 |
| WAN J | 5 | 0.039 |
| WALSH TN | 5 | 0.039 |
| WALLNER B | 5 | 0.039 |
| WAINBERG ZA | 5 | 0.039 |
| VOLANT A | 5 | 0.039 |
| VISTE A | 5 | 0.039 |
| VILLAFLOR V | 5 | 0.039 |
| VESTERMARK LW | 5 | 0.039 |
| VENKAT P | 5 | 0.039 |
| VAZQUEZ-SEQUEIROS E | 5 | 0.039 |
| VAUTHEY JN | 5 | 0.039 |
| VAN VELTHUYSEN MLF | 5 | 0.039 |
| VAN LEEUWEN MS | 5 | 0.039 |
| VAN DAM GM | 5 | 0.039 |
| VAN BOMMEL J | 5 | 0.039 |
| VAN BAAL JWPM | 5 | 0.039 |
| UPTON MP | 5 | 0.039 |
| UNO K | 5 | 0.039 |
| UNDERWOOD TJ | 5 | 0.039 |
| UM SH | 5 | 0.039 |
| UCHIYAMA H | 5 | 0.039 |
| TZENG CH | 5 | 0.039 |
| TSUNODA S | 5 | 0.039 |
| TSUJITANI S | 5 | 0.039 |
| TSUJII Y | 5 | 0.039 |
| TSUJI I | 5 | 0.039 |
| TSUDA T | 5 | 0.039 |
| TSUDA H | 5 | 0.039 |
| TSENG PH | 5 | 0.039 |
| TRARBACH T | 5 | 0.039 |
| TONG Q | 5 | 0.039 |
| TOLLENAAR RAEM | 5 | 0.039 |
| TOKAIRIN Y | 5 | 0.039 |
| TIEU BH | 5 | 0.039 |
| TIAN ZQ | 5 | 0.039 |
| THOTA PN | 5 | 0.039 |
| THEREAUX J | 5 | 0.039 |
| TERRIS B | 5 | 0.039 |
| TERASHIMA H | 5 | 0.039 |
| TERAHARA A | 5 | 0.039 |
| TENG CJ | 5 | 0.039 |
| TEN KATE FJ | 5 | 0.039 |
| TATEMATSU N | 5 | 0.039 |
| TAO H | 5 | 0.039 |
| TANIGAWA T | 5 | 0.039 |
| TANG R | 5 | 0.039 |
| TANABE M | 5 | 0.039 |
| TAMURA S | 5 | 0.039 |
| TAKU K | 5 | 0.039 |
| TAKESUE T | 5 | 0.039 |
| TAKAI Y | 5 | 0.039 |
| TAKAGI Y | 5 | 0.039 |
| TAKAGI M | 5 | 0.039 |
| TAI CM | 5 | 0.039 |
| TACHIBANA M | 5 | 0.039 |
| SZESZENIA-DABROWSKA N | 5 | 0.039 |
| SZELACHOWSKI P | 5 | 0.039 |
| SWANSTROM LL | 5 | 0.039 |
| SUZUKI K | 5 | 0.039 |
| SUR R | 5 | 0.039 |
| SUN ZG | 5 | 0.039 |
| SUN WJ | 5 | 0.039 |
| SUN MH | 5 | 0.039 |
| SUN KL | 5 | 0.039 |
| SUN GJ | 5 | 0.039 |
| SUN G | 5 | 0.039 |
| SUH YS | 5 | 0.039 |
| SU P | 5 | 0.039 |
| STREUTKER CJ | 5 | 0.039 |
| STEINMETZ K | 5 | 0.039 |
| STAIRS DB | 5 | 0.039 |
| SONG QK | 5 | 0.039 |
| SONG M | 5 | 0.039 |
| SONG LMWK | 5 | 0.039 |
| SONG GA | 5 | 0.039 |
| SOHAL D | 5 | 0.039 |
| SOETIKNO R | 5 | 0.039 |
| SITU DR | 5 | 0.039 |
| SIMON R | 5 | 0.039 |
| SIEGEL EM | 5 | 0.039 |
| SHOJI H | 5 | 0.039 |
| SHIOZAWA S | 5 | 0.039 |
| SHINOZAKI E | 5 | 0.039 |
| SHINGAI T | 5 | 0.039 |
| SHIMODAIRA Y | 5 | 0.039 |
| SHIMIZU D | 5 | 0.039 |
| SHIEH TY | 5 | 0.039 |
| SHI ZZ | 5 | 0.039 |
| SHI Y | 5 | 0.039 |
| SHI L | 5 | 0.039 |
| SHI JX | 5 | 0.039 |
| SHENG JY | 5 | 0.039 |
| SHEN LY | 5 | 0.039 |
| SHAH A | 5 | 0.039 |
| SEPANLOU SG | 5 | 0.039 |
| SENGE MO | 5 | 0.039 |
| SELGRAD M | 5 | 0.039 |
| SEKI Y | 5 | 0.039 |
| SEEWALD S | 5 | 0.039 |
| SCOTT AW | 5 | 0.039 |
| SCHURR P | 5 | 0.039 |
| SCHREURS LMA | 5 | 0.039 |
| SCHOUTEN LJ | 5 | 0.039 |
| SCHMIDT H | 5 | 0.039 |
| SCHAFER M | 5 | 0.039 |
| SAUER BG | 5 | 0.039 |
| SATOH Y | 5 | 0.039 |
| SATOH A | 5 | 0.039 |
| SASAKI M | 5 | 0.039 |
| SASAI K | 5 | 0.039 |
| SANCHIS V | 5 | 0.039 |
| SANCHEZ MJ | 5 | 0.039 |
| SAMBYAL V | 5 | 0.039 |
| SALVADOR R | 5 | 0.039 |
| SALASPURO M | 5 | 0.039 |
| SAKOGAWA K | 5 | 0.039 |
| SAKAI S | 5 | 0.039 |
| SAKAI A | 5 | 0.039 |
| SAGAERT X | 5 | 0.039 |
| SAARNIO J | 5 | 0.039 |
| RYGIEL AM | 5 | 0.039 |
| RYAN AM | 5 | 0.039 |
| RUTTEN HJT | 5 | 0.039 |
| RUSCHOFF J | 5 | 0.039 |
| RUBELLO D | 5 | 0.039 |
| ROY R | 5 | 0.039 |
| ROTH A | 5 | 0.039 |
| ROSSIDIS G | 5 | 0.039 |
| ROSMAN C | 5 | 0.039 |
| ROOF KS | 5 | 0.039 |
| ROGERS JE | 5 | 0.039 |
| RODEL C | 5 | 0.039 |
| RIZZETTO C | 5 | 0.039 |
| RINO Y | 5 | 0.039 |
| RHA SY | 5 | 0.039 |
| REN Y | 5 | 0.039 |
| REID TD | 5 | 0.039 |
| REID BJ | 5 | 0.039 |
| REAVIS KM | 5 | 0.039 |
| REALDON S | 5 | 0.039 |
| RAYMOND DP | 5 | 0.039 |
| RAYMOND D | 5 | 0.039 |
| RASOOL S | 5 | 0.039 |
| RASHIDKHANI B | 5 | 0.039 |
| RASANEN JV | 5 | 0.039 |
| RASANEN J | 5 | 0.039 |
| RAO JN | 5 | 0.039 |
| RAN YL | 5 | 0.039 |
| RAMPADO S | 5 | 0.039 |
| RAMOS AJ | 5 | 0.039 |
| RAJA S | 5 | 0.039 |
| QUERO L | 5 | 0.039 |
| QU Y | 5 | 0.039 |
| QU CX | 5 | 0.039 |
| QIU X | 5 | 0.039 |
| QIU H | 5 | 0.039 |
| QIU B | 5 | 0.039 |
| QIN JJ | 5 | 0.039 |
| QI Y | 5 | 0.039 |
| PROBST S | 5 | 0.039 |
| POTTGEN C | 5 | 0.039 |
| PORSCHEN R | 5 | 0.039 |
| POLYDORIDES AD | 5 | 0.039 |
| POLOMSKY M | 5 | 0.039 |
| POLOM K | 5 | 0.039 |
| POLESEL J | 5 | 0.039 |
| PICKENS A | 5 | 0.039 |
| PHUKAN RK | 5 | 0.039 |
| PHATAK P | 5 | 0.039 |
| PHAN A | 5 | 0.039 |
| PETRIOLI R | 5 | 0.039 |
| PESKO P | 5 | 0.039 |
| PEI HL | 5 | 0.039 |
| PATTYN P | 5 | 0.039 |
| PATTERSON GA | 5 | 0.039 |
| PATEL D | 5 | 0.039 |
| PARRY K | 5 | 0.039 |
| PARK M | 5 | 0.039 |
| PARK K | 5 | 0.039 |
| PARK JY | 5 | 0.039 |
| PARK JM | 5 | 0.039 |
| PARK JJ | 5 | 0.039 |
| PANG XL | 5 | 0.039 |
| PAN YQ | 5 | 0.039 |
| PAN X | 5 | 0.039 |
| PALAZZO F | 5 | 0.039 |
| PAARDEKOOPER G | 5 | 0.039 |
| OUE N | 5 | 0.039 |
| OTSUKA Y | 5 | 0.039 |
| ORIUCHI N | 5 | 0.039 |
| ORIGER J | 5 | 0.039 |
| OPITZ OG | 5 | 0.039 |
| OONO Y | 5 | 0.039 |
| ONODA T | 5 | 0.039 |
| ONO K | 5 | 0.039 |
| OMATA M | 5 | 0.039 |
| OKUNO T | 5 | 0.039 |
| OKAMURA S | 5 | 0.039 |
| OKAMURA A | 5 | 0.039 |
| OKAMOTO M | 5 | 0.039 |
| OIDA T | 5 | 0.039 |
| OHTANI H | 5 | 0.039 |
| OHATA K | 5 | 0.039 |
| OGATA H | 5 | 0.039 |
| OFFERHAUS GJA | 5 | 0.039 |
| OELSCHLAGER BK | 5 | 0.039 |
| OECHSNER M | 5 | 0.039 |
| ODENTHAL M | 5 | 0.039 |
| NOURAIE M | 5 | 0.039 |
| NORAT T | 5 | 0.039 |
| NISHIYAMA N | 5 | 0.039 |
| NISHITANI S | 5 | 0.039 |
| NISHIMURA M | 5 | 0.039 |
| NISHIKAWA H | 5 | 0.039 |
| NISHIKAWA A | 5 | 0.039 |
| NISHIBUCHI I | 5 | 0.039 |
| NING Y | 5 | 0.039 |
| NIEUWENHUIJZEN GA | 5 | 0.039 |
| NG EKW | 5 | 0.039 |
| NEUMANN H | 5 | 0.039 |
| NEUHAUS H | 5 | 0.039 |
| NEUGUT AI | 5 | 0.039 |
| NESLAND JM | 5 | 0.039 |
| NENTWICH MF | 5 | 0.039 |
| NEISS S | 5 | 0.039 |
| NAUMNIK W | 5 | 0.039 |
| NASSERI-MOGHADDAM S | 5 | 0.039 |
| NASERI AR | 5 | 0.039 |
| NAPOLITANO V | 5 | 0.039 |
| NAKAYAMA T | 5 | 0.039 |
| NAKASHIMA Y | 5 | 0.039 |
| NAKAO M | 5 | 0.039 |
| NAKANUMA S | 5 | 0.039 |
| NAKAJO A | 5 | 0.039 |
| NAKA T | 5 | 0.039 |
| NAITOH H | 5 | 0.039 |
| NAITO Y | 5 | 0.039 |
| NAGDA S | 5 | 0.039 |
| NAGAWA H | 5 | 0.039 |
| NAGASAKA T | 5 | 0.039 |
| NAGANO H | 5 | 0.039 |
| NAGAMI Y | 5 | 0.039 |
| MURPHY G | 5 | 0.039 |
| MURATA A | 5 | 0.039 |
| MURAKAMI T | 5 | 0.039 |
| MURAKAMI M | 5 | 0.039 |
| MURAI T | 5 | 0.039 |
| MU JW | 5 | 0.039 |
| MOUSAVI SM | 5 | 0.039 |
| MOTOKI T | 5 | 0.039 |
| MOTOHASHI O | 5 | 0.039 |
| MORTON LM | 5 | 0.039 |
| MORSE CR | 5 | 0.039 |
| MORIMOTO M | 5 | 0.039 |
| MONTELLA M | 5 | 0.039 |
| MONIG S | 5 | 0.039 |
| MOHEBBI M | 5 | 0.039 |
| MOHAMMADZADEH M | 5 | 0.039 |
| MOHAMMAD K | 5 | 0.039 |
| MIZUKAMI Y | 5 | 0.039 |
| MIYAMOTO M | 5 | 0.039 |
| MIYAHARA R | 5 | 0.039 |
| MIURA Y | 5 | 0.039 |
| MITSUHASHI N | 5 | 0.039 |
| MITANI T | 5 | 0.039 |
| MISRA S | 5 | 0.039 |
| MISHRA S | 5 | 0.039 |
| MIRZA F | 5 | 0.039 |
| MIRSHARIFI R | 5 | 0.039 |
| MIRINEZHAD SK | 5 | 0.039 |
| MIR MR | 5 | 0.039 |
| MIN BH | 5 | 0.039 |
| MIMATSU K | 5 | 0.039 |
| MIKI H | 5 | 0.039 |
| MIKHAIL S | 5 | 0.039 |
| MICHAYLIRA CZ | 5 | 0.039 |
| MICHAEL M | 5 | 0.039 |
| MIAO LS | 5 | 0.039 |
| MEYER J | 5 | 0.039 |
| MEYER F | 5 | 0.039 |
| MENG XL | 5 | 0.039 |
| MELICHAR B | 5 | 0.039 |
| MCLOUGHLIN JM | 5 | 0.039 |
| MCGRATH K | 5 | 0.039 |
| MATZI V | 5 | 0.039 |
| MATTIELLO A | 5 | 0.039 |
| MATSUSHIMA K | 5 | 0.039 |
| MATSUKI A | 5 | 0.039 |
| MATSUI A | 5 | 0.039 |
| MAROM EM | 5 | 0.039 |
| MARKAR S | 5 | 0.039 |
| MARIN S | 5 | 0.039 |
| MARASAS WFO | 5 | 0.039 |
| MARANO L | 5 | 0.039 |
| MALUF F | 5 | 0.039 |
| MAKISHIMA H | 5 | 0.039 |
| MAFUNE K | 5 | 0.039 |
| MAEDA Y | 5 | 0.039 |
| MADDAUS MA | 5 | 0.039 |
| MAAS KW | 5 | 0.039 |
| MA ZQ | 5 | 0.039 |
| MA YL | 5 | 0.039 |
| MA XL | 5 | 0.039 |
| MA S | 5 | 0.039 |
| MA LL | 5 | 0.039 |
| MA LF | 5 | 0.039 |
| LV L | 5 | 0.039 |
| LV J | 5 | 0.039 |
| LV GD | 5 | 0.039 |
| LUO YJ | 5 | 0.039 |
| LUO RC | 5 | 0.039 |
| LUO GY | 5 | 0.039 |
| LUND E | 5 | 0.039 |
| LULEY K | 5 | 0.039 |
| LU S | 5 | 0.039 |
| LU QY | 5 | 0.039 |
| LU P | 5 | 0.039 |
| LU JC | 5 | 0.039 |
| LOW D | 5 | 0.039 |
| LOVAT LB | 5 | 0.039 |
| LOUIE BE | 5 | 0.039 |
| LIU ZK | 5 | 0.039 |
| LIU YX | 5 | 0.039 |
| LIU YQ | 5 | 0.039 |
| LIU YL | 5 | 0.039 |
| LIU QX | 5 | 0.039 |
| LIU QH | 5 | 0.039 |
| LIU KD | 5 | 0.039 |
| LIU JP | 5 | 0.039 |
| LIU HY | 5 | 0.039 |
| LIU AM | 5 | 0.039 |
| LISOVSKY M | 5 | 0.039 |
| LING FC | 5 | 0.039 |
| LINDKVIST B | 5 | 0.039 |
| LIN SW | 5 | 0.039 |
| LIN G | 5 | 0.039 |
| LIN D | 5 | 0.039 |
| LIN CS | 5 | 0.039 |
| LIM H | 5 | 0.039 |
| LIANG D | 5 | 0.039 |
| LI ZP | 5 | 0.039 |
| LI ZH | 5 | 0.039 |
| LI YX | 5 | 0.039 |
| LI SM | 5 | 0.039 |
| LI QH | 5 | 0.039 |
| LI LY | 5 | 0.039 |
| LI LW | 5 | 0.039 |
| LI LF | 5 | 0.039 |
| LI HS | 5 | 0.039 |
| LI DW | 5 | 0.039 |
| LI DR | 5 | 0.039 |
| LENGLINGER J | 5 | 0.039 |
| LEITZMANN MF | 5 | 0.039 |
| LEERS JM | 5 | 0.039 |
| LEERS J | 5 | 0.039 |
| LEE YS | 5 | 0.039 |
| LEE W | 5 | 0.039 |
| LEE T | 5 | 0.039 |
| LEE SY | 5 | 0.039 |
| LEE SJ | 5 | 0.039 |
| LEE NP | 5 | 0.039 |
| LEE MS | 5 | 0.039 |
| LEE HH | 5 | 0.039 |
| LEE G | 5 | 0.039 |
| LEE CY | 5 | 0.039 |
| LEE C | 5 | 0.039 |
| LANUTI M | 5 | 0.039 |
| KWONG DLW | 5 | 0.039 |
| KWON J | 5 | 0.039 |
| KUSANO M | 5 | 0.039 |
| KURODA D | 5 | 0.039 |
| KURIBAYASHI S | 5 | 0.039 |
| KUNZ PL | 5 | 0.039 |
| KUNDEL Y | 5 | 0.039 |
| KUCHARCZUK JC | 5 | 0.039 |
| KUBOI Y | 5 | 0.039 |
| KROGH M | 5 | 0.039 |
| KRIEG A | 5 | 0.039 |
| KRASINSKAS AM | 5 | 0.039 |
| KOSINSKI AS | 5 | 0.039 |
| KONNO S | 5 | 0.039 |
| KOK FJ | 5 | 0.039 |
| KOJIMA Y | 5 | 0.039 |
| KOENIG AM | 5 | 0.039 |
| KO CY | 5 | 0.039 |
| KNOX J | 5 | 0.039 |
| KLIGERMAN S | 5 | 0.039 |
| KLEINBERG L | 5 | 0.039 |
| KITAYAMA J | 5 | 0.039 |
| KITANO S | 5 | 0.039 |
| KING S | 5 | 0.039 |
| KIMCHI ET | 5 | 0.039 |
| KIM Y | 5 | 0.039 |
| KIM WH | 5 | 0.039 |
| KIM T | 5 | 0.039 |
| KIM SY | 5 | 0.039 |
| KIM SW | 5 | 0.039 |
| KIM MP | 5 | 0.039 |
| KIM E | 5 | 0.039 |
| KIM DW | 5 | 0.039 |
| KIM DK | 5 | 0.039 |
| KIM D | 5 | 0.039 |
| KILIC E | 5 | 0.039 |
| KIKUCHI M | 5 | 0.039 |
| KIKUCHI H | 5 | 0.039 |
| KIJIMA H | 5 | 0.039 |
| KIESSLICH R | 5 | 0.039 |
| KELLEY ST | 5 | 0.039 |
| KAWASAKI A | 5 | 0.039 |
| KAWAI T | 5 | 0.039 |
| KAWADA N | 5 | 0.039 |
| KAUR T | 5 | 0.039 |
| KATOH H | 5 | 0.039 |
| KASAGI Y | 5 | 0.039 |
| KANTETI R | 5 | 0.039 |
| KANO H | 5 | 0.039 |
| KANG X | 5 | 0.039 |
| KANG MC | 5 | 0.039 |
| KANDIOLER D | 5 | 0.039 |
| KANAJI S | 5 | 0.039 |
| KAMPMAN E | 5 | 0.039 |
| KAMEDA Y | 5 | 0.039 |
| KABAT B | 5 | 0.039 |
| JOHANSSON M | 5 | 0.039 |
| JOHANSSON J | 5 | 0.039 |
| JIWNANI S | 5 | 0.039 |
| JIN Y | 5 | 0.039 |
| JIN XC | 5 | 0.039 |
| JIN S | 5 | 0.039 |
| JIN M | 5 | 0.039 |
| JIANG YN | 5 | 0.039 |
| JIANG YB | 5 | 0.039 |
| JIANG XY | 5 | 0.039 |
| JIA XT | 5 | 0.039 |
| JIA XD | 5 | 0.039 |
| JIA L | 5 | 0.039 |
| JI Y | 5 | 0.039 |
| JI JF | 5 | 0.039 |
| JI BT | 5 | 0.039 |
| JEON HM | 5 | 0.039 |
| JAUCH KW | 5 | 0.039 |
| JAROSZEWSKI DE | 5 | 0.039 |
| JANG G | 5 | 0.039 |
| JABBOUR SK | 5 | 0.039 |
| IWAYA T | 5 | 0.039 |
| ITOH T | 5 | 0.039 |
| ITASAKA S | 5 | 0.039 |
| ISOBE M | 5 | 0.039 |
| ISHIYAMA H | 5 | 0.039 |
| ISHIKAWA N | 5 | 0.039 |
| ISHIDA M | 5 | 0.039 |
| INOUE S | 5 | 0.039 |
| INOUE N | 5 | 0.039 |
| INOUE K | 5 | 0.039 |
| IMAOKA S | 5 | 0.039 |
| IMAMOTO H | 5 | 0.039 |
| IDELEVICH E | 5 | 0.039 |
| IDE H | 5 | 0.039 |
| IBIEBELE TI | 5 | 0.039 |
| HWANG TZ | 5 | 0.039 |
| HWANG JC | 5 | 0.039 |
| HUSSAIN S | 5 | 0.039 |
| HURWITZ HI | 5 | 0.039 |
| HUR H | 5 | 0.039 |
| HULSHOFF JB | 5 | 0.039 |
| HUI ZG | 5 | 0.039 |
| HUERTA JM | 5 | 0.039 |
| HUANG YW | 5 | 0.039 |
| HUANG XE | 5 | 0.039 |
| HUANG JF | 5 | 0.039 |
| HUANG HT | 5 | 0.039 |
| HUANG G | 5 | 0.039 |
| HU YW | 5 | 0.039 |
| HU LK | 5 | 0.039 |
| HSU HS | 5 | 0.039 |
| HSU HH | 5 | 0.039 |
| HSU CP | 5 | 0.039 |
| HSU C | 5 | 0.039 |
| HSIEH TC | 5 | 0.039 |
| HSIEH CH | 5 | 0.039 |
| HOU J | 5 | 0.039 |
| HOSHINO A | 5 | 0.039 |
| HORGAN AM | 5 | 0.039 |
| HOQUE A | 5 | 0.039 |
| HOP WCJ | 5 | 0.039 |
| HOLLYWOOD D | 5 | 0.039 |
| HOLLAND JM | 5 | 0.039 |
| HOLCATOVA I | 5 | 0.039 |
| HOCHWALD S | 5 | 0.039 |
| HIURA Y | 5 | 0.039 |
| HIROHASHI Y | 5 | 0.039 |
| HIRAO M | 5 | 0.039 |
| HIRANO K | 5 | 0.039 |
| HIRAIWA K | 5 | 0.039 |
| HIGASHIDA M | 5 | 0.039 |
| HIBI T | 5 | 0.039 |
| HERZOG SL | 5 | 0.039 |
| HERESBACH D | 5 | 0.039 |
| HERBOLD T | 5 | 0.039 |
| HENEGOUWEN MIVB | 5 | 0.039 |
| HEINEMANN V | 5 | 0.039 |
| HEILAND M | 5 | 0.039 |
| HEGER U | 5 | 0.039 |
| HE LR | 5 | 0.039 |
| HE G | 5 | 0.039 |
| HE CY | 5 | 0.039 |
| HAYWARD NK | 5 | 0.039 |
| HAYABUCHI N | 5 | 0.039 |
| HAWKINS MA | 5 | 0.039 |
| HATAKEYAMA K | 5 | 0.039 |
| HASHIMOTO Y | 5 | 0.039 |
| HASHIMOTO S | 5 | 0.039 |
| HASAN Q | 5 | 0.039 |
| HARUMA K | 5 | 0.039 |
| HARTWIG MG | 5 | 0.039 |
| HARRIS CC | 5 | 0.039 |
| HAROLD KL | 5 | 0.039 |
| HARMSEN WS | 5 | 0.039 |
| HARIRCHI I | 5 | 0.039 |
| HARINGSMA J | 5 | 0.039 |
| HARATA K | 5 | 0.039 |
| HARA J | 5 | 0.039 |
| HAO JJ | 5 | 0.039 |
| HAO CQ | 5 | 0.039 |
| HAN S | 5 | 0.039 |
| HALLEMEIER CL | 5 | 0.039 |
| HAJIZADEH B | 5 | 0.039 |
| HAINAUT P | 5 | 0.039 |
| GUPTA T | 5 | 0.039 |
| GUPTA A | 5 | 0.039 |
| GUO LW | 5 | 0.039 |
| GUO CH | 5 | 0.039 |
| GUNTER E | 5 | 0.039 |
| GUNDOGDU B | 5 | 0.039 |
| GUERRERO TM | 5 | 0.039 |
| GUAN SH | 5 | 0.039 |
| GU ZT | 5 | 0.039 |
| GU Y | 5 | 0.039 |
| GU DY | 5 | 0.039 |
| GU CP | 5 | 0.039 |
| GROBLEWSKA M | 5 | 0.039 |
| GRIN A | 5 | 0.039 |
| GRAHAM DY | 5 | 0.039 |
| GRADY WM | 5 | 0.039 |
| GOVINDAN R | 5 | 0.039 |
| GOTO M | 5 | 0.039 |
| GORE EM | 5 | 0.039 |
| GORE E | 5 | 0.039 |
| GONG YL | 5 | 0.039 |
| GONG TQ | 5 | 0.039 |
| GOMEZ D | 5 | 0.039 |
| GOLOZAR A | 5 | 0.039 |
| GOLDBOHM RA | 5 | 0.039 |
| GOH KL | 5 | 0.039 |
| GLATZ T | 5 | 0.039 |
| GLASGOW RE | 5 | 0.039 |
| GIMOTTY PA | 5 | 0.039 |
| GIERCKSKY KE | 5 | 0.039 |
| GHOSH S | 5 | 0.039 |
| GHADBAN T | 5 | 0.039 |
| GEIJSEN ED | 5 | 0.039 |
| GAO Q | 5 | 0.039 |
| GANAI BA | 5 | 0.039 |
| GALLUS S | 5 | 0.039 |
| GALEONE C | 5 | 0.039 |
| GAIL MH | 5 | 0.039 |
| GABRIEL E | 5 | 0.039 |
| FURUKITA Y | 5 | 0.039 |
| FURUKAWA H | 5 | 0.039 |
| FUKAYA M | 5 | 0.039 |
| FUKAGAWA T | 5 | 0.039 |
| FUJIYAMA Y | 5 | 0.039 |
| FUJITANI K | 5 | 0.039 |
| FUJITA I | 5 | 0.039 |
| FREILICH J | 5 | 0.039 |
| FREEMAN RK | 5 | 0.039 |
| FREDERICKSEN MB | 5 | 0.039 |
| FORCE SD | 5 | 0.039 |
| FONT A | 5 | 0.039 |
| FISICHELLA PM | 5 | 0.039 |
| FILIBERTI R | 5 | 0.039 |
| FETZNER UK | 5 | 0.039 |
| FERRI L | 5 | 0.039 |
| FERNANDO HC | 5 | 0.039 |
| FERNANDEZ-ESPARRACH G | 5 | 0.039 |
| FERNANDEZ FG | 5 | 0.039 |
| FENG J | 5 | 0.039 |
| FENG H | 5 | 0.039 |
| FENG F | 5 | 0.039 |
| FEDIRKO V | 5 | 0.039 |
| FAN ZN | 5 | 0.039 |
| FAN M | 5 | 0.039 |
| FALK G | 5 | 0.039 |
| ENG L | 5 | 0.039 |
| EL-RAYES BF | 5 | 0.039 |
| EL-OMAR EM | 5 | 0.039 |
| EDET-SANSON A | 5 | 0.039 |
| EDEFONTI V | 5 | 0.039 |
| EARLY DS | 5 | 0.039 |
| DVORAK K | 5 | 0.039 |
| DUNAGAN KT | 5 | 0.039 |
| DUCROTTE P | 5 | 0.039 |
| DUBRAY B | 5 | 0.039 |
| DUBEY S | 5 | 0.039 |
| DU W | 5 | 0.039 |
| DU H | 5 | 0.039 |
| DONG M | 5 | 0.039 |
| DONG L | 5 | 0.039 |
| DONAHUE DM | 5 | 0.039 |
| DOMEYER M | 5 | 0.039 |
| DOMEKI Y | 5 | 0.039 |
| DOMAGK D | 5 | 0.039 |
| DOI Y | 5 | 0.039 |
| DOHERTY M | 5 | 0.039 |
| DIPETRILLO T | 5 | 0.039 |
| DING JH | 5 | 0.039 |
| DIETLEIN M | 5 | 0.039 |
| DIDDEN P | 5 | 0.039 |
| DIAKOWSKA D | 5 | 0.039 |
| DEWITT J | 5 | 0.039 |
| DEPREZ PH | 5 | 0.039 |
| DENLINGER CS | 5 | 0.039 |
| DENLINGER CE | 5 | 0.039 |
| DEMEESTER T | 5 | 0.039 |
| DEL GENIO A | 5 | 0.039 |
| DE HERTOGH G | 5 | 0.039 |
| DARZI A | 5 | 0.039 |
| DAL MASO L | 5 | 0.039 |
| DAI SQ | 5 | 0.039 |
| CUMMINGS LC | 5 | 0.039 |
| CRUZ A | 5 | 0.039 |
| CORDIANO C | 5 | 0.039 |
| COPPOLA D | 5 | 0.039 |
| COOLS-LARTIGUE J | 5 | 0.039 |
| COHEN DJ | 5 | 0.039 |
| CHYCZEWSKI L | 5 | 0.039 |
| CHOU TY | 5 | 0.039 |
| CHOJNACKI KA | 5 | 0.039 |
| CHOI YS | 5 | 0.039 |
| CHOI J | 5 | 0.039 |
| CHO YK | 5 | 0.039 |
| CHO H | 5 | 0.039 |
| CHIU HM | 5 | 0.039 |
| CHIU CT | 5 | 0.039 |
| CHIANG YJ | 5 | 0.039 |
| CHEUNG WY | 5 | 0.039 |
| CHEONG JH | 5 | 0.039 |
| CHENG DX | 5 | 0.039 |
| CHENG AL | 5 | 0.039 |
| CHEN ZY | 5 | 0.039 |
| CHEN ZG | 5 | 0.039 |
| CHEN YX | 5 | 0.039 |
| CHEN YW | 5 | 0.039 |
| CHEN XJ | 5 | 0.039 |
| CHEN XB | 5 | 0.039 |
| CHEN WG | 5 | 0.039 |
| CHEN SW | 5 | 0.039 |
| CHEN SF | 5 | 0.039 |
| CHEN PS | 5 | 0.039 |
| CHEN ML | 5 | 0.039 |
| CHEN KS | 5 | 0.039 |
| CHEN JJ | 5 | 0.039 |
| CHEN HC | 5 | 0.039 |
| CHEN GN | 5 | 0.039 |
| CHEN CZ | 5 | 0.039 |
| CHATTERJEE A | 5 | 0.039 |
| CHANDRAWANSA K | 5 | 0.039 |
| CHAN TF | 5 | 0.039 |
| CHAN DS | 5 | 0.039 |
| CHAN D | 5 | 0.039 |
| CHA J | 5 | 0.039 |
| CERVINO AR | 5 | 0.039 |
| CERVANTES A | 5 | 0.039 |
| CEELEN W | 5 | 0.039 |
| CASH BD | 5 | 0.039 |
| CARRERE N | 5 | 0.039 |
| CARDWELL CR | 5 | 0.039 |
| CAO CN | 5 | 0.039 |
| CAMPBELL J | 5 | 0.039 |
| CAMPAGNA MC | 5 | 0.039 |
| BURTNESS B | 5 | 0.039 |
| BURTIN P | 5 | 0.039 |
| BUENO R | 5 | 0.039 |
| BRYANT AS | 5 | 0.039 |
| BRUWER M | 5 | 0.039 |
| BRUNNER T | 5 | 0.039 |
| BRUCHER BLDM | 5 | 0.039 |
| BROWN J | 5 | 0.039 |
| BRONSERT P | 5 | 0.039 |
| BROCK M | 5 | 0.039 |
| BRANDT B | 5 | 0.039 |
| BOUCHARDY C | 5 | 0.039 |
| BOTERBERG T | 5 | 0.039 |
| BORNSCHEIN J | 5 | 0.039 |
| BOONSTRA JJ | 5 | 0.039 |
| BOCUS P | 5 | 0.039 |
| BLESSMANN M | 5 | 0.039 |
| BLASZKOWSKY LS | 5 | 0.039 |
| BLANCHI S | 5 | 0.039 |
| BIBEAU F | 5 | 0.039 |
| BEUKEMA J | 5 | 0.039 |
| BERRISFORD RG | 5 | 0.039 |
| BERMAN AT | 5 | 0.039 |
| BENYOUCEF A | 5 | 0.039 |
| BENHAMOU S | 5 | 0.039 |
| BEN-JOSEF E | 5 | 0.039 |
| BELKHIRI A | 5 | 0.039 |
| BEKAII-SAAB TS | 5 | 0.039 |
| BEKAII-SAAB T | 5 | 0.039 |
| BEALES ILP | 5 | 0.039 |
| BASU S | 5 | 0.039 |
| BARZAN L | 5 | 0.039 |
| BARTHEL J | 5 | 0.039 |
| BARRON JP | 5 | 0.039 |
| BARON TH | 5 | 0.039 |
| BAR-SELA G | 5 | 0.039 |
| BANDLA S | 5 | 0.039 |
| BAKIS G | 5 | 0.039 |
| BAJPAI M | 5 | 0.039 |
| BAI JF | 5 | 0.039 |
| BADYLAK SF | 5 | 0.039 |
| AWUT I | 5 | 0.039 |
| AUJESKY R | 5 | 0.039 |
| ASAO T | 5 | 0.039 |
| ARIMURA Y | 5 | 0.039 |
| ARIMA H | 5 | 0.039 |
| ARIGA T | 5 | 0.039 |
| AOYAGI T | 5 | 0.039 |
| AOYAGI K | 5 | 0.039 |
| ANTONIETTI M | 5 | 0.039 |
| ANKER CJ | 5 | 0.039 |
| ANDOH A | 5 | 0.039 |
| ANDERSSON M | 5 | 0.039 |
| AMANO S | 5 | 0.039 |
| ALLUM W | 5 | 0.039 |
| ALLAIX ME | 5 | 0.039 |
| ALEXANDRE L | 5 | 0.039 |
| ALBANO RM | 5 | 0.039 |
| AKAZAWA K | 5 | 0.039 |
| AKAGI I | 5 | 0.039 |
| AIDA J | 5 | 0.039 |
| AHRENS W | 5 | 0.039 |
| AHN YC | 5 | 0.039 |
| AGUDO A | 5 | 0.039 |
| AGRAWAL A | 5 | 0.039 |
| ADUSUMILLI PS | 5 | 0.039 |
| ACHIAM MP | 5 | 0.039 |
| ABRAHAM SC | 5 | 0.039 |
| ABATE E | 5 | 0.039 |
| ZYLSTRA J | 4 | 0.031 |
| ZWISCHENBERGER JB | 4 | 0.031 |
| ZUO LF | 4 | 0.031 |
| ZUIKI T | 4 | 0.031 |
| ZUCCHETTO A | 4 | 0.031 |
| ZOU YB | 4 | 0.031 |
| ZOU SM | 4 | 0.031 |
| ZNAOR A | 4 | 0.031 |
| ZIRLIK K | 4 | 0.031 |
| ZHU ZL | 4 | 0.031 |
| ZHU XY | 4 | 0.031 |
| ZHU XL | 4 | 0.031 |
| ZHU WG | 4 | 0.031 |
| ZHU SM | 4 | 0.031 |
| ZHU R | 4 | 0.031 |
| ZHU ML | 4 | 0.031 |
| ZHU LL | 4 | 0.031 |
| ZHU CC | 4 | 0.031 |
| ZHOU ZW | 4 | 0.031 |
| ZHOU ZR | 4 | 0.031 |
| ZHOU ZH | 4 | 0.031 |
| ZHOU XM | 4 | 0.031 |
| ZHOU XB | 4 | 0.031 |
| ZHOU SH | 4 | 0.031 |
| ZHOU RM | 4 | 0.031 |
| ZHOU JN | 4 | 0.031 |
| ZHOU D | 4 | 0.031 |
| ZHOU B | 4 | 0.031 |
| ZHONG DS | 4 | 0.031 |
| ZHENG YF | 4 | 0.031 |
| ZHENG YB | 4 | 0.031 |
| ZHENG LF | 4 | 0.031 |
| ZHENG B | 4 | 0.031 |
| ZHAO ZM | 4 | 0.031 |
| ZHAO XY | 4 | 0.031 |
| ZHAO HG | 4 | 0.031 |
| ZHAO F | 4 | 0.031 |
| ZHANG ZX | 4 | 0.031 |
| ZHANG ZM | 4 | 0.031 |
| ZHANG ZH | 4 | 0.031 |
| ZHANG YS | 4 | 0.031 |
| ZHANG YB | 4 | 0.031 |
| ZHANG XT | 4 | 0.031 |
| ZHANG XC | 4 | 0.031 |
| ZHANG WC | 4 | 0.031 |
| ZHANG WB | 4 | 0.031 |
| ZHANG RQ | 4 | 0.031 |
| ZHANG QH | 4 | 0.031 |
| ZHANG PP | 4 | 0.031 |
| ZHANG LM | 4 | 0.031 |
| ZHANG JP | 4 | 0.031 |
| ZHANG JF | 4 | 0.031 |
| ZHANG HQ | 4 | 0.031 |
| ZHANG HJ | 4 | 0.031 |
| ZHANG FC | 4 | 0.031 |
| ZHANG D | 4 | 0.031 |
| ZHANG CL | 4 | 0.031 |
| ZERAATI H | 4 | 0.031 |
| ZENG YX | 4 | 0.031 |
| ZENG XX | 4 | 0.031 |
| ZARE M | 4 | 0.031 |
| ZACHARAKIS E | 4 | 0.031 |
| YUAN X | 4 | 0.031 |
| YUAN SH | 4 | 0.031 |
| YUAN P | 4 | 0.031 |
| YU ZC | 4 | 0.031 |
| YU SZ | 4 | 0.031 |
| YU P | 4 | 0.031 |
| YU HL | 4 | 0.031 |
| YU DK | 4 | 0.031 |
| YU CY | 4 | 0.031 |
| YU B | 4 | 0.031 |
| YOU NCY | 4 | 0.031 |
| YOSHITAKE Y | 4 | 0.031 |
| YOSHIO T | 4 | 0.031 |
| YONEYAMA K | 4 | 0.031 |
| YOKOYAMA M | 4 | 0.031 |
| YOKOE T | 4 | 0.031 |
| YIP S | 4 | 0.031 |
| YINGJIE Z | 4 | 0.031 |
| YIN R | 4 | 0.031 |
| YILMAZ O | 4 | 0.031 |
| YI SY | 4 | 0.031 |
| YI J | 4 | 0.031 |
| YEN TC | 4 | 0.031 |
| YEN RF | 4 | 0.031 |
| YEN KY | 4 | 0.031 |
| YEH KH | 4 | 0.031 |
| YEH HL | 4 | 0.031 |
| YE Y | 4 | 0.031 |
| YE T | 4 | 0.031 |
| YE L | 4 | 0.031 |
| YE F | 4 | 0.031 |
| YE B | 4 | 0.031 |
| YAO Y | 4 | 0.031 |
| YAO JC | 4 | 0.031 |
| YAO J | 4 | 0.031 |
| YANG YX | 4 | 0.031 |
| YANG SM | 4 | 0.031 |
| YANG SL | 4 | 0.031 |
| YANG QC | 4 | 0.031 |
| YANG N | 4 | 0.031 |
| YANG JZ | 4 | 0.031 |
| YANG JF | 4 | 0.031 |
| YANG HC | 4 | 0.031 |
| YANG CX | 4 | 0.031 |
| YANAGA K | 4 | 0.031 |
| YAN XL | 4 | 0.031 |
| YAMAZOE S | 4 | 0.031 |
| YAMAZAKI H | 4 | 0.031 |
| YAMATO M | 4 | 0.031 |
| YAMASHITA M | 4 | 0.031 |
| YAMAO K | 4 | 0.031 |
| YAMAMICHI N | 4 | 0.031 |
| YAMAKITA I | 4 | 0.031 |
| YAMADA H | 4 | 0.031 |
| YALCIN S | 4 | 0.031 |
| YAGI N | 4 | 0.031 |
| YACHIMSKI P | 4 | 0.031 |
| YABUSAKI H | 4 | 0.031 |
| XU ZF | 4 | 0.031 |
| XU XH | 4 | 0.031 |
| XU S | 4 | 0.031 |
| XU GZ | 4 | 0.031 |
| XU BH | 4 | 0.031 |
| XU AM | 4 | 0.031 |
| XIONG W | 4 | 0.031 |
| XIONG JP | 4 | 0.031 |
| XIONG HL | 4 | 0.031 |
| XING LX | 4 | 0.031 |
| XIE LX | 4 | 0.031 |
| XIE JD | 4 | 0.031 |
| XIAO JP | 4 | 0.031 |
| XIANG J | 4 | 0.031 |
| WU YW | 4 | 0.031 |
| WU YH | 4 | 0.031 |
| WU TC | 4 | 0.031 |
| WU SX | 4 | 0.031 |
| WU QQ | 4 | 0.031 |
| WU MY | 4 | 0.031 |
| WU MH | 4 | 0.031 |
| WU JY | 4 | 0.031 |
| WU G | 4 | 0.031 |
| WU CL | 4 | 0.031 |
| WU CH | 4 | 0.031 |
| WU CC | 4 | 0.031 |
| WU AW | 4 | 0.031 |
| WORNI M | 4 | 0.031 |
| WONG SKH | 4 | 0.031 |
| WOLFSEN H | 4 | 0.031 |
| WOLFE R | 4 | 0.031 |
| WLODARCZYK J | 4 | 0.031 |
| WINTER K | 4 | 0.031 |
| WIGGINS T | 4 | 0.031 |
| WIEDER HA | 4 | 0.031 |
| WIEDER H | 4 | 0.031 |
| WEN YW | 4 | 0.031 |
| WELSCH J | 4 | 0.031 |
| WELLNER U | 4 | 0.031 |
| WEIGEL T | 4 | 0.031 |
| WEI YT | 4 | 0.031 |
| WEI JY | 4 | 0.031 |
| WEI C | 4 | 0.031 |
| WEBER SM | 4 | 0.031 |
| WEBER M | 4 | 0.031 |
| WATANABE A | 4 | 0.031 |
| WANG ZF | 4 | 0.031 |
| WANG YP | 4 | 0.031 |
| WANG YM | 4 | 0.031 |
| WANG YB | 4 | 0.031 |
| WANG XP | 4 | 0.031 |
| WANG XD | 4 | 0.031 |
| WANG WW | 4 | 0.031 |
| WANG WJ | 4 | 0.031 |
| WANG QY | 4 | 0.031 |
| WANG MH | 4 | 0.031 |
| WANG LJ | 4 | 0.031 |
| WANG JQ | 4 | 0.031 |
| WANG HL | 4 | 0.031 |
| WANG HF | 4 | 0.031 |
| WANG CJ | 4 | 0.031 |
| WANG BS | 4 | 0.031 |
| WAN Y | 4 | 0.031 |
| WAN X | 4 | 0.031 |
| WAJED SA | 4 | 0.031 |
| WAIN J | 4 | 0.031 |
| VULQUIN N | 4 | 0.031 |
| VRBA R | 4 | 0.031 |
| VOSS KA | 4 | 0.031 |
| VON LOGA K | 4 | 0.031 |
| VON HEYDEBRECK A | 4 | 0.031 |
| VON FLUE M | 4 | 0.031 |
| VON DOBELN GA | 4 | 0.031 |
| VOMACKOVA K | 4 | 0.031 |
| VOLLSET SE | 4 | 0.031 |
| VLIEGEN RFA | 4 | 0.031 |
| VITIELLO F | 4 | 0.031 |
| VIGNESH S | 4 | 0.031 |
| VETTORAZZI E | 4 | 0.031 |
| VERSCHUUR EML | 4 | 0.031 |
| VERHEUL HMW | 4 | 0.031 |
| VENNALAGANTI P | 4 | 0.031 |
| VENERITO M | 4 | 0.031 |
| VEGH I | 4 | 0.031 |
| VARGHESE TK | 4 | 0.031 |
| VAN'T VEER P | 4 | 0.031 |
| VAN WIERINGEN N | 4 | 0.031 |
| VAN VEER H | 4 | 0.031 |
| VAN RAEMDONCK D | 4 | 0.031 |
| VAN NIEUWENHOVE Y | 4 | 0.031 |
| VAN LAETHEM JL | 4 | 0.031 |
| VAN DER SCHAAF M | 4 | 0.031 |
| URBA SG | 4 | 0.031 |
| URAKAWA N | 4 | 0.031 |
| UMENO H | 4 | 0.031 |
| UITDEHAAG MJ | 4 | 0.031 |
| UENAKA A | 4 | 0.031 |
| UCHIYAMA K | 4 | 0.031 |
| UCHIDA K | 4 | 0.031 |
| TURNER PC | 4 | 0.031 |
| TSUTSUMI E | 4 | 0.031 |
| TSUJI T | 4 | 0.031 |
| TSUJI K | 4 | 0.031 |
| TSUDA Y | 4 | 0.031 |
| TSENG JF | 4 | 0.031 |
| TSAI TH | 4 | 0.031 |
| TRUONG MT | 4 | 0.031 |
| TRUMP F | 4 | 0.031 |
| TROUSSE D | 4 | 0.031 |
| TROJAN J | 4 | 0.031 |
| TRAVIS RC | 4 | 0.031 |
| TOYOZUMI T | 4 | 0.031 |
| TOYONAGA T | 4 | 0.031 |
| TORIGOE T | 4 | 0.031 |
| TONINI G | 4 | 0.031 |
| TONG D | 4 | 0.031 |
| TOMONAGA T | 4 | 0.031 |
| TOMIZAWA Y | 4 | 0.031 |
| TODAKA A | 4 | 0.031 |
| TODA K | 4 | 0.031 |
| TIXIER F | 4 | 0.031 |
| TIWARI A | 4 | 0.031 |
| TIMME S | 4 | 0.031 |
| TIAN J | 4 | 0.031 |
| TIAN F | 4 | 0.031 |
| THUREAU S | 4 | 0.031 |
| THOSANI N | 4 | 0.031 |
| THOMPSON J | 4 | 0.031 |
| THOMAS RJS | 4 | 0.031 |
| THOMAS JM | 4 | 0.031 |
| THEODOROU D | 4 | 0.031 |
| TEYTON P | 4 | 0.031 |
| TESHIMA J | 4 | 0.031 |
| TERAZAWA T | 4 | 0.031 |
| TE MORSCHE RHM | 4 | 0.031 |
| TAYLOR MD | 4 | 0.031 |
| TASHI T | 4 | 0.031 |
| TAPIAS LF | 4 | 0.031 |
| TANIZAWA Y | 4 | 0.031 |
| TANIYAMA Y | 4 | 0.031 |
| TANIMOTO K | 4 | 0.031 |
| TANG ZZ | 4 | 0.031 |
| TANG Y | 4 | 0.031 |
| TANG WR | 4 | 0.031 |
| TANG W | 4 | 0.031 |
| TANG RX | 4 | 0.031 |
| TANG P | 4 | 0.031 |
| TANG JT | 4 | 0.031 |
| TANG JCO | 4 | 0.031 |
| TANG H | 4 | 0.031 |
| TAN L | 4 | 0.031 |
| TAN DF | 4 | 0.031 |
| TAMURA Y | 4 | 0.031 |
| TAMARU J | 4 | 0.031 |
| TAMAMURA H | 4 | 0.031 |
| TAMAMOTO T | 4 | 0.031 |
| TAMAKOSHI A | 4 | 0.031 |
| TAMAKI T | 4 | 0.031 |
| TAKEMASA I | 4 | 0.031 |
| TAKEHARA T | 4 | 0.031 |
| TAKEDA Y | 4 | 0.031 |
| TAKEDA S | 4 | 0.031 |
| TAKECHI H | 4 | 0.031 |
| TAKEBAYASHI K | 4 | 0.031 |
| TAKATA A | 4 | 0.031 |
| TAKASAWA S | 4 | 0.031 |
| TAKANO T | 4 | 0.031 |
| TAKAISHI H | 4 | 0.031 |
| TAKAGI T | 4 | 0.031 |
| TAKAGI S | 4 | 0.031 |
| TAKAGI R | 4 | 0.031 |
| TAKADA A | 4 | 0.031 |
| TAGAWA M | 4 | 0.031 |
| TACHIBANA S | 4 | 0.031 |
| TACHEZY M | 4 | 0.031 |
| SYED AA | 4 | 0.031 |
| SWANSON SJ | 4 | 0.031 |
| SUWA Y | 4 | 0.031 |
| SUNG JJY | 4 | 0.031 |
| SUNDARESAN S | 4 | 0.031 |
| SUN Z | 4 | 0.031 |
| SUN W | 4 | 0.031 |
| SUN MP | 4 | 0.031 |
| SUN LC | 4 | 0.031 |
| SUN JM | 4 | 0.031 |
| SUN HB | 4 | 0.031 |
| SUN F | 4 | 0.031 |
| SULMAN J | 4 | 0.031 |
| SULLIVAN TR | 4 | 0.031 |
| SUITO H | 4 | 0.031 |
| SUGITO N | 4 | 0.031 |
| SUGIMURA H | 4 | 0.031 |
| SUGIMOTO N | 4 | 0.031 |
| SUGIMORI S | 4 | 0.031 |
| SUGIHARA K | 4 | 0.031 |
| SUEYOSHI S | 4 | 0.031 |
| SUENAGA M | 4 | 0.031 |
| SU YH | 4 | 0.031 |
| SU MY | 4 | 0.031 |
| SU J | 4 | 0.031 |
| SU HF | 4 | 0.031 |
| STRUTYNSKA-KARPINSKA M | 4 | 0.031 |
| STRAUB D | 4 | 0.031 |
| STOLL L | 4 | 0.031 |
| STOEHLMACHER J | 4 | 0.031 |
| STEPANOV I | 4 | 0.031 |
| STEIN A | 4 | 0.031 |
| STEFFEN A | 4 | 0.031 |
| STAUDER MC | 4 | 0.031 |
| STAMOS MJ | 4 | 0.031 |
| STALEY CA | 4 | 0.031 |
| SQUIRES MH | 4 | 0.031 |
| SPRINGFELD C | 4 | 0.031 |
| SPARANO JA | 4 | 0.031 |
| SOUQUET JC | 4 | 0.031 |
| SONTAG SJ | 4 | 0.031 |
| SONG YC | 4 | 0.031 |
| SONG PP | 4 | 0.031 |
| SONG LB | 4 | 0.031 |
| SONG L | 4 | 0.031 |
| SONG JH | 4 | 0.031 |
| SONG IS | 4 | 0.031 |
| SONG B | 4 | 0.031 |
| SOMDYALA NIM | 4 | 0.031 |
| SOLERIO D | 4 | 0.031 |
| SOINI Y | 4 | 0.031 |
| SOERJOMATARAM I | 4 | 0.031 |
| SOBUE T | 4 | 0.031 |
| SO JBY | 4 | 0.031 |
| SMOLLE-JUETTNER FM | 4 | 0.031 |
| SMITH M | 4 | 0.031 |
| SMIT VTHBM | 4 | 0.031 |
| SLOTTA-HUSPENINA J | 4 | 0.031 |
| SLATER JD | 4 | 0.031 |
| SKINNER H | 4 | 0.031 |
| SINGH M | 4 | 0.031 |
| SIMONATO L | 4 | 0.031 |
| SIHVO E | 4 | 0.031 |
| SIEWERT J | 4 | 0.031 |
| SIEGEL BA | 4 | 0.031 |
| SHIWAKU H | 4 | 0.031 |
| SHIRAI Y | 4 | 0.031 |
| SHIRABE K | 4 | 0.031 |
| SHIN MH | 4 | 0.031 |
| SHIMOSEGAWA E | 4 | 0.031 |
| SHIMIZU M | 4 | 0.031 |
| SHIMAMOTO F | 4 | 0.031 |
| SHIMADA A | 4 | 0.031 |
| SHIELDS AF | 4 | 0.031 |
| SHIBUYA H | 4 | 0.031 |
| SHIBATA R | 4 | 0.031 |
| SHIBATA K | 4 | 0.031 |
| SHIBA M | 4 | 0.031 |
| SHI SS | 4 | 0.031 |
| SHI RH | 4 | 0.031 |
| SHI LR | 4 | 0.031 |
| SHEU BS | 4 | 0.031 |
| SHERMAN S | 4 | 0.031 |
| SHENG W | 4 | 0.031 |
| SHEN ZY | 4 | 0.031 |
| SHEN WB | 4 | 0.031 |
| SHEN LX | 4 | 0.031 |
| SHEN JX | 4 | 0.031 |
| SHEN HC | 4 | 0.031 |
| SHARMA V | 4 | 0.031 |
| SHARMA RA | 4 | 0.031 |
| SHANG D | 4 | 0.031 |
| SHAN L | 4 | 0.031 |
| SHAH S | 4 | 0.031 |
| SEWRAM V | 4 | 0.031 |
| SERGEEVA NN | 4 | 0.031 |
| SEKI S | 4 | 0.031 |
| SEKI N | 4 | 0.031 |
| SEIKE J | 4 | 0.031 |
| SCOTT W | 4 | 0.031 |
| SCHWARZBACH M | 4 | 0.031 |
| SCHUMACHER C | 4 | 0.031 |
| SCHULTHEIS A | 4 | 0.031 |
| SCHOPPMANN S | 4 | 0.031 |
| SCHMIDT D | 4 | 0.031 |
| SCHLUCHTER MD | 4 | 0.031 |
| SCHLAG PM | 4 | 0.031 |
| SCHEMBRE D | 4 | 0.031 |
| SCHEFTER TE | 4 | 0.031 |
| SCHAFER G | 4 | 0.031 |
| SCARTOZZI M | 4 | 0.031 |
| SAWYER MB | 4 | 0.031 |
| SAUER IM | 4 | 0.031 |
| SASSON AR | 4 | 0.031 |
| SASAZUKI S | 4 | 0.031 |
| SASANO H | 4 | 0.031 |
| SARBIA M | 4 | 0.031 |
| SANTINI D | 4 | 0.031 |
| SANCHEZ-CANTALEJO E | 4 | 0.031 |
| SANADA Y | 4 | 0.031 |
| SAMADI F | 4 | 0.031 |
| SALAHI R | 4 | 0.031 |
| SAKURAI Y | 4 | 0.031 |
| SAKATA K | 4 | 0.031 |
| SAKAMOTO T | 4 | 0.031 |
| SAKAMOTO S | 4 | 0.031 |
| SAKAMOTO J | 4 | 0.031 |
| SAKAMOTO H | 4 | 0.031 |
| SAITO R | 4 | 0.031 |
| SAIF MW | 4 | 0.031 |
| SAIEVA C | 4 | 0.031 |
| SAEED N | 4 | 0.031 |
| SABA NF | 4 | 0.031 |
| RYU MH | 4 | 0.031 |
| RYU D | 4 | 0.031 |
| RYOTOKUJI T | 4 | 0.031 |
| RUTH M | 4 | 0.031 |
| RUSZKIEWICZ AR | 4 | 0.031 |
| ROTHSTEIN RI | 4 | 0.031 |
| ROTHMAN N | 4 | 0.031 |
| ROTHLING N | 4 | 0.031 |
| ROSENBERG SA | 4 | 0.031 |
| ROMIEU I | 4 | 0.031 |
| ROHREN E | 4 | 0.031 |
| RODRIGUES MAM | 4 | 0.031 |
| ROBLES J | 4 | 0.031 |
| ROBINSON M | 4 | 0.031 |
| RITTER MA | 4 | 0.031 |
| RINTALA RJ | 4 | 0.031 |
| RICHIARDI L | 4 | 0.031 |
| RHEE JC | 4 | 0.031 |
| REVELS SL | 4 | 0.031 |
| REN H | 4 | 0.031 |
| REINHECKEL T | 4 | 0.031 |
| REICHARDT P | 4 | 0.031 |
| REHDERS A | 4 | 0.031 |
| REDDY R | 4 | 0.031 |
| READ M | 4 | 0.031 |
| RAY R | 4 | 0.031 |
| RAWNAQ T | 4 | 0.031 |
| RATTNER DW | 4 | 0.031 |
| RAOUL JL | 4 | 0.031 |
| RAO JY | 4 | 0.031 |
| RAMADORI G | 4 | 0.031 |
| RALHAN R | 4 | 0.031 |
| RAKHSHANI N | 4 | 0.031 |
| RAIMONDO M | 4 | 0.031 |
| RAI AK | 4 | 0.031 |
| RAFIQ R | 4 | 0.031 |
| RACINE A | 4 | 0.031 |
| RABEN A | 4 | 0.031 |
| QURESHI I | 4 | 0.031 |
| QUEISSER A | 4 | 0.031 |
| QUAAS A | 4 | 0.031 |
| QIN Y | 4 | 0.031 |
| QIN W | 4 | 0.031 |
| QIN J | 4 | 0.031 |
| QIAO LL | 4 | 0.031 |
| QIAO L | 4 | 0.031 |
| QI RZ | 4 | 0.031 |
| PROLLA JC | 4 | 0.031 |
| PRICE P | 4 | 0.031 |
| PRENZEL K | 4 | 0.031 |
| POSNER M | 4 | 0.031 |
| POSEY JA | 4 | 0.031 |
| POLKOWSKI M | 4 | 0.031 |
| POHLENZ P | 4 | 0.031 |
| PIZZI M | 4 | 0.031 |
| PINTO H | 4 | 0.031 |
| PINI S | 4 | 0.031 |
| PING YM | 4 | 0.031 |
| PIETROBON R | 4 | 0.031 |
| PIDGEON GP | 4 | 0.031 |
| PHOKU JZ | 4 | 0.031 |
| PHAROAH P | 4 | 0.031 |
| PETTY RD | 4 | 0.031 |
| PETRUZELKA L | 4 | 0.031 |
| PERRY K | 4 | 0.031 |
| PEPPELENBOSCH MP | 4 | 0.031 |
| PENG Y | 4 | 0.031 |
| PENG F | 4 | 0.031 |
| PENA L | 4 | 0.031 |
| PELDSCHUS K | 4 | 0.031 |
| PEIPER M | 4 | 0.031 |
| PAZIANAS M | 4 | 0.031 |
| PAWLIK TM | 4 | 0.031 |
| PATEL P | 4 | 0.031 |
| PASRICHA S | 4 | 0.031 |
| PARKIN DM | 4 | 0.031 |
| PARKER RK | 4 | 0.031 |
| PARK SM | 4 | 0.031 |
| PARK S | 4 | 0.031 |
| PARK JS | 4 | 0.031 |
| PARIPATI H | 4 | 0.031 |
| PAPP A | 4 | 0.031 |
| PANG MH | 4 | 0.031 |
| PANDEY HP | 4 | 0.031 |
| PAN XF | 4 | 0.031 |
| PAN WT | 4 | 0.031 |
| PAN P | 4 | 0.031 |
| PAN L | 4 | 0.031 |
| PAKARINEN MP | 4 | 0.031 |
| PAIREDER M | 4 | 0.031 |
| PAILLOT B | 4 | 0.031 |
| OZAWA D | 4 | 0.031 |
| OVERMAN MJ | 4 | 0.031 |
| OVERGAARD J | 4 | 0.031 |
| OTSUKA K | 4 | 0.031 |
| OTOWA Y | 4 | 0.031 |
| OTA H | 4 | 0.031 |
| OSAKI T | 4 | 0.031 |
| ORITA H | 4 | 0.031 |
| ONO Y | 4 | 0.031 |
| ONISHI H | 4 | 0.031 |
| OLD LJ | 4 | 0.031 |
| OKINES A | 4 | 0.031 |
| OKANO T | 4 | 0.031 |
| OKAMOTO Y | 4 | 0.031 |
| OKAMOTO T | 4 | 0.031 |
| OKADA Y | 4 | 0.031 |
| OKADA S | 4 | 0.031 |
| OKA S | 4 | 0.031 |
| OKA D | 4 | 0.031 |
| OHNISHI H | 4 | 0.031 |
| OHKAWA S | 4 | 0.031 |
| OH SC | 4 | 0.031 |
| OH DS | 4 | 0.031 |
| OGUCHI M | 4 | 0.031 |
| OGAWA O | 4 | 0.031 |
| OGATA T | 4 | 0.031 |
| ODAJIMA H | 4 | 0.031 |
| ODA Y | 4 | 0.031 |
| OATES J | 4 | 0.031 |
| O'ROURKE RW | 4 | 0.031 |
| O'BYRNE K | 4 | 0.031 |
| NWOGU C | 4 | 0.031 |
| NOSHO K | 4 | 0.031 |
| NODA S | 4 | 0.031 |
| NOARO G | 4 | 0.031 |
| NOACK F | 4 | 0.031 |
| NKHALI L | 4 | 0.031 |
| NITTI D | 4 | 0.031 |
| NISHIMURA H | 4 | 0.031 |
| NISHIGORI T | 4 | 0.031 |
| NISHIDA N | 4 | 0.031 |
| NING ZH | 4 | 0.031 |
| NING T | 4 | 0.031 |
| NIIMI K | 4 | 0.031 |
| NIEPONICE A | 4 | 0.031 |
| NIEMIERKO A | 4 | 0.031 |
| NIEDZWIECKI D | 4 | 0.031 |
| NI Y | 4 | 0.031 |
| NGUYEN T | 4 | 0.031 |
| NGUYEN NP | 4 | 0.031 |
| NGUYEN DM | 4 | 0.031 |
| NGAN S | 4 | 0.031 |
| NGAMRUENGPHONG S | 4 | 0.031 |
| NEURATH MF | 4 | 0.031 |
| NEMOTO T | 4 | 0.031 |
| NELSON RA | 4 | 0.031 |
| NAVARRO C | 4 | 0.031 |
| NASIRI B | 4 | 0.031 |
| NASHIMOTO A | 4 | 0.031 |
| NASAR A | 4 | 0.031 |
| NARUSHIMA K | 4 | 0.031 |
| NARUMIYA K | 4 | 0.031 |
| NARAYANASAMY R | 4 | 0.031 |
| NAM SY | 4 | 0.031 |
| NAKAYAMA N | 4 | 0.031 |
| NAKAYAMA M | 4 | 0.031 |
| NAKATANI Y | 4 | 0.031 |
| NAKANOKO T | 4 | 0.031 |
| NAKAMURA N | 4 | 0.031 |
| NAKAJIMA A | 4 | 0.031 |
| NAKAGAWA Y | 4 | 0.031 |
| NAIK AD | 4 | 0.031 |
| NAGLE CM | 4 | 0.031 |
| NAGANUMA S | 4 | 0.031 |
| NAGAHAMA M | 4 | 0.031 |
| MUSCHWECK H | 4 | 0.031 |
| MURRAY L | 4 | 0.031 |
| MURPHY TJ | 4 | 0.031 |
| MURPHY JD | 4 | 0.031 |
| MURAKAMI N | 4 | 0.031 |
| MURAD MH | 4 | 0.031 |
| MUO CH | 4 | 0.031 |
| MUNEKAGE M | 4 | 0.031 |
| MUNDEN RF | 4 | 0.031 |
| MULLEN JT | 4 | 0.031 |
| MULDER DS | 4 | 0.031 |
| MUIJS C | 4 | 0.031 |
| MUELLER A | 4 | 0.031 |
| MU LN | 4 | 0.031 |
| MOSAVI-JARRAHI A | 4 | 0.031 |
| MORRONE FB | 4 | 0.031 |
| MOROGA T | 4 | 0.031 |
| MORIYAMA M | 4 | 0.031 |
| MORIYA H | 4 | 0.031 |
| MORIWAKI T | 4 | 0.031 |
| MORITA K | 4 | 0.031 |
| MORI H | 4 | 0.031 |
| MORADI A | 4 | 0.031 |
| MOORE J | 4 | 0.031 |
| MOON JH | 4 | 0.031 |
| MONJAZEB AM | 4 | 0.031 |
| MOLLS M | 4 | 0.031 |
| MOLLBERG N | 4 | 0.031 |
| MOHAN V | 4 | 0.031 |
| MIZUNUMA N | 4 | 0.031 |
| MIZUNO N | 4 | 0.031 |
| MIZOUE T | 4 | 0.031 |
| MIYOSHI T | 4 | 0.031 |
| MIYAZAKI M | 4 | 0.031 |
| MIYAZAKI A | 4 | 0.031 |
| MIYAMOTO K | 4 | 0.031 |
| MIYAMOTO A | 4 | 0.031 |
| MIYAGI Y | 4 | 0.031 |
| MIWA K | 4 | 0.031 |
| MITCHELL JD | 4 | 0.031 |
| MISTRY RC | 4 | 0.031 |
| MISHRA AK | 4 | 0.031 |
| MIRO M | 4 | 0.031 |
| MIR MM | 4 | 0.031 |
| MINAMINO H | 4 | 0.031 |
| MINA S | 4 | 0.031 |
| MIN YW | 4 | 0.031 |
| MIKI C | 4 | 0.031 |
| MICHIELETTO S | 4 | 0.031 |
| MICEV M | 4 | 0.031 |
| MERRELL KW | 4 | 0.031 |
| MERLETTI F | 4 | 0.031 |
| MENG YQ | 4 | 0.031 |
| MENG H | 4 | 0.031 |
| MELVIN WS | 4 | 0.031 |
| MEISTER T | 4 | 0.031 |
| MEINING A | 4 | 0.031 |
| MEHTA MP | 4 | 0.031 |
| MCLOUGHLIN J | 4 | 0.031 |
| MCKINNEY PA | 4 | 0.031 |
| MCCURDY MR | 4 | 0.031 |
| MCCORRY NK | 4 | 0.031 |
| MCCARTAN BE | 4 | 0.031 |
| MAZUMDAR M | 4 | 0.031 |
| MAYER F | 4 | 0.031 |
| MAYANAGI S | 4 | 0.031 |
| MAY KS | 4 | 0.031 |
| MAUREL J | 4 | 0.031 |
| MATSUYAMA J | 4 | 0.031 |
| MATSUMOTO K | 4 | 0.031 |
| MATSUI T | 4 | 0.031 |
| MATSUHASHI T | 4 | 0.031 |
| MATSUHASHI N | 4 | 0.031 |
| MATSUDA Y | 4 | 0.031 |
| MATSUDA K | 4 | 0.031 |
| MATHIEU LN | 4 | 0.031 |
| MATES D | 4 | 0.031 |
| MASOOD A | 4 | 0.031 |
| MASON RJ | 4 | 0.031 |
| MARTON S | 4 | 0.031 |
| MARTINEK J | 4 | 0.031 |
| MARTIN R | 4 | 0.031 |
| MARTIN J | 4 | 0.031 |
| MARTIN I | 4 | 0.031 |
| MARTI G | 4 | 0.031 |
| MARRON M | 4 | 0.031 |
| MARJANI HA | 4 | 0.031 |
| MAPLE JT | 4 | 0.031 |
| MANTZIARI S | 4 | 0.031 |
| MANSOUR D | 4 | 0.031 |
| MANJER J | 4 | 0.031 |
| MAKARI Y | 4 | 0.031 |
| MAILLARD E | 4 | 0.031 |
| MAI RQ | 4 | 0.031 |
| MAHAR A | 4 | 0.031 |
| MAGERAS GS | 4 | 0.031 |
| MAEHARA T | 4 | 0.031 |
| MAEDA S | 4 | 0.031 |
| MAEDA N | 4 | 0.031 |
| MADHUSUDAN S | 4 | 0.031 |
| MACHLENKIN S | 4 | 0.031 |
| MACFARLANE TV | 4 | 0.031 |
| MACFARLANE GJ | 4 | 0.031 |
| MACDONALD JS | 4 | 0.031 |
| MA W | 4 | 0.031 |
| MA TL | 4 | 0.031 |
| MA QL | 4 | 0.031 |
| MA JF | 4 | 0.031 |
| MA HX | 4 | 0.031 |
| MA HB | 4 | 0.031 |
| LZBICKI JR | 4 | 0.031 |
| LYNCH TJ | 4 | 0.031 |
| LUTZKE L | 4 | 0.031 |
| LUTZ MP | 4 | 0.031 |
| LUTKENHAUS LJ | 4 | 0.031 |
| LUO JW | 4 | 0.031 |
| LUND M | 4 | 0.031 |
| LUK JM | 4 | 0.031 |
| LU ZH | 4 | 0.031 |
| LU WQ | 4 | 0.031 |
| LU QJ | 4 | 0.031 |
| LU HI | 4 | 0.031 |
| LU F | 4 | 0.031 |
| LU CL | 4 | 0.031 |
| LU B | 4 | 0.031 |
| LOZAC'H P | 4 | 0.031 |
| LOUPAKIS F | 4 | 0.031 |
| LORD RVN | 4 | 0.031 |
| LORD RV | 4 | 0.031 |
| LOOMAN CWN | 4 | 0.031 |
| LOMBRISER N | 4 | 0.031 |
| LOMAS H | 4 | 0.031 |
| LOCKE GR | 4 | 0.031 |
| LO WC | 4 | 0.031 |
| LLIMPE FLR | 4 | 0.031 |
| LJUNG R | 4 | 0.031 |
| LIU ZY | 4 | 0.031 |
| LIU YY | 4 | 0.031 |
| LIU WL | 4 | 0.031 |
| LIU LJ | 4 | 0.031 |
| LIU JW | 4 | 0.031 |
| LIU JL | 4 | 0.031 |
| LIU JH | 4 | 0.031 |
| LIU HX | 4 | 0.031 |
| LIU HL | 4 | 0.031 |
| LIU HH | 4 | 0.031 |
| LIU FY | 4 | 0.031 |
| LIU FF | 4 | 0.031 |
| LIU DP | 4 | 0.031 |
| LIU CL | 4 | 0.031 |
| LIU CH | 4 | 0.031 |
| LIU BT | 4 | 0.031 |
| LISTER J | 4 | 0.031 |
| LISSOWSKA J | 4 | 0.031 |
| LIPS IM | 4 | 0.031 |
| LING F | 4 | 0.031 |
| LIN YS | 4 | 0.031 |
| LIN YB | 4 | 0.031 |
| LIN WY | 4 | 0.031 |
| LIN SY | 4 | 0.031 |
| LIN MT | 4 | 0.031 |
| LIN HC | 4 | 0.031 |
| LIN E | 4 | 0.031 |
| LIN DM | 4 | 0.031 |
| LIN DC | 4 | 0.031 |
| LIN CJ | 4 | 0.031 |
| LIAO YC | 4 | 0.031 |
| LIAO LM | 4 | 0.031 |
| LIAO LJ | 4 | 0.031 |
| LIAO LD | 4 | 0.031 |
| LIAO CT | 4 | 0.031 |
| LIANG GY | 4 | 0.031 |
| LIAKAKOS T | 4 | 0.031 |
| LI ZJ | 4 | 0.031 |
| LI ZF | 4 | 0.031 |
| LI YD | 4 | 0.031 |
| LI XR | 4 | 0.031 |
| LI XB | 4 | 0.031 |
| LI WS | 4 | 0.031 |
| LI WQ | 4 | 0.031 |
| LI SX | 4 | 0.031 |
| LI SL | 4 | 0.031 |
| LI MX | 4 | 0.031 |
| LI LJ | 4 | 0.031 |
| LI HW | 4 | 0.031 |
| LI HQ | 4 | 0.031 |
| LI HL | 4 | 0.031 |
| LI GC | 4 | 0.031 |
| LI FY | 4 | 0.031 |
| LI FF | 4 | 0.031 |
| LI DS | 4 | 0.031 |
| LI DP | 4 | 0.031 |
| LI CX | 4 | 0.031 |
| LI CL | 4 | 0.031 |
| LEWIS W | 4 | 0.031 |
| LEWIS MPN | 4 | 0.031 |
| LERUT TEMR | 4 | 0.031 |
| LEREBOURS E | 4 | 0.031 |
| LEI J | 4 | 0.031 |
| LEGGETT CL | 4 | 0.031 |
| LEE YJ | 4 | 0.031 |
| LEE KB | 4 | 0.031 |
| LEE JJ | 4 | 0.031 |
| LEE JG | 4 | 0.031 |
| LEE I | 4 | 0.031 |
| LEE E | 4 | 0.031 |
| LEE BE | 4 | 0.031 |
| LEE A | 4 | 0.031 |
| LEBRETON G | 4 | 0.031 |
| LE N | 4 | 0.031 |
| LAW SYK | 4 | 0.031 |
| LAW C | 4 | 0.031 |
| LAUGIER R | 4 | 0.031 |
| LAU CL | 4 | 0.031 |
| LAPUC G | 4 | 0.031 |
| LAO-SIRIEIX P | 4 | 0.031 |
| LANGLEY RE | 4 | 0.031 |
| LANG HK | 4 | 0.031 |
| LANDAU DB | 4 | 0.031 |
| LAMBIN P | 4 | 0.031 |
| LAMB PJ | 4 | 0.031 |
| LACHENMEIER DW | 4 | 0.031 |
| KUWAKADO S | 4 | 0.031 |
| KUSANO C | 4 | 0.031 |
| KURSCHAT N | 4 | 0.031 |
| KURITA A | 4 | 0.031 |
| KURIHARA M | 4 | 0.031 |
| KURASHINA K | 4 | 0.031 |
| KURABI A | 4 | 0.031 |
| KUO SW | 4 | 0.031 |
| KUO CJ | 4 | 0.031 |
| KUO CH | 4 | 0.031 |
| KUNZ P | 4 | 0.031 |
| KUNIMOTO F | 4 | 0.031 |
| KUMASHIRO R | 4 | 0.031 |
| KUMAR R | 4 | 0.031 |
| KUMAMOTO K | 4 | 0.031 |
| KULKE M | 4 | 0.031 |
| KULEMANN B | 4 | 0.031 |
| KRUPNICK AS | 4 | 0.031 |
| KREISEL D | 4 | 0.031 |
| KRAUS S | 4 | 0.031 |
| KRASNA M | 4 | 0.031 |
| KOZUMI M | 4 | 0.031 |
| KOZHAKHMETOV S | 4 | 0.031 |
| KOYANAGI K | 4 | 0.031 |
| KOWALSKI T | 4 | 0.031 |
| KOWALSKI LP | 4 | 0.031 |
| KOUWENHOVEN EA | 4 | 0.031 |
| KOSOVEC JE | 4 | 0.031 |
| KORN WM | 4 | 0.031 |
| KOONS A | 4 | 0.031 |
| KOONG A | 4 | 0.031 |
| KONSKI A | 4 | 0.031 |
| KONISHI M | 4 | 0.031 |
| KONISHI K | 4 | 0.031 |
| KONIGSRAINER A | 4 | 0.031 |
| KONIARIS LG | 4 | 0.031 |
| KONG Y | 4 | 0.031 |
| KONG SH | 4 | 0.031 |
| KONDA VJ | 4 | 0.031 |
| KONDA V | 4 | 0.031 |
| KOMORI Y | 4 | 0.031 |
| KOMORI T | 4 | 0.031 |
| KOMORI S | 4 | 0.031 |
| KOMATSU A | 4 | 0.031 |
| KOLSTEREN P | 4 | 0.031 |
| KOJIMA H | 4 | 0.031 |
| KOIZUMI M | 4 | 0.031 |
| KOIFMAN S | 4 | 0.031 |
| KOGO M | 4 | 0.031 |
| KOETER M | 4 | 0.031 |
| KOENIG A | 4 | 0.031 |
| KOCHMAN ML | 4 | 0.031 |
| KOCHI M | 4 | 0.031 |
| KO YC | 4 | 0.031 |
| KLINKENBIJL JHG | 4 | 0.031 |
| KLIMSTRA DS | 4 | 0.031 |
| KLEIN Y | 4 | 0.031 |
| KLAPMAN J | 4 | 0.031 |
| KJAERHEIM K | 4 | 0.031 |
| KITA M | 4 | 0.031 |
| KISHIMOTO M | 4 | 0.031 |
| KINOSHITA T | 4 | 0.031 |
| KINNARD M | 4 | 0.031 |
| KINJO T | 4 | 0.031 |
| KIMANYA ME | 4 | 0.031 |
| KIM SG | 4 | 0.031 |
| KIM MJ | 4 | 0.031 |
| KIM KW | 4 | 0.031 |
| KIM JG | 4 | 0.031 |
| KIM IJ | 4 | 0.031 |
| KIM HY | 4 | 0.031 |
| KIM DU | 4 | 0.031 |
| KIM C | 4 | 0.031 |
| KIM B | 4 | 0.031 |
| KIKUCHI Y | 4 | 0.031 |
| KHURI FR | 4 | 0.031 |
| KESHTKAR A | 4 | 0.031 |
| KEOGAN M | 4 | 0.031 |
| KELSELL DP | 4 | 0.031 |
| KELLY JJ | 4 | 0.031 |
| KAZAMA T | 4 | 0.031 |
| KAWAKAMI K | 4 | 0.031 |
| KAUSHAL M | 4 | 0.031 |
| KAUPPILA JH | 4 | 0.031 |
| KATZ JP | 4 | 0.031 |
| KATS-UGURLU G | 4 | 0.031 |
| KATO J | 4 | 0.031 |
| KATAYAMA N | 4 | 0.031 |
| KATAOKA M | 4 | 0.031 |
| KARRENBELD A | 4 | 0.031 |
| KARIMUNDACKAL G | 4 | 0.031 |
| KARIMIAN F | 4 | 0.031 |
| KARASAWA K | 4 | 0.031 |
| KARAPETIS CS | 4 | 0.031 |
| KANG MQ | 4 | 0.031 |
| KANG M | 4 | 0.031 |
| KANG JH | 4 | 0.031 |
| KANEKO H | 4 | 0.031 |
| KANAREK N | 4 | 0.031 |
| KANAMORI N | 4 | 0.031 |
| KANAI M | 4 | 0.031 |
| KANADE G | 4 | 0.031 |
| KAMIKONYA N | 4 | 0.031 |
| KAMADA T | 4 | 0.031 |
| KAJIYA Y | 4 | 0.031 |
| KAJIURA S | 4 | 0.031 |
| KAIFI J | 4 | 0.031 |
| KADOWAKI S | 4 | 0.031 |
| KACHAAMY T | 4 | 0.031 |
| KABURAGI T | 4 | 0.031 |
| JUNG H | 4 | 0.031 |
| JULOORI A | 4 | 0.031 |
| JOUVE JL | 4 | 0.031 |
| JONKER DJ | 4 | 0.031 |
| JONES GE | 4 | 0.031 |
| JONES G | 4 | 0.031 |
| JOMRICH G | 4 | 0.031 |
| JOHANSSON I | 4 | 0.031 |
| JING SW | 4 | 0.031 |
| JIN X | 4 | 0.031 |
| JIN TB | 4 | 0.031 |
| JIN P | 4 | 0.031 |
| JIN JK | 4 | 0.031 |
| JIN GF | 4 | 0.031 |
| JICK SS | 4 | 0.031 |
| JIAO XY | 4 | 0.031 |
| JIANG Z | 4 | 0.031 |
| JIANG YY | 4 | 0.031 |
| JIANG XJ | 4 | 0.031 |
| JIANG S | 4 | 0.031 |
| JIANG Q | 4 | 0.031 |
| JIANG M | 4 | 0.031 |
| JIANG HP | 4 | 0.031 |
| JIANG C | 4 | 0.031 |
| JIANBIN L | 4 | 0.031 |
| JIA RN | 4 | 0.031 |
| JI X | 4 | 0.031 |
| JI WH | 4 | 0.031 |
| JHEON S | 4 | 0.031 |
| JETER M | 4 | 0.031 |
| JESSRI M | 4 | 0.031 |
| JEON SW | 4 | 0.031 |
| JENSSEN C | 4 | 0.031 |
| JENSEN HA | 4 | 0.031 |
| JENAB M | 4 | 0.031 |
| JEMAL A | 4 | 0.031 |
| JELSKI W | 4 | 0.031 |
| JAZII FR | 4 | 0.031 |
| JAVLE MM | 4 | 0.031 |
| JAVLE M | 4 | 0.031 |
| JAROSZEWSKI D | 4 | 0.031 |
| JANOUT V | 4 | 0.031 |
| JANKU F | 4 | 0.031 |
| JANGJOO AG | 4 | 0.031 |
| JANG S | 4 | 0.031 |
| JANG JY | 4 | 0.031 |
| JAMAL MM | 4 | 0.031 |
| JAHANGIRI Y | 4 | 0.031 |
| JACOBSON BC | 4 | 0.031 |
| JACOBSEN AB | 4 | 0.031 |
| IYAMA K | 4 | 0.031 |
| IWASE H | 4 | 0.031 |
| IWANUMA Y | 4 | 0.031 |
| ITAMI A | 4 | 0.031 |
| ISOHASHI F | 4 | 0.031 |
| ISHIZUKA T | 4 | 0.031 |
| ISHIYAMA K | 4 | 0.031 |
| ISHIKAWA S | 4 | 0.031 |
| ISHII Y | 4 | 0.031 |
| ISHII N | 4 | 0.031 |
| ISHIDA K | 4 | 0.031 |
| ISBELL JM | 4 | 0.031 |
| IRANI S | 4 | 0.031 |
| IQBAL B | 4 | 0.031 |
| INOUE J | 4 | 0.031 |
| INOKUCHI H | 4 | 0.031 |
| INFANTE JR | 4 | 0.031 |
| INCULET R | 4 | 0.031 |
| INABA H | 4 | 0.031 |
| IMAWARI M | 4 | 0.031 |
| IMAOKA H | 4 | 0.031 |
| IMANO M | 4 | 0.031 |
| IMAI Y | 4 | 0.031 |
| ILHAN-MUTLU A | 4 | 0.031 |
| IKEGAMI M | 4 | 0.031 |
| IKEDA Y | 4 | 0.031 |
| IKEDA M | 4 | 0.031 |
| IINUMA H | 4 | 0.031 |
| IHSAN R | 4 | 0.031 |
| IBUKI Y | 4 | 0.031 |
| HYLAND PL | 4 | 0.031 |
| HURMUZLU M | 4 | 0.031 |
| HUNTER J | 4 | 0.031 |
| HUI Z | 4 | 0.031 |
| HUGUET F | 4 | 0.031 |
| HUEBNER K | 4 | 0.031 |
| HUBER PE | 4 | 0.031 |
| HUANG YJ | 4 | 0.031 |
| HUANG YH | 4 | 0.031 |
| HUANG XD | 4 | 0.031 |
| HUANG WT | 4 | 0.031 |
| HUANG WC | 4 | 0.031 |
| HUANG TC | 4 | 0.031 |
| HUANG S | 4 | 0.031 |
| HUANG JW | 4 | 0.031 |
| HUANG JL | 4 | 0.031 |
| HUANG H | 4 | 0.031 |
| HUANG F | 4 | 0.031 |
| HUANG CH | 4 | 0.031 |
| HU YH | 4 | 0.031 |
| HU WN | 4 | 0.031 |
| HU PP | 4 | 0.031 |
| HU CF | 4 | 0.031 |
| HSIEH MS | 4 | 0.031 |
| HSIAO M | 4 | 0.031 |
| HOSONO M | 4 | 0.031 |
| HOSODA K | 4 | 0.031 |
| HORII T | 4 | 0.031 |
| HORGAN S | 4 | 0.031 |
| HORGAN PG | 4 | 0.031 |
| HOPKINS J | 4 | 0.031 |
| HOOKER CM | 4 | 0.031 |
| HOOKER C | 4 | 0.031 |
| HONDA M | 4 | 0.031 |
| HONDA K | 4 | 0.031 |
| HOHENBERGER W | 4 | 0.031 |
| HOARE J | 4 | 0.031 |
| HIROOKA Y | 4 | 0.031 |
| HIRDES MMC | 4 | 0.031 |
| HIRAYAMA M | 4 | 0.031 |
| HIRASHIMA Y | 4 | 0.031 |
| HIRAOKA M | 4 | 0.031 |
| HIRAMATSU M | 4 | 0.031 |
| HIRAKI S | 4 | 0.031 |
| HIRAKI A | 4 | 0.031 |
| HINOHARA H | 4 | 0.031 |
| HILDEN K | 4 | 0.031 |
| HIJIOKA S | 4 | 0.031 |
| HIGUCHI T | 4 | 0.031 |
| HESS V | 4 | 0.031 |
| HERIOT AG | 4 | 0.031 |
| HERBELLA FAM | 4 | 0.031 |
| HERBELLA FA | 4 | 0.031 |
| HENNEQUIN C | 4 | 0.031 |
| HEEG S | 4 | 0.031 |
| HEBERT JR | 4 | 0.031 |
| HE ZH | 4 | 0.031 |
| HE YL | 4 | 0.031 |
| HE XF | 4 | 0.031 |
| HE T | 4 | 0.031 |
| HE M | 4 | 0.031 |
| HE LJ | 4 | 0.031 |
| HE H | 4 | 0.031 |
| HE D | 4 | 0.031 |
| HE B | 4 | 0.031 |
| HAZAMA S | 4 | 0.031 |
| HAYDEN JD | 4 | 0.031 |
| HAYASHI R | 4 | 0.031 |
| HAYASHI M | 4 | 0.031 |
| HAYANO K | 4 | 0.031 |
| HAYAKAWA Y | 4 | 0.031 |
| HAYAKAWA K | 4 | 0.031 |
| HAVARD T | 4 | 0.031 |
| HAUSCHKE D | 4 | 0.031 |
| HATTORI S | 4 | 0.031 |
| HATOGAI K | 4 | 0.031 |
| HATA A | 4 | 0.031 |
| HASEGAWA T | 4 | 0.031 |
| HARVEY JA | 4 | 0.031 |
| HARRISON M | 4 | 0.031 |
| HARPOLE DH | 4 | 0.031 |
| HARDIE LJ | 4 | 0.031 |
| HANNA A | 4 | 0.031 |
| HANDORF E | 4 | 0.031 |
| HANARI N | 4 | 0.031 |
| HANADA T | 4 | 0.031 |
| HAN YL | 4 | 0.031 |
| HAN SY | 4 | 0.031 |
| HAN RQ | 4 | 0.031 |
| HALUSZKA O | 4 | 0.031 |
| HALLMANS G | 4 | 0.031 |
| HALL P | 4 | 0.031 |
| HALKJAER J | 4 | 0.031 |
| HAISA M | 4 | 0.031 |
| HAGHDOOST AA | 4 | 0.031 |
| HACKER M | 4 | 0.031 |
| HABERKORN U | 4 | 0.031 |
| GUTSCHOW CA | 4 | 0.031 |
| GUSELLA M | 4 | 0.031 |
| GURSKI RR | 4 | 0.031 |
| GUPTA P | 4 | 0.031 |
| GUO ZJ | 4 | 0.031 |
| GUO P | 4 | 0.031 |
| GUO M | 4 | 0.031 |
| GUO HZ | 4 | 0.031 |
| GU XP | 4 | 0.031 |
| GRUGAN KD | 4 | 0.031 |
| GROMAN A | 4 | 0.031 |
| GRIONI S | 4 | 0.031 |
| GREWAL A | 4 | 0.031 |
| GREENSTEIN AJ | 4 | 0.031 |
| GREENE CL | 4 | 0.031 |
| GRAVALOS C | 4 | 0.031 |
| GOTO Y | 4 | 0.031 |
| GOTO T | 4 | 0.031 |
| GORDON SR | 4 | 0.031 |
| GOPAL DV | 4 | 0.031 |
| GOODING WE | 4 | 0.031 |
| GONG YY | 4 | 0.031 |
| GONG J | 4 | 0.031 |
| GONG HY | 4 | 0.031 |
| GONG H | 4 | 0.031 |
| GOMI K | 4 | 0.031 |
| GOMEZ-MARTIN C | 4 | 0.031 |
| GOLDIN R | 4 | 0.031 |
| GIOVANNINI M | 4 | 0.031 |
| GILL P | 4 | 0.031 |
| GHIRINGHELLI F | 4 | 0.031 |
| GENERALI D | 4 | 0.031 |
| GEISINGER KR | 4 | 0.031 |
| GE ZJ | 4 | 0.031 |
| GE XL | 4 | 0.031 |
| GE MH | 4 | 0.031 |
| GE D | 4 | 0.031 |
| GAYET B | 4 | 0.031 |
| GATTER KM | 4 | 0.031 |
| GASTINGER I | 4 | 0.031 |
| GARRETT-MAYER E | 4 | 0.031 |
| GARCIA-CARBONERO R | 4 | 0.031 |
| GAO YS | 4 | 0.031 |
| GAO HJ | 4 | 0.031 |
| GAMBARDELLA V | 4 | 0.031 |
| GALUPPO S | 4 | 0.031 |
| FUWA N | 4 | 0.031 |
| FURUTANI K | 4 | 0.031 |
| FURUHATA T | 4 | 0.031 |
| FUQUA L | 4 | 0.031 |
| FULP WJ | 4 | 0.031 |
| FUKUNAGA T | 4 | 0.031 |
| FUKUNAGA S | 4 | 0.031 |
| FUKUDA S | 4 | 0.031 |
| FUKINO N | 4 | 0.031 |
| FUKAYAMA M | 4 | 0.031 |
| FUKAMI N | 4 | 0.031 |
| FUJIWARA J | 4 | 0.031 |
| FUJISHIMA F | 4 | 0.031 |
| FUJIMOTO K | 4 | 0.031 |
| FUJI H | 4 | 0.031 |
| FRIEDRICH J | 4 | 0.031 |
| FRICK TJ | 4 | 0.031 |
| FREISLING H | 4 | 0.031 |
| FRAGA ECD | 4 | 0.031 |
| FOXWELL TJ | 4 | 0.031 |
| FLETT BC | 4 | 0.031 |
| FISCHMAN AJ | 4 | 0.031 |
| FISCHBACH W | 4 | 0.031 |
| FIELDS RC | 4 | 0.031 |
| FICHTER CD | 4 | 0.031 |
| FERRY D | 4 | 0.031 |
| FERREIRA MA | 4 | 0.031 |
| FERRARONI M | 4 | 0.031 |
| FERLAY J | 4 | 0.031 |
| FERGUSON M | 4 | 0.031 |
| FARJAH F | 4 | 0.031 |
| FARIED LS | 4 | 0.031 |
| FANTI S | 4 | 0.031 |
| FANG M | 4 | 0.031 |
| FANG JY | 4 | 0.031 |
| FANG FM | 4 | 0.031 |
| FANG DC | 4 | 0.031 |
| FANELLI RD | 4 | 0.031 |
| FAN ZM | 4 | 0.031 |
| FAN YH | 4 | 0.031 |
| FAN Y | 4 | 0.031 |
| FAN XM | 4 | 0.031 |
| FALUYI OO | 4 | 0.031 |
| FALK SJ | 4 | 0.031 |
| FALK S | 4 | 0.031 |
| FAHIMI S | 4 | 0.031 |
| FAGHERAZZI G | 4 | 0.031 |
| FABOZZI A | 4 | 0.031 |
| EVANS NR | 4 | 0.031 |
| ETIENNE PL | 4 | 0.031 |
| ESTRELLA JS | 4 | 0.031 |
| ESAKI M | 4 | 0.031 |
| ERICSON U | 4 | 0.031 |
| ENESTVEDT CK | 4 | 0.031 |
| EMIG M | 4 | 0.031 |
| EMI Y | 4 | 0.031 |
| ELSTON R | 4 | 0.031 |
| ELLIS S | 4 | 0.031 |
| ELICEIRI B | 4 | 0.031 |
| EL-KHOUEIRY R | 4 | 0.031 |
| EISENBERGER CF | 4 | 0.031 |
| EIKMAN E | 4 | 0.031 |
| EIJKEMANS MJC | 4 | 0.031 |
| EGGER M | 4 | 0.031 |
| EGASHIRA H | 4 | 0.031 |
| DZOBO K | 4 | 0.031 |
| DZIEGIELEWSKI P | 4 | 0.031 |
| DWIVEDI PD | 4 | 0.031 |
| DUTTA S | 4 | 0.031 |
| DUONG CP | 4 | 0.031 |
| DUNBAR KB | 4 | 0.031 |
| DUAN XF | 4 | 0.031 |
| DU LC | 4 | 0.031 |
| DU HZ | 4 | 0.031 |
| DRENTH JPH | 4 | 0.031 |
| DOUCET L | 4 | 0.031 |
| DORES GM | 4 | 0.031 |
| DONG ZW | 4 | 0.031 |
| DONG S | 4 | 0.031 |
| DONG JG | 4 | 0.031 |
| DONEHOWER RC | 4 | 0.031 |
| DOMSCHKE W | 4 | 0.031 |
| DOMINITZ JA | 4 | 0.031 |
| DOMINELLO MM | 4 | 0.031 |
| DODDOLI C | 4 | 0.031 |
| DIVINO CM | 4 | 0.031 |
| DING WL | 4 | 0.031 |
| DIKKEN JL | 4 | 0.031 |
| DIENES HP | 4 | 0.031 |
| DIASIO RB | 4 | 0.031 |
| DI LAURO L | 4 | 0.031 |
| DI BARTOLOMEO M | 4 | 0.031 |
| DEMPSTER M | 4 | 0.031 |
| DEMMY TL | 4 | 0.031 |
| DEMEESTER S | 4 | 0.031 |
| DELLON ES | 4 | 0.031 |
| DECARLI A | 4 | 0.031 |
| DECAESTECKER J | 4 | 0.031 |
| DE MEULENAER B | 4 | 0.031 |
| DE LEYN P | 4 | 0.031 |
| DE CEGLIE A | 4 | 0.031 |
| DAVYDOV M | 4 | 0.031 |
| DAVIS CS | 4 | 0.031 |
| DAVIES L | 4 | 0.031 |
| DAVIES A | 4 | 0.031 |
| DAUM S | 4 | 0.031 |
| DASTER S | 4 | 0.031 |
| DAS BC | 4 | 0.031 |
| DANENBERG PV | 4 | 0.031 |
| DANENBERG KD | 4 | 0.031 |
| DAI W | 4 | 0.031 |
| DAI SB | 4 | 0.031 |
| CURVERS WL | 4 | 0.031 |
| CURTIS RE | 4 | 0.031 |
| CURADO MP | 4 | 0.031 |
| CUI Z | 4 | 0.031 |
| CUI L | 4 | 0.031 |
| CUI H | 4 | 0.031 |
| CUI GH | 4 | 0.031 |
| CSEKE L | 4 | 0.031 |
| COX MR | 4 | 0.031 |
| COURT LE | 4 | 0.031 |
| COSTA-MAIA J | 4 | 0.031 |
| CORSO G | 4 | 0.031 |
| CORREA B | 4 | 0.031 |
| CONWAY DI | 4 | 0.031 |
| CLAXTON Z | 4 | 0.031 |
| CLARK PI | 4 | 0.031 |
| CLARK JW | 4 | 0.031 |
| CLARK GWB | 4 | 0.031 |
| CINCIBUCH J | 4 | 0.031 |
| CIHORIC N | 4 | 0.031 |
| CHUONG M | 4 | 0.031 |
| CHUNG WK | 4 | 0.031 |
| CHUNG JB | 4 | 0.031 |
| CHUANG CY | 4 | 0.031 |
| CHRISTIANI D | 4 | 0.031 |
| CHOW WB | 4 | 0.031 |
| CHOW SC | 4 | 0.031 |
| CHOI SH | 4 | 0.031 |
| CHO C | 4 | 0.031 |
| CHO BC | 4 | 0.031 |
| CHIU R | 4 | 0.031 |
| CHIU CF | 4 | 0.031 |
| CHIRLAQUE MD | 4 | 0.031 |
| CHILUKURI S | 4 | 0.031 |
| CHI CW | 4 | 0.031 |
| CHEON YK | 4 | 0.031 |
| CHENG MF | 4 | 0.031 |
| CHENG JD | 4 | 0.031 |
| CHENG JC | 4 | 0.031 |
| CHENARD MP | 4 | 0.031 |
| CHEN YM | 4 | 0.031 |
| CHEN YH | 4 | 0.031 |
| CHEN WS | 4 | 0.031 |
| CHEN TJ | 4 | 0.031 |
| CHEN TC | 4 | 0.031 |
| CHEN SZ | 4 | 0.031 |
| CHEN SM | 4 | 0.031 |
| CHEN SB | 4 | 0.031 |
| CHEN PC | 4 | 0.031 |
| CHEN MW | 4 | 0.031 |
| CHEN LB | 4 | 0.031 |
| CHEN JW | 4 | 0.031 |
| CHEN HS | 4 | 0.031 |
| CHEN HN | 4 | 0.031 |
| CHEN GY | 4 | 0.031 |
| CHEN GQ | 4 | 0.031 |
| CHEN F | 4 | 0.031 |
| CHEN CL | 4 | 0.031 |
| CHEN CG | 4 | 0.031 |
| CHE Y | 4 | 0.031 |
| CHAYAMA K | 4 | 0.031 |
| CHANG YC | 4 | 0.031 |
| CHANG WL | 4 | 0.031 |
| CHANG SN | 4 | 0.031 |
| CHANG DK | 4 | 0.031 |
| CHAN YP | 4 | 0.031 |
| CHAN KT | 4 | 0.031 |
| CHAN E | 4 | 0.031 |
| CHAN CH | 4 | 0.031 |
| CHAKRAVARTHY B | 4 | 0.031 |
| CHAI Y | 4 | 0.031 |
| CAYGILL CPJ | 4 | 0.031 |
| CATALDEGIRMEN G | 4 | 0.031 |
| CATALANO PJ | 4 | 0.031 |
| CASTELLSAGUE X | 4 | 0.031 |
| CASARETTI R | 4 | 0.031 |
| CANTO MI | 4 | 0.031 |
| CANOVA C | 4 | 0.031 |
| CALLISTER MD | 4 | 0.031 |
| CALIFANO JA | 4 | 0.031 |
| CAI XW | 4 | 0.031 |
| CAI XL | 4 | 0.031 |
| CAI Q | 4 | 0.031 |
| BYRNE PJ | 4 | 0.031 |
| BYRNE JP | 4 | 0.031 |
| BUTTAR N | 4 | 0.031 |
| BUSCAGLIA JM | 4 | 0.031 |
| BURTNESS BA | 4 | 0.031 |
| BURRIS HA | 4 | 0.031 |
| BURMEISTER BH | 4 | 0.031 |
| BURGERHOF J | 4 | 0.031 |
| BUHLER H | 4 | 0.031 |
| BRUNS CJ | 4 | 0.031 |
| BRUNS C | 4 | 0.031 |
| BROWN RE | 4 | 0.031 |
| BROOKS-WILSON A | 4 | 0.031 |
| BRIERLEY J | 4 | 0.031 |
| BRADLEY J | 4 | 0.031 |
| BRADBURY PA | 4 | 0.031 |
| BOZZETTI F | 4 | 0.031 |
| BOWREY DJ | 4 | 0.031 |
| BOWER M | 4 | 0.031 |
| BOUVET M | 4 | 0.031 |
| BOUGRINI M | 4 | 0.031 |
| BOUCHER E | 4 | 0.031 |
| BORRAS JM | 4 | 0.031 |
| BORNER M | 4 | 0.031 |
| BONENKAMP JJ | 4 | 0.031 |
| BOLT TA | 4 | 0.031 |
| BOGATYREVA L | 4 | 0.031 |
| BOBRYSHEV YV | 4 | 0.031 |
| BLOUNT PL | 4 | 0.031 |
| BLAZEBY J | 4 | 0.031 |
| BLACKSHAW G | 4 | 0.031 |
| BLACKHAM AU | 4 | 0.031 |
| BJELOVIC M | 4 | 0.031 |
| BIRKMEYER JD | 4 | 0.031 |
| BILEN Y | 4 | 0.031 |
| BICHEV D | 4 | 0.031 |
| BHATT A | 4 | 0.031 |
| BHATIA S | 4 | 0.031 |
| BERGQUIST H | 4 | 0.031 |
| BENNETT C | 4 | 0.031 |
| BENETOU V | 4 | 0.031 |
| BEN ABDELGHANI M | 4 | 0.031 |
| BEHRENS A | 4 | 0.031 |
| BASHASH M | 4 | 0.031 |
| BARTENSTEIN P | 4 | 0.031 |
| BARHAM CP | 4 | 0.031 |
| BAMEZAI RNK | 4 | 0.031 |
| BAMBA T | 4 | 0.031 |
| BALDUS S | 4 | 0.031 |
| BAKER J | 4 | 0.031 |
| BAINS M | 4 | 0.031 |
| BAILEY IS | 4 | 0.031 |
| BAI YX | 4 | 0.031 |
| BAI G | 4 | 0.031 |
| BAHRA M | 4 | 0.031 |
| BAE WK | 4 | 0.031 |
| BACKEMAR L | 4 | 0.031 |
| BACHMANN P | 4 | 0.031 |
| BACHMANN K | 4 | 0.031 |
| BABA S | 4 | 0.031 |
| AYE RW | 4 | 0.031 |
| AWIKA JM | 4 | 0.031 |
| AVISAR E | 4 | 0.031 |
| AURILIO G | 4 | 0.031 |
| AUNE D | 4 | 0.031 |
| ATLANI D | 4 | 0.031 |
| ASTSATUROV IA | 4 | 0.031 |
| ASCIOTI AJ | 4 | 0.031 |
| ASAKURA H | 4 | 0.031 |
| ARITA T | 4 | 0.031 |
| ARDANAZ E | 4 | 0.031 |
| ARANHA GV | 4 | 0.031 |
| ARAI A | 4 | 0.031 |
| APRILE G | 4 | 0.031 |
| APPELMAN HD | 4 | 0.031 |
| APARICIO T | 4 | 0.031 |
| ANWER J | 4 | 0.031 |
| ANBAI A | 4 | 0.031 |
| AN JY | 4 | 0.031 |
| ALSOP BR | 4 | 0.031 |
| ALSINA M | 4 | 0.031 |
| ALOIA TA | 4 | 0.031 |
| ALNAJI RM | 4 | 0.031 |
| ALIZADEH AM | 4 | 0.031 |
| ALAVI A | 4 | 0.031 |
| AKITA H | 4 | 0.031 |
| AIZAWA M | 4 | 0.031 |
| AIYER HS | 4 | 0.031 |
| AIKO S | 4 | 0.031 |
| AHUJA YR | 4 | 0.031 |
| AHUJA N | 4 | 0.031 |
| AHN MJ | 4 | 0.031 |
| ADES S | 4 | 0.031 |
| ADAM G | 4 | 0.031 |
| ACKERMANN C | 4 | 0.031 |
| ABE S | 4 | 0.031 |
| ABE M | 4 | 0.031 |
| ABE E | 4 | 0.031 |
| ABDEL-LATIF MMM | 4 | 0.031 |
| ABBASZADEGAN MR | 4 | 0.031 |
| ABBAS AE | 4 | 0.031 |
| ZWINDERMAN AH | 3 | 0.023 |
| ZUR HAUSEN A | 3 | 0.023 |
| ZUO Y | 3 | 0.023 |
| ZOU X | 3 | 0.023 |
| ZOU ST | 3 | 0.023 |
| ZOU L | 3 | 0.023 |
| ZITVOGEL L | 3 | 0.023 |
| ZIMMITTI G | 3 | 0.023 |
| ZHU ZH | 3 | 0.023 |
| ZHU Z | 3 | 0.023 |
| ZHU YG | 3 | 0.023 |
| ZHU XZ | 3 | 0.023 |
| ZHU WY | 3 | 0.023 |
| ZHU TN | 3 | 0.023 |
| ZHU S | 3 | 0.023 |
| ZHU LJ | 3 | 0.023 |
| ZHU JB | 3 | 0.023 |
| ZHU DY | 3 | 0.023 |
| ZHU C | 3 | 0.023 |
| ZHU AX | 3 | 0.023 |
| ZHOU ZY | 3 | 0.023 |
| ZHOU ZM | 3 | 0.023 |
| ZHOU YQ | 3 | 0.023 |
| ZHOU YL | 3 | 0.023 |
| ZHOU YA | 3 | 0.023 |
| ZHOU MZ | 3 | 0.023 |
| ZHOU K | 3 | 0.023 |
| ZHOU FX | 3 | 0.023 |
| ZHOU DM | 3 | 0.023 |
| ZHOU C | 3 | 0.023 |
| ZHONG YS | 3 | 0.023 |
| ZHONG XJ | 3 | 0.023 |
| ZHONG L | 3 | 0.023 |
| ZHONG CX | 3 | 0.023 |
| ZHONG CJ | 3 | 0.023 |
| ZHENG XY | 3 | 0.023 |
| ZHENG TL | 3 | 0.023 |
| ZHENG PY | 3 | 0.023 |
| ZHENG M | 3 | 0.023 |
| ZHENG K | 3 | 0.023 |
| ZHENG JY | 3 | 0.023 |
| ZHENG H | 3 | 0.023 |
| ZHEN YS | 3 | 0.023 |
| ZHAO ZZ | 3 | 0.023 |
| ZHAO ZY | 3 | 0.023 |
| ZHAO ZH | 3 | 0.023 |
| ZHAO YN | 3 | 0.023 |
| ZHAO YJ | 3 | 0.023 |
| ZHAO YH | 3 | 0.023 |
| ZHAO YG | 3 | 0.023 |
| ZHAO XL | 3 | 0.023 |
| ZHAO XJ | 3 | 0.023 |
| ZHAO M | 3 | 0.023 |
| ZHAO LY | 3 | 0.023 |
| ZHAO LN | 3 | 0.023 |
| ZHAO LL | 3 | 0.023 |
| ZHAO JS | 3 | 0.023 |
| ZHAO EJ | 3 | 0.023 |
| ZHAO CL | 3 | 0.023 |
| ZHAO C | 3 | 0.023 |
| ZHANG WZ | 3 | 0.023 |
| ZHANG WY | 3 | 0.023 |
| ZHANG WX | 3 | 0.023 |
| ZHANG SP | 3 | 0.023 |
| ZHANG SL | 3 | 0.023 |
| ZHANG SJ | 3 | 0.023 |
| ZHANG SH | 3 | 0.023 |
| ZHANG QQ | 3 | 0.023 |
| ZHANG PJ | 3 | 0.023 |
| ZHANG MH | 3 | 0.023 |
| ZHANG LX | 3 | 0.023 |
| ZHANG HT | 3 | 0.023 |
| ZHANG HS | 3 | 0.023 |
| ZHANG HD | 3 | 0.023 |
| ZHANG HB | 3 | 0.023 |
| ZHANG GJ | 3 | 0.023 |
| ZHANG GD | 3 | 0.023 |
| ZHANG FM | 3 | 0.023 |
| ZHANG FJ | 3 | 0.023 |
| ZHANG FG | 3 | 0.023 |
| ZHANG CP | 3 | 0.023 |
| ZHAI R | 3 | 0.023 |
| ZFASS AM | 3 | 0.023 |
| ZERBINI LF | 3 | 0.023 |
| ZERBIB F | 3 | 0.023 |
| ZENG XT | 3 | 0.023 |
| ZENG TT | 3 | 0.023 |
| ZENG L | 3 | 0.023 |
| ZEHENTMAYR F | 3 | 0.023 |
| ZAVOS C | 3 | 0.023 |
| ZAORSKY NG | 3 | 0.023 |
| ZANG Q | 3 | 0.023 |
| ZANG L | 3 | 0.023 |
| ZAMORA-ROS R | 3 | 0.023 |
| ZAKI MA | 3 | 0.023 |
| ZAITSU Y | 3 | 0.023 |
| YUNO A | 3 | 0.023 |
| YUKI S | 3 | 0.023 |
| YUKAWA Y | 3 | 0.023 |
| YUE BL | 3 | 0.023 |
| YUAN F | 3 | 0.023 |
| YUAN DW | 3 | 0.023 |
| YUAN DM | 3 | 0.023 |
| YU ZL | 3 | 0.023 |
| YU YC | 3 | 0.023 |
| YU XF | 3 | 0.023 |
| YU WF | 3 | 0.023 |
| YU SY | 3 | 0.023 |
| YU MG | 3 | 0.023 |
| YU ITS | 3 | 0.023 |
| YU HM | 3 | 0.023 |
| YU F | 3 | 0.023 |
| YU DH | 3 | 0.023 |
| YU D | 3 | 0.023 |
| YOUNUS J | 3 | 0.023 |
| YOUNG J | 3 | 0.023 |
| YOU YJ | 3 | 0.023 |
| YOU B | 3 | 0.023 |
| YOSHIOKA T | 3 | 0.023 |
| YOSHIOKA H | 3 | 0.023 |
| YOSHIOKA A | 3 | 0.023 |
| YOSHIMURA F | 3 | 0.023 |
| YOSHIHARA M | 3 | 0.023 |
| YOSHIDA A | 3 | 0.023 |
| YOON SS | 3 | 0.023 |
| YOON MS | 3 | 0.023 |
| YOON HJ | 3 | 0.023 |
| YOON H | 3 | 0.023 |
| YONEZAWA M | 3 | 0.023 |
| YONEKURA R | 3 | 0.023 |
| YOKOZAKI H | 3 | 0.023 |
| YING LS | 3 | 0.023 |
| YIN W | 3 | 0.023 |
| YIN N | 3 | 0.023 |
| YIN HY | 3 | 0.023 |
| YIN D | 3 | 0.023 |
| YIN BL | 3 | 0.023 |
| YILMAZ M | 3 | 0.023 |
| YENDAMURI SS | 3 | 0.023 |
| YEN R | 3 | 0.023 |
| YEN CC | 3 | 0.023 |
| YEH KY | 3 | 0.023 |
| YEH KT | 3 | 0.023 |
| YEH CH | 3 | 0.023 |
| YE WG | 3 | 0.023 |
| YE W | 3 | 0.023 |
| YE S | 3 | 0.023 |
| YE Q | 3 | 0.023 |
| YAZDANBOD M | 3 | 0.023 |
| YAYLIM I | 3 | 0.023 |
| YASUDA K | 3 | 0.023 |
| YASUDA A | 3 | 0.023 |
| YAREMKO B | 3 | 0.023 |
| YAO K | 3 | 0.023 |
| YAO F | 3 | 0.023 |
| YANTISS RK | 3 | 0.023 |
| YANG ZZ | 3 | 0.023 |
| YANG ZY | 3 | 0.023 |
| YANG YZ | 3 | 0.023 |
| YANG YQ | 3 | 0.023 |
| YANG YF | 3 | 0.023 |
| YANG XP | 3 | 0.023 |
| YANG XM | 3 | 0.023 |
| YANG XB | 3 | 0.023 |
| YANG VW | 3 | 0.023 |
| YANG SN | 3 | 0.023 |
| YANG QY | 3 | 0.023 |
| YANG MJ | 3 | 0.023 |
| YANG LX | 3 | 0.023 |
| YANG JS | 3 | 0.023 |
| YANG JB | 3 | 0.023 |
| YANG HJ | 3 | 0.023 |
| YANG GT | 3 | 0.023 |
| YANG FY | 3 | 0.023 |
| YANG D | 3 | 0.023 |
| YANG CQ | 3 | 0.023 |
| YANG CJ | 3 | 0.023 |
| YANG CC | 3 | 0.023 |
| YANAGISAWA A | 3 | 0.023 |
| YANAGIMOTO Y | 3 | 0.023 |
| YANAGAWA T | 3 | 0.023 |
| YANAGAWA N | 3 | 0.023 |
| YAN YX | 3 | 0.023 |
| YAN WP | 3 | 0.023 |
| YAN L | 3 | 0.023 |
| YAN CH | 3 | 0.023 |
| YAMAZAKI Y | 3 | 0.023 |
| YAMASAKI Y | 3 | 0.023 |
| YAMANAKA T | 3 | 0.023 |
| YAMAGUCHI H | 3 | 0.023 |
| YAMABUKI T | 3 | 0.023 |
| YALA S | 3 | 0.023 |
| YAKABI K | 3 | 0.023 |
| YAHAGI N | 3 | 0.023 |
| YAGUCHI Y | 3 | 0.023 |
| YADAV DS | 3 | 0.023 |
| YACOUB WN | 3 | 0.023 |
| XUE S | 3 | 0.023 |
| XU YH | 3 | 0.023 |
| XU T | 3 | 0.023 |
| XU SD | 3 | 0.023 |
| XU K | 3 | 0.023 |
| XU HW | 3 | 0.023 |
| XU GH | 3 | 0.023 |
| XU DG | 3 | 0.023 |
| XU D | 3 | 0.023 |
| XU CS | 3 | 0.023 |
| XIONG Y | 3 | 0.023 |
| XING S | 3 | 0.023 |
| XING L | 3 | 0.023 |
| XING JL | 3 | 0.023 |
| XIN ZL | 3 | 0.023 |
| XIN H | 3 | 0.023 |
| XIE XY | 3 | 0.023 |
| XIE TP | 3 | 0.023 |
| XIE R | 3 | 0.023 |
| XIE L | 3 | 0.023 |
| XIE FJ | 3 | 0.023 |
| XIAO YP | 3 | 0.023 |
| XIAO XQ | 3 | 0.023 |
| XIAO SY | 3 | 0.023 |
| XIAO Q | 3 | 0.023 |
| XIAO P | 3 | 0.023 |
| XIAO HJ | 3 | 0.023 |
| XIAO H | 3 | 0.023 |
| XIANG YB | 3 | 0.023 |
| XIAN L | 3 | 0.023 |
| XIA X | 3 | 0.023 |
| XIA Q | 3 | 0.023 |
| XIA JH | 3 | 0.023 |
| XIA JC | 3 | 0.023 |
| XIA J | 3 | 0.023 |
| XIA HF | 3 | 0.023 |
| WUNSCH V | 3 | 0.023 |
| WU YM | 3 | 0.023 |
| WU XP | 3 | 0.023 |
| WU XL | 3 | 0.023 |
| WU XH | 3 | 0.023 |
| WU TH | 3 | 0.023 |
| WU T | 3 | 0.023 |
| WU R | 3 | 0.023 |
| WU PC | 3 | 0.023 |
| WU P | 3 | 0.023 |
| WU LY | 3 | 0.023 |
| WU JK | 3 | 0.023 |
| WU JB | 3 | 0.023 |
| WU HQ | 3 | 0.023 |
| WU GX | 3 | 0.023 |
| WU D | 3 | 0.023 |
| WRIGHT JJ | 3 | 0.023 |
| WRIGHT E | 3 | 0.023 |
| WOTHERSPOON A | 3 | 0.023 |
| WONG YC | 3 | 0.023 |
| WONG VCL | 3 | 0.023 |
| WONG RKH | 3 | 0.023 |
| WONG LF | 3 | 0.023 |
| WONG GS | 3 | 0.023 |
| WOLF M | 3 | 0.023 |
| WOLF B | 3 | 0.023 |
| WINTER R | 3 | 0.023 |
| WINTER KA | 3 | 0.023 |
| WILMINK JW | 3 | 0.023 |
| WILLS L | 3 | 0.023 |
| WILLIS JE | 3 | 0.023 |
| WILLIAMS N | 3 | 0.023 |
| WILLER B | 3 | 0.023 |
| WILHELM D | 3 | 0.023 |
| WIKTOR AE | 3 | 0.023 |
| WIJNHOVEN BP | 3 | 0.023 |
| WIIK H | 3 | 0.023 |
| WIDMER L | 3 | 0.023 |
| WHITE GE | 3 | 0.023 |
| WHIBLEY CE | 3 | 0.023 |
| WHEELER W | 3 | 0.023 |
| WEYANT MJ | 3 | 0.023 |
| WEX T | 3 | 0.023 |
| WEST CM | 3 | 0.023 |
| WERNISCH L | 3 | 0.023 |
| WEN YY | 3 | 0.023 |
| WEN JF | 3 | 0.023 |
| WEISS C | 3 | 0.023 |
| WEINSTEIN SJ | 3 | 0.023 |
| WEIN A | 3 | 0.023 |
| WEIGEL TL | 3 | 0.023 |
| WEI YM | 3 | 0.023 |
| WEI LZ | 3 | 0.023 |
| WEI KL | 3 | 0.023 |
| WEI JX | 3 | 0.023 |
| WEI JS | 3 | 0.023 |
| WEE JO | 3 | 0.023 |
| WEATHERS RE | 3 | 0.023 |
| WAUGH E | 3 | 0.023 |
| WATANABE H | 3 | 0.023 |
| WANI SB | 3 | 0.023 |
| WANG ZZ | 3 | 0.023 |
| WANG ZJ | 3 | 0.023 |
| WANG YS | 3 | 0.023 |
| WANG YN | 3 | 0.023 |
| WANG YK | 3 | 0.023 |
| WANG YG | 3 | 0.023 |
| WANG XT | 3 | 0.023 |
| WANG WX | 3 | 0.023 |
| WANG WQ | 3 | 0.023 |
| WANG WC | 3 | 0.023 |
| WANG TD | 3 | 0.023 |
| WANG SW | 3 | 0.023 |
| WANG SL | 3 | 0.023 |
| WANG SH | 3 | 0.023 |
| WANG QX | 3 | 0.023 |
| WANG QH | 3 | 0.023 |
| WANG MS | 3 | 0.023 |
| WANG LY | 3 | 0.023 |
| WANG LB | 3 | 0.023 |
| WANG KN | 3 | 0.023 |
| WANG JP | 3 | 0.023 |
| WANG JG | 3 | 0.023 |
| WANG JA | 3 | 0.023 |
| WANG HX | 3 | 0.023 |
| WANG HQ | 3 | 0.023 |
| WANG GY | 3 | 0.023 |
| WANG GM | 3 | 0.023 |
| WANG GD | 3 | 0.023 |
| WANG GC | 3 | 0.023 |
| WANG GB | 3 | 0.023 |
| WANG DY | 3 | 0.023 |
| WANG DH | 3 | 0.023 |
| WANG DF | 3 | 0.023 |
| WANG CS | 3 | 0.023 |
| WANG CG | 3 | 0.023 |
| WAN SG | 3 | 0.023 |
| WAN JY | 3 | 0.023 |
| WALPOLE ET | 3 | 0.023 |
| WALL C | 3 | 0.023 |
| WALDRON TJ | 3 | 0.023 |
| WAKUI R | 3 | 0.023 |
| WAKATSUKI T | 3 | 0.023 |
| WAKASUGI T | 3 | 0.023 |
| WAKAI K | 3 | 0.023 |
| WAKABAYASHI G | 3 | 0.023 |
| WAGNER AD | 3 | 0.023 |
| WAGENIUS G | 3 | 0.023 |
| WADASAKI K | 3 | 0.023 |
| VOS P | 3 | 0.023 |
| VOEST EE | 3 | 0.023 |
| VISSERS KJ | 3 | 0.023 |
| VISSER M | 3 | 0.023 |
| VISMER HF | 3 | 0.023 |
| VISCONTI A | 3 | 0.023 |
| VIOQUE J | 3 | 0.023 |
| VINCENZI B | 3 | 0.023 |
| VINCENT M | 3 | 0.023 |
| VILLANACCI V | 3 | 0.023 |
| VERMA Y | 3 | 0.023 |
| VERMA A | 3 | 0.023 |
| VERHOEVEN RHA | 3 | 0.023 |
| VERGNAUD AC | 3 | 0.023 |
| VERECZKEI A | 3 | 0.023 |
| VENKAT PS | 3 | 0.023 |
| VELENIK V | 3 | 0.023 |
| VELANOVICH V | 3 | 0.023 |
| VEGA-VILLEGAS ME | 3 | 0.023 |
| VEGA KJ | 3 | 0.023 |
| VEDANTHAN R | 3 | 0.023 |
| VAY C | 3 | 0.023 |
| VASHIST Y | 3 | 0.023 |
| VASEI M | 3 | 0.023 |
| VARIN O | 3 | 0.023 |
| VARDI I | 3 | 0.023 |
| VARADARAJULU S | 3 | 0.023 |
| VAPORCIYAN A | 3 | 0.023 |
| VANHUYSE M | 3 | 0.023 |
| VANETTI E | 3 | 0.023 |
| VAN SEUNINGEN I | 3 | 0.023 |
| VAN RIJ CM | 3 | 0.023 |
| VAN OIJEN MG | 3 | 0.023 |
| VAN NISTELROOIJ AMJ | 3 | 0.023 |
| VAN LIER ALHMW | 3 | 0.023 |
| VAN LEEUWEN FE | 3 | 0.023 |
| VAN LAARHOVEN H | 3 | 0.023 |
| VAN GRIEKEN N | 3 | 0.023 |
| VAN EIJCK CHJ | 3 | 0.023 |
| VAN DER SANGEN MJC | 3 | 0.023 |
| VAN DE VOORDE L | 3 | 0.023 |
| VAN DAM G | 3 | 0.023 |
| VAN DAELE E | 3 | 0.023 |
| VAN COEVORDEN F | 3 | 0.023 |
| VAN CAMP J | 3 | 0.023 |
| VAILLANT JC | 3 | 0.023 |
| VACKOVA Z | 3 | 0.023 |
| VACCHELLI E | 3 | 0.023 |
| USNARSKA-ZUBKIEWICZ L | 3 | 0.023 |
| USMAN N | 3 | 0.023 |
| USHIJIMA T | 3 | 0.023 |
| URAYAMA S | 3 | 0.023 |
| UNER A | 3 | 0.023 |
| ULM K | 3 | 0.023 |
| UJIKI MB | 3 | 0.023 |
| UI T | 3 | 0.023 |
| UHL W | 3 | 0.023 |
| UEDA K | 3 | 0.023 |
| UEDA A | 3 | 0.023 |
| UCHIYAMA A | 3 | 0.023 |
| UCHIDA T | 3 | 0.023 |
| UCHIDA S | 3 | 0.023 |
| UCAR DA | 3 | 0.023 |
| TZENG CWD | 3 | 0.023 |
| TZEN KY | 3 | 0.023 |
| TWINE C | 3 | 0.023 |
| TURNER K | 3 | 0.023 |
| TURNER DJ | 3 | 0.023 |
| TURKINGTON RC | 3 | 0.023 |
| TURAGA KK | 3 | 0.023 |
| TUERSUN A | 3 | 0.023 |
| TUCKER MA | 3 | 0.023 |
| TUBBS RR | 3 | 0.023 |
| TSUKAMOTO N | 3 | 0.023 |
| TSUJINAKA T | 3 | 0.023 |
| TSUJIMURA T | 3 | 0.023 |
| TSUJII M | 3 | 0.023 |
| TSUJIE H | 3 | 0.023 |
| TSUJI Y | 3 | 0.023 |
| TSUJI S | 3 | 0.023 |
| TSUDA M | 3 | 0.023 |
| TSUCHIYA S | 3 | 0.023 |
| TSUCHIKAWA T | 3 | 0.023 |
| TSUCHIDA K | 3 | 0.023 |
| TSUBURAYA A | 3 | 0.023 |
| TSUBOYAMA T | 3 | 0.023 |
| TSUBOI K | 3 | 0.023 |
| TSOU YK | 3 | 0.023 |
| TSAI YF | 3 | 0.023 |
| TSAI S | 3 | 0.023 |
| TSAI JT | 3 | 0.023 |
| TSAI HT | 3 | 0.023 |
| TSAI CL | 3 | 0.023 |
| TROWBRIDGE R | 3 | 0.023 |
| TRIPATHI M | 3 | 0.023 |
| TRESCH E | 3 | 0.023 |
| TRAVIS LB | 3 | 0.023 |
| TRAMACERE I | 3 | 0.023 |
| TOYOOKA S | 3 | 0.023 |
| TOXOPEUS ELA | 3 | 0.023 |
| TOUGERON D | 3 | 0.023 |
| TORGERSEN Z | 3 | 0.023 |
| TORELLI E | 3 | 0.023 |
| TOPAZIAN M | 3 | 0.023 |
| TONOMOTO Y | 3 | 0.023 |
| TONG YS | 3 | 0.023 |
| TONG T | 3 | 0.023 |
| TONG DK | 3 | 0.023 |
| TOMURA D | 3 | 0.023 |
| TOMORI A | 3 | 0.023 |
| TOMLINSON I | 3 | 0.023 |
| TOMITA H | 3 | 0.023 |
| TOKUNAGA S | 3 | 0.023 |
| TOKUNAGA A | 3 | 0.023 |
| TOKUHISA M | 3 | 0.023 |
| TOKAR JL | 3 | 0.023 |
| TOCHIGI T | 3 | 0.023 |
| TOBA T | 3 | 0.023 |
| TISZLAVICZ L | 3 | 0.023 |
| TIO M | 3 | 0.023 |
| TINMOUTH J | 3 | 0.023 |
| TIMMER PR | 3 | 0.023 |
| TIISEKWA B | 3 | 0.023 |
| TIAN XY | 3 | 0.023 |
| TIAN X | 3 | 0.023 |
| TIAN L | 3 | 0.023 |
| THURAU K | 3 | 0.023 |
| THORNS C | 3 | 0.023 |
| THOMSON DB | 3 | 0.023 |
| THOMAY AA | 3 | 0.023 |
| THOMAS R | 3 | 0.023 |
| THOMAS P | 3 | 0.023 |
| THOMAS A | 3 | 0.023 |
| THIELTGES S | 3 | 0.023 |
| THEUMER MG | 3 | 0.023 |
| THALLINGER CMR | 3 | 0.023 |
| THAKUR B | 3 | 0.023 |
| THAKRAR HV | 3 | 0.023 |
| TETZLAFF ED | 3 | 0.023 |
| TESSIER W | 3 | 0.023 |
| TESSELAAR ME | 3 | 0.023 |
| TERUNUMA T | 3 | 0.023 |
| TERASHIMA K | 3 | 0.023 |
| TEOH EJ | 3 | 0.023 |
| TEBBUTT NC | 3 | 0.023 |
| TATSUMI M | 3 | 0.023 |
| TARLETON HP | 3 | 0.023 |
| TAO YP | 3 | 0.023 |
| TANIZAWA T | 3 | 0.023 |
| TANIKI T | 3 | 0.023 |
| TANIGUCHI M | 3 | 0.023 |
| TANG WY | 3 | 0.023 |
| TANG S | 3 | 0.023 |
| TANG JC | 3 | 0.023 |
| TANG J | 3 | 0.023 |
| TANAKA A | 3 | 0.023 |
| TANABE Y | 3 | 0.023 |
| TANABE K | 3 | 0.023 |
| TAN X | 3 | 0.023 |
| TAN IB | 3 | 0.023 |
| TAN HS | 3 | 0.023 |
| TAN C | 3 | 0.023 |
| TAMURA K | 3 | 0.023 |
| TAMOTSU K | 3 | 0.023 |
| TAMEGAI H | 3 | 0.023 |
| TAMARI K | 3 | 0.023 |
| TAMAI S | 3 | 0.023 |
| TAKIZAWA D | 3 | 0.023 |
| TAKISAWA H | 3 | 0.023 |
| TAKIGUCHI N | 3 | 0.023 |
| TAKIGUCHI G | 3 | 0.023 |
| TAKIFUJI K | 3 | 0.023 |
| TAKEZAKI T | 3 | 0.023 |
| TAKEUCHI T | 3 | 0.023 |
| TAKEUCHI D | 3 | 0.023 |
| TAKEUCHI A | 3 | 0.023 |
| TAKESAKO K | 3 | 0.023 |
| TAKENOUCHI T | 3 | 0.023 |
| TAKEMURA M | 3 | 0.023 |
| TAKEBE N | 3 | 0.023 |
| TAKATA Y | 3 | 0.023 |
| TAKATA O | 3 | 0.023 |
| TAKATA K | 3 | 0.023 |
| TAKASE N | 3 | 0.023 |
| TAKAHASHI I | 3 | 0.023 |
| TAKACHI K | 3 | 0.023 |
| TAI P | 3 | 0.023 |
| TAHARA H | 3 | 0.023 |
| TAGO M | 3 | 0.023 |
| TADA M | 3 | 0.023 |
| TABOLA R | 3 | 0.023 |
| SZENTPALI K | 3 | 0.023 |
| SZEMRAJ J | 3 | 0.023 |
| SWANSON PE | 3 | 0.023 |
| SUZUKI R | 3 | 0.023 |
| SUO ZH | 3 | 0.023 |
| SUNPAWERAVONG S | 3 | 0.023 |
| SUNDAR H | 3 | 0.023 |
| SUN ZY | 3 | 0.023 |
| SUN ZF | 3 | 0.023 |
| SUN YS | 3 | 0.023 |
| SUN XH | 3 | 0.023 |
| SUN XF | 3 | 0.023 |
| SUN SQ | 3 | 0.023 |
| SUN QF | 3 | 0.023 |
| SUN MZ | 3 | 0.023 |
| SUN LM | 3 | 0.023 |
| SUN FY | 3 | 0.023 |
| SUN FH | 3 | 0.023 |
| SUN C | 3 | 0.023 |
| SULKES A | 3 | 0.023 |
| SUKOV WR | 3 | 0.023 |
| SUI H | 3 | 0.023 |
| SUGIURA H | 3 | 0.023 |
| SUGISAWA N | 3 | 0.023 |
| SUGIMOTO R | 3 | 0.023 |
| SUGIMOTO K | 3 | 0.023 |
| SUGIMACHI K | 3 | 0.023 |
| SUGIHARA Y | 3 | 0.023 |
| SUGAWARA A | 3 | 0.023 |
| SUGAI H | 3 | 0.023 |
| SUGAHARA S | 3 | 0.023 |
| SUDARSHAN M | 3 | 0.023 |
| SUBAR AF | 3 | 0.023 |
| SU Z | 3 | 0.023 |
| SU YF | 3 | 0.023 |
| SU S | 3 | 0.023 |
| SU JW | 3 | 0.023 |
| STUERMER L | 3 | 0.023 |
| STUART RC | 3 | 0.023 |
| STRUMBERG D | 3 | 0.023 |
| STRONG S | 3 | 0.023 |
| STROBEL O | 3 | 0.023 |
| STROBEL K | 3 | 0.023 |
| STRICKLAND PT | 3 | 0.023 |
| STRAIF K | 3 | 0.023 |
| STOVALL M | 3 | 0.023 |
| STONER K | 3 | 0.023 |
| STITT L | 3 | 0.023 |
| STEVENS T | 3 | 0.023 |
| STENNING SP | 3 | 0.023 |
| STENLING R | 3 | 0.023 |
| STEINERT R | 3 | 0.023 |
| STEINEMANN G | 3 | 0.023 |
| STEIN H | 3 | 0.023 |
| STEEVENS J | 3 | 0.023 |
| STEBBING J | 3 | 0.023 |
| STAUFFER JA | 3 | 0.023 |
| STARKSCHALL G | 3 | 0.023 |
| STAHL A | 3 | 0.023 |
| SPRANGERS M | 3 | 0.023 |
| SPIGEL DR | 3 | 0.023 |
| SPEZI E | 3 | 0.023 |
| SPECHLER S | 3 | 0.023 |
| SPANEL P | 3 | 0.023 |
| SOTIROPOULOS GC | 3 | 0.023 |
| SONG YM | 3 | 0.023 |
| SONG XR | 3 | 0.023 |
| SONG W | 3 | 0.023 |
| SONG K | 3 | 0.023 |
| SONG J | 3 | 0.023 |
| SONG GQ | 3 | 0.023 |
| SONETT JR | 3 | 0.023 |
| SON S | 3 | 0.023 |
| SOLOMON DH | 3 | 0.023 |
| SOLITO B | 3 | 0.023 |
| SOHEILI ZS | 3 | 0.023 |
| SMYRK TC | 3 | 0.023 |
| SMITH SA | 3 | 0.023 |
| SMITH L | 3 | 0.023 |
| SMITH JK | 3 | 0.023 |
| SMITH IM | 3 | 0.023 |
| SMITH CD | 3 | 0.023 |
| SMITH A | 3 | 0.023 |
| SMAALAND R | 3 | 0.023 |
| SLIMANI N | 3 | 0.023 |
| SITAS F | 3 | 0.023 |
| SINGH RK | 3 | 0.023 |
| SINGH PP | 3 | 0.023 |
| SINGH H | 3 | 0.023 |
| SIMA CS | 3 | 0.023 |
| SILVA IDCG | 3 | 0.023 |
| SIHVO EI | 3 | 0.023 |
| SIERZEGA M | 3 | 0.023 |
| SIENA S | 3 | 0.023 |
| SIDDIQUI UD | 3 | 0.023 |
| SIDDIQI MA | 3 | 0.023 |
| SICK O | 3 | 0.023 |
| SHUENG PW | 3 | 0.023 |
| SHU YQ | 3 | 0.023 |
| SHON IH | 3 | 0.023 |
| SHIVAPPA N | 3 | 0.023 |
| SHIROSHITA T | 3 | 0.023 |
| SHIRATSUCHI H | 3 | 0.023 |
| SHIRATO H | 3 | 0.023 |
| SHIRAKAWA T | 3 | 0.023 |
| SHIOYA M | 3 | 0.023 |
| SHIOTANI A | 3 | 0.023 |
| SHINZAKI S | 3 | 0.023 |
| SHINOTO M | 3 | 0.023 |
| SHINCHI H | 3 | 0.023 |
| SHIN EJ | 3 | 0.023 |
| SHIN CM | 3 | 0.023 |
| SHIMURA T | 3 | 0.023 |
| SHIMOYAMA Y | 3 | 0.023 |
| SHIMOKAWA T | 3 | 0.023 |
| SHIMADA K | 3 | 0.023 |
| SHIM JH | 3 | 0.023 |
| SHIM H | 3 | 0.023 |
| SHIM CS | 3 | 0.023 |
| SHIH CH | 3 | 0.023 |
| SHIGEOKA M | 3 | 0.023 |
| SHIELDS HM | 3 | 0.023 |
| SHIBUYA Y | 3 | 0.023 |
| SHIBUTANI M | 3 | 0.023 |
| SHI SB | 3 | 0.023 |
| SHI MX | 3 | 0.023 |
| SHI GF | 3 | 0.023 |
| SHI F | 3 | 0.023 |
| SHEYHEDIN I | 3 | 0.023 |
| SHEN YZ | 3 | 0.023 |
| SHEN WY | 3 | 0.023 |
| SHEN W | 3 | 0.023 |
| SHEN JH | 3 | 0.023 |
| SHEN HT | 3 | 0.023 |
| SHEN FY | 3 | 0.023 |
| SHEN CY | 3 | 0.023 |
| SHEN CC | 3 | 0.023 |
| SHATERIAN A | 3 | 0.023 |
| SHARMA N | 3 | 0.023 |
| SHARMA JD | 3 | 0.023 |
| SHARMA AK | 3 | 0.023 |
| SHARAFKHAH M | 3 | 0.023 |
| SHAO YJ | 3 | 0.023 |
| SHAO K | 3 | 0.023 |
| SHANNON NB | 3 | 0.023 |
| SHANGINA O | 3 | 0.023 |
| SHANG L | 3 | 0.023 |
| SHAMJI FM | 3 | 0.023 |
| SHAH PC | 3 | 0.023 |
| SHAH N | 3 | 0.023 |
| SHAH M | 3 | 0.023 |
| SEYEDNEZHAD F | 3 | 0.023 |
| SERUCA R | 3 | 0.023 |
| SERGI D | 3 | 0.023 |
| SEPEHR A | 3 | 0.023 |
| SENZER N | 3 | 0.023 |
| SENDO H | 3 | 0.023 |
| SEKIGUCHI K | 3 | 0.023 |
| SEIFERT H | 3 | 0.023 |
| SEIFERT B | 3 | 0.023 |
| SEGUIN C | 3 | 0.023 |
| SEGELOV E | 3 | 0.023 |
| SEELY AJ | 3 | 0.023 |
| SEEHOFER D | 3 | 0.023 |
| SCOTTI L | 3 | 0.023 |
| SCHWARTZ D | 3 | 0.023 |
| SCHWAMEIS K | 3 | 0.023 |
| SCHUMACHER B | 3 | 0.023 |
| SCHROEDER W | 3 | 0.023 |
| SCHREURS LM | 3 | 0.023 |
| SCHREIBER D | 3 | 0.023 |
| SCHRAG D | 3 | 0.023 |
| SCHOPP M | 3 | 0.023 |
| SCHOON EJ | 3 | 0.023 |
| SCHONNEMANN KR | 3 | 0.023 |
| SCHOENNEMANN KR | 3 | 0.023 |
| SCHNOLL-SUSSMAN F | 3 | 0.023 |
| SCHNIDER A | 3 | 0.023 |
| SCHNEIDER BJ | 3 | 0.023 |
| SCHMIEGEL W | 3 | 0.023 |
| SCHMID T | 3 | 0.023 |
| SCHMID RM | 3 | 0.023 |
| SCHLECHTWEG N | 3 | 0.023 |
| SCHIRMACHER P | 3 | 0.023 |
| SCHEUNEMANN P | 3 | 0.023 |
| SCHERER V | 3 | 0.023 |
| SCHERER A | 3 | 0.023 |
| SCHENA M | 3 | 0.023 |
| SCHEMBRE DB | 3 | 0.023 |
| SCHELLENS JHM | 3 | 0.023 |
| SCHEFTER T | 3 | 0.023 |
| SCHANTZ MM | 3 | 0.023 |
| SAZUKA T | 3 | 0.023 |
| SAXENA PUP | 3 | 0.023 |
| SAWAKI A | 3 | 0.023 |
| SAWADA G | 3 | 0.023 |
| SAVAS S | 3 | 0.023 |
| SAVARINO E | 3 | 0.023 |
| SAUSSEZ S | 3 | 0.023 |
| SATTLER CA | 3 | 0.023 |
| SATOMURA H | 3 | 0.023 |
| SATOH S | 3 | 0.023 |
| SATO M | 3 | 0.023 |
| SATO C | 3 | 0.023 |
| SATA N | 3 | 0.023 |
| SASAJIMA K | 3 | 0.023 |
| SASAHIRA T | 3 | 0.023 |
| SAROSI GA | 3 | 0.023 |
| SARAYA A | 3 | 0.023 |
| SARANOVIC D | 3 | 0.023 |
| SAPKOTA A | 3 | 0.023 |
| SANTIBANEZ M | 3 | 0.023 |
| SANKARANARAYANAN R | 3 | 0.023 |
| SANG YH | 3 | 0.023 |
| SANDERS G | 3 | 0.023 |
| SANAKA MR | 3 | 0.023 |
| SAMPLINER RE | 3 | 0.023 |
| SALUJA SS | 3 | 0.023 |
| SALTZ LB | 3 | 0.023 |
| SALO JC | 3 | 0.023 |
| SALEHI M | 3 | 0.023 |
| SALAM I | 3 | 0.023 |
| SALADINI G | 3 | 0.023 |
| SAKON M | 3 | 0.023 |
| SAKANO T | 3 | 0.023 |
| SAKAMOTO A | 3 | 0.023 |
| SAKAI P | 3 | 0.023 |
| SAKAE T | 3 | 0.023 |
| SAITO A | 3 | 0.023 |
| SAIDI F | 3 | 0.023 |
| SAHANI DV | 3 | 0.023 |
| SAGAWA T | 3 | 0.023 |
| SAFE S | 3 | 0.023 |
| SADR-AZODI O | 3 | 0.023 |
| SADEGHI S | 3 | 0.023 |
| SAADEH L | 3 | 0.023 |
| RUTH KJ | 3 | 0.023 |
| RUTH K | 3 | 0.023 |
| RUMORE GJ | 3 | 0.023 |
| RUDIN CM | 3 | 0.023 |
| RUBINSTEIN HR | 3 | 0.023 |
| ROZEMA T | 3 | 0.023 |
| ROULLET B | 3 | 0.023 |
| ROTH W | 3 | 0.023 |
| ROTA M | 3 | 0.023 |
| ROSSINI A | 3 | 0.023 |
| ROSS P | 3 | 0.023 |
| ROSS JS | 3 | 0.023 |
| ROSS AS | 3 | 0.023 |
| ROSES RE | 3 | 0.023 |
| ROMANO O | 3 | 0.023 |
| ROHATGI A | 3 | 0.023 |
| ROESCH T | 3 | 0.023 |
| ROECKEN C | 3 | 0.023 |
| RODRIGUEZ S | 3 | 0.023 |
| RODRIGUEZ C | 3 | 0.023 |
| RODRIGUEZ A | 3 | 0.023 |
| RODRIGUES G | 3 | 0.023 |
| ROCHA C | 3 | 0.023 |
| ROBERTSON EV | 3 | 0.023 |
| RITTER G | 3 | 0.023 |
| RIKIYAMA T | 3 | 0.023 |
| RIHAWI K | 3 | 0.023 |
| RIES P | 3 | 0.023 |
| RIEDLINGER G | 3 | 0.023 |
| RICHEL D | 3 | 0.023 |
| RICHARDSON P | 3 | 0.023 |
| RICHARDS-KORTUM RR | 3 | 0.023 |
| RICHARDS DA | 3 | 0.023 |
| RICHARDS D | 3 | 0.023 |
| RICE T | 3 | 0.023 |
| RICE D | 3 | 0.023 |
| RICARDI U | 3 | 0.023 |
| REST CCL | 3 | 0.023 |
| RENDA A | 3 | 0.023 |
| REN JL | 3 | 0.023 |
| REN HZ | 3 | 0.023 |
| REINACHER-SCHICK A | 3 | 0.023 |
| REID T | 3 | 0.023 |
| REIBER C | 3 | 0.023 |
| REDDY JB | 3 | 0.023 |
| RAVI K | 3 | 0.023 |
| RAVELLI A | 3 | 0.023 |
| RASOULI M | 3 | 0.023 |
| RAPOZO DCM | 3 | 0.023 |
| RANTANEN T | 3 | 0.023 |
| RANGARAJAN V | 3 | 0.023 |
| RANDI G | 3 | 0.023 |
| RAMZAN Z | 3 | 0.023 |
| RAMUS JR | 3 | 0.023 |
| RAMANATHAN RK | 3 | 0.023 |
| RADERER M | 3 | 0.023 |
| RABKIN CS | 3 | 0.023 |
| QUESENBERRY CP | 3 | 0.023 |
| QU HH | 3 | 0.023 |
| QIU WS | 3 | 0.023 |
| QIU MT | 3 | 0.023 |
| QIN WZ | 3 | 0.023 |
| QIN SD | 3 | 0.023 |
| QIN R | 3 | 0.023 |
| QIAO Z | 3 | 0.023 |
| QIAO Y | 3 | 0.023 |
| QIAN J | 3 | 0.023 |
| QI Z | 3 | 0.023 |
| QI YJ | 3 | 0.023 |
| QAZI F | 3 | 0.023 |
| PYO S | 3 | 0.023 |
| PUTNAM JB | 3 | 0.023 |
| PURKAYASTHA S | 3 | 0.023 |
| PURANDARE NC | 3 | 0.023 |
| PUJOL B | 3 | 0.023 |
| PTOK H | 3 | 0.023 |
| PROBST A | 3 | 0.023 |
| PRITCHARD SA | 3 | 0.023 |
| PRINS MJD | 3 | 0.023 |
| PRIFTI S | 3 | 0.023 |
| PRESTON SR | 3 | 0.023 |
| PREDINA JD | 3 | 0.023 |
| PRAT F | 3 | 0.023 |
| PRASAD G | 3 | 0.023 |
| PRABHASH K | 3 | 0.023 |
| POWELL M | 3 | 0.023 |
| POST S | 3 | 0.023 |
| PONSKY JL | 3 | 0.023 |
| POLLOCK J | 3 | 0.023 |
| POLEY JW | 3 | 0.023 |
| POHLABELN H | 3 | 0.023 |
| POHL M | 3 | 0.023 |
| POHL J | 3 | 0.023 |
| POHL H | 3 | 0.023 |
| PLESEC T | 3 | 0.023 |
| PLANJERY V | 3 | 0.023 |
| PINNA AD | 3 | 0.023 |
| PIETRANTONIO F | 3 | 0.023 |
| PIESSEVAUX H | 3 | 0.023 |
| PHULUKDAREE A | 3 | 0.023 |
| PHOA KN | 3 | 0.023 |
| PHILLIPS BE | 3 | 0.023 |
| PHILIP PA | 3 | 0.023 |
| PHAM N | 3 | 0.023 |
| PFAU PR | 3 | 0.023 |
| PEZNER RD | 3 | 0.023 |
| PEYRE CG | 3 | 0.023 |
| PETO R | 3 | 0.023 |
| PETERS J | 3 | 0.023 |
| PETERS CJ | 3 | 0.023 |
| PETERS C | 3 | 0.023 |
| PERLMAN SB | 3 | 0.023 |
| PERKMANN T | 3 | 0.023 |
| PERICAY C | 3 | 0.023 |
| PEREZ K | 3 | 0.023 |
| PEREZ DR | 3 | 0.023 |
| PENNIMENT MG | 3 | 0.023 |
| PENNELLI G | 3 | 0.023 |
| PENMAN I | 3 | 0.023 |
| PENG Q | 3 | 0.023 |
| PENG GY | 3 | 0.023 |
| PELLEGRINI CA | 3 | 0.023 |
| PECKITT C | 3 | 0.023 |
| PAWAR Y | 3 | 0.023 |
| PAVLOVA SI | 3 | 0.023 |
| PAUN BC | 3 | 0.023 |
| PAUMIER A | 3 | 0.023 |
| PAUL A | 3 | 0.023 |
| PATIL S | 3 | 0.023 |
| PATEL V | 3 | 0.023 |
| PATEL SA | 3 | 0.023 |
| PATEL S | 3 | 0.023 |
| PASZKO E | 3 | 0.023 |
| PASSALACQUA R | 3 | 0.023 |
| PASELLO G | 3 | 0.023 |
| PARTRIDGE M | 3 | 0.023 |
| PARKER M | 3 | 0.023 |
| PARK YH | 3 | 0.023 |
| PARK SL | 3 | 0.023 |
| PARK SJ | 3 | 0.023 |
| PARK MH | 3 | 0.023 |
| PARK KU | 3 | 0.023 |
| PARK JW | 3 | 0.023 |
| PARK I | 3 | 0.023 |
| PARK HY | 3 | 0.023 |
| PARK HJ | 3 | 0.023 |
| PARIKH K | 3 | 0.023 |
| PARENTI A | 3 | 0.023 |
| PARASA S | 3 | 0.023 |
| PARAMESWARAN R | 3 | 0.023 |
| PANNALA R | 3 | 0.023 |
| PANG LW | 3 | 0.023 |
| PAN Z | 3 | 0.023 |
| PAN Y | 3 | 0.023 |
| PAN T | 3 | 0.023 |
| PAN QJ | 3 | 0.023 |
| PAN JJ | 3 | 0.023 |
| PAN JH | 3 | 0.023 |
| PAN F | 3 | 0.023 |
| PALMER MB | 3 | 0.023 |
| PALA V | 3 | 0.023 |
| PAK K | 3 | 0.023 |
| PAEZ D | 3 | 0.023 |
| PACKER S | 3 | 0.023 |
| PACCEZ JD | 3 | 0.023 |
| OZET A | 3 | 0.023 |
| OYEN WJG | 3 | 0.023 |
| OYA S | 3 | 0.023 |
| OVERHOLT BF | 3 | 0.023 |
| OUATTARA M | 3 | 0.023 |
| OTTO R | 3 | 0.023 |
| OSAROGIAGBON RU | 3 | 0.023 |
| OSADA S | 3 | 0.023 |
| ORTH K | 3 | 0.023 |
| ORRINGER M | 3 | 0.023 |
| ORDU AD | 3 | 0.023 |
| OOSTENBRUG LE | 3 | 0.023 |
| OOKI A | 3 | 0.023 |
| ONO T | 3 | 0.023 |
| ONO R | 3 | 0.023 |
| ONO M | 3 | 0.023 |
| ONITSUKA T | 3 | 0.023 |
| ONIMARU R | 3 | 0.023 |
| ONG CAJ | 3 | 0.023 |
| ONAITIS M | 3 | 0.023 |
| OMURTAG GZ | 3 | 0.023 |
| OMORI O | 3 | 0.023 |
| OMORI M | 3 | 0.023 |
| OMLOO JM | 3 | 0.023 |
| OMINAMI M | 3 | 0.023 |
| OMAE M | 3 | 0.023 |
| OLYAEE MS | 3 | 0.023 |
| OLSEN A | 3 | 0.023 |
| OLIVER JA | 3 | 0.023 |
| OKUYAMA M | 3 | 0.023 |
| OKUGAWA Y | 3 | 0.023 |
| OKUBO M | 3 | 0.023 |
| OKSALA N | 3 | 0.023 |
| OKAWA T | 3 | 0.023 |
| OKAMURA H | 3 | 0.023 |
| OKAMOTO H | 3 | 0.023 |
| OKAMI K | 3 | 0.023 |
| OKAHARA S | 3 | 0.023 |
| OISHI T | 3 | 0.023 |
| OHRR H | 3 | 0.023 |
| OHNUMA H | 3 | 0.023 |
| OHMIYA N | 3 | 0.023 |
| OHKURA Y | 3 | 0.023 |
| OHISHI K | 3 | 0.023 |
| OHASHI M | 3 | 0.023 |
| OH Y | 3 | 0.023 |
| OH D | 3 | 0.023 |
| OGURA G | 3 | 0.023 |
| OGO E | 3 | 0.023 |
| OGISO S | 3 | 0.023 |
| OGINO H | 3 | 0.023 |
| OGAWA A | 3 | 0.023 |
| OBLAK I | 3 | 0.023 |
| O'SULLIVAN KE | 3 | 0.023 |
| O'SULLIVAN GC | 3 | 0.023 |
| O'DONOVAN M | 3 | 0.023 |
| NUMANS ME | 3 | 0.023 |
| NUKAYA I | 3 | 0.023 |
| NOVELLI M | 3 | 0.023 |
| NOSHIRO H | 3 | 0.023 |
| NORTON JA | 3 | 0.023 |
| NORONHA V | 3 | 0.023 |
| NORDSMARK M | 3 | 0.023 |
| NOMURA K | 3 | 0.023 |
| NOEL G | 3 | 0.023 |
| NODIN B | 3 | 0.023 |
| NODA E | 3 | 0.023 |
| NJOBEH PB | 3 | 0.023 |
| NIXON L | 3 | 0.023 |
| NIU ZX | 3 | 0.023 |
| NIU YM | 3 | 0.023 |
| NIU J | 3 | 0.023 |
| NIU HJ | 3 | 0.023 |
| NISHITANI H | 3 | 0.023 |
| NISHIOKA NS | 3 | 0.023 |
| NISHIKAWA Y | 3 | 0.023 |
| NISHIDA Y | 3 | 0.023 |
| NIKLINSKA W | 3 | 0.023 |
| NIKI T | 3 | 0.023 |
| NIJSTEN MWN | 3 | 0.023 |
| NIIMI M | 3 | 0.023 |
| NICOLINI G | 3 | 0.023 |
| NICOLAY NH | 3 | 0.023 |
| NICOLAS-PEREZ D | 3 | 0.023 |
| NG SH | 3 | 0.023 |
| NG D | 3 | 0.023 |
| NEWTON R | 3 | 0.023 |
| NESI G | 3 | 0.023 |
| NASU J | 3 | 0.023 |
| NARITA Y | 3 | 0.023 |
| NARDONE G | 3 | 0.023 |
| NARAZAKI K | 3 | 0.023 |
| NARAYAN S | 3 | 0.023 |
| NANJO H | 3 | 0.023 |
| NANCARROW DJ | 3 | 0.023 |
| NANAMI T | 3 | 0.023 |
| NAM T | 3 | 0.023 |
| NAM H | 3 | 0.023 |
| NAM BH | 3 | 0.023 |
| NAKUI M | 3 | 0.023 |
| NAKATANI M | 3 | 0.023 |
| NAKATANI K | 3 | 0.023 |
| NAKATA K | 3 | 0.023 |
| NAKANISHI H | 3 | 0.023 |
| NAKAMURA C | 3 | 0.023 |
| NAKAJIMA J | 3 | 0.023 |
| NAKAHARA R | 3 | 0.023 |
| NAJAFI F | 3 | 0.023 |
| NAGATA C | 3 | 0.023 |
| NAGAO T | 3 | 0.023 |
| NAGANAWA Y | 3 | 0.023 |
| NAGAI H | 3 | 0.023 |
| NABI S | 3 | 0.023 |
| NA KJ | 3 | 0.023 |
| NA HK | 3 | 0.023 |
| MYLES BH | 3 | 0.023 |
| MWANZA M | 3 | 0.023 |
| MUTREJA K | 3 | 0.023 |
| MUSICI S | 3 | 0.023 |
| MURRAY WK | 3 | 0.023 |
| MURPHY A | 3 | 0.023 |
| MURATA S | 3 | 0.023 |
| MURAKAMI A | 3 | 0.023 |
| MULLER LB | 3 | 0.023 |
| MULDER KE | 3 | 0.023 |
| MUL V | 3 | 0.023 |
| MUKAISHO K | 3 | 0.023 |
| MUELLER RP | 3 | 0.023 |
| MUELLER J | 3 | 0.023 |
| MOWLA SJ | 3 | 0.023 |
| MOSS SF | 3 | 0.023 |
| MORSE MA | 3 | 0.023 |
| MORSCHE RHMT | 3 | 0.023 |
| MORNEX F | 3 | 0.023 |
| MORIYASU F | 3 | 0.023 |
| MORIUCHI T | 3 | 0.023 |
| MORI T | 3 | 0.023 |
| MORGENSTERN H | 3 | 0.023 |
| MOORTHY K | 3 | 0.023 |
| MOORE M | 3 | 0.023 |
| MOORCRAFT SY | 3 | 0.023 |
| MOON SW | 3 | 0.023 |
| MONTERO AJ | 3 | 0.023 |
| MONNIER P | 3 | 0.023 |
| MONJAZEB A | 3 | 0.023 |
| MONGES G | 3 | 0.023 |
| MOLINA E | 3 | 0.023 |
| MOHIUDDIN K | 3 | 0.023 |
| MOEHLER MH | 3 | 0.023 |
| MIZOGUCHI N | 3 | 0.023 |
| MIZOGUCHI K | 3 | 0.023 |
| MIYOSHI N | 3 | 0.023 |
| MIYATA K | 3 | 0.023 |
| MIYAMOTO T | 3 | 0.023 |
| MIYAMAE M | 3 | 0.023 |
| MITTLBOCK M | 3 | 0.023 |
| MITTAL S | 3 | 0.023 |
| MITRY E | 3 | 0.023 |
| MITRA N | 3 | 0.023 |
| MITHANI SK | 3 | 0.023 |
| MITAMURA A | 3 | 0.023 |
| MIRZA A | 3 | 0.023 |
| MINO-KENUDSON M | 3 | 0.023 |
| MINGRONE W | 3 | 0.023 |
| MING L | 3 | 0.023 |
| MINEUR L | 3 | 0.023 |
| MINENO J | 3 | 0.023 |
| MINEMATSU H | 3 | 0.023 |
| MIN H | 3 | 0.023 |
| MIMA K | 3 | 0.023 |
| MILLER DL | 3 | 0.023 |
| MILLER D | 3 | 0.023 |
| MILDENBERGER P | 3 | 0.023 |
| MILBY AB | 3 | 0.023 |
| MIKI Y | 3 | 0.023 |
| MIKI A | 3 | 0.023 |
| MIHARA Y | 3 | 0.023 |
| MIENO H | 3 | 0.023 |
| MICK R | 3 | 0.023 |
| MICHEL A | 3 | 0.023 |
| MICHAEL MZ | 3 | 0.023 |
| MEYERS B | 3 | 0.023 |
| MEULENDIJKS D | 3 | 0.023 |
| METZGER U | 3 | 0.023 |
| MESTERI I | 3 | 0.023 |
| MESCOLI C | 3 | 0.023 |
| MERTINEIT N | 3 | 0.023 |
| MERIGLIANO S | 3 | 0.023 |
| MENON D | 3 | 0.023 |
| MENG JY | 3 | 0.023 |
| MENEZES A | 3 | 0.023 |
| MENASHEROV N | 3 | 0.023 |
| MEIJER GA | 3 | 0.023 |
| MEIER CR | 3 | 0.023 |
| MEHRAN R | 3 | 0.023 |
| MEGUID RA | 3 | 0.023 |
| MEENAN J | 3 | 0.023 |
| MCNAIR AGK | 3 | 0.023 |
| MCMURRY TL | 3 | 0.023 |
| MCMAHON BP | 3 | 0.023 |
| MCCALL SJ | 3 | 0.023 |
| MAZIAK DE | 3 | 0.023 |
| MAYNE GC | 3 | 0.023 |
| MAY AM | 3 | 0.023 |
| MAWLAWI O | 3 | 0.023 |
| MATUSIEWICZ M | 3 | 0.023 |
| MATUSCHEK C | 3 | 0.023 |
| MATTIOLI S | 3 | 0.023 |
| MATSUZAKI J | 3 | 0.023 |
| MATSUURA T | 3 | 0.023 |
| MATSUURA H | 3 | 0.023 |
| MATSUURA B | 3 | 0.023 |
| MATSUSHITA S | 3 | 0.023 |
| MATSUSHITA D | 3 | 0.023 |
| MATSUSAKA S | 3 | 0.023 |
| MATSUO Y | 3 | 0.023 |
| MATSUNAGA N | 3 | 0.023 |
| MATSUI Y | 3 | 0.023 |
| MATSUDA M | 3 | 0.023 |
| MATOS E | 3 | 0.023 |
| MATHEY K | 3 | 0.023 |
| MATAMOROS A | 3 | 0.023 |
| MASUDA K | 3 | 0.023 |
| MAST R | 3 | 0.023 |
| MARTONI AA | 3 | 0.023 |
| MARTONI A | 3 | 0.023 |
| MARTINEZ J | 3 | 0.023 |
| MARTIN-RICHARD M | 3 | 0.023 |
| MARTIN RC | 3 | 0.023 |
| MARTIN NE | 3 | 0.023 |
| MARTIN L | 3 | 0.023 |
| MARTIN JF | 3 | 0.023 |
| MARTI GP | 3 | 0.023 |
| MARTENSON JA | 3 | 0.023 |
| MARSHALL REK | 3 | 0.023 |
| MARSHALL AL | 3 | 0.023 |
| MARSH S | 3 | 0.023 |
| MARKL B | 3 | 0.023 |
| MARK SD | 3 | 0.023 |
| MARESCH J | 3 | 0.023 |
| MAO QX | 3 | 0.023 |
| MAO AW | 3 | 0.023 |
| MANABE N | 3 | 0.023 |
| MAMEDE M | 3 | 0.023 |
| MALIK NK | 3 | 0.023 |
| MALEKZADEH MM | 3 | 0.023 |
| MALEKSHAH AFT | 3 | 0.023 |
| MALEKSHAH AF | 3 | 0.023 |
| MAKIURA D | 3 | 0.023 |
| MAKITA C | 3 | 0.023 |
| MAKINO M | 3 | 0.023 |
| MAK RH | 3 | 0.023 |
| MAITRA A | 3 | 0.023 |
| MAI S | 3 | 0.023 |
| MAHMUD A | 3 | 0.023 |
| MAGNO P | 3 | 0.023 |
| MAEMURA K | 3 | 0.023 |
| MAEDA T | 3 | 0.023 |
| MADANICK RD | 3 | 0.023 |
| MADANI A | 3 | 0.023 |
| MACKAY H | 3 | 0.023 |
| MACHIELS M | 3 | 0.023 |
| MACHIDA H | 3 | 0.023 |
| MACAPINLAC H | 3 | 0.023 |
| MAAK M | 3 | 0.023 |
| MA Y | 3 | 0.023 |
| MA XJ | 3 | 0.023 |
| MA WJ | 3 | 0.023 |
| MA RL | 3 | 0.023 |
| MA P | 3 | 0.023 |
| MA M | 3 | 0.023 |
| MA JL | 3 | 0.023 |
| MA HT | 3 | 0.023 |
| MA HH | 3 | 0.023 |
| LYSAGHT J | 3 | 0.023 |
| LV W | 3 | 0.023 |
| LV JM | 3 | 0.023 |
| LV JJ | 3 | 0.023 |
| LV HB | 3 | 0.023 |
| LUTHRA R | 3 | 0.023 |
| LUO ZM | 3 | 0.023 |
| LUO ZG | 3 | 0.023 |
| LUO YH | 3 | 0.023 |
| LUO T | 3 | 0.023 |
| LUO SX | 3 | 0.023 |
| LUO M | 3 | 0.023 |
| LUO JY | 3 | 0.023 |
| LUO JH | 3 | 0.023 |
| LUO JF | 3 | 0.023 |
| LUO HY | 3 | 0.023 |
| LUO HS | 3 | 0.023 |
| LUO B | 3 | 0.023 |
| LUNET N | 3 | 0.023 |
| LULEY KB | 3 | 0.023 |
| LUKETICH J | 3 | 0.023 |
| LUBIN JH | 3 | 0.023 |
| LU YF | 3 | 0.023 |
| LU SX | 3 | 0.023 |
| LU PJ | 3 | 0.023 |
| LU NH | 3 | 0.023 |
| LU JG | 3 | 0.023 |
| LU JB | 3 | 0.023 |
| LU CX | 3 | 0.023 |
| LOVAT L | 3 | 0.023 |
| LOU JN | 3 | 0.023 |
| LOREN D | 3 | 0.023 |
| LORD SJ | 3 | 0.023 |
| LOPES LR | 3 | 0.023 |
| LOPES AB | 3 | 0.023 |
| LOBO DN | 3 | 0.023 |
| LO JL | 3 | 0.023 |
| LLOYD S | 3 | 0.023 |
| LIVINGSTONE A | 3 | 0.023 |
| LIU ZX | 3 | 0.023 |
| LIU ZS | 3 | 0.023 |
| LIU ZL | 3 | 0.023 |
| LIU ZJ | 3 | 0.023 |
| LIU ZG | 3 | 0.023 |
| LIU YW | 3 | 0.023 |
| LIU YB | 3 | 0.023 |
| LIU XP | 3 | 0.023 |
| LIU XD | 3 | 0.023 |
| LIU XB | 3 | 0.023 |
| LIU WZ | 3 | 0.023 |
| LIU WX | 3 | 0.023 |
| LIU WG | 3 | 0.023 |
| LIU QL | 3 | 0.023 |
| LIU PF | 3 | 0.023 |
| LIU NR | 3 | 0.023 |
| LIU KY | 3 | 0.023 |
| LIU JZ | 3 | 0.023 |
| LIU JM | 3 | 0.023 |
| LIU JB | 3 | 0.023 |
| LIU HQ | 3 | 0.023 |
| LIU HC | 3 | 0.023 |
| LIU GL | 3 | 0.023 |
| LIU FX | 3 | 0.023 |
| LIU DS | 3 | 0.023 |
| LIPHSHITZ I | 3 | 0.023 |
| LINK BC | 3 | 0.023 |
| LING MT | 3 | 0.023 |
| LINET MS | 3 | 0.023 |
| LIN WC | 3 | 0.023 |
| LIN RY | 3 | 0.023 |
| LIN RH | 3 | 0.023 |
| LIN JQ | 3 | 0.023 |
| LIN JF | 3 | 0.023 |
| LIN HH | 3 | 0.023 |
| LIN F | 3 | 0.023 |
| LIN CL | 3 | 0.023 |
| LIMA SCS | 3 | 0.023 |
| LIMA M | 3 | 0.023 |
| LIM HY | 3 | 0.023 |
| LIM D | 3 | 0.023 |
| LIKHACHEVA A | 3 | 0.023 |
| LIEPA AM | 3 | 0.023 |
| LIANG YM | 3 | 0.023 |
| LIANG M | 3 | 0.023 |
| LIANG H | 3 | 0.023 |
| LIANG B | 3 | 0.023 |
| LI ZZ | 3 | 0.023 |
| LI ZX | 3 | 0.023 |
| LI ZR | 3 | 0.023 |
| LI YW | 3 | 0.023 |
| LI YC | 3 | 0.023 |
| LI YB | 3 | 0.023 |
| LI XS | 3 | 0.023 |
| LI XD | 3 | 0.023 |
| LI XA | 3 | 0.023 |
| LI WL | 3 | 0.023 |
| LI TJ | 3 | 0.023 |
| LI MN | 3 | 0.023 |
| LI MB | 3 | 0.023 |
| LI LB | 3 | 0.023 |
| LI JT | 3 | 0.023 |
| LI JM | 3 | 0.023 |
| LI JG | 3 | 0.023 |
| LI HK | 3 | 0.023 |
| LI HJ | 3 | 0.023 |
| LI HB | 3 | 0.023 |
| LI GL | 3 | 0.023 |
| LI G | 3 | 0.023 |
| LI DQ | 3 | 0.023 |
| LI DF | 3 | 0.023 |
| LI DD | 3 | 0.023 |
| LI CS | 3 | 0.023 |
| LI CF | 3 | 0.023 |
| LI BZ | 3 | 0.023 |
| LI AFY | 3 | 0.023 |
| LI A | 3 | 0.023 |
| LEWIS JT | 3 | 0.023 |
| LEWIS J | 3 | 0.023 |
| LEWIECKI EM | 3 | 0.023 |
| LEVIN TR | 3 | 0.023 |
| LEVEA CM | 3 | 0.023 |
| LEVEA C | 3 | 0.023 |
| LEUZZI G | 3 | 0.023 |
| LERCO MM | 3 | 0.023 |
| LENNARTSSON J | 3 | 0.023 |
| LENG WD | 3 | 0.023 |
| LEICHMAN LP | 3 | 0.023 |
| LEFORT C | 3 | 0.023 |
| LEFOR A | 3 | 0.023 |
| LEEUWENBURGH I | 3 | 0.023 |
| LEE YH | 3 | 0.023 |
| LEE YCA | 3 | 0.023 |
| LEE WH | 3 | 0.023 |
| LEE TS | 3 | 0.023 |
| LEE TJ | 3 | 0.023 |
| LEE SC | 3 | 0.023 |
| LEE NPY | 3 | 0.023 |
| LEE MH | 3 | 0.023 |
| LEE M | 3 | 0.023 |
| LEE K | 3 | 0.023 |
| LEE JK | 3 | 0.023 |
| LEE HY | 3 | 0.023 |
| LEE EJ | 3 | 0.023 |
| LEE EH | 3 | 0.023 |
| LEE CC | 3 | 0.023 |
| LECHNER JF | 3 | 0.023 |
| LEBLANC J | 3 | 0.023 |
| LATIFI K | 3 | 0.023 |
| LASSEN U | 3 | 0.023 |
| LARGHI A | 3 | 0.023 |
| LAPIN B | 3 | 0.023 |
| LAPAR DJ | 3 | 0.023 |
| LANGMARK F | 3 | 0.023 |
| LANG JY | 3 | 0.023 |
| LAMMERING G | 3 | 0.023 |
| LAMART S | 3 | 0.023 |
| LAM AKY | 3 | 0.023 |
| LAI YL | 3 | 0.023 |
| LAI WW | 3 | 0.023 |
| LAI SW | 3 | 0.023 |
| LAI L | 3 | 0.023 |
| LAI KKY | 3 | 0.023 |
| LACHAT C | 3 | 0.023 |
| LABONTE MJ | 3 | 0.023 |
| LA T | 3 | 0.023 |
| KWEE RM | 3 | 0.023 |
| KWAN PS | 3 | 0.023 |
| KWAH J | 3 | 0.023 |
| KUZDZAL J | 3 | 0.023 |
| KUWAJIMA A | 3 | 0.023 |
| KUWABARA S | 3 | 0.023 |
| KUSTERS JG | 3 | 0.023 |
| KUSAFUKA K | 3 | 0.023 |
| KURZROCK R | 3 | 0.023 |
| KUROGOCHI T | 3 | 0.023 |
| KURODA S | 3 | 0.023 |
| KURIYAMA S | 3 | 0.023 |
| KURIBAYASHI Y | 3 | 0.023 |
| KURIAN AA | 3 | 0.023 |
| KURAOKA K | 3 | 0.023 |
| KUPCSULIK PK | 3 | 0.023 |
| KUO CT | 3 | 0.023 |
| KUNZMANN R | 3 | 0.023 |
| KUNZ S | 3 | 0.023 |
| KUNIYASU H | 3 | 0.023 |
| KUNIEDA E | 3 | 0.023 |
| KUNERT H | 3 | 0.023 |
| KUME M | 3 | 0.023 |
| KUMAR M | 3 | 0.023 |
| KUMABE A | 3 | 0.023 |
| KULLOLLI S | 3 | 0.023 |
| KULIG J | 3 | 0.023 |
| KUBO H | 3 | 0.023 |
| KUBISA B | 3 | 0.023 |
| KU FC | 3 | 0.023 |
| KSHIVETS O | 3 | 0.023 |
| KRZYSTEK-KORPACKA M | 3 | 0.023 |
| KRUG B | 3 | 0.023 |
| KROEMER G | 3 | 0.023 |
| KRISHNA S | 3 | 0.023 |
| KRIPP M | 3 | 0.023 |
| KRESTY LA | 3 | 0.023 |
| KRANZFELDER M | 3 | 0.023 |
| KRAMAR A | 3 | 0.023 |
| KRAJA B | 3 | 0.023 |
| KOZUKA T | 3 | 0.023 |
| KOZONO T | 3 | 0.023 |
| KOZARSKI R | 3 | 0.023 |
| KOYAMA T | 3 | 0.023 |
| KOUNTOURAS J | 3 | 0.023 |
| KOU TD | 3 | 0.023 |
| KOTO M | 3 | 0.023 |
| KOSHENKOV VP | 3 | 0.023 |
| KORU-SENGUL T | 3 | 0.023 |
| KORST RJ | 3 | 0.023 |
| KORIYAMA C | 3 | 0.023 |
| KORFAGE IJ | 3 | 0.023 |
| KORDES S | 3 | 0.023 |
| KOPP HG | 3 | 0.023 |
| KOOM WS | 3 | 0.023 |
| KOOBY DA | 3 | 0.023 |
| KONNO H | 3 | 0.023 |
| KONG RR | 3 | 0.023 |
| KONG QQ | 3 | 0.023 |
| KONG QP | 3 | 0.023 |
| KONG CY | 3 | 0.023 |
| KONDO M | 3 | 0.023 |
| KOMORI A | 3 | 0.023 |
| KOMATSU D | 3 | 0.023 |
| KOLODZIEJCZYK P | 3 | 0.023 |
| KOKURA S | 3 | 0.023 |
| KOIZUMI Y | 3 | 0.023 |
| KOIZUMI S | 3 | 0.023 |
| KOINUMA J | 3 | 0.023 |
| KOIKE Y | 3 | 0.023 |
| KOIDE Y | 3 | 0.023 |
| KOHNO N | 3 | 0.023 |
| KOFOED SC | 3 | 0.023 |
| KOEBERLE D | 3 | 0.023 |
| KOCHA W | 3 | 0.023 |
| KOBAYASHI R | 3 | 0.023 |
| KOBAYASHI H | 3 | 0.023 |
| KOBARA H | 3 | 0.023 |
| KO JMY | 3 | 0.023 |
| KO AH | 3 | 0.023 |
| KNABE M | 3 | 0.023 |
| KLINK CD | 3 | 0.023 |
| KLIMSTRA D | 3 | 0.023 |
| KLEMPNER SJ | 3 | 0.023 |
| KLEIN CA | 3 | 0.023 |
| KIYOZAKI H | 3 | 0.023 |
| KIYOTA N | 3 | 0.023 |
| KIUCHI Y | 3 | 0.023 |
| KITAZAWA M | 3 | 0.023 |
| KITAMURA S | 3 | 0.023 |
| KITAMURA M | 3 | 0.023 |
| KITAJIMA T | 3 | 0.023 |
| KITAJIMA S | 3 | 0.023 |
| KITADANI J | 3 | 0.023 |
| KISS R | 3 | 0.023 |
| KIROVA YM | 3 | 0.023 |
| KIRCHER S | 3 | 0.023 |
| KINUGASA S | 3 | 0.023 |
| KINUGASA H | 3 | 0.023 |
| KINJO Y | 3 | 0.023 |
| KINGHORN AD | 3 | 0.023 |
| KINDLER HL | 3 | 0.023 |
| KIM WJ | 3 | 0.023 |
| KIM TY | 3 | 0.023 |
| KIM TJ | 3 | 0.023 |
| KIM R | 3 | 0.023 |
| KIM KS | 3 | 0.023 |
| KIM KR | 3 | 0.023 |
| KIM HG | 3 | 0.023 |
| KIM HC | 3 | 0.023 |
| KIM GJ | 3 | 0.023 |
| KIM CY | 3 | 0.023 |
| KIM CH | 3 | 0.023 |
| KIM BW | 3 | 0.023 |
| KIM BT | 3 | 0.023 |
| KIKUCHI T | 3 | 0.023 |
| KIESSLING R | 3 | 0.023 |
| KIESER M | 3 | 0.023 |
| KIDANE B | 3 | 0.023 |
| KIDA K | 3 | 0.023 |
| KHUSHALANI N | 3 | 0.023 |
| KHONG PL | 3 | 0.023 |
| KHITHANI AS | 3 | 0.023 |
| KHATTAK S | 3 | 0.023 |
| KHAN T | 3 | 0.023 |
| KHAN MA | 3 | 0.023 |
| KEY TJ | 3 | 0.023 |
| KESHAVJEE S | 3 | 0.023 |
| KESHAVARZI B | 3 | 0.023 |
| KERNSTINE K | 3 | 0.023 |
| KENNEDY CW | 3 | 0.023 |
| KELSEY CR | 3 | 0.023 |
| KELLY LA | 3 | 0.023 |
| KELLEY S | 3 | 0.023 |
| KAZUMOTO T | 3 | 0.023 |
| KAWASHIMA Y | 3 | 0.023 |
| KAWASAKI Y | 3 | 0.023 |
| KAWASAKI H | 3 | 0.023 |
| KAWAMURA O | 3 | 0.023 |
| KAWAMOTO T | 3 | 0.023 |
| KAWAMOTO K | 3 | 0.023 |
| KAWAKITA D | 3 | 0.023 |
| KAWAI H | 3 | 0.023 |
| KAWAGUCHI O | 3 | 0.023 |
| KAWABE A | 3 | 0.023 |
| KAVADI V | 3 | 0.023 |
| KAUPPI J | 3 | 0.023 |
| KATSURA K | 3 | 0.023 |
| KATOH Y | 3 | 0.023 |
| KATOH R | 3 | 0.023 |
| KATHIRVEL M | 3 | 0.023 |
| KATAOKA H | 3 | 0.023 |
| KATAKI AC | 3 | 0.023 |
| KATAKI A | 3 | 0.023 |
| KATAGIRI Y | 3 | 0.023 |
| KASHIWAGI K | 3 | 0.023 |
| KASCHULA CH | 3 | 0.023 |
| KARRAN A | 3 | 0.023 |
| KARIM-KOS HE | 3 | 0.023 |
| KARALIOTAS C | 3 | 0.023 |
| KARAKASHEVA TA | 3 | 0.023 |
| KAPPEL S | 3 | 0.023 |
| KAPLAN I | 3 | 0.023 |
| KAO S | 3 | 0.023 |
| KANZLER S | 3 | 0.023 |
| KANO Y | 3 | 0.023 |
| KANG NN | 3 | 0.023 |
| KANEMURA T | 3 | 0.023 |
| KANEKO T | 3 | 0.023 |
| KANEHIRA E | 3 | 0.023 |
| KANAZAWA Y | 3 | 0.023 |
| KANAZAWA S | 3 | 0.023 |
| KANAYA S | 3 | 0.023 |
| KANAI Y | 3 | 0.023 |
| KANAI N | 3 | 0.023 |
| KAN QC | 3 | 0.023 |
| KAMIYA K | 3 | 0.023 |
| KAMINISHI M | 3 | 0.023 |
| KAMEYAMA K | 3 | 0.023 |
| KAMATA M | 3 | 0.023 |
| KALMAN S | 3 | 0.023 |
| KALFF V | 3 | 0.023 |
| KAKIMI K | 3 | 0.023 |
| KAJIWARA T | 3 | 0.023 |
| KAISER GM | 3 | 0.023 |
| KAIRA K | 3 | 0.023 |
| KAIJSER M | 3 | 0.023 |
| KAIGA T | 3 | 0.023 |
| KAIDAR-PERSON O | 3 | 0.023 |
| KAGAWA Y | 3 | 0.023 |
| KAGANOI J | 3 | 0.023 |
| KADOYA K | 3 | 0.023 |
| KADLA SA | 3 | 0.023 |
| JUNG I | 3 | 0.023 |
| JOSHI S | 3 | 0.023 |
| JOSHI A | 3 | 0.023 |
| JOSHAGHANI H | 3 | 0.023 |
| JOHNSTONE C | 3 | 0.023 |
| JOHNSTON MH | 3 | 0.023 |
| JOHNSSON E | 3 | 0.023 |
| JOHANSEN D | 3 | 0.023 |
| JOENSUU H | 3 | 0.023 |
| JIRSTROM K | 3 | 0.023 |
| JIRAWAT S | 3 | 0.023 |
| JING XB | 3 | 0.023 |
| JING C | 3 | 0.023 |
| JIN YS | 3 | 0.023 |
| JIN XF | 3 | 0.023 |
| JIN LX | 3 | 0.023 |
| JIN C | 3 | 0.023 |
| JIAO ZJ | 3 | 0.023 |
| JIAO Y | 3 | 0.023 |
| JIAO WJ | 3 | 0.023 |
| JIAO F | 3 | 0.023 |
| JIANG ZM | 3 | 0.023 |
| JIANG YX | 3 | 0.023 |
| JIANG YJ | 3 | 0.023 |
| JIANG YH | 3 | 0.023 |
| JIANG XR | 3 | 0.023 |
| JIANG WJ | 3 | 0.023 |
| JIANG R | 3 | 0.023 |
| JIANG QW | 3 | 0.023 |
| JIANG QF | 3 | 0.023 |
| JIANG PC | 3 | 0.023 |
| JIANG JQ | 3 | 0.023 |
| JIANG GJ | 3 | 0.023 |
| JIA XM | 3 | 0.023 |
| JIA R | 3 | 0.023 |
| JI YH | 3 | 0.023 |
| JI J | 3 | 0.023 |
| JI HL | 3 | 0.023 |
| JI AF | 3 | 0.023 |
| JHAMB J | 3 | 0.023 |
| JEROMEN A | 3 | 0.023 |
| JEREMIC B | 3 | 0.023 |
| JEON HW | 3 | 0.023 |
| JENKINS RB | 3 | 0.023 |
| JAYASEKERA CS | 3 | 0.023 |
| JAY J | 3 | 0.023 |
| JAVERI H | 3 | 0.023 |
| JAVED A | 3 | 0.023 |
| JANSSON C | 3 | 0.023 |
| JANSEN EPM | 3 | 0.023 |
| JANG YH | 3 | 0.023 |
| JANG SY | 3 | 0.023 |
| JANG HJ | 3 | 0.023 |
| JAMASBI RJ | 3 | 0.023 |
| JAKSZYN P | 3 | 0.023 |
| JAKLITSCH MT | 3 | 0.023 |
| JAHNE J | 3 | 0.023 |
| JAGER PL | 3 | 0.023 |
| JAFFEE EM | 3 | 0.023 |
| JACKSON LS | 3 | 0.023 |
| JABBARI A | 3 | 0.023 |
| IZZO J | 3 | 0.023 |
| IYER RV | 3 | 0.023 |
| IWATA H | 3 | 0.023 |
| ITOH F | 3 | 0.023 |
| ITATSU K | 3 | 0.023 |
| ISONO K | 3 | 0.023 |
| ISHINO Y | 3 | 0.023 |
| ISHIKAWA M | 3 | 0.023 |
| ISHIHARA S | 3 | 0.023 |
| ISAYAMA F | 3 | 0.023 |
| IRINO T | 3 | 0.023 |
| INAZAWA J | 3 | 0.023 |
| IMMANUEL A | 3 | 0.023 |
| IMDAHL A | 3 | 0.023 |
| IMAZEKI H | 3 | 0.023 |
| IMANISHI S | 3 | 0.023 |
| IMAMURA H | 3 | 0.023 |
| IMAI A | 3 | 0.023 |
| IM YH | 3 | 0.023 |
| IKEGUCHI M | 3 | 0.023 |
| IKEDA O | 3 | 0.023 |
| IKEDA N | 3 | 0.023 |
| IGISSINOVA G | 3 | 0.023 |
| IDE S | 3 | 0.023 |
| ICHIKURA T | 3 | 0.023 |
| IAFRATE AJ | 3 | 0.023 |
| HWANG SH | 3 | 0.023 |
| HWANG J | 3 | 0.023 |
| HWANG IG | 3 | 0.023 |
| HUSSAIN Z | 3 | 0.023 |
| HUSAIN AN | 3 | 0.023 |
| HUO X | 3 | 0.023 |
| HUNG YH | 3 | 0.023 |
| HUNG RJ | 3 | 0.023 |
| HUMPF HU | 3 | 0.023 |
| HULSHOFF J | 3 | 0.023 |
| HUIJSMANS J | 3 | 0.023 |
| HUBNER M | 3 | 0.023 |
| HUBKA M | 3 | 0.023 |
| HUANG ZL | 3 | 0.023 |
| HUANG ZG | 3 | 0.023 |
| HUANG ZF | 3 | 0.023 |
| HUANG YL | 3 | 0.023 |
| HUANG XC | 3 | 0.023 |
| HUANG WY | 3 | 0.023 |
| HUANG WL | 3 | 0.023 |
| HUANG MJ | 3 | 0.023 |
| HUANG MF | 3 | 0.023 |
| HUANG JX | 3 | 0.023 |
| HUANG JS | 3 | 0.023 |
| HUANG GL | 3 | 0.023 |
| HUANG GH | 3 | 0.023 |
| HUANG FR | 3 | 0.023 |
| HUANG CL | 3 | 0.023 |
| HUANG CC | 3 | 0.023 |
| HUA ZL | 3 | 0.023 |
| HU ZQ | 3 | 0.023 |
| HU YL | 3 | 0.023 |
| HU XP | 3 | 0.023 |
| HU WP | 3 | 0.023 |
| HU WG | 3 | 0.023 |
| HU T | 3 | 0.023 |
| HU RG | 3 | 0.023 |
| HU LY | 3 | 0.023 |
| HU LJ | 3 | 0.023 |
| HU JJ | 3 | 0.023 |
| HU FJ | 3 | 0.023 |
| HU CY | 3 | 0.023 |
| HSUEH C | 3 | 0.023 |
| HSU Y | 3 | 0.023 |
| HSU F | 3 | 0.023 |
| HSU CC | 3 | 0.023 |
| HOWINGTON JA | 3 | 0.023 |
| HOWELL D | 3 | 0.023 |
| HOWARD JM | 3 | 0.023 |
| HOU YY | 3 | 0.023 |
| HOU X | 3 | 0.023 |
| HOU L | 3 | 0.023 |
| HOU B | 3 | 0.023 |
| HOSSEIN-NEZHAD A | 3 | 0.023 |
| HOSONE M | 3 | 0.023 |
| HOSOE N | 3 | 0.023 |
| HOSHINO E | 3 | 0.023 |
| HOSHIHARA Y | 3 | 0.023 |
| HOSHIDA T | 3 | 0.023 |
| HOSHI N | 3 | 0.023 |
| HOSCH SB | 3 | 0.023 |
| HOSAKA H | 3 | 0.023 |
| HORVATH OP | 3 | 0.023 |
| HORNBY J | 3 | 0.023 |
| HORIKI N | 3 | 0.023 |
| HORIGUCHI H | 3 | 0.023 |
| HORIBE D | 3 | 0.023 |
| HORI Y | 3 | 0.023 |
| HORI S | 3 | 0.023 |
| HOPPER AN | 3 | 0.023 |
| HOPFNER M | 3 | 0.023 |
| HONMA K | 3 | 0.023 |
| HONJYO H | 3 | 0.023 |
| HONGO M | 3 | 0.023 |
| HONG WK | 3 | 0.023 |
| HONG R | 3 | 0.023 |
| HONG JA | 3 | 0.023 |
| HONG DS | 3 | 0.023 |
| HONG CC | 3 | 0.023 |
| HONG B | 3 | 0.023 |
| HOLTVED E | 3 | 0.023 |
| HOLD GL | 3 | 0.023 |
| HOEKSTRA OS | 3 | 0.023 |
| HOEHLER T | 3 | 0.023 |
| HOEFLER H | 3 | 0.023 |
| HIYAMA K | 3 | 0.023 |
| HIYAMA E | 3 | 0.023 |
| HIWASA T | 3 | 0.023 |
| HISLOP G | 3 | 0.023 |
| HIRT N | 3 | 0.023 |
| HIROTA J | 3 | 0.023 |
| HIROHASHI S | 3 | 0.023 |
| HIRDES MM | 3 | 0.023 |
| HIRASAWA D | 3 | 0.023 |
| HIRAO T | 3 | 0.023 |
| HIRAKI T | 3 | 0.023 |
| HIRAJIMA S | 3 | 0.023 |
| HIRABAYASHI Y | 3 | 0.023 |
| HIMPENS J | 3 | 0.023 |
| HILDEBRANDT MAT | 3 | 0.023 |
| HIGUCHI S | 3 | 0.023 |
| HIGUCHI I | 3 | 0.023 |
| HICKESON M | 3 | 0.023 |
| HERRMANN E | 3 | 0.023 |
| HERMANS JJ | 3 | 0.023 |
| HENRY MACD | 3 | 0.023 |
| HENEGHAN HM | 3 | 0.023 |
| HENDLISZ A | 3 | 0.023 |
| HEKMATDOOST A | 3 | 0.023 |
| HEITMANN M | 3 | 0.023 |
| HEIDARI P | 3 | 0.023 |
| HEGEWISCH-BECKER S | 3 | 0.023 |
| HEDNER C | 3 | 0.023 |
| HECHT JR | 3 | 0.023 |
| HEALY LA | 3 | 0.023 |
| HEALY CM | 3 | 0.023 |
| HE XY | 3 | 0.023 |
| HE XP | 3 | 0.023 |
| HE Q | 3 | 0.023 |
| HE N | 3 | 0.023 |
| HE L | 3 | 0.023 |
| HE JX | 3 | 0.023 |
| HE JH | 3 | 0.023 |
| HE BS | 3 | 0.023 |
| HAYASHI S | 3 | 0.023 |
| HAWK E | 3 | 0.023 |
| HAUSS S | 3 | 0.023 |
| HAUG AR | 3 | 0.023 |
| HAUG A | 3 | 0.023 |
| HATTA W | 3 | 0.023 |
| HATAKEYAMA S | 3 | 0.023 |
| HATAKE K | 3 | 0.023 |
| HASHIMOTO N | 3 | 0.023 |
| HASHII H | 3 | 0.023 |
| HASEGAWA M | 3 | 0.023 |
| HASAN S | 3 | 0.023 |
| HARUTA H | 3 | 0.023 |
| HARUKI S | 3 | 0.023 |
| HARTWIG W | 3 | 0.023 |
| HARTGRINK HH | 3 | 0.023 |
| HART AR | 3 | 0.023 |
| HARRIS CL | 3 | 0.023 |
| HAREYAMA M | 3 | 0.023 |
| HARARI PM | 3 | 0.023 |
| HARADA T | 3 | 0.023 |
| HARADA N | 3 | 0.023 |
| HARA H | 3 | 0.023 |
| HAO YT | 3 | 0.023 |
| HAO CY | 3 | 0.023 |
| HANYU T | 3 | 0.023 |
| HANNA MA | 3 | 0.023 |
| HAN ZY | 3 | 0.023 |
| HAN YM | 3 | 0.023 |
| HAN YH | 3 | 0.023 |
| HAN XY | 3 | 0.023 |
| HAN SU | 3 | 0.023 |
| HAN P | 3 | 0.023 |
| HAN LH | 3 | 0.023 |
| HAN B | 3 | 0.023 |
| HAMMOUD Z | 3 | 0.023 |
| HAMAUCHI S | 3 | 0.023 |
| HAMANO R | 3 | 0.023 |
| HAMAJIMA N | 3 | 0.023 |
| HAJDUCH M | 3 | 0.023 |
| HAINSWORTH JD | 3 | 0.023 |
| HAIM N | 3 | 0.023 |
| HAIDER M | 3 | 0.023 |
| HAGA A | 3 | 0.023 |
| HACKERT T | 3 | 0.023 |
| HACHEY KJ | 3 | 0.023 |
| HABR F | 3 | 0.023 |
| GUSANI NJ | 3 | 0.023 |
| GURZU S | 3 | 0.023 |
| GUPTA R | 3 | 0.023 |
| GUPTA D | 3 | 0.023 |
| GUO ZY | 3 | 0.023 |
| GUO YM | 3 | 0.023 |
| GUO XL | 3 | 0.023 |
| GUO XJ | 3 | 0.023 |
| GUO T | 3 | 0.023 |
| GUO SR | 3 | 0.023 |
| GUO QH | 3 | 0.023 |
| GUO LY | 3 | 0.023 |
| GUO LM | 3 | 0.023 |
| GUO K | 3 | 0.023 |
| GUO JW | 3 | 0.023 |
| GUO HQ | 3 | 0.023 |
| GUO HB | 3 | 0.023 |
| GUNDUZ M | 3 | 0.023 |
| GULER S | 3 | 0.023 |
| GUAN X | 3 | 0.023 |
| GUAN QL | 3 | 0.023 |
| GU WD | 3 | 0.023 |
| GU W | 3 | 0.023 |
| GU P | 3 | 0.023 |
| GU L | 3 | 0.023 |
| GU H | 3 | 0.023 |
| GROTH SS | 3 | 0.023 |
| GROBMYER SR | 3 | 0.023 |
| GRIMM M | 3 | 0.023 |
| GRETSCHEL S | 3 | 0.023 |
| GRESS FG | 3 | 0.023 |
| GREER KB | 3 | 0.023 |
| GRECO FA | 3 | 0.023 |
| GRAY J | 3 | 0.023 |
| GRANTZAU T | 3 | 0.023 |
| GRANONE P | 3 | 0.023 |
| GRANDIS JR | 3 | 0.023 |
| GRANDINETTI A | 3 | 0.023 |
| GRABENBAUER GG | 3 | 0.023 |
| GOUMA DJ | 3 | 0.023 |
| GOTOHDA N | 3 | 0.023 |
| GOTO O | 3 | 0.023 |
| GOTO K | 3 | 0.023 |
| GOTO A | 3 | 0.023 |
| GOTAY C | 3 | 0.023 |
| GOSSAGE J | 3 | 0.023 |
| GOSCINSKI MA | 3 | 0.023 |
| GORDON C | 3 | 0.023 |
| GONG Y | 3 | 0.023 |
| GONG M | 3 | 0.023 |
| GONG L | 3 | 0.023 |
| GOMEZ DR | 3 | 0.023 |
| GOLLUB MJ | 3 | 0.023 |
| GOLLINS S | 3 | 0.023 |
| GOLDSTEIN BY | 3 | 0.023 |
| GOLDMAN B | 3 | 0.023 |
| GOLDMAN A | 3 | 0.023 |
| GOLDBLUM J | 3 | 0.023 |
| GOLDBERG RM | 3 | 0.023 |
| GOLD PJ | 3 | 0.023 |
| GOFF LW | 3 | 0.023 |
| GNJATIC S | 3 | 0.023 |
| GLOOR B | 3 | 0.023 |
| GISLESKOG PO | 3 | 0.023 |
| GIRALT J | 3 | 0.023 |
| GINES A | 3 | 0.023 |
| GILLESPIE T | 3 | 0.023 |
| GILLEN S | 3 | 0.023 |
| GILBERT S | 3 | 0.023 |
| GILBERT ES | 3 | 0.023 |
| GIDAY SA | 3 | 0.023 |
| GIBSON M | 3 | 0.023 |
| GIANNARELLI D | 3 | 0.023 |
| GHOLAMIN M | 3 | 0.023 |
| GHOJAZADEH M | 3 | 0.023 |
| GEUSZ ME | 3 | 0.023 |
| GERSON LB | 3 | 0.023 |
| GERMER CT | 3 | 0.023 |
| GERKE H | 3 | 0.023 |
| GERBER R | 3 | 0.023 |
| GERBER PA | 3 | 0.023 |
| GERBAUDO VH | 3 | 0.023 |
| GEORGE J | 3 | 0.023 |
| GEORGE B | 3 | 0.023 |
| GENG Y | 3 | 0.023 |
| GENG GJ | 3 | 0.023 |
| GEDDERT H | 3 | 0.023 |
| GEBOES K | 3 | 0.023 |
| GE X | 3 | 0.023 |
| GE W | 3 | 0.023 |
| GATTERMANN N | 3 | 0.023 |
| GATTER K | 3 | 0.023 |
| GATENBY P | 3 | 0.023 |
| GARG S | 3 | 0.023 |
| GARDIN I | 3 | 0.023 |
| GAO YY | 3 | 0.023 |
| GAO XY | 3 | 0.023 |
| GAO XJ | 3 | 0.023 |
| GAO X | 3 | 0.023 |
| GAO TY | 3 | 0.023 |
| GAO S | 3 | 0.023 |
| GAO R | 3 | 0.023 |
| GAO M | 3 | 0.023 |
| GAO LL | 3 | 0.023 |
| GAO H | 3 | 0.023 |
| GANESHAN B | 3 | 0.023 |
| GANAPATHI AM | 3 | 0.023 |
| GALLEGO J | 3 | 0.023 |
| GALAIS MP | 3 | 0.023 |
| GAGNIERE J | 3 | 0.023 |
| GADDAM S | 3 | 0.023 |
| FURUTA K | 3 | 0.023 |
| FURLONG H | 3 | 0.023 |
| FUNAIOLI C | 3 | 0.023 |
| FUMOTO S | 3 | 0.023 |
| FULLARTON GM | 3 | 0.023 |
| FULLARTON G | 3 | 0.023 |
| FUKUSHIMA S | 3 | 0.023 |
| FUKUSHIMA R | 3 | 0.023 |
| FUKUOKA J | 3 | 0.023 |
| FUKUHARA S | 3 | 0.023 |
| FUKUDA M | 3 | 0.023 |
| FUJIWARA S | 3 | 0.023 |
| FUJIWARA N | 3 | 0.023 |
| FUJIWARA K | 3 | 0.023 |
| FUJITA S | 3 | 0.023 |
| FUJITA J | 3 | 0.023 |
| FUJISAWA K | 3 | 0.023 |
| FUJINAKA Y | 3 | 0.023 |
| FUJIHARA S | 3 | 0.023 |
| FUCHS HF | 3 | 0.023 |
| FUCHS H | 3 | 0.023 |
| FUCCIO L | 3 | 0.023 |
| FU ZM | 3 | 0.023 |
| FU Y | 3 | 0.023 |
| FU X | 3 | 0.023 |
| FU T | 3 | 0.023 |
| FU KI | 3 | 0.023 |
| FROSSARD JL | 3 | 0.023 |
| FRITZ M | 3 | 0.023 |
| FRISTRUP C | 3 | 0.023 |
| FRIEDLAND S | 3 | 0.023 |
| FREIRE J | 3 | 0.023 |
| FRANCOIS E | 3 | 0.023 |
| FRANCIS SR | 3 | 0.023 |
| FRAKES J | 3 | 0.023 |
| FOXWELL T | 3 | 0.023 |
| FOX SB | 3 | 0.023 |
| FOWERS KD | 3 | 0.023 |
| FOURQUET A | 3 | 0.023 |
| FOURNIER KF | 3 | 0.023 |
| FOURNIER C | 3 | 0.023 |
| FOSSA SD | 3 | 0.023 |
| FORONES NM | 3 | 0.023 |
| FONTANA E | 3 | 0.023 |
| FOGLIATA A | 3 | 0.023 |
| FLORES R | 3 | 0.023 |
| FLIEGER D | 3 | 0.023 |
| FLEMING JB | 3 | 0.023 |
| FITZGERALD TL | 3 | 0.023 |
| FISHER DA | 3 | 0.023 |
| FINLEY RJ | 3 | 0.023 |
| FIEDLER W | 3 | 0.023 |
| FIDIAS PM | 3 | 0.023 |
| FIDIAS P | 3 | 0.023 |
| FEUCHTINGER A | 3 | 0.023 |
| FERRARI L | 3 | 0.023 |
| FERNANDES DJ | 3 | 0.023 |
| FENG YB | 3 | 0.023 |
| FENG T | 3 | 0.023 |
| FENG QF | 3 | 0.023 |
| FENG Q | 3 | 0.023 |
| FENG M | 3 | 0.023 |
| FENG JG | 3 | 0.023 |
| FENCL P | 3 | 0.023 |
| FELDMAN LS | 3 | 0.023 |
| FEI ZH | 3 | 0.023 |
| FEARON KCH | 3 | 0.023 |
| FATTORUSO SI | 3 | 0.023 |
| FARIVAR AS | 3 | 0.023 |
| FANG X | 3 | 0.023 |
| FANG WK | 3 | 0.023 |
| FANG W | 3 | 0.023 |
| FANG C | 3 | 0.023 |
| FAN T | 3 | 0.023 |
| FAN KH | 3 | 0.023 |
| FAN J | 3 | 0.023 |
| FAN H | 3 | 0.023 |
| FAN CX | 3 | 0.023 |
| FALLAH M | 3 | 0.023 |
| FALCONE A | 3 | 0.023 |
| FAKHERI H | 3 | 0.023 |
| FAIGEL DO | 3 | 0.023 |
| FAIGEL D | 3 | 0.023 |
| FAHEY PP | 3 | 0.023 |
| FABOZZI T | 3 | 0.023 |
| FABIANOVA E | 3 | 0.023 |
| FABIAN T | 3 | 0.023 |
| EVANS M | 3 | 0.023 |
| EVANS D | 3 | 0.023 |
| ETO H | 3 | 0.023 |
| ESTERMAN A | 3 | 0.023 |
| ESPIN-GARCIA O | 3 | 0.023 |
| ESMAILLZADEH A | 3 | 0.023 |
| ESCH JSA | 3 | 0.023 |
| ESAKI T | 3 | 0.023 |
| ERUSLANOV E | 3 | 0.023 |
| ERHUNMWUNSEE L | 3 | 0.023 |
| ERHARDT A | 3 | 0.023 |
| ERDOGAN A | 3 | 0.023 |
| ERBERSDOBLER A | 3 | 0.023 |
| EMAMI B | 3 | 0.023 |
| EMADI A | 3 | 0.023 |
| ELSTAD NL | 3 | 0.023 |
| EL-ZIMAITY H | 3 | 0.023 |
| EL-NEKEETY AA | 3 | 0.023 |
| EL-KHOUEIRY AB | 3 | 0.023 |
| EL-DEIRY WS | 3 | 0.023 |
| EL NAKADI I | 3 | 0.023 |
| EL GAMMAL AT | 3 | 0.023 |
| EKMAN S | 3 | 0.023 |
| EKINCI O | 3 | 0.023 |
| EJNELL H | 3 | 0.023 |
| EJAZ A | 3 | 0.023 |
| EISTERER W | 3 | 0.023 |
| EHARA K | 3 | 0.023 |
| EGENTER E | 3 | 0.023 |
| EGBERTS JH | 3 | 0.023 |
| EDMUNDOWICZ SA | 3 | 0.023 |
| EBERT MP | 3 | 0.023 |
| EBERHARD J | 3 | 0.023 |
| EBARA T | 3 | 0.023 |
| EASTELL R | 3 | 0.023 |
| EARLE CC | 3 | 0.023 |
| DYE C | 3 | 0.023 |
| DUSEMUND F | 3 | 0.023 |
| DURANCEAU A | 3 | 0.023 |
| DUONG C | 3 | 0.023 |
| DUNN JM | 3 | 0.023 |
| DUMOT J | 3 | 0.023 |
| DUMMER R | 3 | 0.023 |
| DUFFY JP | 3 | 0.023 |
| DUELL EJ | 3 | 0.023 |
| DUAN ZG | 3 | 0.023 |
| DUAN XL | 3 | 0.023 |
| DUA KS | 3 | 0.023 |
| DU XL | 3 | 0.023 |
| DU X | 3 | 0.023 |
| DU RIEU MC | 3 | 0.023 |
| DU L | 3 | 0.023 |
| DU J | 3 | 0.023 |
| DREW PA | 3 | 0.023 |
| DRENCKHAN A | 3 | 0.023 |
| DOYLE LA | 3 | 0.023 |
| DOYEUX K | 3 | 0.023 |
| DOWNEY RJ | 3 | 0.023 |
| DOWLATI A | 3 | 0.023 |
| DOSHI S | 3 | 0.023 |
| DONOVAN JL | 3 | 0.023 |
| DONGPING S | 3 | 0.023 |
| DONG Z | 3 | 0.023 |
| DONG SW | 3 | 0.023 |
| DONG J | 3 | 0.023 |
| DONG H | 3 | 0.023 |
| DONG B | 3 | 0.023 |
| DONAHUE J | 3 | 0.023 |
| DOLAN J | 3 | 0.023 |
| DODBIBA L | 3 | 0.023 |
| DJURIC-STEFANOVIC A | 3 | 0.023 |
| DIZDAR L | 3 | 0.023 |
| DIXIT S | 3 | 0.023 |
| DISARIO JA | 3 | 0.023 |
| DIONISI F | 3 | 0.023 |
| DINIS-RIBEIRO M | 3 | 0.023 |
| DINGLE B | 3 | 0.023 |
| DING XW | 3 | 0.023 |
| DING BG | 3 | 0.023 |
| DILLING TJ | 3 | 0.023 |
| DIAO YT | 3 | 0.023 |
| DIAO DM | 3 | 0.023 |
| DIAMANTIS G | 3 | 0.023 |
| DI TULLIO P | 3 | 0.023 |
| DI SY | 3 | 0.023 |
| DI PIETRO M | 3 | 0.023 |
| DI CARLO S | 3 | 0.023 |
| DEUTSCH M | 3 | 0.023 |
| DENLINGER C | 3 | 0.023 |
| DENG YC | 3 | 0.023 |
| DENG W | 3 | 0.023 |
| DENG HY | 3 | 0.023 |
| DENG HF | 3 | 0.023 |
| DENG GD | 3 | 0.023 |
| DENG B | 3 | 0.023 |
| DENEVE JL | 3 | 0.023 |
| DEMIRCI U | 3 | 0.023 |
| DEMICCO EG | 3 | 0.023 |
| DELVAUX G | 3 | 0.023 |
| DELEYN P | 3 | 0.023 |
| DECKER PA | 3 | 0.023 |
| DECALUWE H | 3 | 0.023 |
| DEARMAN L | 3 | 0.023 |
| DEAN EM | 3 | 0.023 |
| DE VOGELAERE K | 3 | 0.023 |
| DE SALVO GL | 3 | 0.023 |
| DE LA HERA MG | 3 | 0.023 |
| DE LA FUENTE H | 3 | 0.023 |
| DE HAES JCJM | 3 | 0.023 |
| DE BRAUD F | 3 | 0.023 |
| DE BOCK GH | 3 | 0.023 |
| DAWSEY SP | 3 | 0.023 |
| DAVYDOVA J | 3 | 0.023 |
| DAVULURI R | 3 | 0.023 |
| DAVILA M | 3 | 0.023 |
| DAVIES AM | 3 | 0.023 |
| DAVID M | 3 | 0.023 |
| DAUDT AW | 3 | 0.023 |
| DASANU CA | 3 | 0.023 |
| DAR R | 3 | 0.023 |
| DAPRI G | 3 | 0.023 |
| DANENBERG P | 3 | 0.023 |
| DANENBERG K | 3 | 0.023 |
| DAI YD | 3 | 0.023 |
| DAI L | 3 | 0.023 |
| DAI GH | 3 | 0.023 |
| DAI F | 3 | 0.023 |
| DAHLE-SMITH A | 3 | 0.023 |
| DABROWSKI A | 3 | 0.023 |
| D'SOUZA WD | 3 | 0.023 |
| D'HONDT L | 3 | 0.023 |
| CZARNOTA GJ | 3 | 0.023 |
| CUI YH | 3 | 0.023 |
| CUI LL | 3 | 0.023 |
| CUI LH | 3 | 0.023 |
| CUI JF | 3 | 0.023 |
| CROWE SE | 3 | 0.023 |
| CROSS AJ | 3 | 0.023 |
| CRONJE MJ | 3 | 0.023 |
| CRANE SJ | 3 | 0.023 |
| CRABTREE TD | 3 | 0.023 |
| CRABTREE T | 3 | 0.023 |
| COZZI L | 3 | 0.023 |
| COZEN W | 3 | 0.023 |
| COSTANTINI M | 3 | 0.023 |
| COSKUN U | 3 | 0.023 |
| CORRAO G | 3 | 0.023 |
| CORIAT R | 3 | 0.023 |
| COPPES RP | 3 | 0.023 |
| COOPER SL | 3 | 0.023 |
| COOK GJR | 3 | 0.023 |
| CONSTANTINOIU S | 3 | 0.023 |
| CONRAD C | 3 | 0.023 |
| COLSON YL | 3 | 0.023 |
| COEBERGH JWW | 3 | 0.023 |
| COCCOLINI F | 3 | 0.023 |
| COBURN NG | 3 | 0.023 |
| CLIVIO A | 3 | 0.023 |
| CLISANT S | 3 | 0.023 |
| CLARK AB | 3 | 0.023 |
| CIUFFREDA L | 3 | 0.023 |
| CHUTTANI R | 3 | 0.023 |
| CHUNG I | 3 | 0.023 |
| CHUANG TH | 3 | 0.023 |
| CHUANG EY | 3 | 0.023 |
| CHRISTOS PJ | 3 | 0.023 |
| CHOUDHARY A | 3 | 0.023 |
| CHOPRA SS | 3 | 0.023 |
| CHOI YY | 3 | 0.023 |
| CHOI Y | 3 | 0.023 |
| CHOI MK | 3 | 0.023 |
| CHOI JH | 3 | 0.023 |
| CHOI CW | 3 | 0.023 |
| CHOFLET A | 3 | 0.023 |
| CHO SI | 3 | 0.023 |
| CHO JH | 3 | 0.023 |
| CHIU S | 3 | 0.023 |
| CHIU JF | 3 | 0.023 |
| CHIOU TJ | 3 | 0.023 |
| CHIBA H | 3 | 0.023 |
| CHIANG Y | 3 | 0.023 |
| CHIANG SL | 3 | 0.023 |
| CHI BR | 3 | 0.023 |
| CHI A | 3 | 0.023 |
| CHEUNG PY | 3 | 0.023 |
| CHETTY R | 3 | 0.023 |
| CHENNAT J | 3 | 0.023 |
| CHENG XJ | 3 | 0.023 |
| CHENG X | 3 | 0.023 |
| CHENG TH | 3 | 0.023 |
| CHENG R | 3 | 0.023 |
| CHENG GY | 3 | 0.023 |
| CHENG C | 3 | 0.023 |
| CHENG AJ | 3 | 0.023 |
| CHEN ZP | 3 | 0.023 |
| CHEN ZN | 3 | 0.023 |
| CHEN YY | 3 | 0.023 |
| CHEN YQ | 3 | 0.023 |
| CHEN XW | 3 | 0.023 |
| CHEN WW | 3 | 0.023 |
| CHEN WJ | 3 | 0.023 |
| CHEN WF | 3 | 0.023 |
| CHEN TT | 3 | 0.023 |
| CHEN TH | 3 | 0.023 |
| CHEN QJ | 3 | 0.023 |
| CHEN PM | 3 | 0.023 |
| CHEN PH | 3 | 0.023 |
| CHEN MK | 3 | 0.023 |
| CHEN LX | 3 | 0.023 |
| CHEN JM | 3 | 0.023 |
| CHEN HW | 3 | 0.023 |
| CHEN HP | 3 | 0.023 |
| CHEN HL | 3 | 0.023 |
| CHEN HB | 3 | 0.023 |
| CHEN EC | 3 | 0.023 |
| CHEN DY | 3 | 0.023 |
| CHEN CJ | 3 | 0.023 |
| CHE SM | 3 | 0.023 |
| CHAVES DM | 3 | 0.023 |
| CHAVA S | 3 | 0.023 |
| CHAUSSADE S | 3 | 0.023 |
| CHAUHAN SS | 3 | 0.023 |
| CHAUHAN PS | 3 | 0.023 |
| CHASEN M | 3 | 0.023 |
| CHAO Y | 3 | 0.023 |
| CHAO KSC | 3 | 0.023 |
| CHANOCK SJ | 3 | 0.023 |
| CHANG YW | 3 | 0.023 |
| CHANG PH | 3 | 0.023 |
| CHANG FB | 3 | 0.023 |
| CHANG CF | 3 | 0.023 |
| CHAI CY | 3 | 0.023 |
| CESARIO A | 3 | 0.023 |
| CENDAN JC | 3 | 0.023 |
| CEN P | 3 | 0.023 |
| CELLINI F | 3 | 0.023 |
| CELLA D | 3 | 0.023 |
| CELIZ P | 3 | 0.023 |
| CECCARELLI C | 3 | 0.023 |
| CATENA F | 3 | 0.023 |
| CASTILLO R | 3 | 0.023 |
| CASTELLUCCI P | 3 | 0.023 |
| CARTER BW | 3 | 0.023 |
| CARNEIRO F | 3 | 0.023 |
| CARMELLA SG | 3 | 0.023 |
| CAPUSSOTTI L | 3 | 0.023 |
| CAPPELLESSO R | 3 | 0.023 |
| CAPIRCI C | 3 | 0.023 |
| CAPELLO A | 3 | 0.023 |
| CAO YW | 3 | 0.023 |
| CAO YD | 3 | 0.023 |
| CAO W | 3 | 0.023 |
| CAO Q | 3 | 0.023 |
| CAO JL | 3 | 0.023 |
| CAO BW | 3 | 0.023 |
| CAMAJ P | 3 | 0.023 |
| CALVO M | 3 | 0.023 |
| CALPE S | 3 | 0.023 |
| CALATAYUD D | 3 | 0.023 |
| CAI Z | 3 | 0.023 |
| CAI W | 3 | 0.023 |
| CADIERE GB | 3 | 0.023 |
| CACINA C | 3 | 0.023 |
| BYRNES KA | 3 | 0.023 |
| BUYUKBERBER S | 3 | 0.023 |
| BUTTE JM | 3 | 0.023 |
| BUSCH OR | 3 | 0.023 |
| BURROWS WM | 3 | 0.023 |
| BURDETT L | 3 | 0.023 |
| BULLERMAN LB | 3 | 0.023 |
| BUHREN BA | 3 | 0.023 |
| BUFFONI L | 3 | 0.023 |
| BUFFLER P | 3 | 0.023 |
| BUCCI MK | 3 | 0.023 |
| BUBENHEIM M | 3 | 0.023 |
| BRUZZI JF | 3 | 0.023 |
| BRUEWER M | 3 | 0.023 |
| BRUCKNER-TUDERMANN L | 3 | 0.023 |
| BROWN CS | 3 | 0.023 |
| BROWN C | 3 | 0.023 |
| BROWER JV | 3 | 0.023 |
| BROOKS PJ | 3 | 0.023 |
| BROOKS C | 3 | 0.023 |
| BROOKES ST | 3 | 0.023 |
| BRODERICK SR | 3 | 0.023 |
| BRINKMANN S | 3 | 0.023 |
| BRESSEL M | 3 | 0.023 |
| BRENNER H | 3 | 0.023 |
| BRENNAN PJ | 3 | 0.023 |
| BREITHAUPT K | 3 | 0.023 |
| BRAUCHLI P | 3 | 0.023 |
| BRAGG JD | 3 | 0.023 |
| BRADBURY P | 3 | 0.023 |
| BOWMAN ED | 3 | 0.023 |
| BOWEN SR | 3 | 0.023 |
| BOUTELOUP C | 3 | 0.023 |
| BOTROS M | 3 | 0.023 |
| BOTHA AJ | 3 | 0.023 |
| BOTHA A | 3 | 0.023 |
| BOTELHO NK | 3 | 0.023 |
| BOSCH D | 3 | 0.023 |
| BOREIRI M | 3 | 0.023 |
| BOOTZ F | 3 | 0.023 |
| BONI C | 3 | 0.023 |
| BONA D | 3 | 0.023 |
| BOLKE E | 3 | 0.023 |
| BOGGS H | 3 | 0.023 |
| BOELLAARD R | 3 | 0.023 |
| BOEHM KA | 3 | 0.023 |
| BODOKY G | 3 | 0.023 |
| BODNAR A | 3 | 0.023 |
| BO YC | 3 | 0.023 |
| BLOT WJ | 3 | 0.023 |
| BLOCK G | 3 | 0.023 |
| BLOCK A | 3 | 0.023 |
| BLANCHARD P | 3 | 0.023 |
| BLAKE PA | 3 | 0.023 |
| BLACKHAM A | 3 | 0.023 |
| BJORDAL K | 3 | 0.023 |
| BITTINGER M | 3 | 0.023 |
| BISSCHOPS R | 3 | 0.023 |
| BIRRER MJ | 3 | 0.023 |
| BIRK JW | 3 | 0.023 |
| BIRAMIJAMAL F | 3 | 0.023 |
| BILYALOVA Z | 3 | 0.023 |
| BIESTERFELD S | 3 | 0.023 |
| BIANCHINI A | 3 | 0.023 |
| BIAGIOLI M | 3 | 0.023 |
| BI C | 3 | 0.023 |
| BHUTTA HY | 3 | 0.023 |
| BHOOSHAN N | 3 | 0.023 |
| BHATTACHARYA A | 3 | 0.023 |
| BHAT S | 3 | 0.023 |
| BHAT MA | 3 | 0.023 |
| BHARGAVA R | 3 | 0.023 |
| BHARADWAJ M | 3 | 0.023 |
| BERRISFORD R | 3 | 0.023 |
| BERGQVIST M | 3 | 0.023 |
| BERGER B | 3 | 0.023 |
| BERARDI R | 3 | 0.023 |
| BENSON M | 3 | 0.023 |
| BENNINK RJ | 3 | 0.023 |
| BENEKLI M | 3 | 0.023 |
| BENEDIX F | 3 | 0.023 |
| BENEDETTI JK | 3 | 0.023 |
| BENCKO V | 3 | 0.023 |
| BELOHLAVEK O | 3 | 0.023 |
| BELLIZZI AM | 3 | 0.023 |
| BELLER L | 3 | 0.023 |
| BEIJNEN JH | 3 | 0.023 |
| BEHAM A | 3 | 0.023 |
| BECKER V | 3 | 0.023 |
| BECKER T | 3 | 0.023 |
| BECKER H | 3 | 0.023 |
| BECKER C | 3 | 0.023 |
| BAUES C | 3 | 0.023 |
| BAUER K | 3 | 0.023 |
| BATTLEY JE | 3 | 0.023 |
| BATTAFARANO R | 3 | 0.023 |
| BATEMAN AC | 3 | 0.023 |
| BASU A | 3 | 0.023 |
| BASSOTTI G | 3 | 0.023 |
| BASSETTI M | 3 | 0.023 |
| BASSERMANN F | 3 | 0.023 |
| BASHA R | 3 | 0.023 |
| BASELGA J | 3 | 0.023 |
| BASCHNAGEL AM | 3 | 0.023 |
| BARTHOLOMEUSZ D | 3 | 0.023 |
| BARTELS H | 3 | 0.023 |
| BARTALOS C | 3 | 0.023 |
| BARRIUSO J | 3 | 0.023 |
| BARBOUR A | 3 | 0.023 |
| BARANDA JC | 3 | 0.023 |
| BAMIA C | 3 | 0.023 |
| BAMBERG M | 3 | 0.023 |
| BALLUFF B | 3 | 0.023 |
| BALISE RR | 3 | 0.023 |
| BALAZS A | 3 | 0.023 |
| BAJROVIC A | 3 | 0.023 |
| BAJDIK C | 3 | 0.023 |
| BAI XH | 3 | 0.023 |
| BAI W | 3 | 0.023 |
| BAI S | 3 | 0.023 |
| BAGNARDI V | 3 | 0.023 |
| BAGHERI M | 3 | 0.023 |
| BAEK JH | 3 | 0.023 |
| BAE W | 3 | 0.023 |
| BAE SH | 3 | 0.023 |
| BAE JM | 3 | 0.023 |
| BADGWELL BD | 3 | 0.023 |
| BABA E | 3 | 0.023 |
| AZUMA T | 3 | 0.023 |
| AZRIA D | 3 | 0.023 |
| AZAR RR | 3 | 0.023 |
| AZAD AK | 3 | 0.023 |
| AWAD ZT | 3 | 0.023 |
| AUGOFF K | 3 | 0.023 |
| AU HJ | 3 | 0.023 |
| ATAC A | 3 | 0.023 |
| ASSELAIN B | 3 | 0.023 |
| ASSELAH J | 3 | 0.023 |
| ASMIS TR | 3 | 0.023 |
| ASMAR L | 3 | 0.023 |
| ASHLEY SW | 3 | 0.023 |
| ASANO N | 3 | 0.023 |
| ASAISHI K | 3 | 0.023 |
| ARYA S | 3 | 0.023 |
| ARTS D | 3 | 0.023 |
| ARNOLD M | 3 | 0.023 |
| ARKENAU HT | 3 | 0.023 |
| ARIMURA H | 3 | 0.023 |
| ARENA MG | 3 | 0.023 |
| ARDALAN B | 3 | 0.023 |
| ARAUJO JL | 3 | 0.023 |
| ARAI T | 3 | 0.023 |
| ARAI K | 3 | 0.023 |
| ARAGONES N | 3 | 0.023 |
| AOYAMA T | 3 | 0.023 |
| AOYAMA K | 3 | 0.023 |
| AOSASA S | 3 | 0.023 |
| ANTONI D | 3 | 0.023 |
| ANSARI RH | 3 | 0.023 |
| ANSARI R | 3 | 0.023 |
| ANSALONI L | 3 | 0.023 |
| ANGHEL R | 3 | 0.023 |
| ANEGG U | 3 | 0.023 |
| ANDRADE RS | 3 | 0.023 |
| ANDO Y | 3 | 0.023 |
| ANDERSON LA | 3 | 0.023 |
| ANDERLUH F | 3 | 0.023 |
| ANAMI K | 3 | 0.023 |
| AN X | 3 | 0.023 |
| AMANUMA Y | 3 | 0.023 |
| AMANO T | 3 | 0.023 |
| AMADORI D | 3 | 0.023 |
| ALY A | 3 | 0.023 |
| ALTUNTAS B | 3 | 0.023 |
| ALTMANNSBERGER HM | 3 | 0.023 |
| ALLEN NE | 3 | 0.023 |
| ALDIYAROVA G | 3 | 0.023 |
| ALDERSON D | 3 | 0.023 |
| ALDERLIESTEN T | 3 | 0.023 |
| ALDER H | 3 | 0.023 |
| ALBARRACIN CT | 3 | 0.023 |
| ALBANES D | 3 | 0.023 |
| AL-NAHHAS A | 3 | 0.023 |
| AKSOY S | 3 | 0.023 |
| AKIYAMA T | 3 | 0.023 |
| AKIMOTO T | 3 | 0.023 |
| AKIBA S | 3 | 0.023 |
| AKHURST TJ | 3 | 0.023 |
| AKERMAN P | 3 | 0.023 |
| AKBARI M | 3 | 0.023 |
| AKATEH C | 3 | 0.023 |
| AKAGI Y | 3 | 0.023 |
| AHRENS TD | 3 | 0.023 |
| AHN SJ | 3 | 0.023 |
| AHN JH | 3 | 0.023 |
| AGUIRRE ACR | 3 | 0.023 |
| AGRAWAL S | 3 | 0.023 |
| AGRAWAL DK | 3 | 0.023 |
| AGHAYERE O | 3 | 0.023 |
| AGARWAL G | 3 | 0.023 |
| AGAIMY A | 3 | 0.023 |
| ABU DAYYEH BK | 3 | 0.023 |
| ABOAGYE EO | 3 | 0.023 |
| ABDEL-WAHHAB MA | 3 | 0.023 |
| ABBASSI-GHADI N | 3 | 0.023 |
| ABBAS H | 3 | 0.023 |
| AARABI M | 3 | 0.023 |
| ZYCHLA L | 2 | 0.015 |
| ZUO TT | 2 | 0.015 |
| ZUND M | 2 | 0.015 |
| ZUERCHER BF | 2 | 0.015 |
| ZUCCOLO M | 2 | 0.015 |
| ZOU XF | 2 | 0.015 |
| ZOU Q | 2 | 0.015 |
| ZOU J | 2 | 0.015 |
| ZOU H | 2 | 0.015 |
| ZOPHEL K | 2 | 0.015 |
| ZOOLE JB | 2 | 0.015 |
| ZONG Y | 2 | 0.015 |
| ZONG H | 2 | 0.015 |
| ZOMAWIA E | 2 | 0.015 |
| ZOLI W | 2 | 0.015 |
| ZLOBEC I | 2 | 0.015 |
| ZIV-SOKOLOVSKY N | 2 | 0.015 |
| ZITZELSBERGER H | 2 | 0.015 |
| ZINOVYEVA MV | 2 | 0.015 |
| ZINGARETTI C | 2 | 0.015 |
| ZIMMERMANN A | 2 | 0.015 |
| ZIMMERMAN NP | 2 | 0.015 |
| ZILBERMINTS V | 2 | 0.015 |
| ZILBERMAN ST | 2 | 0.015 |
| ZI XL | 2 | 0.015 |
| ZI X | 2 | 0.015 |
| ZHUO WL | 2 | 0.015 |
| ZHUO SM | 2 | 0.015 |
| ZHUGE Y | 2 | 0.015 |
| ZHUANG ZH | 2 | 0.015 |
| ZHUANG Y | 2 | 0.015 |
| ZHU ZG | 2 | 0.015 |
| ZHU ZF | 2 | 0.015 |
| ZHU ZD | 2 | 0.015 |
| ZHU YQ | 2 | 0.015 |
| ZHU YL | 2 | 0.015 |
| ZHU YJ | 2 | 0.015 |
| ZHU YF | 2 | 0.015 |
| ZHU XW | 2 | 0.015 |
| ZHU XR | 2 | 0.015 |
| ZHU XD | 2 | 0.015 |
| ZHU WJ | 2 | 0.015 |
| ZHU TF | 2 | 0.015 |
| ZHU SW | 2 | 0.015 |
| ZHU SL | 2 | 0.015 |
| ZHU MC | 2 | 0.015 |
| ZHU LY | 2 | 0.015 |
| ZHU LH | 2 | 0.015 |
| ZHU L | 2 | 0.015 |
| ZHU JY | 2 | 0.015 |
| ZHU HW | 2 | 0.015 |
| ZHU HT | 2 | 0.015 |
| ZHU GY | 2 | 0.015 |
| ZHU GS | 2 | 0.015 |
| ZHU GJ | 2 | 0.015 |
| ZHU GH | 2 | 0.015 |
| ZHU F | 2 | 0.015 |
| ZHU DX | 2 | 0.015 |
| ZHU CL | 2 | 0.015 |
| ZHOU ZX | 2 | 0.015 |
| ZHOU ZT | 2 | 0.015 |
| ZHOU ZJ | 2 | 0.015 |
| ZHOU YZ | 2 | 0.015 |
| ZHOU YX | 2 | 0.015 |
| ZHOU YH | 2 | 0.015 |
| ZHOU WD | 2 | 0.015 |
| ZHOU SY | 2 | 0.015 |
| ZHOU SN | 2 | 0.015 |
| ZHOU SM | 2 | 0.015 |
| ZHOU SF | 2 | 0.015 |
| ZHOU QZ | 2 | 0.015 |
| ZHOU MH | 2 | 0.015 |
| ZHOU LY | 2 | 0.015 |
| ZHOU LX | 2 | 0.015 |
| ZHOU JW | 2 | 0.015 |
| ZHOU JM | 2 | 0.015 |
| ZHOU JL | 2 | 0.015 |
| ZHOU HL | 2 | 0.015 |
| ZHOU DJ | 2 | 0.015 |
| ZHOU CQ | 2 | 0.015 |
| ZHOU CH | 2 | 0.015 |
| ZHONG Y | 2 | 0.015 |
| ZHONG XY | 2 | 0.015 |
| ZHONG XX | 2 | 0.015 |
| ZHONG R | 2 | 0.015 |
| ZHONG Q | 2 | 0.015 |
| ZHONG M | 2 | 0.015 |
| ZHONG GS | 2 | 0.015 |
| ZHONG GL | 2 | 0.015 |
| ZHONG BL | 2 | 0.015 |
| ZHENG XS | 2 | 0.015 |
| ZHENG XF | 2 | 0.015 |
| ZHENG QQ | 2 | 0.015 |
| ZHENG P | 2 | 0.015 |
| ZHENG MH | 2 | 0.015 |
| ZHENG MF | 2 | 0.015 |
| ZHENG LZ | 2 | 0.015 |
| ZHENG JS | 2 | 0.015 |
| ZHENG DY | 2 | 0.015 |
| ZHENG CL | 2 | 0.015 |
| ZHEN N | 2 | 0.015 |
| ZHE H | 2 | 0.015 |
| ZHAO ZX | 2 | 0.015 |
| ZHAO YZ | 2 | 0.015 |
| ZHAO YX | 2 | 0.015 |
| ZHAO YT | 2 | 0.015 |
| ZHAO YS | 2 | 0.015 |
| ZHAO YL | 2 | 0.015 |
| ZHAO XW | 2 | 0.015 |
| ZHAO WQ | 2 | 0.015 |
| ZHAO WH | 2 | 0.015 |
| ZHAO WC | 2 | 0.015 |
| ZHAO R | 2 | 0.015 |
| ZHAO NM | 2 | 0.015 |
| ZHAO MY | 2 | 0.015 |
| ZHAO LM | 2 | 0.015 |
| ZHAO LH | 2 | 0.015 |
| ZHAO LF | 2 | 0.015 |
| ZHAO JY | 2 | 0.015 |
| ZHAO JQ | 2 | 0.015 |
| ZHAO JB | 2 | 0.015 |
| ZHAO HX | 2 | 0.015 |
| ZHAO HL | 2 | 0.015 |
| ZHAO GH | 2 | 0.015 |
| ZHAO GB | 2 | 0.015 |
| ZHAO DQ | 2 | 0.015 |
| ZHAO DB | 2 | 0.015 |
| ZHAO CH | 2 | 0.015 |
| ZHANG ZP | 2 | 0.015 |
| ZHANG YZ | 2 | 0.015 |
| ZHANG YR | 2 | 0.015 |
| ZHANG YD | 2 | 0.015 |
| ZHANG YC | 2 | 0.015 |
| ZHANG XN | 2 | 0.015 |
| ZHANG XG | 2 | 0.015 |
| ZHANG WW | 2 | 0.015 |
| ZHANG WL | 2 | 0.015 |
| ZHANG WD | 2 | 0.015 |
| ZHANG TH | 2 | 0.015 |
| ZHANG SG | 2 | 0.015 |
| ZHANG QG | 2 | 0.015 |
| ZHANG PF | 2 | 0.015 |
| ZHANG NJ | 2 | 0.015 |
| ZHANG MZ | 2 | 0.015 |
| ZHANG MP | 2 | 0.015 |
| ZHANG LZ | 2 | 0.015 |
| ZHANG LQ | 2 | 0.015 |
| ZHANG LN | 2 | 0.015 |
| ZHANG KJ | 2 | 0.015 |
| ZHANG JZ | 2 | 0.015 |
| ZHANG JT | 2 | 0.015 |
| ZHANG JR | 2 | 0.015 |
| ZHANG JM | 2 | 0.015 |
| ZHANG JG | 2 | 0.015 |
| ZHANG JC | 2 | 0.015 |
| ZHANG HH | 2 | 0.015 |
| ZHANG GX | 2 | 0.015 |
| ZHANG GG | 2 | 0.015 |
| ZHANG FL | 2 | 0.015 |
| ZHANG DX | 2 | 0.015 |
| ZHANG DS | 2 | 0.015 |
| ZHANG DK | 2 | 0.015 |
| ZHANG DH | 2 | 0.015 |
| ZHANG DG | 2 | 0.015 |
| ZHANG DD | 2 | 0.015 |
| ZHANG DC | 2 | 0.015 |
| ZHANG CQ | 2 | 0.015 |
| ZHANG CB | 2 | 0.015 |
| ZHANG BX | 2 | 0.015 |
| ZHANG BH | 2 | 0.015 |
| ZHANG BF | 2 | 0.015 |
| ZHAN LL | 2 | 0.015 |
| ZHAN F | 2 | 0.015 |
| ZHAI YR | 2 | 0.015 |
| ZHAI YC | 2 | 0.015 |
| ZHAI TT | 2 | 0.015 |
| ZHAI K | 2 | 0.015 |
| ZHA WW | 2 | 0.015 |
| ZETTL A | 2 | 0.015 |
| ZERZ A | 2 | 0.015 |
| ZENGIN N | 2 | 0.015 |
| ZENG ZY | 2 | 0.015 |
| ZENG ZC | 2 | 0.015 |
| ZENG YP | 2 | 0.015 |
| ZENG QY | 2 | 0.015 |
| ZENG LP | 2 | 0.015 |
| ZENG B | 2 | 0.015 |
| ZEMAN M | 2 | 0.015 |
| ZELMAN D | 2 | 0.015 |
| ZELEFSKY MJ | 2 | 0.015 |
| ZEKRI JE | 2 | 0.015 |
| ZATOSKIKH V | 2 | 0.015 |
| ZATONSKI W | 2 | 0.015 |
| ZARGAR MA | 2 | 0.015 |
| ZANOVELLO P | 2 | 0.015 |
| ZANIBONI A | 2 | 0.015 |
| ZANG YZ | 2 | 0.015 |
| ZANDER H | 2 | 0.015 |
| ZANDBERG DP | 2 | 0.015 |
| ZAMBON P | 2 | 0.015 |
| ZALUSKI J | 2 | 0.015 |
| ZAKHAROV V | 2 | 0.015 |
| ZAHEDI MJ | 2 | 0.015 |
| ZAGONEL V | 2 | 0.015 |
| ZABOROWSKI A | 2 | 0.015 |
| ZAANAN A | 2 | 0.015 |
| YUSUP G | 2 | 0.015 |
| YUN YX | 2 | 0.015 |
| YUN MJ | 2 | 0.015 |
| YUN JP | 2 | 0.015 |
| YUN J | 2 | 0.015 |
| YUN HL | 2 | 0.015 |
| YUKAWA M | 2 | 0.015 |
| YUEN HF | 2 | 0.015 |
| YUE WM | 2 | 0.015 |
| YUE GGL | 2 | 0.015 |
| YUE DL | 2 | 0.015 |
| YUAN YG | 2 | 0.015 |
| YUAN XS | 2 | 0.015 |
| YUAN XL | 2 | 0.015 |
| YUAN HH | 2 | 0.015 |
| YUAN GY | 2 | 0.015 |
| YU YZ | 2 | 0.015 |
| YU YT | 2 | 0.015 |
| YU YH | 2 | 0.015 |
| YU Y | 2 | 0.015 |
| YU XQ | 2 | 0.015 |
| YU VZ | 2 | 0.015 |
| YU T | 2 | 0.015 |
| YU SF | 2 | 0.015 |
| YU S | 2 | 0.015 |
| YU R | 2 | 0.015 |
| YU QX | 2 | 0.015 |
| YU LF | 2 | 0.015 |
| YU KZ | 2 | 0.015 |
| YU JQ | 2 | 0.015 |
| YU JL | 2 | 0.015 |
| YU JC | 2 | 0.015 |
| YU I | 2 | 0.015 |
| YU HY | 2 | 0.015 |
| YU HS | 2 | 0.015 |
| YU GQ | 2 | 0.015 |
| YU GP | 2 | 0.015 |
| YU FJ | 2 | 0.015 |
| YU DL | 2 | 0.015 |
| YU CP | 2 | 0.015 |
| YOUSIF NG | 2 | 0.015 |
| YOUSEF F | 2 | 0.015 |
| YOUNG PE | 2 | 0.015 |
| YOUNG P | 2 | 0.015 |
| YOU WC | 2 | 0.015 |
| YOU JC | 2 | 0.015 |
| YOSHIZUMI Y | 2 | 0.015 |
| YOSHIZAKI T | 2 | 0.015 |
| YOSHITA H | 2 | 0.015 |
| YOSHIOKA Y | 2 | 0.015 |
| YOSHIOKA M | 2 | 0.015 |
| YOSHIOKA I | 2 | 0.015 |
| YOSHIOKA D | 2 | 0.015 |
| YOSHINO I | 2 | 0.015 |
| YOSHIMURA S | 2 | 0.015 |
| YOSHIMURA H | 2 | 0.015 |
| YOSHIFUKU K | 2 | 0.015 |
| YORKE E | 2 | 0.015 |
| YOON KH | 2 | 0.015 |
| YOON J | 2 | 0.015 |
| YOON HY | 2 | 0.015 |
| YOOK JI | 2 | 0.015 |
| YOO S | 2 | 0.015 |
| YOO IR | 2 | 0.015 |
| YOO HM | 2 | 0.015 |
| YOO C | 2 | 0.015 |
| YOKOYAMA Y | 2 | 0.015 |
| YOKOMAKURA N | 2 | 0.015 |
| YOKOI S | 2 | 0.015 |
| YOKOI C | 2 | 0.015 |
| YNGVE A | 2 | 0.015 |
| YIP SSF | 2 | 0.015 |
| YING SM | 2 | 0.015 |
| YIN XS | 2 | 0.015 |
| YIN X | 2 | 0.015 |
| YIN WB | 2 | 0.015 |
| YIN M | 2 | 0.015 |
| YIN L | 2 | 0.015 |
| YIN F | 2 | 0.015 |
| YIN CJ | 2 | 0.015 |
| YILMAZ A | 2 | 0.015 |
| YILDIZ I | 2 | 0.015 |
| YI YF | 2 | 0.015 |
| YI SW | 2 | 0.015 |
| YERIAN L | 2 | 0.015 |
| YEREB M | 2 | 0.015 |
| YEOH KG | 2 | 0.015 |
| YEO CJ | 2 | 0.015 |
| YEN T | 2 | 0.015 |
| YELENSKY R | 2 | 0.015 |
| YEH TS | 2 | 0.015 |
| YEH CY | 2 | 0.015 |
| YEH CT | 2 | 0.015 |
| YE YW | 2 | 0.015 |
| YE YJ | 2 | 0.015 |
| YE XB | 2 | 0.015 |
| YE X | 2 | 0.015 |
| YAZUMI S | 2 | 0.015 |
| YAZICI O | 2 | 0.015 |
| YAZDANI N | 2 | 0.015 |
| YAU T | 2 | 0.015 |
| YASUMOTO K | 2 | 0.015 |
| YASUI Y | 2 | 0.015 |
| YASUFUKU K | 2 | 0.015 |
| YASUDA S | 2 | 0.015 |
| YASUDA M | 2 | 0.015 |
| YASHIMA K | 2 | 0.015 |
| YASERI M | 2 | 0.015 |
| YAREMKO BP | 2 | 0.015 |
| YAO YW | 2 | 0.015 |
| YAO YG | 2 | 0.015 |
| YAO YC | 2 | 0.015 |
| YAO T | 2 | 0.015 |
| YAO HR | 2 | 0.015 |
| YAO H | 2 | 0.015 |
| YAO GH | 2 | 0.015 |
| YANG ZS | 2 | 0.015 |
| YANG ZQ | 2 | 0.015 |
| YANG ZM | 2 | 0.015 |
| YANG ZD | 2 | 0.015 |
| YANG ZB | 2 | 0.015 |
| YANG YY | 2 | 0.015 |
| YANG YT | 2 | 0.015 |
| YANG YC | 2 | 0.015 |
| YANG XZ | 2 | 0.015 |
| YANG XX | 2 | 0.015 |
| YANG XW | 2 | 0.015 |
| YANG XQ | 2 | 0.015 |
| YANG XL | 2 | 0.015 |
| YANG XD | 2 | 0.015 |
| YANG WP | 2 | 0.015 |
| YANG WL | 2 | 0.015 |
| YANG TX | 2 | 0.015 |
| YANG T | 2 | 0.015 |
| YANG SH | 2 | 0.015 |
| YANG R | 2 | 0.015 |
| YANG QH | 2 | 0.015 |
| YANG QF | 2 | 0.015 |
| YANG LW | 2 | 0.015 |
| YANG LP | 2 | 0.015 |
| YANG LG | 2 | 0.015 |
| YANG LC | 2 | 0.015 |
| YANG KH | 2 | 0.015 |
| YANG JY | 2 | 0.015 |
| YANG JX | 2 | 0.015 |
| YANG JW | 2 | 0.015 |
| YANG JR | 2 | 0.015 |
| YANG JQ | 2 | 0.015 |
| YANG JL | 2 | 0.015 |
| YANG HF | 2 | 0.015 |
| YANG HB | 2 | 0.015 |
| YANG GR | 2 | 0.015 |
| YANG GH | 2 | 0.015 |
| YANG ES | 2 | 0.015 |
| YANG DH | 2 | 0.015 |
| YANG CW | 2 | 0.015 |
| YANG CM | 2 | 0.015 |
| YANG CH | 2 | 0.015 |
| YANG AK | 2 | 0.015 |
| YANAGIDA N | 2 | 0.015 |
| YANAGI T | 2 | 0.015 |
| YANAGAWA M | 2 | 0.015 |
| YAN XW | 2 | 0.015 |
| YAN W | 2 | 0.015 |
| YAN T | 2 | 0.015 |
| YAN R | 2 | 0.015 |
| YAN JJ | 2 | 0.015 |
| YAN HW | 2 | 0.015 |
| YAN HJ | 2 | 0.015 |
| YAN B | 2 | 0.015 |
| YAMAZAKI T | 2 | 0.015 |
| YAMAWAKI H | 2 | 0.015 |
| YAMAURA T | 2 | 0.015 |
| YAMATO I | 2 | 0.015 |
| YAMANOUCHI T | 2 | 0.015 |
| YAMANI NM | 2 | 0.015 |
| YAMANAKA Y | 2 | 0.015 |
| YAMANA N | 2 | 0.015 |
| YAMAMURA Y | 2 | 0.015 |
| YAMAMURA T | 2 | 0.015 |
| YAMAMURA M | 2 | 0.015 |
| YAMAMURA K | 2 | 0.015 |
| YAMAMORI M | 2 | 0.015 |
| YAMADE M | 2 | 0.015 |
| YALCIN B | 2 | 0.015 |
| YAJIMA K | 2 | 0.015 |
| YAGISHITA A | 2 | 0.015 |
| YAGI Y | 2 | 0.015 |
| YAGI S | 2 | 0.015 |
| YACOUB J | 2 | 0.015 |
| YACHIDA S | 2 | 0.015 |
| YABUUCHI H | 2 | 0.015 |
| XUN TR | 2 | 0.015 |
| XUE Y | 2 | 0.015 |
| XUE XY | 2 | 0.015 |
| XUE JX | 2 | 0.015 |
| XUE HC | 2 | 0.015 |
| XUAN Y | 2 | 0.015 |
| XUAN XY | 2 | 0.015 |
| XU ZY | 2 | 0.015 |
| XU ZL | 2 | 0.015 |
| XU YT | 2 | 0.015 |
| XU XM | 2 | 0.015 |
| XU XE | 2 | 0.015 |
| XU WT | 2 | 0.015 |
| XU WH | 2 | 0.015 |
| XU WD | 2 | 0.015 |
| XU TZ | 2 | 0.015 |
| XU ST | 2 | 0.015 |
| XU SP | 2 | 0.015 |
| XU QR | 2 | 0.015 |
| XU MY | 2 | 0.015 |
| XU MF | 2 | 0.015 |
| XU LX | 2 | 0.015 |
| XU LH | 2 | 0.015 |
| XU JY | 2 | 0.015 |
| XU JL | 2 | 0.015 |
| XU JH | 2 | 0.015 |
| XU GF | 2 | 0.015 |
| XU GC | 2 | 0.015 |
| XU DF | 2 | 0.015 |
| XU CW | 2 | 0.015 |
| XU CQ | 2 | 0.015 |
| XIU X | 2 | 0.015 |
| XIONG XY | 2 | 0.015 |
| XIONG WL | 2 | 0.015 |
| XIONG HC | 2 | 0.015 |
| XIONG H | 2 | 0.015 |
| XIONG F | 2 | 0.015 |
| XING XZ | 2 | 0.015 |
| XING HX | 2 | 0.015 |
| XIE ZY | 2 | 0.015 |
| XIE ZJ | 2 | 0.015 |
| XIE YE | 2 | 0.015 |
| XIE XX | 2 | 0.015 |
| XIE WH | 2 | 0.015 |
| XIE W | 2 | 0.015 |
| XIE SS | 2 | 0.015 |
| XIE SP | 2 | 0.015 |
| XIE MR | 2 | 0.015 |
| XIE JW | 2 | 0.015 |
| XIE F | 2 | 0.015 |
| XIE DR | 2 | 0.015 |
| XIE CH | 2 | 0.015 |
| XIAO ZY | 2 | 0.015 |
| XIAO YJ | 2 | 0.015 |
| XIAO W | 2 | 0.015 |
| XIAO SW | 2 | 0.015 |
| XIAO HL | 2 | 0.015 |
| XIAO F | 2 | 0.015 |
| XIAO DY | 2 | 0.015 |
| XIAO DJ | 2 | 0.015 |
| XIAO B | 2 | 0.015 |
| XIANG SS | 2 | 0.015 |
| XIANG M | 2 | 0.015 |
| XIA ZK | 2 | 0.015 |
| XIA YB | 2 | 0.015 |
| XIA WL | 2 | 0.015 |
| XIA TS | 2 | 0.015 |
| XIA QY | 2 | 0.015 |
| XIA L | 2 | 0.015 |
| XIA JF | 2 | 0.015 |
| XIA CQ | 2 | 0.015 |
| XI Z | 2 | 0.015 |
| XI Y | 2 | 0.015 |
| XI SC | 2 | 0.015 |
| WYSOWSKI DK | 2 | 0.015 |
| WYRWICZ L | 2 | 0.015 |
| WYKYPIEL H | 2 | 0.015 |
| WUTHRICK EJ | 2 | 0.015 |
| WU ZQ | 2 | 0.015 |
| WU ZM | 2 | 0.015 |
| WU YT | 2 | 0.015 |
| WU YJ | 2 | 0.015 |
| WU YG | 2 | 0.015 |
| WU XW | 2 | 0.015 |
| WU XR | 2 | 0.015 |
| WU XN | 2 | 0.015 |
| WU XM | 2 | 0.015 |
| WU XB | 2 | 0.015 |
| WU WT | 2 | 0.015 |
| WU WJ | 2 | 0.015 |
| WU WD | 2 | 0.015 |
| WU WB | 2 | 0.015 |
| WU SW | 2 | 0.015 |
| WU SQ | 2 | 0.015 |
| WU S | 2 | 0.015 |
| WU PP | 2 | 0.015 |
| WU MX | 2 | 0.015 |
| WU KQ | 2 | 0.015 |
| WU JW | 2 | 0.015 |
| WU JQ | 2 | 0.015 |
| WU JM | 2 | 0.015 |
| WU JL | 2 | 0.015 |
| WU HC | 2 | 0.015 |
| WU GZ | 2 | 0.015 |
| WU GL | 2 | 0.015 |
| WU CS | 2 | 0.015 |
| WU BQ | 2 | 0.015 |
| WRIGHT FC | 2 | 0.015 |
| WORMALD JCR | 2 | 0.015 |
| WORHUNSKY DJ | 2 | 0.015 |
| WOODWARD M | 2 | 0.015 |
| WOO M | 2 | 0.015 |
| WONG KY | 2 | 0.015 |
| WONG KK | 2 | 0.015 |
| WONG JY | 2 | 0.015 |
| WONG H | 2 | 0.015 |
| WONG G | 2 | 0.015 |
| WONG FH | 2 | 0.015 |
| WONG C | 2 | 0.015 |
| WONG BCY | 2 | 0.015 |
| WONG AT | 2 | 0.015 |
| WON E | 2 | 0.015 |
| WOLTERS J | 2 | 0.015 |
| WOLLNER M | 2 | 0.015 |
| WOLFF RA | 2 | 0.015 |
| WOLFF R | 2 | 0.015 |
| WOLFENSBERGER P | 2 | 0.015 |
| WOLF WA | 2 | 0.015 |
| WOJTYS M | 2 | 0.015 |
| WOJCIK N | 2 | 0.015 |
| WOJCIK J | 2 | 0.015 |
| WOJCIESZYNSKI A | 2 | 0.015 |
| WITKOWSKI ER | 2 | 0.015 |
| WITEK M | 2 | 0.015 |
| WISSMEYER M | 2 | 0.015 |
| WIRTALLA C | 2 | 0.015 |
| WINTER H | 2 | 0.015 |
| WINSLET MC | 2 | 0.015 |
| WINSLET M | 2 | 0.015 |
| WINOKUR TS | 2 | 0.015 |
| WINKLER M | 2 | 0.015 |
| WINK JC | 2 | 0.015 |
| WINDER T | 2 | 0.015 |
| WILSON RH | 2 | 0.015 |
| WILSON PM | 2 | 0.015 |
| WILLS J | 2 | 0.015 |
| WILLINGHAM FF | 2 | 0.015 |
| WILLIAMS TM | 2 | 0.015 |
| WILLIAMS S | 2 | 0.015 |
| WILLIAMS RN | 2 | 0.015 |
| WILLIAMS R | 2 | 0.015 |
| WILLIAMS LA | 2 | 0.015 |
| WILLIAMS GV | 2 | 0.015 |
| WILLIAMS DG | 2 | 0.015 |
| WILLERT R | 2 | 0.015 |
| WILKINSON B | 2 | 0.015 |
| WILKENS JJ | 2 | 0.015 |
| WIJNHOVEN B | 2 | 0.015 |
| WIJETUNGE S | 2 | 0.015 |
| WIGGINS CL | 2 | 0.015 |
| WIEGEL T | 2 | 0.015 |
| WIEDENMANN B | 2 | 0.015 |
| WIECHMANN V | 2 | 0.015 |
| WIECHA C | 2 | 0.015 |
| WIDDER J | 2 | 0.015 |
| WICKRAMASINGHE K | 2 | 0.015 |
| WIAZZANE N | 2 | 0.015 |
| WHITSON BA | 2 | 0.015 |
| WHITEMAN D | 2 | 0.015 |
| WHISTANCE RN | 2 | 0.015 |
| WHELER JJ | 2 | 0.015 |
| WEUSTEN BL | 2 | 0.015 |
| WESTRA W | 2 | 0.015 |
| WENTZEL-LARSEN T | 2 | 0.015 |
| WENG YL | 2 | 0.015 |
| WENG HR | 2 | 0.015 |
| WENG DS | 2 | 0.015 |
| WENCZL M | 2 | 0.015 |
| WEN XM | 2 | 0.015 |
| WEN XD | 2 | 0.015 |
| WEN SW | 2 | 0.015 |
| WEN JG | 2 | 0.015 |
| WEN FB | 2 | 0.015 |
| WEN C | 2 | 0.015 |
| WELLS J | 2 | 0.015 |
| WELCH NT | 2 | 0.015 |
| WELCH J | 2 | 0.015 |
| WELCH I | 2 | 0.015 |
| WELCH A | 2 | 0.015 |
| WEITZ J | 2 | 0.015 |
| WEISSINGER F | 2 | 0.015 |
| WEISS J | 2 | 0.015 |
| WEISHAUPT D | 2 | 0.015 |
| WEISENBURGER DD | 2 | 0.015 |
| WEIS J | 2 | 0.015 |
| WEINSTEIN GS | 2 | 0.015 |
| WEINER JP | 2 | 0.015 |
| WEINDELMAYER J | 2 | 0.015 |
| WEINBERG DS | 2 | 0.015 |
| WEIMER M | 2 | 0.015 |
| WEIDLING E | 2 | 0.015 |
| WEIDENHAGEN R | 2 | 0.015 |
| WEI XL | 2 | 0.015 |
| WEI WI | 2 | 0.015 |
| WEI S | 2 | 0.015 |
| WEI M | 2 | 0.015 |
| WEI LY | 2 | 0.015 |
| WEI B | 2 | 0.015 |
| WEHRMANN T | 2 | 0.015 |
| WEERSMA R | 2 | 0.015 |
| WEBER C | 2 | 0.015 |
| WEAVER DT | 2 | 0.015 |
| WAY LW | 2 | 0.015 |
| WATTS NB | 2 | 0.015 |
| WATSON P | 2 | 0.015 |
| WATKINS JM | 2 | 0.015 |
| WATKINS D | 2 | 0.015 |
| WATARAI Y | 2 | 0.015 |
| WATANABE O | 2 | 0.015 |
| WATABE T | 2 | 0.015 |
| WASHINGTON K | 2 | 0.015 |
| WARTH B | 2 | 0.015 |
| WARREN S | 2 | 0.015 |
| WARREN G | 2 | 0.015 |
| WARNER N | 2 | 0.015 |
| WAREHAM NJ | 2 | 0.015 |
| WARD K | 2 | 0.015 |
| WARD EM | 2 | 0.015 |
| WARAYA M | 2 | 0.015 |
| WANI KA | 2 | 0.015 |
| WANG-GILLAM A | 2 | 0.015 |
| WANG ZS | 2 | 0.015 |
| WANG ZK | 2 | 0.015 |
| WANG ZB | 2 | 0.015 |
| WANG YW | 2 | 0.015 |
| WANG XQ | 2 | 0.015 |
| WANG XN | 2 | 0.015 |
| WANG XG | 2 | 0.015 |
| WANG XC | 2 | 0.015 |
| WANG WZ | 2 | 0.015 |
| WANG WH | 2 | 0.015 |
| WANG WG | 2 | 0.015 |
| WANG WB | 2 | 0.015 |
| WANG TH | 2 | 0.015 |
| WANG SZ | 2 | 0.015 |
| WANG SY | 2 | 0.015 |
| WANG SM | 2 | 0.015 |
| WANG RB | 2 | 0.015 |
| WANG QQ | 2 | 0.015 |
| WANG QJ | 2 | 0.015 |
| WANG QA | 2 | 0.015 |
| WANG PL | 2 | 0.015 |
| WANG MW | 2 | 0.015 |
| WANG LQ | 2 | 0.015 |
| WANG LE | 2 | 0.015 |
| WANG KP | 2 | 0.015 |
| WANG KL | 2 | 0.015 |
| WANG JX | 2 | 0.015 |
| WANG JR | 2 | 0.015 |
| WANG HW | 2 | 0.015 |
| WANG HS | 2 | 0.015 |
| WANG HK | 2 | 0.015 |
| WANG HC | 2 | 0.015 |
| WANG HA | 2 | 0.015 |
| WANG GP | 2 | 0.015 |
| WANG FY | 2 | 0.015 |
| WANG FL | 2 | 0.015 |
| WANG DW | 2 | 0.015 |
| WANG DS | 2 | 0.015 |
| WANG CX | 2 | 0.015 |
| WANG CKC | 2 | 0.015 |
| WANG CD | 2 | 0.015 |
| WANG BX | 2 | 0.015 |
| WANG BR | 2 | 0.015 |
| WANG AZ | 2 | 0.015 |
| WANG AY | 2 | 0.015 |
| WANG AQ | 2 | 0.015 |
| WANG A | 2 | 0.015 |
| WANDHOFER C | 2 | 0.015 |
| WAN MZ | 2 | 0.015 |
| WAN F | 2 | 0.015 |
| WAN BL | 2 | 0.015 |
| WALTHER LE | 2 | 0.015 |
| WALTERS DM | 2 | 0.015 |
| WALTER D | 2 | 0.015 |
| WALSH G | 2 | 0.015 |
| WALLNER G | 2 | 0.015 |
| WALLE T | 2 | 0.015 |
| WALL TL | 2 | 0.015 |
| WALKER JP | 2 | 0.015 |
| WALKER J | 2 | 0.015 |
| WALKER G | 2 | 0.015 |
| WALDMAN SA | 2 | 0.015 |
| WALCH AK | 2 | 0.015 |
| WAKITA T | 2 | 0.015 |
| WAKEFIELD J | 2 | 0.015 |
| WAKABAYASHI T | 2 | 0.015 |
| WAKABAYASHI H | 2 | 0.015 |
| WAGNER U | 2 | 0.015 |
| WADE JL | 2 | 0.015 |
| WADA Y | 2 | 0.015 |
| WACHOWIAK R | 2 | 0.015 |
| WABINGA H | 2 | 0.015 |
| VYVERBERG A | 2 | 0.015 |
| VUILLEUMIER H | 2 | 0.015 |
| VRADELIS S | 2 | 0.015 |
| VOTANOPOULOS KI | 2 | 0.015 |
| VOTANOPOULOS K | 2 | 0.015 |
| VOSMIK M | 2 | 0.015 |
| VONCKEN FEM | 2 | 0.015 |
| VON GALL C | 2 | 0.015 |
| VON DEIMLING A | 2 | 0.015 |
| VOLKWEIS BS | 2 | 0.015 |
| VOIDAZAN S | 2 | 0.015 |
| VOGTMANN E | 2 | 0.015 |
| VOCK J | 2 | 0.015 |
| VLAD RM | 2 | 0.015 |
| VIVEKANANDAN N | 2 | 0.015 |
| VISWANATHAN C | 2 | 0.015 |
| VISSER E | 2 | 0.015 |
| VISSER B | 2 | 0.015 |
| VISSCHER A | 2 | 0.015 |
| VISBAL A | 2 | 0.015 |
| VIRTAMO J | 2 | 0.015 |
| VIRGILI N | 2 | 0.015 |
| VINOGRADOVA Y | 2 | 0.015 |
| VILLENEUVE PJ | 2 | 0.015 |
| VILJOEN A | 2 | 0.015 |
| VILGELM AE | 2 | 0.015 |
| VILCEA ID | 2 | 0.015 |
| VILCEA AM | 2 | 0.015 |
| VIKRAMAN S | 2 | 0.015 |
| VIJAPURA C | 2 | 0.015 |
| VIGANO L | 2 | 0.015 |
| VIDYASAGAR MS | 2 | 0.015 |
| VIDMAR MS | 2 | 0.015 |
| VIDETIC G | 2 | 0.015 |
| VICI P | 2 | 0.015 |
| VETTOR R | 2 | 0.015 |
| VESSELS B | 2 | 0.015 |
| VESELINOVIC M | 2 | 0.015 |
| VERONESE N | 2 | 0.015 |
| VERNAT SS | 2 | 0.015 |
| VERMA V | 2 | 0.015 |
| VERLATO G | 2 | 0.015 |
| VERHOEF CC | 2 | 0.015 |
| VERHOEF C | 2 | 0.015 |
| VERHEIJ M | 2 | 0.015 |
| VERAS IM | 2 | 0.015 |
| VENNETTILLI A | 2 | 0.015 |
| VELTZKE-SCHLIEKER W | 2 | 0.015 |
| VEITS L | 2 | 0.015 |
| VEIT-HAIBACH P | 2 | 0.015 |
| VEHLING-KAISER U | 2 | 0.015 |
| VEENHOF AAFA | 2 | 0.015 |
| VAZQUEZ-ARAUJO L | 2 | 0.015 |
| VAZ GM | 2 | 0.015 |
| VASUDEVAN M | 2 | 0.015 |
| VASSON MP | 2 | 0.015 |
| VASILEVSKA-RISTOVSKA J | 2 | 0.015 |
| VASILE I | 2 | 0.015 |
| VASILE E | 2 | 0.015 |
| VASHI P | 2 | 0.015 |
| VARGHESE S | 2 | 0.015 |
| VARGA G | 2 | 0.015 |
| VARDHANABHUTI V | 2 | 0.015 |
| VARAYIL JE | 2 | 0.015 |
| VANNESTE B | 2 | 0.015 |
| VANDRIS K | 2 | 0.015 |
| VANDERPUYE V | 2 | 0.015 |
| VANDER BORGHT T | 2 | 0.015 |
| VAN WORKUM F | 2 | 0.015 |
| VAN WOERKOM JM | 2 | 0.015 |
| VAN WERVEN JR | 2 | 0.015 |
| VAN VILSTEREN FGI | 2 | 0.015 |
| VAN VEEN EM | 2 | 0.015 |
| VAN TINTEREN H | 2 | 0.015 |
| VAN STEENBERGEN LN | 2 | 0.015 |
| VAN SCHOOTEN FJ | 2 | 0.015 |
| VAN RENSBURG BJ | 2 | 0.015 |
| VAN PUTTEN M | 2 | 0.015 |
| VAN OS RM | 2 | 0.015 |
| VAN NESTE L | 2 | 0.015 |
| VAN MEERTEN E | 2 | 0.015 |
| VAN LIER A | 2 | 0.015 |
| VAN LEEUWEN TG | 2 | 0.015 |
| VAN LEEUWEN PAM | 2 | 0.015 |
| VAN LANSCHOT J | 2 | 0.015 |
| VAN HEEL NCM | 2 | 0.015 |
| VAN HAAREN PMA | 2 | 0.015 |
| VAN GENDEREN M | 2 | 0.015 |
| VAN EYCKEN E | 2 | 0.015 |
| VAN DUIN M | 2 | 0.015 |
| VAN DRIEL OJR | 2 | 0.015 |
| VAN DIJK S | 2 | 0.015 |
| VAN DIEST PJ | 2 | 0.015 |
| VAN DET MJ | 2 | 0.015 |
| VAN DER VLIET HJ | 2 | 0.015 |
| VAN DER TWEEL I | 2 | 0.015 |
| VAN DER SANGEN MJ | 2 | 0.015 |
| VAN DER SANGEN M | 2 | 0.015 |
| VAN DER RIJT CCD | 2 | 0.015 |
| VAN DER MEIJ BS | 2 | 0.015 |
| VAN DER HOEK M | 2 | 0.015 |
| VAN DER GRAAF WTA | 2 | 0.015 |
| VAN DEN HOFF J | 2 | 0.015 |
| VAN DEN BERG MW | 2 | 0.015 |
| VAN DEN BERG C | 2 | 0.015 |
| VAN DE VIJVER MJ | 2 | 0.015 |
| VAN DE PUTTE D | 2 | 0.015 |
| VAN BLANKENSTEIN M | 2 | 0.015 |
| VAN BALLEGOOIJEN M | 2 | 0.015 |
| VAN BAAL JW | 2 | 0.015 |
| VALVERE V | 2 | 0.015 |
| VALMASONI M | 2 | 0.015 |
| VALLBOEHMER D | 2 | 0.015 |
| VALKENET K | 2 | 0.015 |
| VALIEVA S | 2 | 0.015 |
| VALENTINI V | 2 | 0.015 |
| VALEAN S | 2 | 0.015 |
| VAKIL N | 2 | 0.015 |
| VAKEVAINEN S | 2 | 0.015 |
| VAIPHEI K | 2 | 0.015 |
| VAALAVIRTA L | 2 | 0.015 |
| UZZAU A | 2 | 0.015 |
| UZUNOGLU G | 2 | 0.015 |
| UZANA R | 2 | 0.015 |
| UTSUNOMIYA T | 2 | 0.015 |
| UTSUNOMIYA S | 2 | 0.015 |
| USAMI M | 2 | 0.015 |
| URSACHE E | 2 | 0.015 |
| URONIS HE | 2 | 0.015 |
| URBAN MI | 2 | 0.015 |
| URBA SC | 2 | 0.015 |
| URASHIMA M | 2 | 0.015 |
| URAOKA N | 2 | 0.015 |
| URANO S | 2 | 0.015 |
| URAMOTO H | 2 | 0.015 |
| URABE S | 2 | 0.015 |
| UPPOT RN | 2 | 0.015 |
| UNO F | 2 | 0.015 |
| UNNO N | 2 | 0.015 |
| UNAL OU | 2 | 0.015 |
| UMEZAWA A | 2 | 0.015 |
| UMEOKA S | 2 | 0.015 |
| UMEMURA T | 2 | 0.015 |
| UMEHARA S | 2 | 0.015 |
| ULRICH A | 2 | 0.015 |
| ULLERICH H | 2 | 0.015 |
| ULLA M | 2 | 0.015 |
| UJIKI M | 2 | 0.015 |
| UHLEN M | 2 | 0.015 |
| UGUR VI | 2 | 0.015 |
| UENO N | 2 | 0.015 |
| UENAKA M | 2 | 0.015 |
| UEKI N | 2 | 0.015 |
| UEFFING M | 2 | 0.015 |
| UEDA T | 2 | 0.015 |
| UDREA AA | 2 | 0.015 |
| UDDIN S | 2 | 0.015 |
| UCHIYAMA Y | 2 | 0.015 |
| UCHINAMI Y | 2 | 0.015 |
| UCHIDA N | 2 | 0.015 |
| UCHI Y | 2 | 0.015 |
| UCHI R | 2 | 0.015 |
| UBHI SS | 2 | 0.015 |
| UBBINK DT | 2 | 0.015 |
| TWAROCK S | 2 | 0.015 |
| TWADDELL WS | 2 | 0.015 |
| TUTTLE TM | 2 | 0.015 |
| TURNER A | 2 | 0.015 |
| TURGHUN A | 2 | 0.015 |
| TURECI O | 2 | 0.015 |
| TURAN S | 2 | 0.015 |
| TURAGA K | 2 | 0.015 |
| TUNG LN | 2 | 0.015 |
| TUNG EC | 2 | 0.015 |
| TUNCER M | 2 | 0.015 |
| TUEBERGEN D | 2 | 0.015 |
| TU HP | 2 | 0.015 |
| TU CH | 2 | 0.015 |
| TSUTSUMI S | 2 | 0.015 |
| TSUTSUI S | 2 | 0.015 |
| TSUTANI Y | 2 | 0.015 |
| TSURUTA S | 2 | 0.015 |
| TSURUMARU D | 2 | 0.015 |
| TSUKIYAMA I | 2 | 0.015 |
| TSUKAGOSHI H | 2 | 0.015 |
| TSUKADA J | 2 | 0.015 |
| TSUJIURA M | 2 | 0.015 |
| TSUJITA E | 2 | 0.015 |
| TSUJIMURA H | 2 | 0.015 |
| TSUJII H | 2 | 0.015 |
| TSUJIE M | 2 | 0.015 |
| TSUI TY | 2 | 0.015 |
| TSUCHIHARA K | 2 | 0.015 |
| TSUCHIDA E | 2 | 0.015 |
| TSOURLAKIS MC | 2 | 0.015 |
| TSENG CH | 2 | 0.015 |
| TSE A | 2 | 0.015 |
| TSAO GSW | 2 | 0.015 |
| TSAI YS | 2 | 0.015 |
| TSAI ST | 2 | 0.015 |
| TSAI SJ | 2 | 0.015 |
| TSAI PY | 2 | 0.015 |
| TSAI MH | 2 | 0.015 |
| TSAI JC | 2 | 0.015 |
| TSAI FC | 2 | 0.015 |
| TSAI CH | 2 | 0.015 |
| TSAI C | 2 | 0.015 |
| TRYAKIN A | 2 | 0.015 |
| TRUONG P | 2 | 0.015 |
| TRUMM C | 2 | 0.015 |
| TRUDGILL NJ | 2 | 0.015 |
| TRUDEL JG | 2 | 0.015 |
| TRUC G | 2 | 0.015 |
| TRIVERS KF | 2 | 0.015 |
| TRIPODIS Y | 2 | 0.015 |
| TRINH TT | 2 | 0.015 |
| TREVELLIN E | 2 | 0.015 |
| TREMBLAY L | 2 | 0.015 |
| TRECKMANN JW | 2 | 0.015 |
| TREANOR D | 2 | 0.015 |
| TRAPPENBURG JCA | 2 | 0.015 |
| TRAN KTC | 2 | 0.015 |
| TRAN B | 2 | 0.015 |
| TOYAMA E | 2 | 0.015 |
| TOWNLEY P | 2 | 0.015 |
| TOUILLAUD M | 2 | 0.015 |
| TOTSUKA Y | 2 | 0.015 |
| TOTH E | 2 | 0.015 |
| TOSHIKAWA C | 2 | 0.015 |
| TORRE LA | 2 | 0.015 |
| TORII K | 2 | 0.015 |
| TORIGIAN DA | 2 | 0.015 |
| TOPPO L | 2 | 0.015 |
| TOPPLER G | 2 | 0.015 |
| TOPP SA | 2 | 0.015 |
| TOPAZIAN MD | 2 | 0.015 |
| TOPAL B | 2 | 0.015 |
| TONOUCHI H | 2 | 0.015 |
| TONKOPI E | 2 | 0.015 |
| TONG XJ | 2 | 0.015 |
| TONG M | 2 | 0.015 |
| TONG LJ | 2 | 0.015 |
| TOMLINSON D | 2 | 0.015 |
| TOMITA T | 2 | 0.015 |
| TOMINAGA H | 2 | 0.015 |
| TOMIAK A | 2 | 0.015 |
| TOMASELLO G | 2 | 0.015 |
| TOMASEK J | 2 | 0.015 |
| TOKUUYE K | 2 | 0.015 |
| TOKUNO K | 2 | 0.015 |
| TOKAR J | 2 | 0.015 |
| TOITA T | 2 | 0.015 |
| TOHMA T | 2 | 0.015 |
| TOGNI M | 2 | 0.015 |
| TOGASHI K | 2 | 0.015 |
| TODO M | 2 | 0.015 |
| TJULANDIN SA | 2 | 0.015 |
| TITCOMB DR | 2 | 0.015 |
| TIRUMANI SH | 2 | 0.015 |
| TIRUMANI H | 2 | 0.015 |
| TIMKE C | 2 | 0.015 |
| TILG H | 2 | 0.015 |
| TIKE P | 2 | 0.015 |
| TIESI G | 2 | 0.015 |
| TIERNEY WM | 2 | 0.015 |
| TIEN WY | 2 | 0.015 |
| TIAN XS | 2 | 0.015 |
| TIAN JH | 2 | 0.015 |
| TIAN D | 2 | 0.015 |
| TIAN CY | 2 | 0.015 |
| THUKRAL A | 2 | 0.015 |
| THRUMURTHY S | 2 | 0.015 |
| THORELL A | 2 | 0.015 |
| THOMFORD NE | 2 | 0.015 |
| THOMAY A | 2 | 0.015 |
| THOMAS N | 2 | 0.015 |
| THOMAS JP | 2 | 0.015 |
| THOMAS DG | 2 | 0.015 |
| THOMAS AL | 2 | 0.015 |
| THOMAIDIS T | 2 | 0.015 |
| THIRUMALAI S | 2 | 0.015 |
| THIRLWELL MP | 2 | 0.015 |
| THEZENAS S | 2 | 0.015 |
| THEODOSAKIS N | 2 | 0.015 |
| THEODOROPOULOS G | 2 | 0.015 |
| THATIKONDA C | 2 | 0.015 |
| THARAVEJ C | 2 | 0.015 |
| THALER M | 2 | 0.015 |
| TEUCHER B | 2 | 0.015 |
| TETREAULT MP | 2 | 0.015 |
| TESTONI PA | 2 | 0.015 |
| TERROSU G | 2 | 0.015 |
| TERLECKI G | 2 | 0.015 |
| TERESHKEVICH D | 2 | 0.015 |
| TERASHITA Y | 2 | 0.015 |
| TER VEER E | 2 | 0.015 |
| TEPPER JE | 2 | 0.015 |
| TEOH AYB | 2 | 0.015 |
| TEOH AY | 2 | 0.015 |
| TENG LS | 2 | 0.015 |
| TEN BRINKE A | 2 | 0.015 |
| TEMAN NR | 2 | 0.015 |
| TEKINBAS C | 2 | 0.015 |
| TEKIN SB | 2 | 0.015 |
| TEJANI MA | 2 | 0.015 |
| TEIXEIRA R | 2 | 0.015 |
| TEITELBAUM U | 2 | 0.015 |
| TEHFE MA | 2 | 0.015 |
| TEH M | 2 | 0.015 |
| TAWARAYA S | 2 | 0.015 |
| TAVAKKOLY-BAZZAZ J | 2 | 0.015 |
| TAUCHI K | 2 | 0.015 |
| TAUBENSLAG KJ | 2 | 0.015 |
| TAUBE JM | 2 | 0.015 |
| TATUM RP | 2 | 0.015 |
| TATSUGUCHI A | 2 | 0.015 |
| TATEISHI Y | 2 | 0.015 |
| TATEBE H | 2 | 0.015 |
| TASIOUDI KE | 2 | 0.015 |
| TASHIRO S | 2 | 0.015 |
| TASHIRO K | 2 | 0.015 |
| TASAKA S | 2 | 0.015 |
| TARAZONA N | 2 | 0.015 |
| TARABOLOUS C | 2 | 0.015 |
| TARABAR D | 2 | 0.015 |
| TAO YL | 2 | 0.015 |
| TAO KY | 2 | 0.015 |
| TAO KX | 2 | 0.015 |
| TAO K | 2 | 0.015 |
| TAO J | 2 | 0.015 |
| TAO B | 2 | 0.015 |
| TANUMA T | 2 | 0.015 |
| TANOUE K | 2 | 0.015 |
| TANIOKA D | 2 | 0.015 |
| TANIGUCHI S | 2 | 0.015 |
| TANIDA T | 2 | 0.015 |
| TANI Y | 2 | 0.015 |
| TANI N | 2 | 0.015 |
| TANG XY | 2 | 0.015 |
| TANG XW | 2 | 0.015 |
| TANG XF | 2 | 0.015 |
| TANG ST | 2 | 0.015 |
| TANG SH | 2 | 0.015 |
| TANG M | 2 | 0.015 |
| TANG KH | 2 | 0.015 |
| TANG ET | 2 | 0.015 |
| TANG CW | 2 | 0.015 |
| TANG CB | 2 | 0.015 |
| TANG C | 2 | 0.015 |
| TANG B | 2 | 0.015 |
| TANAHASHI T | 2 | 0.015 |
| TANABE T | 2 | 0.015 |
| TANABE A | 2 | 0.015 |
| TAN Y | 2 | 0.015 |
| TAN WN | 2 | 0.015 |
| TAN QY | 2 | 0.015 |
| TAN HH | 2 | 0.015 |
| TAMANDL D | 2 | 0.015 |
| TAMAKI W | 2 | 0.015 |
| TAM J | 2 | 0.015 |
| TALVAS J | 2 | 0.015 |
| TALSMA AK | 2 | 0.015 |
| TALLEC VJL | 2 | 0.015 |
| TALATI AA | 2 | 0.015 |
| TALAMONTI MS | 2 | 0.015 |
| TALAMONTI M | 2 | 0.015 |
| TALAMINI MA | 2 | 0.015 |
| TAKIZAWA H | 2 | 0.015 |
| TAKIMOTO R | 2 | 0.015 |
| TAKIGUCHI Y | 2 | 0.015 |
| TAKIGUCHI M | 2 | 0.015 |
| TAKIGAWA N | 2 | 0.015 |
| TAKI Y | 2 | 0.015 |
| TAKEYOSHI I | 2 | 0.015 |
| TAKEUCHI S | 2 | 0.015 |
| TAKETOMI A | 2 | 0.015 |
| TAKESHITA T | 2 | 0.015 |
| TAKENOYAMA M | 2 | 0.015 |
| TAKENAKA Y | 2 | 0.015 |
| TAKEMURA K | 2 | 0.015 |
| TAKEI Y | 2 | 0.015 |
| TAKEI R | 2 | 0.015 |
| TAKEDATSU H | 2 | 0.015 |
| TAKEDA H | 2 | 0.015 |
| TAKAYAMA Y | 2 | 0.015 |
| TAKAYAMA K | 2 | 0.015 |
| TAKATORI H | 2 | 0.015 |
| TAKASHIMA N | 2 | 0.015 |
| TAKALA H | 2 | 0.015 |
| TAKAHASHI C | 2 | 0.015 |
| TAKAGANE A | 2 | 0.015 |
| TAKABAYASHI A | 2 | 0.015 |
| TAIT DM | 2 | 0.015 |
| TAIRA K | 2 | 0.015 |
| TAIOLI E | 2 | 0.015 |
| TAI A | 2 | 0.015 |
| TAHIRI S | 2 | 0.015 |
| TAHERI NS | 2 | 0.015 |
| TAGHAVI N | 2 | 0.015 |
| TAGAYA N | 2 | 0.015 |
| TADDEI A | 2 | 0.015 |
| TADA H | 2 | 0.015 |
| TACK J | 2 | 0.015 |
| TACHIBANA I | 2 | 0.015 |
| TABATA H | 2 | 0.015 |
| SZYMONIFKA J | 2 | 0.015 |
| SZYMANSKA K | 2 | 0.015 |
| SZIGETI A | 2 | 0.015 |
| SZETO L | 2 | 0.015 |
| SZENTIRMAY Z | 2 | 0.015 |
| SWALLOW C | 2 | 0.015 |
| SVOBODA M | 2 | 0.015 |
| SUTTON CD | 2 | 0.015 |
| SUSSMAN MS | 2 | 0.015 |
| SURATI M | 2 | 0.015 |
| SUO J | 2 | 0.015 |
| SUNPAWERAVONG P | 2 | 0.015 |
| SUNGUR-STASIK K | 2 | 0.015 |
| SUNG L | 2 | 0.015 |
| SUNDHEIM L | 2 | 0.015 |
| SUNDELOF M | 2 | 0.015 |
| SUNDBOM M | 2 | 0.015 |
| SUNDARESAN SR | 2 | 0.015 |
| SUNAGAWA R | 2 | 0.015 |
| SUNAGA T | 2 | 0.015 |
| SUN YY | 2 | 0.015 |
| SUN YL | 2 | 0.015 |
| SUN YF | 2 | 0.015 |
| SUN YB | 2 | 0.015 |
| SUN XX | 2 | 0.015 |
| SUN XM | 2 | 0.015 |
| SUN XG | 2 | 0.015 |
| SUN WY | 2 | 0.015 |
| SUN SY | 2 | 0.015 |
| SUN SS | 2 | 0.015 |
| SUN SR | 2 | 0.015 |
| SUN SL | 2 | 0.015 |
| SUN S | 2 | 0.015 |
| SUN P | 2 | 0.015 |
| SUN NN | 2 | 0.015 |
| SUN MM | 2 | 0.015 |
| SUN ML | 2 | 0.015 |
| SUN LY | 2 | 0.015 |
| SUN JJ | 2 | 0.015 |
| SUN JH | 2 | 0.015 |
| SUN H | 2 | 0.015 |
| SUN DY | 2 | 0.015 |
| SUN CL | 2 | 0.015 |
| SUMPTER K | 2 | 0.015 |
| SUMIYAMA K | 2 | 0.015 |
| SULYOK M | 2 | 0.015 |
| SULTANOV FS | 2 | 0.015 |
| SULEWSKA A | 2 | 0.015 |
| SUKOCHEVA OA | 2 | 0.015 |
| SUKAWA Y | 2 | 0.015 |
| SUH Y | 2 | 0.015 |
| SUH C | 2 | 0.015 |
| SUGIURA Y | 2 | 0.015 |
| SUGIMOTO M | 2 | 0.015 |
| SUGIE C | 2 | 0.015 |
| SUGAWARA M | 2 | 0.015 |
| SUGAWARA K | 2 | 0.015 |
| SUGASAWA H | 2 | 0.015 |
| SUGARBAKER DJ | 2 | 0.015 |
| SUGANUMA K | 2 | 0.015 |
| SUGANO K | 2 | 0.015 |
| SUGAMOTO Y | 2 | 0.015 |
| SUEOKA S | 2 | 0.015 |
| SUEHIRO S | 2 | 0.015 |
| SUDA T | 2 | 0.015 |
| SUCHI K | 2 | 0.015 |
| SUBRAMANYAM SS | 2 | 0.015 |
| SUBRAMANIAN H | 2 | 0.015 |
| SUBBIAH S | 2 | 0.015 |
| SUBASI M | 2 | 0.015 |
| SU ZJ | 2 | 0.015 |
| SU XW | 2 | 0.015 |
| SU XH | 2 | 0.015 |
| SU LP | 2 | 0.015 |
| SU JL | 2 | 0.015 |
| SU HX | 2 | 0.015 |
| SU F | 2 | 0.015 |
| SU D | 2 | 0.015 |
| SU CQ | 2 | 0.015 |
| SU CH | 2 | 0.015 |
| STURGIS EM | 2 | 0.015 |
| STUBBS B | 2 | 0.015 |
| STROSBERG JR | 2 | 0.015 |
| STRONG VEM | 2 | 0.015 |
| STROEHLEIN JR | 2 | 0.015 |
| STROBEL P | 2 | 0.015 |
| STREETS CG | 2 | 0.015 |
| STRATE TG | 2 | 0.015 |
| STRANDBY RB | 2 | 0.015 |
| STORM HH | 2 | 0.015 |
| STOLZENBERG-SOLOMON RZ | 2 | 0.015 |
| STOLZEL U | 2 | 0.015 |
| STOJAKOV D | 2 | 0.015 |
| STOEV SD | 2 | 0.015 |
| STOECKLEIN N | 2 | 0.015 |
| STOCKELD D | 2 | 0.015 |
| STILIDI I | 2 | 0.015 |
| STIEVANO L | 2 | 0.015 |
| STIELER J | 2 | 0.015 |
| STIEKEMA J | 2 | 0.015 |
| STEVENS C | 2 | 0.015 |
| STEURER S | 2 | 0.015 |
| STEUP WH | 2 | 0.015 |
| STESSIN A | 2 | 0.015 |
| STEPHENS S | 2 | 0.015 |
| STEPHENS PJ | 2 | 0.015 |
| STELLA PJ | 2 | 0.015 |
| STEINERT HC | 2 | 0.015 |
| STEINBERG J | 2 | 0.015 |
| STEIN M | 2 | 0.015 |
| STATNIKOV A | 2 | 0.015 |
| STASSEN LPS | 2 | 0.015 |
| STASSEN LP | 2 | 0.015 |
| STARLINGER P | 2 | 0.015 |
| STARK M | 2 | 0.015 |
| STANKOVIC V | 2 | 0.015 |
| STANKOVA B | 2 | 0.015 |
| STANGL JR | 2 | 0.015 |
| STANCIC-ROKOTOV D | 2 | 0.015 |
| STAIGER W | 2 | 0.015 |
| STAGG J | 2 | 0.015 |
| STAFINSKI T | 2 | 0.015 |
| STADELMANN T | 2 | 0.015 |
| SROVNAL J | 2 | 0.015 |
| SRIRAM P | 2 | 0.015 |
| SRINIVASAN S | 2 | 0.015 |
| SRINIVASAN A | 2 | 0.015 |
| SRIDHAR R | 2 | 0.015 |
| SREY C | 2 | 0.015 |
| SPRATLIN JL | 2 | 0.015 |
| SPOLVERATO G | 2 | 0.015 |
| SPIZZO G | 2 | 0.015 |
| SPITZER-NAAYKENS JMJ | 2 | 0.015 |
| SPITZ MR | 2 | 0.015 |
| SPIRA AI | 2 | 0.015 |
| SPICAK J | 2 | 0.015 |
| SPICA B | 2 | 0.015 |
| SPERDUTI I | 2 | 0.015 |
| SPENCE GM | 2 | 0.015 |
| SPELSBERG FW | 2 | 0.015 |
| SPECTOR SA | 2 | 0.015 |
| SPAUN G | 2 | 0.015 |
| SPARLING L | 2 | 0.015 |
| SPARLING JL | 2 | 0.015 |
| SPACEK MB | 2 | 0.015 |
| SPAANDER M | 2 | 0.015 |
| SOYSAL SD | 2 | 0.015 |
| SOYKA JD | 2 | 0.015 |
| SOWA Y | 2 | 0.015 |
| SOUZA R | 2 | 0.015 |
| SOUVATZOGLOU M | 2 | 0.015 |
| SOUTTO M | 2 | 0.015 |
| SOUCY G | 2 | 0.015 |
| SOSEF M | 2 | 0.015 |
| SORRENTINO M | 2 | 0.015 |
| SORIA JC | 2 | 0.015 |
| SORENSEN M | 2 | 0.015 |
| SORENSEN LM | 2 | 0.015 |
| SONTAG S | 2 | 0.015 |
| SONG ZM | 2 | 0.015 |
| SONG YJ | 2 | 0.015 |
| SONG YB | 2 | 0.015 |
| SONG XM | 2 | 0.015 |
| SONG TN | 2 | 0.015 |
| SONG SW | 2 | 0.015 |
| SONG SL | 2 | 0.015 |
| SONG MQ | 2 | 0.015 |
| SONG MJ | 2 | 0.015 |
| SONG JM | 2 | 0.015 |
| SONG IH | 2 | 0.015 |
| SONG HZ | 2 | 0.015 |
| SONG HX | 2 | 0.015 |
| SONG HS | 2 | 0.015 |
| SONG HM | 2 | 0.015 |
| SONG GB | 2 | 0.015 |
| SONG G | 2 | 0.015 |
| SONG FZ | 2 | 0.015 |
| SONG EW | 2 | 0.015 |
| SONG C | 2 | 0.015 |
| SONESTEDT E | 2 | 0.015 |
| SONDERMEIJER CMT | 2 | 0.015 |
| SONDERMEIJER C | 2 | 0.015 |
| SON YI | 2 | 0.015 |
| SON JB | 2 | 0.015 |
| SON HJ | 2 | 0.015 |
| SOMOGYI L | 2 | 0.015 |
| SOMMERS SR | 2 | 0.015 |
| SOMEYA M | 2 | 0.015 |
| SOLTERMANN A | 2 | 0.015 |
| SOLOMON N | 2 | 0.015 |
| SOLMI M | 2 | 0.015 |
| SOLLANO JD | 2 | 0.015 |
| SOLFRIZZO M | 2 | 0.015 |
| SOLE CV | 2 | 0.015 |
| SOKOUTI M | 2 | 0.015 |
| SOKO R | 2 | 0.015 |
| SOIPOVA M | 2 | 0.015 |
| SOHRABPOUR AA | 2 | 0.015 |
| SOHN TS | 2 | 0.015 |
| SOHN HM | 2 | 0.015 |
| SOHMA I | 2 | 0.015 |
| SOHANAKI H | 2 | 0.015 |
| SOHAL DPS | 2 | 0.015 |
| SOEJIMA Y | 2 | 0.015 |
| SODA H | 2 | 0.015 |
| SOARES-LIMA SC | 2 | 0.015 |
| SOARES HP | 2 | 0.015 |
| SNOOK A | 2 | 0.015 |
| SMYTH E | 2 | 0.015 |
| SMOLLE J | 2 | 0.015 |
| SMOLKA AJ | 2 | 0.015 |
| SMITS MM | 2 | 0.015 |
| SMITH-RAYMOND L | 2 | 0.015 |
| SMITH MG | 2 | 0.015 |
| SMITH JW | 2 | 0.015 |
| SMITH GS | 2 | 0.015 |
| SMITH G | 2 | 0.015 |
| SMITH DA | 2 | 0.015 |
| SMITH CA | 2 | 0.015 |
| SMETS E | 2 | 0.015 |
| SMART HL | 2 | 0.015 |
| SMARANDACHE G | 2 | 0.015 |
| SMALL W | 2 | 0.015 |
| SMALL AJ | 2 | 0.015 |
| SLUMP K | 2 | 0.015 |
| SLAUGHTER JC | 2 | 0.015 |
| SLATER JM | 2 | 0.015 |
| SLART R | 2 | 0.015 |
| SLANGER TE | 2 | 0.015 |
| SLAMOVA A | 2 | 0.015 |
| SLAMAN AE | 2 | 0.015 |
| SLACK R | 2 | 0.015 |
| SLABY O | 2 | 0.015 |
| SKOV BG | 2 | 0.015 |
| SKOCZYLAS T | 2 | 0.015 |
| SKIPWORTH RJE | 2 | 0.015 |
| SKAAR TC | 2 | 0.015 |
| SIVEKE J | 2 | 0.015 |
| SITNIKOVA K | 2 | 0.015 |
| SISTONEN SJ | 2 | 0.015 |
| SIRAK I | 2 | 0.015 |
| SIO TT | 2 | 0.015 |
| SINHA R | 2 | 0.015 |
| SINHA P | 2 | 0.015 |
| SINGLA S | 2 | 0.015 |
| SINGH P | 2 | 0.015 |
| SINGH LC | 2 | 0.015 |
| SINGH J | 2 | 0.015 |
| SINGH I | 2 | 0.015 |
| SINGH G | 2 | 0.015 |
| SINGH D | 2 | 0.015 |
| SINGH AP | 2 | 0.015 |
| SINGARAJU M | 2 | 0.015 |
| SINACORE J | 2 | 0.015 |
| SIMON SL | 2 | 0.015 |
| SIMAO TD | 2 | 0.015 |
| SILVESTRO L | 2 | 0.015 |
| SILVESTRIS N | 2 | 0.015 |
| SILVERMAN DT | 2 | 0.015 |
| SILSKI L | 2 | 0.015 |
| SILBERSTEIN PT | 2 | 0.015 |
| SIKORSKI AF | 2 | 0.015 |
| SIKORA AG | 2 | 0.015 |
| SIJTSEMA M | 2 | 0.015 |
| SIFFERT W | 2 | 0.015 |
| SIEWKO M | 2 | 0.015 |
| SIESLING S | 2 | 0.015 |
| SIERSEMA P | 2 | 0.015 |
| SIERI S | 2 | 0.015 |
| SIEGEL RL | 2 | 0.015 |
| SIDRANSKY D | 2 | 0.015 |
| SIDHU S | 2 | 0.015 |
| SIDDIQUI FA | 2 | 0.015 |
| SIBONI S | 2 | 0.015 |
| SHULMAN K | 2 | 0.015 |
| SHUKLA V | 2 | 0.015 |
| SHRIVASTAVA R | 2 | 0.015 |
| SHRIEVE DC | 2 | 0.015 |
| SHOWALTER TN | 2 | 0.015 |
| SHOU CC | 2 | 0.015 |
| SHONO T | 2 | 0.015 |
| SHOJI M | 2 | 0.015 |
| SHO M | 2 | 0.015 |
| SHIRYAJEV YN | 2 | 0.015 |
| SHIRAO K | 2 | 0.015 |
| SHIRAISHI N | 2 | 0.015 |
| SHIODE J | 2 | 0.015 |
| SHINOZUKA E | 2 | 0.015 |
| SHINOZAKI T | 2 | 0.015 |
| SHINKAI M | 2 | 0.015 |
| SHING M | 2 | 0.015 |
| SHINDEN Y | 2 | 0.015 |
| SHINAGARE AB | 2 | 0.015 |
| SHIN SJ | 2 | 0.015 |
| SHIN DM | 2 | 0.015 |
| SHIN D | 2 | 0.015 |
| SHIMPUKU M | 2 | 0.015 |
| SHIMPI RA | 2 | 0.015 |
| SHIMONO T | 2 | 0.015 |
| SHIMIZU N | 2 | 0.015 |
| SHIMIZU A | 2 | 0.015 |
| SHIMAMURA T | 2 | 0.015 |
| SHIM CN | 2 | 0.015 |
| SHIH SC | 2 | 0.015 |
| SHIH CS | 2 | 0.015 |
| SHIGEMITSU K | 2 | 0.015 |
| SHIELDS A | 2 | 0.015 |
| SHIELD KD | 2 | 0.015 |
| SHIBAMOTO Y | 2 | 0.015 |
| SHIBA S | 2 | 0.015 |
| SHIA J | 2 | 0.015 |
| SHI YP | 2 | 0.015 |
| SHI YK | 2 | 0.015 |
| SHI W | 2 | 0.015 |
| SHI QL | 2 | 0.015 |
| SHI N | 2 | 0.015 |
| SHI LL | 2 | 0.015 |
| SHI JZ | 2 | 0.015 |
| SHI JL | 2 | 0.015 |
| SHI HY | 2 | 0.015 |
| SHI DL | 2 | 0.015 |
| SHERMAN KL | 2 | 0.015 |
| SHERMAN CA | 2 | 0.015 |
| SHER T | 2 | 0.015 |
| SHEPHERD N | 2 | 0.015 |
| SHEPHERD FA | 2 | 0.015 |
| SHENG YQ | 2 | 0.015 |
| SHENG Y | 2 | 0.015 |
| SHENG WQ | 2 | 0.015 |
| SHENG JQ | 2 | 0.015 |
| SHENFINE J | 2 | 0.015 |
| SHENDE M | 2 | 0.015 |
| SHEN XY | 2 | 0.015 |
| SHEN XM | 2 | 0.015 |
| SHEN XC | 2 | 0.015 |
| SHEN X | 2 | 0.015 |
| SHEN WD | 2 | 0.015 |
| SHEN R | 2 | 0.015 |
| SHEN P | 2 | 0.015 |
| SHEN N | 2 | 0.015 |
| SHEN LJ | 2 | 0.015 |
| SHEN KH | 2 | 0.015 |
| SHEN K | 2 | 0.015 |
| SHEN JG | 2 | 0.015 |
| SHEN H | 2 | 0.015 |
| SHEN G | 2 | 0.015 |
| SHEN FF | 2 | 0.015 |
| SHEN CH | 2 | 0.015 |
| SHEN B | 2 | 0.015 |
| SHEILS OM | 2 | 0.015 |
| SHARP L | 2 | 0.015 |
| SHARMA SK | 2 | 0.015 |
| SHARMA MK | 2 | 0.015 |
| SHARMA JB | 2 | 0.015 |
| SHARMA D | 2 | 0.015 |
| SHARMA B | 2 | 0.015 |
| SHARGALL Y | 2 | 0.015 |
| SHARATA A | 2 | 0.015 |
| SHARAN K | 2 | 0.015 |
| SHARAIHA RZ | 2 | 0.015 |
| SHAO YX | 2 | 0.015 |
| SHAO Y | 2 | 0.015 |
| SHAO WL | 2 | 0.015 |
| SHAO LL | 2 | 0.015 |
| SHAO LJ | 2 | 0.015 |
| SHAO L | 2 | 0.015 |
| SHAO JC | 2 | 0.015 |
| SHAO C | 2 | 0.015 |
| SHANKARAN V | 2 | 0.015 |
| SHANG Y | 2 | 0.015 |
| SHANG DP | 2 | 0.015 |
| SHANG CG | 2 | 0.015 |
| SHAN X | 2 | 0.015 |
| SHAN TY | 2 | 0.015 |
| SHAN FB | 2 | 0.015 |
| SHAN F | 2 | 0.015 |
| SHAIDAROV M | 2 | 0.015 |
| SHAHSAFAEI A | 2 | 0.015 |
| SHAH SA | 2 | 0.015 |
| SHAH RJ | 2 | 0.015 |
| SHAH JL | 2 | 0.015 |
| SHAFFI S | 2 | 0.015 |
| SHAFFER J | 2 | 0.015 |
| SHABIR N | 2 | 0.015 |
| SHABBIR A | 2 | 0.015 |
| SEYEDNEJAD F | 2 | 0.015 |
| SETTLE SH | 2 | 0.015 |
| SETTER C | 2 | 0.015 |
| SERVARAYAN CM | 2 | 0.015 |
| SERVAGI-VERNAT S | 2 | 0.015 |
| SERRE AA | 2 | 0.015 |
| SERRANO M | 2 | 0.015 |
| SERIZAWA M | 2 | 0.015 |
| SERGI G | 2 | 0.015 |
| SEPULVEDA AR | 2 | 0.015 |
| SEONG J | 2 | 0.015 |
| SEO Y | 2 | 0.015 |
| SENESSE P | 2 | 0.015 |
| SENENT SG | 2 | 0.015 |
| SEMRAU S | 2 | 0.015 |
| SEMPLE ME | 2 | 0.015 |
| SEMERARO M | 2 | 0.015 |
| SEMBA Y | 2 | 0.015 |
| SEKINO N | 2 | 0.015 |
| SEKINE Y | 2 | 0.015 |
| SEKINE M | 2 | 0.015 |
| SEKIMOTO M | 2 | 0.015 |
| SEKII S | 2 | 0.015 |
| SEKIGUCHI R | 2 | 0.015 |
| SEKHON GS | 2 | 0.015 |
| SEJIMA K | 2 | 0.015 |
| SEITZ HK | 2 | 0.015 |
| SEIPELT G | 2 | 0.015 |
| SEINO H | 2 | 0.015 |
| SEHDEV A | 2 | 0.015 |
| SEGOL P | 2 | 0.015 |
| SEGAMI K | 2 | 0.015 |
| SEEVARATNAM R | 2 | 0.015 |
| SEESING MFJ | 2 | 0.015 |
| SEERY TE | 2 | 0.015 |
| SEDAGHAT SM | 2 | 0.015 |
| SCORILAS A | 2 | 0.015 |
| SCOGGINS C | 2 | 0.015 |
| SCHWERTNER C | 2 | 0.015 |
| SCHWARZ S | 2 | 0.015 |
| SCHWARZ RA | 2 | 0.015 |
| SCHWARZ B | 2 | 0.015 |
| SCHWARTZ SJ | 2 | 0.015 |
| SCHWARTZ LH | 2 | 0.015 |
| SCHWARTZ L | 2 | 0.015 |
| SCHWARTZ J | 2 | 0.015 |
| SCHWARTZ G | 2 | 0.015 |
| SCHWACKE J | 2 | 0.015 |
| SCHUMACHER S | 2 | 0.015 |
| SCHULZ S | 2 | 0.015 |
| SCHULTZE A | 2 | 0.015 |
| SCHULER M | 2 | 0.015 |
| SCHUEBEL KE | 2 | 0.015 |
| SCHUCH G | 2 | 0.015 |
| SCHREURS WMJ | 2 | 0.015 |
| SCHRAEPEN MC | 2 | 0.015 |
| SCHOPFLIN A | 2 | 0.015 |
| SCHONE C | 2 | 0.015 |
| SCHOMISCH SJ | 2 | 0.015 |
| SCHOLLAERT P | 2 | 0.015 |
| SCHOFIELD PT | 2 | 0.015 |
| SCHOFFSKI P | 2 | 0.015 |
| SCHOENBERG JB | 2 | 0.015 |
| SCHOEMAN A | 2 | 0.015 |
| SCHODER H | 2 | 0.015 |
| SCHNOY E | 2 | 0.015 |
| SCHNIEWIND B | 2 | 0.015 |
| SCHNEIDERS FI | 2 | 0.015 |
| SCHNEIDER S | 2 | 0.015 |
| SCHNEIDER M | 2 | 0.015 |
| SCHNEIDER L | 2 | 0.015 |
| SCHMOLLACK J | 2 | 0.015 |
| SCHMITT M | 2 | 0.015 |
| SCHMITT JM | 2 | 0.015 |
| SCHMIDTMANN I | 2 | 0.015 |
| SCHMIDT-YANG M | 2 | 0.015 |
| SCHMIDT R | 2 | 0.015 |
| SCHMIDT J | 2 | 0.015 |
| SCHMIDT HM | 2 | 0.015 |
| SCHMIDT CR | 2 | 0.015 |
| SCHMIDT C | 2 | 0.015 |
| SCHMID R | 2 | 0.015 |
| SCHMETZLE R | 2 | 0.015 |
| SCHMELZLE R | 2 | 0.015 |
| SCHMELZLE M | 2 | 0.015 |
| SCHMEIER S | 2 | 0.015 |
| SCHLITT HJ | 2 | 0.015 |
| SCHLEGEL V | 2 | 0.015 |
| SCHLECK CD | 2 | 0.015 |
| SCHJOTH JE | 2 | 0.015 |
| SCHIRMER CC | 2 | 0.015 |
| SCHIPPER P | 2 | 0.015 |
| SCHILLER DE | 2 | 0.015 |
| SCHILL S | 2 | 0.015 |
| SCHIFFNER DC | 2 | 0.015 |
| SCHIFFMAN SC | 2 | 0.015 |
| SCHIFF M | 2 | 0.015 |
| SCHIEMAN C | 2 | 0.015 |
| SCHICHA H | 2 | 0.015 |
| SCHETTER AJ | 2 | 0.015 |
| SCHEJBALOVA M | 2 | 0.015 |
| SCHATTNER MA | 2 | 0.015 |
| SCHAIBLE A | 2 | 0.015 |
| SCHAFMAYER C | 2 | 0.015 |
| SCHAEFER H | 2 | 0.015 |
| SCHACHNER M | 2 | 0.015 |
| SCARPIGNATO C | 2 | 0.015 |
| SCAGLIORI E | 2 | 0.015 |
| SAYEHMIRI K | 2 | 0.015 |
| SAXENA R | 2 | 0.015 |
| SAWHNEY MS | 2 | 0.015 |
| SAWADA K | 2 | 0.015 |
| SAWA Y | 2 | 0.015 |
| SAVAS B | 2 | 0.015 |
| SAVANI RC | 2 | 0.015 |
| SAVA M | 2 | 0.015 |
| SAUNDERS MD | 2 | 0.015 |
| SAUNDERS M | 2 | 0.015 |
| SAUERLAND C | 2 | 0.015 |
| SAUER R | 2 | 0.015 |
| SATODATE H | 2 | 0.015 |
| SATO S | 2 | 0.015 |
| SATAKE H | 2 | 0.015 |
| SASTRE J | 2 | 0.015 |
| SASSEN S | 2 | 0.015 |
| SASS AV | 2 | 0.015 |
| SASAKI R | 2 | 0.015 |
| SASAGAWA H | 2 | 0.015 |
| SARSENOVA S | 2 | 0.015 |
| SARKAR A | 2 | 0.015 |
| SAPINO A | 2 | 0.015 |
| SANTOSO JT | 2 | 0.015 |
| SANTORO A | 2 | 0.015 |
| SANTI S | 2 | 0.015 |
| SANTELLA RM | 2 | 0.015 |
| SANS M | 2 | 0.015 |
| SANRI E | 2 | 0.015 |
| SANO M | 2 | 0.015 |
| SANKPAL UT | 2 | 0.015 |
| SANDSTAD B | 2 | 0.015 |
| SANDLER BJ | 2 | 0.015 |
| SANDHU H | 2 | 0.015 |
| SANCHO-SERRA MD | 2 | 0.015 |
| SANCHEZ-YAGUE A | 2 | 0.015 |
| SANCHEZ-ESPIRIDION B | 2 | 0.015 |
| SANATANI M | 2 | 0.015 |
| SAMSON P | 2 | 0.015 |
| SAMMON AM | 2 | 0.015 |
| SAMEER AS | 2 | 0.015 |
| SAMBASIVASELLI R | 2 | 0.015 |
| SAMAPUNDO S | 2 | 0.015 |
| SAMAEI NM | 2 | 0.015 |
| SALTER BJ | 2 | 0.015 |
| SALOMON Y | 2 | 0.015 |
| SALMINEN E | 2 | 0.015 |
| SALIGRAM S | 2 | 0.015 |
| SALEMME M | 2 | 0.015 |
| SALEHI MH | 2 | 0.015 |
| SALEH-EBRAHIMI L | 2 | 0.015 |
| SALAMA J | 2 | 0.015 |
| SALAGRAMA S | 2 | 0.015 |
| SAKURAZAWA N | 2 | 0.015 |
| SAKURAI S | 2 | 0.015 |
| SAKURADA M | 2 | 0.015 |
| SAKURABA M | 2 | 0.015 |
| SAKUMA H | 2 | 0.015 |
| SAKELLARIOU S | 2 | 0.015 |
| SAKATA J | 2 | 0.015 |
| SAKAMOTO C | 2 | 0.015 |
| SAKAMAKI K | 2 | 0.015 |
| SAKAI C | 2 | 0.015 |
| SAKAGUCHI S | 2 | 0.015 |
| SAKAEDA T | 2 | 0.015 |
| SAKA M | 2 | 0.015 |
| SAKA E | 2 | 0.015 |
| SAITO D | 2 | 0.015 |
| SAIKIA BJ | 2 | 0.015 |
| SAIKA T | 2 | 0.015 |
| SAIGENJI K | 2 | 0.015 |
| SAI S | 2 | 0.015 |
| SAHNI P | 2 | 0.015 |
| SAHIN U | 2 | 0.015 |
| SAHA A | 2 | 0.015 |
| SAGAWA H | 2 | 0.015 |
| SAFFERY C | 2 | 0.015 |
| SAFATLE-RIBEIRO AV | 2 | 0.015 |
| SAEGER HD | 2 | 0.015 |
| SAEED M | 2 | 0.015 |
| SAEDI HS | 2 | 0.015 |
| SADJADI AR | 2 | 0.015 |
| SACHLOVA M | 2 | 0.015 |
| SABLJAK P | 2 | 0.015 |
| SABHARWAL T | 2 | 0.015 |
| SABET A | 2 | 0.015 |
| SABAU D | 2 | 0.015 |
| SABAU A | 2 | 0.015 |
| SABA N | 2 | 0.015 |
| RYUGE N | 2 | 0.015 |
| RYU KW | 2 | 0.015 |
| RYU J | 2 | 0.015 |
| RYOUTOKUJI T | 2 | 0.015 |
| RYOO BY | 2 | 0.015 |
| RYAN CE | 2 | 0.015 |
| RYAN A | 2 | 0.015 |
| RUSTHOVEN K | 2 | 0.015 |
| RUSSO JK | 2 | 0.015 |
| RUSSO A | 2 | 0.015 |
| RUSSELL RGG | 2 | 0.015 |
| RUSSELL MC | 2 | 0.015 |
| RUSIDANMU A | 2 | 0.015 |
| RUSCH V | 2 | 0.015 |
| RUPPERT BN | 2 | 0.015 |
| RULE WG | 2 | 0.015 |
| RUFFATO A | 2 | 0.015 |
| RUETH NM | 2 | 0.015 |
| RUERS TJM | 2 | 0.015 |
| RUDNAI P | 2 | 0.015 |
| RUBIO CA | 2 | 0.015 |
| RUBESIN SE | 2 | 0.015 |
| RUANGSIN S | 2 | 0.015 |
| RUAN XK | 2 | 0.015 |
| RU Y | 2 | 0.015 |
| ROZAS MS | 2 | 0.015 |
| ROY-CHOWDHURI S | 2 | 0.015 |
| ROWINSKY EK | 2 | 0.015 |
| ROWE A | 2 | 0.015 |
| ROUTLEDGE MN | 2 | 0.015 |
| ROUSSEAU M | 2 | 0.015 |
| ROUSSEAU D | 2 | 0.015 |
| ROUKOS DH | 2 | 0.015 |
| ROTTINGHAUS GE | 2 | 0.015 |
| ROSSI E | 2 | 0.015 |
| ROSSETTI S | 2 | 0.015 |
| ROSS-INNES CS | 2 | 0.015 |
| ROSS JA | 2 | 0.015 |
| ROSS A | 2 | 0.015 |
| ROSENTHAL MH | 2 | 0.015 |
| ROSEN L | 2 | 0.015 |
| ROSATO V | 2 | 0.015 |
| ROSATO A | 2 | 0.015 |
| ROSATI C | 2 | 0.015 |
| ROSA AA | 2 | 0.015 |
| ROPER H | 2 | 0.015 |
| RONG YM | 2 | 0.015 |
| RONDAK IC | 2 | 0.015 |
| ROLFO C | 2 | 0.015 |
| ROKKAS T | 2 | 0.015 |
| ROHRBERG KS | 2 | 0.015 |
| ROH JL | 2 | 0.015 |
| ROESSNER A | 2 | 0.015 |
| RODRIGUES GB | 2 | 0.015 |
| RODGERS MGG | 2 | 0.015 |
| ROBINSON E | 2 | 0.015 |
| ROBINSON C | 2 | 0.015 |
| RIZK MK | 2 | 0.015 |
| RITZ JP | 2 | 0.015 |
| RITTER M | 2 | 0.015 |
| RITCH PS | 2 | 0.015 |
| RISTIMAKI A | 2 | 0.015 |
| RIPLEY RT | 2 | 0.015 |
| RIOS J | 2 | 0.015 |
| RINKES IHB | 2 | 0.015 |
| RIMNER A | 2 | 0.015 |
| RIMKUS C | 2 | 0.015 |
| RIEGLER FM | 2 | 0.015 |
| RIEFF EA | 2 | 0.015 |
| RIEDL KM | 2 | 0.015 |
| RIEDER E | 2 | 0.015 |
| RIDDELL RH | 2 | 0.015 |
| RICCI C | 2 | 0.015 |
| RICCHINI F | 2 | 0.015 |
| RIBI K | 2 | 0.015 |
| RIBEIRO U | 2 | 0.015 |
| RIBEIRO ML | 2 | 0.015 |
| RHOADES B | 2 | 0.015 |
| REZVANI SM | 2 | 0.015 |
| REZAEI N | 2 | 0.015 |
| REYNOLDS AR | 2 | 0.015 |
| REX D | 2 | 0.015 |
| RETHWISCH V | 2 | 0.015 |
| REN ZB | 2 | 0.015 |
| REN XB | 2 | 0.015 |
| REN P | 2 | 0.015 |
| REN MX | 2 | 0.015 |
| REN KW | 2 | 0.015 |
| REN FH | 2 | 0.015 |
| REN C | 2 | 0.015 |
| REISNER ML | 2 | 0.015 |
| REISMAN D | 2 | 0.015 |
| REIS TA | 2 | 0.015 |
| REINHARDT R | 2 | 0.015 |
| REINDERS JG | 2 | 0.015 |
| REIN LE | 2 | 0.015 |
| REICHERT R | 2 | 0.015 |
| REGULA J | 2 | 0.015 |
| REGMI R | 2 | 0.015 |
| REGINE WF | 2 | 0.015 |
| REGAN J | 2 | 0.015 |
| REES JR | 2 | 0.015 |
| REEN RK | 2 | 0.015 |
| REED MF | 2 | 0.015 |
| REECE-SMITH AM | 2 | 0.015 |
| REDDY K | 2 | 0.015 |
| REBISCHUNG C | 2 | 0.015 |
| RAZIEE HR | 2 | 0.015 |
| RAZ D | 2 | 0.015 |
| RAWLINSON CE | 2 | 0.015 |
| RAWLINSON C | 2 | 0.015 |
| RAVINDRAN NC | 2 | 0.015 |
| RAVAIOLI A | 2 | 0.015 |
| RAUSER S | 2 | 0.015 |
| RATTI M | 2 | 0.015 |
| RATOSA I | 2 | 0.015 |
| RAT P | 2 | 0.015 |
| RASTOGI N | 2 | 0.015 |
| RASANEN K | 2 | 0.015 |
| RAPP C | 2 | 0.015 |
| RAOUL J | 2 | 0.015 |
| RAO M | 2 | 0.015 |
| RAO D | 2 | 0.015 |
| RANTANEN TK | 2 | 0.015 |
| RANSOM BWS | 2 | 0.015 |
| RANGAD GM | 2 | 0.015 |
| RAN YG | 2 | 0.015 |
| RAMOS-FONT C | 2 | 0.015 |
| RAMOS FJ | 2 | 0.015 |
| RAMIREZ F | 2 | 0.015 |
| RAMAN V | 2 | 0.015 |
| RAMAIYA NH | 2 | 0.015 |
| RAMACCIATO G | 2 | 0.015 |
| RAJENDRA S | 2 | 0.015 |
| RAJDEV L | 2 | 0.015 |
| RAJAN E | 2 | 0.015 |
| RAJ SM | 2 | 0.015 |
| RAHMANI F | 2 | 0.015 |
| RAHIMZADEH-BARZOKI H | 2 | 0.015 |
| RAHAL MM | 2 | 0.015 |
| RAGULIN-COYNE E | 2 | 0.015 |
| RAGGI M | 2 | 0.015 |
| RAEDLE J | 2 | 0.015 |
| RA J | 2 | 0.015 |
| QVORTRUP C | 2 | 0.015 |
| QVIST N | 2 | 0.015 |
| QURIESHI MA | 2 | 0.015 |
| QURESHI A | 2 | 0.015 |
| QUJEQ D | 2 | 0.015 |
| QUIVRIN M | 2 | 0.015 |
| QUANG T | 2 | 0.015 |
| QU X | 2 | 0.015 |
| QU SN | 2 | 0.015 |
| QIU YP | 2 | 0.015 |
| QIU T | 2 | 0.015 |
| QIU LX | 2 | 0.015 |
| QIU HX | 2 | 0.015 |
| QIU HJ | 2 | 0.015 |
| QIU GQ | 2 | 0.015 |
| QIU G | 2 | 0.015 |
| QIN YH | 2 | 0.015 |
| QIN G | 2 | 0.015 |
| QIN CY | 2 | 0.015 |
| QIAO JJ | 2 | 0.015 |
| QIAO B | 2 | 0.015 |
| QIAN XM | 2 | 0.015 |
| QIAN X | 2 | 0.015 |
| QIAN W | 2 | 0.015 |
| QIAN T | 2 | 0.015 |
| QIAN RY | 2 | 0.015 |
| QIAN H | 2 | 0.015 |
| QIAN C | 2 | 0.015 |
| QI XT | 2 | 0.015 |
| QI WX | 2 | 0.015 |
| QI QF | 2 | 0.015 |
| QI L | 2 | 0.015 |
| QI J | 2 | 0.015 |
| QI HP | 2 | 0.015 |
| QI HC | 2 | 0.015 |
| PUYRAVEAU M | 2 | 0.015 |
| PUTTAWIBUL P | 2 | 0.015 |
| PUSTOWKA A | 2 | 0.015 |
| PUSKULLUOGLU M | 2 | 0.015 |
| PURWAR P | 2 | 0.015 |
| PURIM O | 2 | 0.015 |
| PURCELL C | 2 | 0.015 |
| PUNTAMBEKAR SP | 2 | 0.015 |
| PULA B | 2 | 0.015 |
| PUGH J | 2 | 0.015 |
| PUCCI MJ | 2 | 0.015 |
| PU Q | 2 | 0.015 |
| PROTANO MA | 2 | 0.015 |
| PRITCHARD S | 2 | 0.015 |
| PRIOR JO | 2 | 0.015 |
| PRICE T | 2 | 0.015 |
| PRIANTE AVM | 2 | 0.015 |
| PRESTON S | 2 | 0.015 |
| PRESTON D | 2 | 0.015 |
| PRESSER SJ | 2 | 0.015 |
| PRESCOTT AT | 2 | 0.015 |
| PRENEN H | 2 | 0.015 |
| PREISE D | 2 | 0.015 |
| PRAUD D | 2 | 0.015 |
| PRATI A | 2 | 0.015 |
| PRASNIKAR N | 2 | 0.015 |
| POZZO C | 2 | 0.015 |
| POZSGAI E | 2 | 0.015 |
| POWER D | 2 | 0.015 |
| POUW RE | 2 | 0.015 |
| POURFARZI F | 2 | 0.015 |
| POUPORE AK | 2 | 0.015 |
| POTENZA B | 2 | 0.015 |
| POSTLEWAIT LM | 2 | 0.015 |
| PORZSOLT F | 2 | 0.015 |
| PORZIELLA V | 2 | 0.015 |
| PORUBSKY C | 2 | 0.015 |
| PORTERO RV | 2 | 0.015 |
| PORTA M | 2 | 0.015 |
| POPESCU R | 2 | 0.015 |
| POPESCU I | 2 | 0.015 |
| POP D | 2 | 0.015 |
| POLOTZEK U | 2 | 0.015 |
| POLI MD | 2 | 0.015 |
| POLEDNAK AP | 2 | 0.015 |
| POKIESER P | 2 | 0.015 |
| POKATAEV I | 2 | 0.015 |
| POHL A | 2 | 0.015 |
| PLUM PS | 2 | 0.015 |
| PLEBANI M | 2 | 0.015 |
| PLASTARAS J | 2 | 0.015 |
| PIZZUTI L | 2 | 0.015 |
| PIZZOLATO E | 2 | 0.015 |
| PISTERS PWT | 2 | 0.015 |
| PISTERS PW | 2 | 0.015 |
| PIOTET E | 2 | 0.015 |
| PIOCHE M | 2 | 0.015 |
| PINES G | 2 | 0.015 |
| PILLAI A | 2 | 0.015 |
| PIKE IM | 2 | 0.015 |
| PIHA-PAUL SA | 2 | 0.015 |
| PIERRE A | 2 | 0.015 |
| PIEROG J | 2 | 0.015 |
| PIERCE MC | 2 | 0.015 |
| PICHLER M | 2 | 0.015 |
| PHOA SSKS | 2 | 0.015 |
| PHILLIPS TD | 2 | 0.015 |
| PHILIPS S | 2 | 0.015 |
| PHAN AT | 2 | 0.015 |
| PHAM TH | 2 | 0.015 |
| PHAM H | 2 | 0.015 |
| PFEIFFER RM | 2 | 0.015 |
| PEUGNIEZ C | 2 | 0.015 |
| PETROS WP | 2 | 0.015 |
| PETRI R | 2 | 0.015 |
| PETRELLI F | 2 | 0.015 |
| PETITFILS A | 2 | 0.015 |
| PETERSEN BT | 2 | 0.015 |
| PETERS U | 2 | 0.015 |
| PETERLI R | 2 | 0.015 |
| PETERA J | 2 | 0.015 |
| PESTALOZZI BC | 2 | 0.015 |
| PESTALOZZI B | 2 | 0.015 |
| PESENTI C | 2 | 0.015 |
| PERSSON EC | 2 | 0.015 |
| PERRONE F | 2 | 0.015 |
| PERNOT S | 2 | 0.015 |
| PERNICENI T | 2 | 0.015 |
| PEREIRA P | 2 | 0.015 |
| PEPARINI N | 2 | 0.015 |
| PENG ZH | 2 | 0.015 |
| PENG Z | 2 | 0.015 |
| PENG W | 2 | 0.015 |
| PENG JJ | 2 | 0.015 |
| PENG JH | 2 | 0.015 |
| PENG DF | 2 | 0.015 |
| PENG CY | 2 | 0.015 |
| PENG CL | 2 | 0.015 |
| PENG B | 2 | 0.015 |
| PENEL N | 2 | 0.015 |
| PENDYALA L | 2 | 0.015 |
| PENAULT-LLORCA FM | 2 | 0.015 |
| PENAULT-LLORCA F | 2 | 0.015 |
| PELLER PJ | 2 | 0.015 |
| PELLA N | 2 | 0.015 |
| PELETEIRO B | 2 | 0.015 |
| PEITZ U | 2 | 0.015 |
| PEIFFER DS | 2 | 0.015 |
| PEI T | 2 | 0.015 |
| PEETERS PH | 2 | 0.015 |
| PEETERS M | 2 | 0.015 |
| PAZO R | 2 | 0.015 |
| PAVLOV KV | 2 | 0.015 |
| PAVLOV K | 2 | 0.015 |
| PAUTHNER M | 2 | 0.015 |
| PAUN B | 2 | 0.015 |
| PAULSON EC | 2 | 0.015 |
| PAULES MJ | 2 | 0.015 |
| PATYAL B | 2 | 0.015 |
| PATTERSON A | 2 | 0.015 |
| PATSOURIS E | 2 | 0.015 |
| PATRITI A | 2 | 0.015 |
| PATNANA SV | 2 | 0.015 |
| PATEL R | 2 | 0.015 |
| PATEL N | 2 | 0.015 |
| PASZT A | 2 | 0.015 |
| PASTORINO U | 2 | 0.015 |
| PASTER BJ | 2 | 0.015 |
| PASSIGLIA F | 2 | 0.015 |
| PASSARDI A | 2 | 0.015 |
| PARSONS R | 2 | 0.015 |
| PARKER R | 2 | 0.015 |
| PARKER E | 2 | 0.015 |
| PARK YI | 2 | 0.015 |
| PARK WG | 2 | 0.015 |
| PARK W | 2 | 0.015 |
| PARK TS | 2 | 0.015 |
| PARK SW | 2 | 0.015 |
| PARK SB | 2 | 0.015 |
| PARK KW | 2 | 0.015 |
| PARK KJ | 2 | 0.015 |
| PARK KGM | 2 | 0.015 |
| PARK JO | 2 | 0.015 |
| PARK JG | 2 | 0.015 |
| PARK HW | 2 | 0.015 |
| PARK HC | 2 | 0.015 |
| PARK H | 2 | 0.015 |
| PARK EY | 2 | 0.015 |
| PARK D | 2 | 0.015 |
| PARK C | 2 | 0.015 |
| PARK BJ | 2 | 0.015 |
| PARIVAR K | 2 | 0.015 |
| PARIKH SJ | 2 | 0.015 |
| PARIKH N | 2 | 0.015 |
| PARASHAR B | 2 | 0.015 |
| PARADA H | 2 | 0.015 |
| PAPPOT H | 2 | 0.015 |
| PAPINENI S | 2 | 0.015 |
| PAPADOPOULOS KP | 2 | 0.015 |
| PANZRAM B | 2 | 0.015 |
| PANTANOWITZ L | 2 | 0.015 |
| PANG ZL | 2 | 0.015 |
| PANG J | 2 | 0.015 |
| PANDOLFINO JE | 2 | 0.015 |
| PANDITH AA | 2 | 0.015 |
| PANDEY A | 2 | 0.015 |
| PANDE A | 2 | 0.015 |
| PAN XL | 2 | 0.015 |
| PAN TS | 2 | 0.015 |
| PAN TC | 2 | 0.015 |
| PAN QZ | 2 | 0.015 |
| PAN Q | 2 | 0.015 |
| PAN K | 2 | 0.015 |
| PAN JY | 2 | 0.015 |
| PAN HM | 2 | 0.015 |
| PAN HL | 2 | 0.015 |
| PAN H | 2 | 0.015 |
| PAN CX | 2 | 0.015 |
| PALUMBO A | 2 | 0.015 |
| PALTA M | 2 | 0.015 |
| PALMQVIST A | 2 | 0.015 |
| PALMER M | 2 | 0.015 |
| PALMA DA | 2 | 0.015 |
| PALAZZO L | 2 | 0.015 |
| PALAZZO J | 2 | 0.015 |
| PALAZZO A | 2 | 0.015 |
| PAL S | 2 | 0.015 |
| PAI RK | 2 | 0.015 |
| PAGES M | 2 | 0.015 |
| PAESMANS M | 2 | 0.015 |
| PACZONA R | 2 | 0.015 |
| PAAVONEN T | 2 | 0.015 |
| OZGEN A | 2 | 0.015 |
| OZDEMIR N | 2 | 0.015 |
| OZAWA Y | 2 | 0.015 |
| OZAWA T | 2 | 0.015 |
| OZAKI Y | 2 | 0.015 |
| OZAKI T | 2 | 0.015 |
| OZAKI N | 2 | 0.015 |
| OZAKI A | 2 | 0.015 |
| OYASIJI T | 2 | 0.015 |
| OYAMA S | 2 | 0.015 |
| OYAMA M | 2 | 0.015 |
| OYA M | 2 | 0.015 |
| OVREBO K | 2 | 0.015 |
| OUCHI N | 2 | 0.015 |
| OTTO C | 2 | 0.015 |
| OTSURU M | 2 | 0.015 |
| OTSUKA S | 2 | 0.015 |
| OTANI K | 2 | 0.015 |
| OTA S | 2 | 0.015 |
| OTA K | 2 | 0.015 |
| OSUMI H | 2 | 0.015 |
| OSUGA T | 2 | 0.015 |
| OSTENDORP J | 2 | 0.015 |
| OSHIRO T | 2 | 0.015 |
| ORMECI N | 2 | 0.015 |
| ORLANDO RC | 2 | 0.015 |
| OREKHOV AN | 2 | 0.015 |
| OPITZ O | 2 | 0.015 |
| OOTA T | 2 | 0.015 |
| OOSTERHUIS JK | 2 | 0.015 |
| OOI A | 2 | 0.015 |
| ONSTAD L | 2 | 0.015 |
| ONOCHI K | 2 | 0.015 |
| ONO A | 2 | 0.015 |
| ONJI M | 2 | 0.015 |
| ONISHI Y | 2 | 0.015 |
| ONISHI K | 2 | 0.015 |
| ONIMARU M | 2 | 0.015 |
| ONGARO E | 2 | 0.015 |
| ONESTI CE | 2 | 0.015 |
| ONATE-OCANA LF | 2 | 0.015 |
| OMURO Y | 2 | 0.015 |
| OMURA T | 2 | 0.015 |
| OMURA N | 2 | 0.015 |
| OLSEN CM | 2 | 0.015 |
| OLMOS RAV | 2 | 0.015 |
| OLMOS D | 2 | 0.015 |
| OLIVEIRA J | 2 | 0.015 |
| OKURA Y | 2 | 0.015 |
| OKUMURA Y | 2 | 0.015 |
| OKUMURA N | 2 | 0.015 |
| OKUMURA M | 2 | 0.015 |
| OKUDA H | 2 | 0.015 |
| OKUBO T | 2 | 0.015 |
| OKSUZOGLU B | 2 | 0.015 |
| OKONOGI N | 2 | 0.015 |
| OKITA R | 2 | 0.015 |
| OKAZUMI SI | 2 | 0.015 |
| OKAZAKI M | 2 | 0.015 |
| OKAZAKI H | 2 | 0.015 |
| OKAYAMA T | 2 | 0.015 |
| OKAJIMA W | 2 | 0.015 |
| OKAGAWA Y | 2 | 0.015 |
| OKADA N | 2 | 0.015 |
| OKADA A | 2 | 0.015 |
| OKABAYASHI T | 2 | 0.015 |
| OKABAYASHI K | 2 | 0.015 |
| OKA H | 2 | 0.015 |
| OIKAWA M | 2 | 0.015 |
| OHURA H | 2 | 0.015 |
| OHUCHI N | 2 | 0.015 |
| OHTSUKA S | 2 | 0.015 |
| OHTAKI M | 2 | 0.015 |
| OHTA M | 2 | 0.015 |
| OHTA A | 2 | 0.015 |
| OHRI N | 2 | 0.015 |
| OHNISHI S | 2 | 0.015 |
| OHNISHI M | 2 | 0.015 |
| OHIRA T | 2 | 0.015 |
| OHBA R | 2 | 0.015 |
| OHASHI Y | 2 | 0.015 |
| OHASHI K | 2 | 0.015 |
| OH YS | 2 | 0.015 |
| OH ST | 2 | 0.015 |
| OH S | 2 | 0.015 |
| OH KS | 2 | 0.015 |
| OH IJ | 2 | 0.015 |
| OGUMA J | 2 | 0.015 |
| OGUL H | 2 | 0.015 |
| OGIYA K | 2 | 0.015 |
| OGINO S | 2 | 0.015 |
| OGAWA T | 2 | 0.015 |
| OGAWA S | 2 | 0.015 |
| OGAWA M | 2 | 0.015 |
| OGAWA H | 2 | 0.015 |
| OFFERHAUS GJ | 2 | 0.015 |
| OEDA M | 2 | 0.015 |
| ODZE R | 2 | 0.015 |
| ODA K | 2 | 0.015 |
| OCKE MC | 2 | 0.015 |
| OCHS-BALCOM HM | 2 | 0.015 |
| OCHENDUSZKO S | 2 | 0.015 |
| OCEAN AJ | 2 | 0.015 |
| OBUCHI T | 2 | 0.015 |
| OBERSCHMID B | 2 | 0.015 |
| OBERMAIER R | 2 | 0.015 |
| OBAYASHI M | 2 | 0.015 |
| OBARA K | 2 | 0.015 |
| OBAMA K | 2 | 0.015 |
| O'SULLIVAN JN | 2 | 0.015 |
| O'SULLIVAN F | 2 | 0.015 |
| O'ROURKE P | 2 | 0.015 |
| O'REILLY S | 2 | 0.015 |
| O'REILLY M | 2 | 0.015 |
| O'REILLY E | 2 | 0.015 |
| O'HANLON C | 2 | 0.015 |
| O'FARRELL DA | 2 | 0.015 |
| O'DWYER PJ | 2 | 0.015 |
| O'DWYER P | 2 | 0.015 |
| O'DONOVAN TR | 2 | 0.015 |
| O'DONNELL ME | 2 | 0.015 |
| O'DOHERTY MG | 2 | 0.015 |
| O'CALLAGHAN CJ | 2 | 0.015 |
| O'BRIEN SM | 2 | 0.015 |
| O'BRIEN S | 2 | 0.015 |
| NYEMBA V | 2 | 0.015 |
| NUSHIJIMA Y | 2 | 0.015 |
| NURKIN S | 2 | 0.015 |
| NURALINA I | 2 | 0.015 |
| NUMAJIRI T | 2 | 0.015 |
| NTOUNDA R | 2 | 0.015 |
| NOURIJELYANI K | 2 | 0.015 |
| NOURAIE SM | 2 | 0.015 |
| NOURAEE N | 2 | 0.015 |
| NOSE V | 2 | 0.015 |
| NORTHUP PG | 2 | 0.015 |
| NORGUET E | 2 | 0.015 |
| NORDESTGAARD BG | 2 | 0.015 |
| NORDENSTEDT H | 2 | 0.015 |
| NOORDZIJ W | 2 | 0.015 |
| NOORANI AS | 2 | 0.015 |
| NOOKALA P | 2 | 0.015 |
| NONGRUM HB | 2 | 0.015 |
| NOMURA AMY | 2 | 0.015 |
| NOMOTO K | 2 | 0.015 |
| NOJOMI M | 2 | 0.015 |
| NOHARA S | 2 | 0.015 |
| NOH JM | 2 | 0.015 |
| NOGUERAS-GONZALEZ GM | 2 | 0.015 |
| NOFFSINGER A | 2 | 0.015 |
| NODA Y | 2 | 0.015 |
| NOCE L | 2 | 0.015 |
| NJEI B | 2 | 0.015 |
| NIYAZI M | 2 | 0.015 |
| NIU ZY | 2 | 0.015 |
| NIU ZJ | 2 | 0.015 |
| NIU NN | 2 | 0.015 |
| NITZSCHE B | 2 | 0.015 |
| NITTA N | 2 | 0.015 |
| NITTA H | 2 | 0.015 |
| NITSCHE U | 2 | 0.015 |
| NISHIZUKA S | 2 | 0.015 |
| NISHIZAWA S | 2 | 0.015 |
| NISHISAKI H | 2 | 0.015 |
| NISHIOKA M | 2 | 0.015 |
| NISHIO T | 2 | 0.015 |
| NISHINO Y | 2 | 0.015 |
| NISHINO T | 2 | 0.015 |
| NISHINO N | 2 | 0.015 |
| NISHINO M | 2 | 0.015 |
| NISHINO K | 2 | 0.015 |
| NISHIMURA S | 2 | 0.015 |
| NISHIMURA G | 2 | 0.015 |
| NISHIMIYA H | 2 | 0.015 |
| NISHIKAWA J | 2 | 0.015 |
| NISHIGUCHI K | 2 | 0.015 |
| NISHIDA R | 2 | 0.015 |
| NISHI H | 2 | 0.015 |
| NINOMIYA Y | 2 | 0.015 |
| NINOMIYA T | 2 | 0.015 |
| NIKPOUR S | 2 | 0.015 |
| NIJJAR T | 2 | 0.015 |
| NIIDA A | 2 | 0.015 |
| NIGRI G | 2 | 0.015 |
| NIESS H | 2 | 0.015 |
| NIENARTOWICZ M | 2 | 0.015 |
| NIELSEN HO | 2 | 0.015 |
| NIEBISCH S | 2 | 0.015 |
| NIE CJ | 2 | 0.015 |
| NICCOLAI E | 2 | 0.015 |
| NI ZH | 2 | 0.015 |
| NI PZ | 2 | 0.015 |
| NGUYEN XMT | 2 | 0.015 |
| NGUYEN S | 2 | 0.015 |
| NGUYEN Q | 2 | 0.015 |
| NGUYEN GH | 2 | 0.015 |
| NGUYEN C | 2 | 0.015 |
| NGUYEN B | 2 | 0.015 |
| NGAI SM | 2 | 0.015 |
| NG Y | 2 | 0.015 |
| NG WM | 2 | 0.015 |
| NG SC | 2 | 0.015 |
| NG S | 2 | 0.015 |
| NG M | 2 | 0.015 |
| NG EK | 2 | 0.015 |
| NEWMAN F | 2 | 0.015 |
| NEUREITER D | 2 | 0.015 |
| NEUMANN UP | 2 | 0.015 |
| NEUBOECK N | 2 | 0.015 |
| NENCIONI M | 2 | 0.015 |
| NEMETH I | 2 | 0.015 |
| NELSON R | 2 | 0.015 |
| NELSON PJ | 2 | 0.015 |
| NELIS M | 2 | 0.015 |
| NEDERLOF N | 2 | 0.015 |
| NEDELCU M | 2 | 0.015 |
| NAYAR R | 2 | 0.015 |
| NAVIDI M | 2 | 0.015 |
| NAVANEETHAN U | 2 | 0.015 |
| NASSRI AB | 2 | 0.015 |
| NASELLI F | 2 | 0.015 |
| NARUKE A | 2 | 0.015 |
| NARSULE CK | 2 | 0.015 |
| NARODITSKY I | 2 | 0.015 |
| NARIKAZU B | 2 | 0.015 |
| NAOTA H | 2 | 0.015 |
| NANCARROW D | 2 | 0.015 |
| NAMIN BM | 2 | 0.015 |
| NAMASIVAYAM V | 2 | 0.015 |
| NAM SJ | 2 | 0.015 |
| NAM CM | 2 | 0.015 |
| NALINI Y | 2 | 0.015 |
| NAKO Y | 2 | 0.015 |
| NAKAZAWA M | 2 | 0.015 |
| NAKAZAWA E | 2 | 0.015 |
| NAKAYAMA S | 2 | 0.015 |
| NAKAU M | 2 | 0.015 |
| NAKASHIMA H | 2 | 0.015 |
| NAKANISHI T | 2 | 0.015 |
| NAKAMURA J | 2 | 0.015 |
| NAKAMURA F | 2 | 0.015 |
| NAKAJO M | 2 | 0.015 |
| NAKAJI Y | 2 | 0.015 |
| NAKAI Y | 2 | 0.015 |
| NAKAI S | 2 | 0.015 |
| NAKAI M | 2 | 0.015 |
| NAKAHARA A | 2 | 0.015 |
| NAKAGI M | 2 | 0.015 |
| NAKAGAWA A | 2 | 0.015 |
| NAKAE H | 2 | 0.015 |
| NAJMEDDIN A | 2 | 0.015 |
| NAJARIAN S | 2 | 0.015 |
| NAIR CK | 2 | 0.015 |
| NAINI BV | 2 | 0.015 |
| NAING A | 2 | 0.015 |
| NAIK H | 2 | 0.015 |
| NAH BS | 2 | 0.015 |
| NAGPAL K | 2 | 0.015 |
[truncated: 87,947 more chars]
